# Target and Nontarget Screening to Support Capacity Scaling for Substance Use Assessment through a Statewide Wastewater Surveillance Network in New York

**DOI:** 10.1021/acs.est.4c01251

**Published:** 2024-05-01

**Authors:** Emily J. Vogel, Milagros Neyra, David A. Larsen, Teng Zeng

**Affiliations:** † Department of Civil and Environmental Engineering, Syracuse University, 151 Link Hall, Syracuse, New York 13244, United States; ‡ Department of Public Health, Syracuse University, 444 White Hall, Syracuse, New York 13244, United States

**Keywords:** WBE, high-resolution mass spectrometry, nontargeted analysis, psychoactive substances, sewer

## Abstract

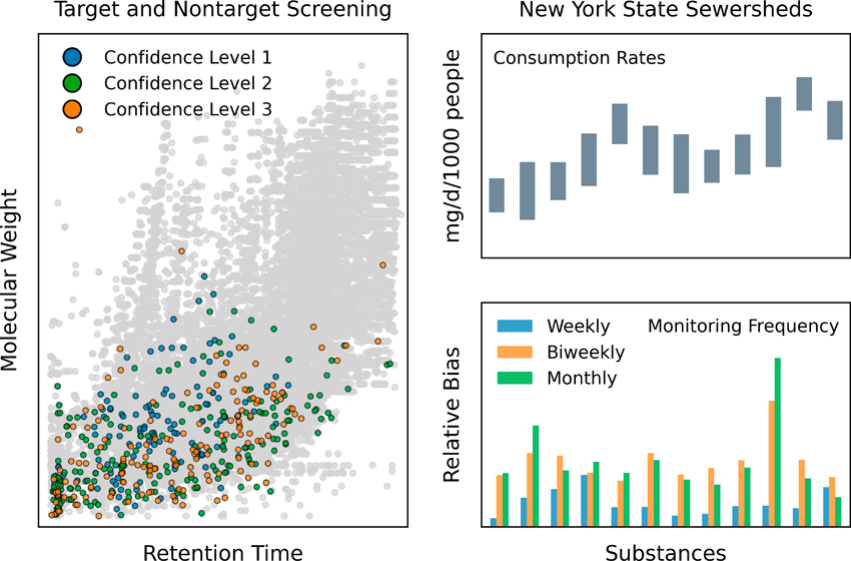

Wastewater-based epidemiology (WBE) has been widely implemented around the world as a complementary tool to conventional surveillance techniques to inform and improve public health responses. Currently, wastewater surveillance programs in the U.S. are evaluating integrated approaches to address public health challenges across multiple domains, including substance abuse. In this work, we demonstrated the potential of online solid-phase extraction coupled with liquid chromatography–high-resolution mass spectrometry to support targeted quantification and nontargeted analysis of psychoactive and lifestyle substances as a step toward understanding the operational feasibility of a statewide wastewater surveillance program for substance use assessment in New York. Target screening confirmed 39 substances in influent samples collected from 10 wastewater treatment plants with varying sewershed characteristics and is anticipated to meet the throughput demands as the statewide program scales up to full capacity. Nontarget screening prioritized additional compounds for identification at three confidence levels, including psychoactive substances, such as opioid analgesics, phenethylamines, and cathinone derivatives. Consumption rates of 12 target substances detected in over 80% of wastewater samples were similar to those reported by previous U.S.-based WBE studies despite the uncertainty associated with back-calculations. For selected substances, the relative bias in consumption estimates was sensitive to variations in monitoring frequency, and factors beyond human excretion (e.g., as indicated by the parent-to-metabolite ratios) might also contribute to their prevalence at the sewershed scale. Overall, our study marks the initial phase of refining analytical workflows and data interpretation in preparation for the incorporation of substance use assessment into the statewide wastewater surveillance program in New York.

## Abstract 2

High-throughput quantification and nontargeted analysis enabled by liquid chromatography−high-resolution mass spectrometry support the scaling of a statewide wastewater surveillance program for substance use assessment.

## Introduction

Wastewater-based epidemiology (WBE) involves the systematic analysis of untreated wastewater taken from sewer infrastructure to extract chemical signatures and/or biological markers and therefore represents a versatile tool for collecting community-wide information on various aspects of public health.^1^ Many wastewater surveillance programs have been established or expanded in the U.S. and globally since the COVID-19 pandemic to track the emergence and circulation of SARS-CoV-2 variants.^2^ For instance, the U.S. Centers for Disease Control and Prevention (CDC) launched the National Wastewater Surveillance System with partner agencies in September 2020 to monitor the spatiotemporal trends of SARS-CoV-2 viral activity levels and, more recently, the occurrence of monkeypox viral DNA, in wastewater.^3^ In New York State (NYS), a collaborative wastewater surveillance network was also established to support pandemic management^4^ and has expanded to cover 15.3 million residents (approximately 80% of the state’s population).^5^ With the COVID-19 pandemic subsiding, there is increasing momentum to utilize the existing wastewater surveillance infrastructure in NYS and nationwide for assessing the impact of the opioid epidemic^6^ and other substance use disorders given the rising number of fatal overdoses and nonfatal intoxications resulting from drug abuse in the U.S.^7,8^

Over the past decade, wastewater surveillance in Europe,^9^ Australia,^10^ and Canada^11^ has proven successful in providing large-scale spatiotemporal data sets on population-level consumption of opioids, amphetamines, cocaine, cannabis, and other substances of concern. Wastewater surveillance in these regions has also demonstrated its potential to function as a warning system for detecting the emergence of new psychoactive substances despite their constantly changing profiles and lower prevalence of use compared to traditional drugs of abuse.^12,13^ To date, most WBE studies in the U.S. have focused on assessing the mass loads and/or consumption rates of priority opioids and stimulants,^14,15^ therapeutic drugs,^16,17^ and lifestyle chemicals,^18,19^ although recent efforts have sought to investigate the occurrence of emerging psychoactive substances.^13,20^ Taken together, these studies have gathered important baseline data on substance consumption by diverse communities in the U.S. and underscored the opportunity to leverage ongoing or planned wastewater surveillance initiatives in supporting substance use assessment. From a practical standpoint, pivoting current wastewater surveillance initiatives, particularly those with broad spatiotemporal sampling regimes, to incorporate substance use assessment requires a phased approach to evaluate operational feasibility before the programs are scaled to reach their full potential.

Our primary objective of this study was to develop an integrated analytical framework that not only streamlines the routine, high-throughput quantification of target substances to ensure timely data sharing with stakeholders (e.g., health departments) and the public but also enables the qualitative screening of nontargeted analogs or derivatives that share similar structural cores with known or emerging substances of concern. To this end, we developed a target screening method based on online solid-phase extraction (SPE) coupled with liquid chromatography–high-resolution mass spectrometry (LC-HRMS) to quantify commonly targeted psychoactive and lifestyle substances (including their metabolites) in influent samples collected from 10 wastewater treatment plants (WWTPs) within the NYS wastewater surveillance network. Given the vast amount of chemical data embodied in untreated wastewater, we further developed a nontarget screening workflow to prioritize additional compounds, including new psychoactive substances, for identification at different confidence levels through mass spectral library searching complemented by the simultaneous filtering of diagnostic fragment ions and neutral losses extracted from the forensic toxicology literature. To place our work within the broader context of WBE, we also (i) estimated the population-level consumption rates of the 12 most frequently detected target substances for comparison with those previously measured for U.S. communities, (ii) evaluated the sensitivity of relative bias in substance consumption estimates to variations in monitoring frequency, and (iii) examined the parent-to-metabolite ratios of selected substances to assess the importance of human excretion relative to other sources in contributing to substance loads entering WWTPs. Our study did not aim to map substance consumption patterns in a spatiotemporally resolved manner or to establish a standardized protocol for substance use monitoring; instead, it served to explore the operational feasibility in anticipation of the growing capacity of wastewater surveillance for substance use assessment in the U.S.

## Materials and Methods

### Chemicals and Materials

Target substances of interest (Table S1) included compounds listed in the CDC’s Opioid Polysubstance Mix Kit as well as additional psychoactive drugs and lifestyle chemicals frequently monitored in previous WBE studies. High-purity native standards of target substances (*n* = 51) and isotope-labeled internal standards (ILIS; *n* = 42) were purchased from Sigma-Aldrich, Toronto Research Chemicals, and C/D/N Isotopes.

### Sample Collection

Over the study period, 24 h flow-proportional composite influent samples were collected at mixed intervals (e.g., twice per week on weekdays) over multiple months between 2021 and 2022 from 10 WWTPs with an average design capacity of 9.46 × 10^3^ to 3.19 × 10^5^ m^3^/day (i.e., 2.5 to 84.2 million gallons per day), an estimated sewer transit time of 0.6 ± 1.3 to 4.1 ± 2.8 h, and a service population of 3076 to 242,377 (extracted from the U.S. Census Bureau’s American Community Survey 2017–2021 5-Year Data^21^). Complete details of the sociodemographic attributes, health indicators, and opioid burdens of sewershed populations are summarized in Table S2. Samples were shipped overnight to SUNY Upstate Medical University for SARS-CoV-2 analysis and were stored at −80 °C until they were transferred to Syracuse University for substance analysis. General operational (e.g., flow rates) and hydrochemical parameters (e.g., 5-day biochemical oxygen demand (BOD_5_), 5-day carbonaceous BOD (CBOD_5_), total Kjeldahl nitrogen (TKN), and ammonia nitrogen (NH_3_–N)) were provided by the WWTPs.

### Sample Analysis

Wastewater samples were spiked with a mixture of 42 ILIS (400 ng/L each), filtered by 0.22 μm polyethersulfone syringe filters, and analyzed in duplicate by a Thermo Scientific TriPlus RSH autosampler and liquid handling system hyphenated with a Vanquish Horizon ultrahigh-performance liquid chromatograph and an Orbitrap Exploris 240 quadrupole-Orbitrap mass spectrometer. Briefly, 1 mL of filtered sample was loaded from a 5 mL stainless-steel sample loop onto a Hypersil GOLD aQ C18 trap column (20 × 2.1 mm i.d., 12 μm) at 1 mL/min for preconcentration and extraction, and the trap column was subsequently washed with LC-MS grade water, followed by elution using the analytical pump gradient. Chromatographic separation was performed on a Hypersil GOLD aQ C18 analytical column (100 × 2.1 mm, 1.9 μm; preceded with a 10 × 2.1 mm guard cartridge) running LC-MS-grade water and methanol (acidified with 0.1% v/v formic acid) as the mobile phases at a flow rate of 200 μL/min and a column temperature of 35 °C for 32 min. Mass spectrometric analysis was conducted in positive and negative electrospray ionization modes. External mass calibration was performed using the Pierce FlexMix calibration solution. Internal mass calibration was activated through EASY-IC (fluoranthene) lock mass during data acquisition. Full-scan mass spectra were acquired from 100 to 1000 Da with a mass resolution of 120,000 at *m*/*z* 200. Full-scan triggered data-dependent tandem mass (dd-MS2) spectra were acquired for targeted precursor ions in the inclusion list or for the five most intense precursor ions (excluding those registered in the exclusion list) with a mass resolution of 15,000 at *m*/*z* 200 by higher energy collisional dissociation across five normalized collision energies ranging from 15 to 75%. Complete details of instrument settings and method parameters are provided in Tables S3–S5.

Calibration standards (i.e., prepared by spiking LC-MS grade water with 1–5000 or 500–25,000 ng/L of target substances and 400 ng/L of ILIS as a mixture) were run with each sample sequence. Continuous check standards (i.e., prepared by spiking deionized water with 400 ng/L of target substances and ILIS as a mixture) and procedural blanks were run every 10 samples to monitor any drift in instrument performance or carryover. Quality control samples (i.e., prepared by spiking pooled wastewater samples with 400 or 4000 ng/L of target substances and ILIS as a mixture) were also analyzed alongside nonspiked controls in triplicate within 1 day to evaluate intraday precision and accuracy and over 3 days to assess interday precision and accuracy. Complete details of the online SPE-LC-HRMS method performance (e.g., intraday/interday accuracy, intraday/interday precision, recoveries, and limits of quantification (LOQs) in wastewater) are summarized in Table S6.

### Target and Nontarget Screening

Target screening was conducted using *TraceFinder 5.2 SP1* (Thermo Scientific). Calibration curves for target substances were generated by 1/*x*- or 1/*x*^2^-weighted linear or quadratic regression. Target substances were confirmed by verifying their chromatographic retention times and dd-MS2 spectra against those of the reference standards. Concentrations of substances detected in more than 50% of wastewater samples were quantified with reference to matching (i.e., structurally identical) ILIS or nonmatching ILIS with similar retention times. Complete details of *TraceFinder* method settings are provided in Table S7.

Nontarget screening was conducted using *Compound Discoverer 3.3 SP2* (Thermo Scientific) with a node-based workflow consisting of spectrum processing (e.g., align retention times), compound detection (e.g., group compounds and fill gaps), peak area refinement (e.g., apply QC correction and mark background compounds), compound identification (e.g., search *mzCloud*, search *mzVault*, search *ChemSpider*, and search mass lists), and compound scoring (e.g., compound class scoring and search neutral losses). To extend library search beyond *mzCloud* and *MassBank*,^22,23^ the search *mzVault* node was set to import the *High-Resolution Mass Spectral Libraries for Opioid Analysis* curated by the CDC^24^ and the HighResNPS consensus library (October 2023 version).^25^ Given that psychoactive substances typically contain characteristic structural cores,^26^ the compound class scoring node was implemented to enable the filtering of diagnostic fragment ions for fentanyl analogs, synthetic cannabinoids, synthetic cathinones, phenyl-substituted phenethylamines, arylcyclohexylamines, and indolealkylamines (e.g., lysergamides and tryptamines).^27^ To complement class coverage scoring, the search neutral loss node was also configured to enable the simultaneous filtering of neutral losses commonly observed for these substance classes.^27^ With this workflow, mass spectral features that met all the following criteria were prioritized for inspection: a peak rating of >5, a mass accuracy tolerance of 5 ppm, a *mzCloud* and/or *mzVault* best match score of >60, and a retention time within the 95% confidence interval predicted by the Log*P*–retention time relationship established via the analysis of 432 compounds (e.g., pharmaceuticals, pesticides, personal care and household chemicals, industrial additives, and their transformation products) covering a range of polarities (Figure S1). Mass spectral features of interest were either confirmed by reference standards (i.e., confidence level 1), identified as probable structures (i.e., level 2), or assigned as tentative candidates (i.e., level 3) when applicable.^28^ Complete details of *Compound Discoverer* node settings, the list of substances in the CDC and HighResNPS libraries, and fragment ions for selected classes of psychoactive substances are provided in Tables S8–S10.

### Substance Consumption Estimation

For target substances (*n* = 12) detected in over 80% of wastewater samples, the population-normalized mass loads (PNMLs) and consumption rates (CRs) were back-calculated^29,30^ using corresponding drug target residues (DTRs):
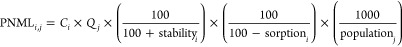
1
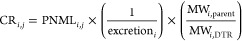
2where *C*_*i*_ is the aqueous concentration of substance *i* in each WWTP *j* influent sample averaged from duplicate measurements by online SPE-LC-HRMS (ng/L), *Q*_*j*_ is the average daily influent flow rate recorded by WWTP *j* for each sampling date (m^3^/day), stability_*i*_ is the percentage in-sample and/or in-sewer stability change of substance *i* derived from literature data (Table S11), sorption_*i*_ is the percentage of substance *i* sorbed to suspended particulate matter derived from literature data (Table S12), PNML_*i*,*j*_ is the mass load of substance *i* entering WWTP *j* normalized by the population in sewershed *j* (mg/day/1000 people) estimated for each sampling date, population_*j*_ is the *de facto* population in sewershed *j* estimated using the concentration of NH_3_-N measured in each WWTP *j* influent sample, CR_*i*,*j*_ is the consumption rate of substance *i* in sewershed *j* (mg/day/1000 people) estimated for each sampling date, excretion_*i*_ is the excretion rate of substance *i* derived from literature data (Table S13), MW_*i*,parent_ is the molecular weight of the parent compound of substance *i*, and MW_*i*,DTR_ is the molecular weight of the DTR of substance *i* (i.e., either the parent compound or its metabolite(s); Table S14). For each parameter (except for MW_*i*,parent_ and MW_*i*,DTR_), the uncertainty was characterized by a probability distribution (e.g., normal or beta distribution) as proposed by Jones *et al*.^30^

To propagate uncertainty (i.e., standard errors associated with parameter estimates) in eqs 1 and 2, Monte Carlo simulations were performed using *Colab Pro* (Google) to estimate the PNMLs and CRs of substances over 50,000 iterations.^30^ Monte Carlo-simulated PNMLs and CRs were exported as the means and 95% confidence intervals and aggregated by substance. To contextualize the impact of the monitoring frequency on CR estimates, Monte Carlo simulations were also performed for bias analysis by subsampling CR data sets for three sampling intervals of practical relevance (i.e., weekly, biweekly, and monthly). Missing CR estimates were imputed using a random forest regressor following hyperparameter optimization and 10-fold cross-validation, and the distribution similarity between raw and imputed data was assessed by the Kolmogorov–Smirnov statistic. For each substance, the relative bias in CRs calculated from reduced sample sets was averaged across WWTPs to evaluate statistical differences in deviations from baseline values (i.e., calculated based on the complete CR data sets) for the three monitoring scenarios.^31^ For a subset of target substances, the parent-to-metabolite (P:M) ratios were also calculated by dividing the PNMLs estimated using parent compounds by those estimated using corresponding metabolites to assess the relative importance of human excretion versus other sources in contributing to substance loads entering WWTPs.^32^

## Results and Discussion

### Occurrence Patterns of Target Substances

**Figure 1:**
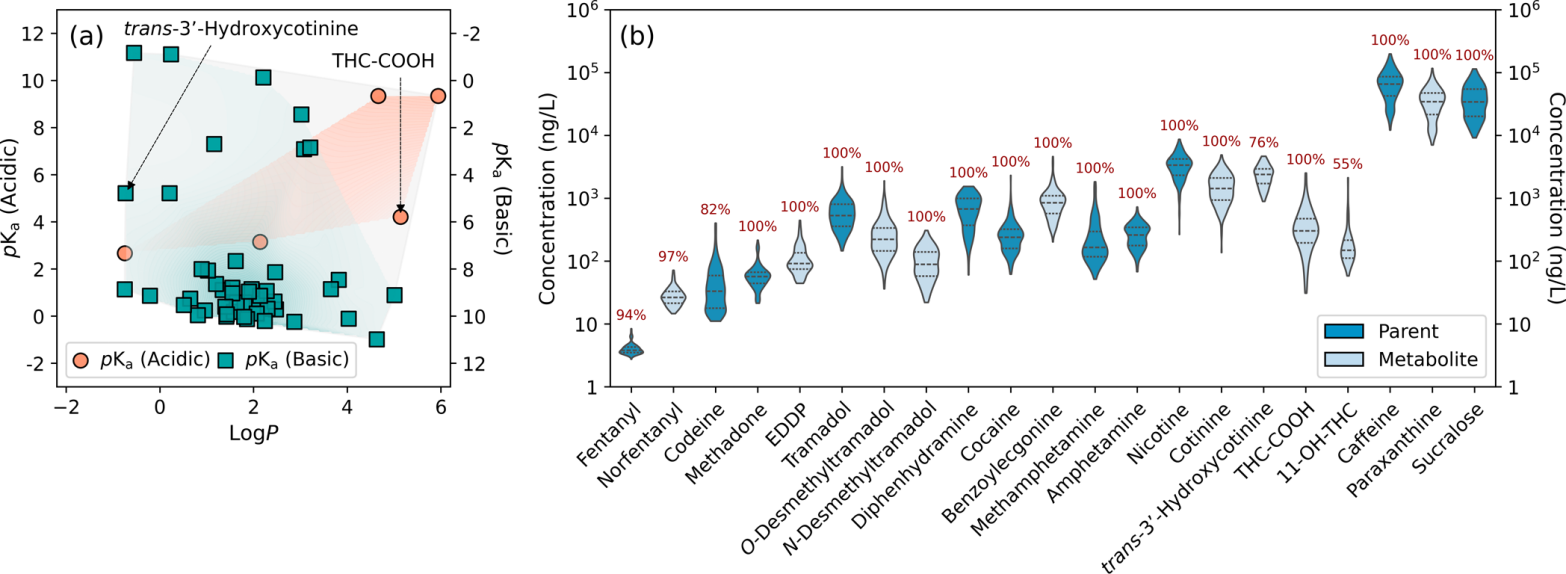
Target screening of substances in wastewater samples. (a) Target substances (*n* = 51) validated by the online SPE-LC-HRMS method developed in this work. Shaded areas highlight the analytical space defined by target substances. Predicted Log*P* and p*K*_a_ of target substances are summarized in Table S15. Strongest acidic p*K*_a_ values are plotted on the left *y*-axis, whereas strongest basic p*K*_a_ values are plotted on the right *y*-axis (reversed). Sucralose is not plotted because it lacks ionizable atoms within the range of a minimal basic p*K*_a_ of −2 and a maximum acidic p*K*_a_ of 12 as defined by *MarvinSketch 23.10.0* (ChemAxon Ltd.). (b) Target substances (*n* = 21) detected in over 50% of wastewater samples. On each violin plot, the dashed centerline marks the median, and the dotted lines bracket the interquartile range of concentrations. The percentage above each violin represents the detection frequency of each substance. Concentration ranges and detection frequencies of target substances are summarized in Table S16.

Our online SPE-LC-HRMS method enabled the high-throughput quantification of 51 substances, including 28 opioids and their metabolites, four benzodiazepines and their metabolites, and four amphetamines, as well as multiple lifestyle substances (i.e., cocaine, nicotine, cannabis, caffeine, and their metabolites). Compared to similar techniques developed by prior WBE studies (Figure S2),^33−38^ our method achieved satisfactory sensitivity (LOQs in wastewater ranging from 1.1 to 31 ng/L) and captured both basic, hydrophilic compounds such as *trans*-3′-hydroxycotinine (with a predicted strongest basic p*K*_a_ of 4.79 and a predicted Log*P* of −0.73; Figure 1a) and acidic, lipophilic compounds such as 11-nor-9-carboxy-Δ^9^-tetrahydrocannabinol (THC–COOH; with a predicted strongest acidic p*K*_a_ of 4.21 and a predicted Log*P* of 5.14; Figure 1a). On average, the recoveries of target substances ranged from 69 ± 6% for morphine-3-glucuronide to 95 ± 3% for norfentanyl, the intraday or interday precision fell within 17 ± 10%, and the intraday or interday accuracy varied between 91 ± 11% and 125 ± 20%, respectively (Figure S3). With a method runtime of 32 min, it was feasible to analyze up to 40 samples per day with continuous check standards and procedural blanks, which should provide the throughput needed to support fixed-interval (e.g., weekly to monthly) monitoring for WWTPs participating in the NYS wastewater surveillance network (Figure S4), assuming minimal supply chain disruptions and instrument downtime.

Overall, 39 out of 51 target substances were detected and confirmed in one or more WWTP influent samples (Table S16), of which 21 occurred in over 50% of samples at concentrations ranging from low ng/L to tens of μg/L (Figure 1b). Four opioids (i.e., fentanyl, codeine, methadone, and tramadol) and their metabolites (i.e., norfentanyl, 2-ethylidene-1,5-dimethyl-3,3-diphenylpyrrolidine (EDDP), *O*-desmethyltramadol, and *N*-desmethyltramadol) were detected in 82–100% of the samples. Furthermore, the concentrations of these four opioids and their metabolites were positively correlated with the concentration of diphenhydramine (Spearman’s ρ = 0.433–0.894; *p* < 0.0001), which is an over-the-counter H1 antihistamine commonly combined with opioids as an adulterant.^39^ Two amphetamines (i.e., amphetamine and methamphetamine) as well as cocaine and its metabolite benzoylecgonine were detected in 100% of the samples. Amphetamine might be excreted as a minor metabolite of methamphetamine,^40^ although their concentrations exhibited a much weaker correlation (Spearman’s ρ = 0.418; *p* < 0.0001) than those observed for the other six parent-metabolite pairs (Spearman’s ρ = 0.786–0.974; *p* < 0.0001). Nicotine and its two metabolites (i.e., cotinine and *trans*-3′-hydroxycotinine) were detected in 76–100% of the samples, whereas the two metabolites of cannabis (i.e., THC–COOH and 11-hydroxy-Δ^9^-tetrahydrocannabinol (11-OH-THC)) were detected in 100 and 55% of the samples, respectively. Caffeine, its metabolite paraxanthine, and sucralose were detected in every sample at the highest median concentrations of 64.9, 34.6, and 33.8 μg/L, respectively, as expected from their widespread consumption among the U.S. population.^41,42^ Eighteen other target substances (i.e., meperidine, normeperidine, norcodeine, hydrocodone, morphine, oxycodone, noroxycodone, dihydromorphine, buprenorphine, norbuprenorphine, naloxone, alprazolam, α-hydroxyalprazolam, diazepam, nordiazepam, norcocaine, 3,4-methylenedioxymethamphetamine, and 3,4-methylenedioxyamphetamine) were also confirmed in wastewater samples but not quantified due to their low detection frequencies (i.e., 0.4–10.9%).

### Nontarget Screening beyond Target Substances

**Figure 2:**
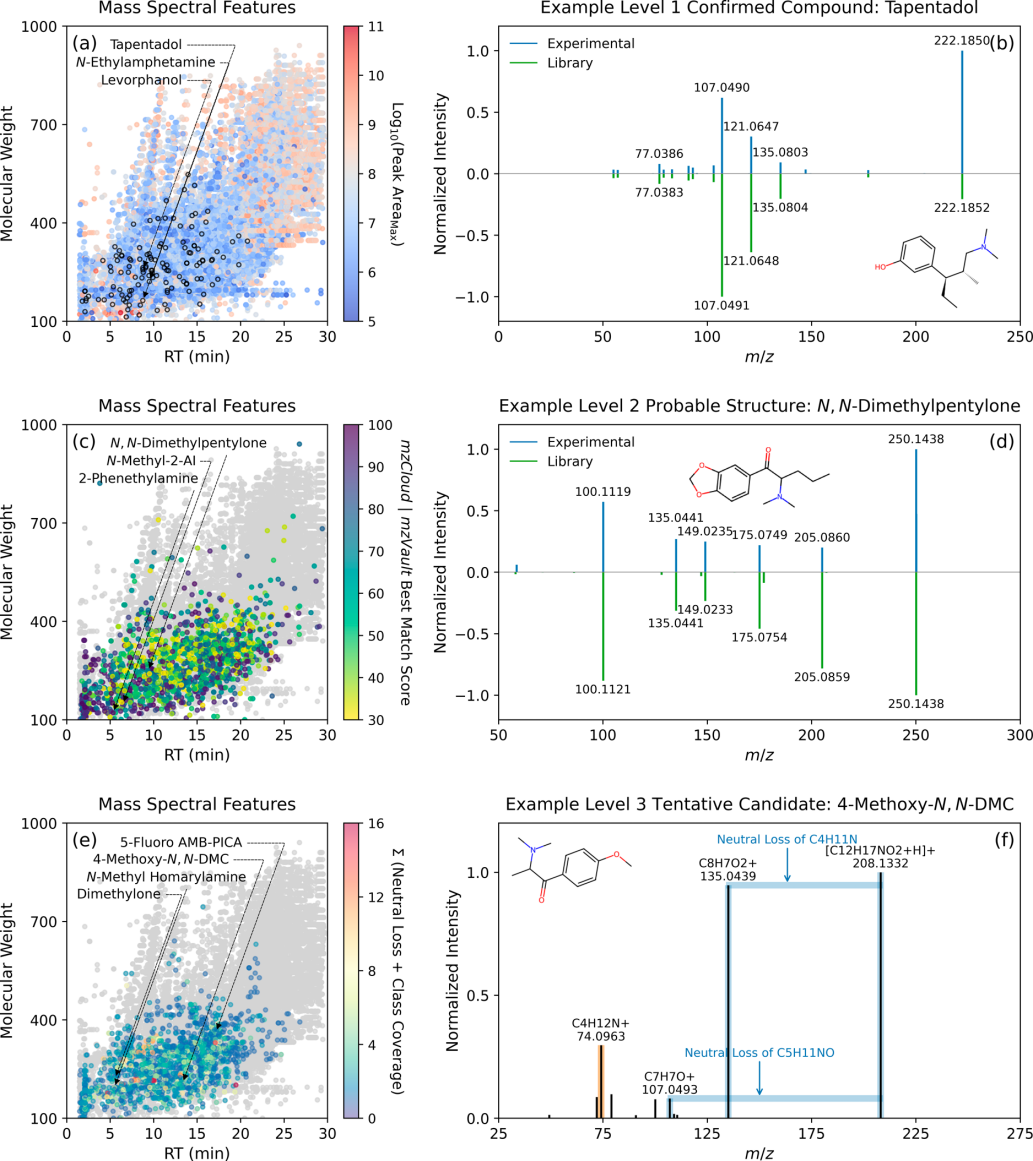
Nontarget screening of substances in wastewater samples. (a) Mass spectral features (nonredundant) with a range of molecular weights, retention times, and peak intensities. The color bar measures the maximum peak areas of features on a logarithmic scale. Circles with black outline represent mass spectral features (*n* = 125, including 39 confirmed by target screening) confirmed at confidence level 1 by reference standards (Table S17). (b) Head-to-tail plot of experimental (top) and library (bottom) dd-MS2 spectra of tapentadol with additional fragmentation information provided in Table S20. (c) Mass spectral features with a range of *mzCloud* and/or *mzVault* best match scores. The color bar indicates the *mzCloud* or *mzVault* library best match score. (d) Head-to-tail plot of experimental (top) and library (bottom) dd-MS2 spectra of *N*,*N*-dimethylpentylone with additional fragmentation information provided in Table S23. (e) Mass spectral features with a range of diagnostic fragment ions and neutral losses present in the dd-MS2 spectra. The color bar measures the total number of diagnostic fragment ions and neutral losses. (f) Experimental dd-MS2 spectrum of mass spectral feature C_12_H_17_NO_2_ assigned tentatively as 4-methoxy-*N,N*-dimethylcathinone or its isomer based on the diagnostic fragment ions and neutral losses as detailed in Table S26.

Nontargeted analysis of wastewater samples led to the detection of nonredundant mass spectral features^43^ spanning a wide range of molecular weights, polarities, and peak intensities (Figure 2a). Of these mass spectral features, 86 were further confirmed at level 1 by reference standards (Table S17; beyond those detected by target screening), 196 were identified at level 2 as probable structures by library matching (Table S18) and 158 were assigned level 3 as tentative candidates (Table S19), respectively. Confirmed compounds mostly consisted of over-the-counter and prescription pharmaceuticals such as anti-inflammatories (e.g., diclofenac and naproxen), antiarrhythmics (e.g., flecainide and propafenone), antiepileptics (e.g., carbamazepine and gabapentin), antidepressants (e.g., bupropion and venlafaxine), antidiabetics (e.g., metformin and sitagliptin), antihypertensives (e.g., diltiazem and valsartan), antivirals (e.g., abacavir and darunavir), and contraceptives (e.g., norgestrel) as well as insect repellents (e.g., DEET and icaridin), cosmetic ingredients (e.g., benzophenones), pesticides (e.g., triazines), and rubber-derived chemicals (e.g., benzothiazoles and benzotriazoles), again highlighting the chemical diversity of substances in sewer systems.

Three of the compounds prioritized by nontarget screening (Tables S20–S22; detected in 5.3–27% of wastewater samples) were confirmed as psychoactive substances with abuse potential. Tapentadol (Figure 2b) is a synthetic opioid analgesic structurally similar to tramadol,^44^ and its occurrence in untreated wastewater has been reported by WBE studies conducted in the Western U.S.,^45^ Australia,^46^ and Greece.^47^ Levorphanol (Figure S6) is another synthetic opioid analgesic with properties similar to those of morphine^44^ and has been identified in primary sludge extracts collected from WWTPs in Connecticut during the early months of the COVID-19 pandemic.^48^ Tapentadol and levorphanol were also detected at moderate frequencies in urban wastewater samples collected from multiple countries in a recent international collaborative study.^49^*N*-Ethylamphetamine (Figure S7) is an *N*-substituted derivative of amphetamine with lower potency and prevalence^50^ and was previously identified as a synthesis impurity of amphetamine in WWTP influent samples from a Lithuanian city following a suspected dumping event.^51^

Three of the level 2 mass spectral features (Tables S23–S25; detected in 2.9–29% of wastewater samples) were identified as probable structures of psychoactive substances through mass spectral library matching. *N*,*N*-Dimethylpentylone (also known as dipentylone; Figure 2d) is a synthetic cathinone increasingly being identified in forensic toxicology samples in the U.S.^52^ and has been detected at low ng/L levels in untreated wastewater collected on weekends or during special events in Spain.^53,54^*N*-Methyl-2-aminoindane (also known as *N*-methyl-2-AI; Figure S9) is a cyclic analog of methamphetamine,^55^ which has been tentatively identified in untreated wastewater from Poland^56^ and Greece^57^ and, more recently, quantified at a high frequency in South Korean WWTP influent samples.^58^ 2-Phenethylamine (Figure S10) is a structural motif widely presented in endogenous catecholamines and naturally occurring alkaloids;^59^ however, it is also a central nervous system stimulant and was first detected in WWTP influent samples collected from urban areas in Poland and Slovenia^56^ as part of a European-wide study and later in pooled urine and untreated wastewater collected during music festivals in Norway and Portugal, respectively.^60^

Four level 3 mass spectral features (Tables S26–S29; detected in 0.4–37% of wastewater samples) were assigned as tentative candidates of psychoactive substances following the examination of diagnostic fragment ions and neutral losses present in their dd-MS2 spectra. C_12_H_17_NO_2_ (*m*/*z* 208.1332 for [M + H]+; ΔMass = −0.32 ppm) was tentatively identified as 4-methoxy-*N*,*N*-dimethylcathinone (also known as 4-methoxy-*N*,*N*-DMC; Figure 2f) or its isomer based on the characteristic neutral losses of C_4_H_11_N (e.g., *N*,*N*-dimethylethanamine) and C_5_H_11_NO (e.g., 2-(dimethylamino)propanal) from the precursor ion to form the diagnostic (4-formylphenyl)(methylene)oxonium ion C_8_H_7_O_2_^+^ (i.e., *m*/*z* 135.0439; ΔMass = −1.11 ppm) found for methoxy cathinones^27^ and the 4-methoxybenzene-1-ylium ion C_7_H_7_O^+^ (i.e., *m*/*z* 107.0493; ΔMass = 1.40 ppm), respectively. C_12_H_15_NO_3_ (*m*/*z* 222.1124 for [M + H]+; ΔMass = −0.41 ppm) was tentatively identified as dimethylone (also known as bk-MDDMA; Figure S12) or its isomer based on the characteristic neutral losses of C_4_H_11_N and C_5_H_11_NO from the precursor ion to form the diagnostic (benzo[*d*][1,3]dioxol-5-ylmethylidyne)oxonium ion C_8_H_5_O_3_^+^ (i.e., *m*/*z* 149.0230; ΔMass = −2.28 ppm) found for methylenedioxy cathinones^27^ and the benzo[*d*][1,3]dioxol-5-ylium ion C_7_H_5_O_2_^+^ (i.e., *m*/*z* 121.0284; ΔMass = 0.08 ppm), respectively. C_11_H_15_NO_2_ (*m*/*z* 194.1174 for [M + H]+; ΔMass = −0.57 ppm) was tentatively identified as *N*-methyl homarylamine (Figure S13) or its isomer based on the characteristic neutral losses of C_2_H_7_N (e.g., dimethylamine) and C_2_H_7_N plus CO (i.e., C_3_H_7_NO) from the precursor ion that led to the formation of two diagnostic ions, 1-(benzo[*d*][1,3]dioxol-5-yl)ethan-1-ylium ion C_9_H_9_O_2_^+^ (i.e., *m*/*z* 149.0595; ΔMass = −1.68 ppm) and benzo[*d*][1,3]dioxol-5-ylmethylium ion C_8_H_7_O_2_^+^ (i.e., *m*/*z* 135.0442; ΔMass = 0.81 ppm), as typically observed for methylenedioxy phenethylamines.^27^ C_20_H_27_FN_2_O_3_ (*m*/*z* 363.2078 for [M + H]+; ΔMass = 0.01 ppm) was tentatively identified as 5-fluoro AMB-PICA (also known as MMB-2201; Figure S14) or its isomer based on the detection of the diagnostic (1*H*-indol-3-yl)(oxo)methylium ion C_9_H_6_NO^+^ (i.e., *m*/*z* 144.0440; ΔMass = −2.71 ppm) found for indole-3-carboxamide-based synthetic cannabinoids^27^ and the neutral loss of C_6_H_13_NO_2_ (e.g., methyl 2-amino-3-methylbutanoate) from the precursor ion to form the (1-(5-fluoropentyl)-1*H*-indol-3-yl)(oxo)methylium ion C_14_H_15_FNO^+^ (i.e., *m*/*z* 232.1130; ΔMass = −1.42 ppm). Overall, nontargeted analysis complemented target screening by prioritizing additional psychoactive substances for identification at confidence level 3 or higher, although reference standards are necessary to confirm probable structures and tentative candidates before quantitative analysis. Given the occurrence of tapentadol and levorphanol in multiple sewersheds, further analytical efforts are warranted to incorporate the monitoring of these two substances and their metabolites into the surveillance program.

### Consumption Estimates of Target Substances

**Figure 3:**
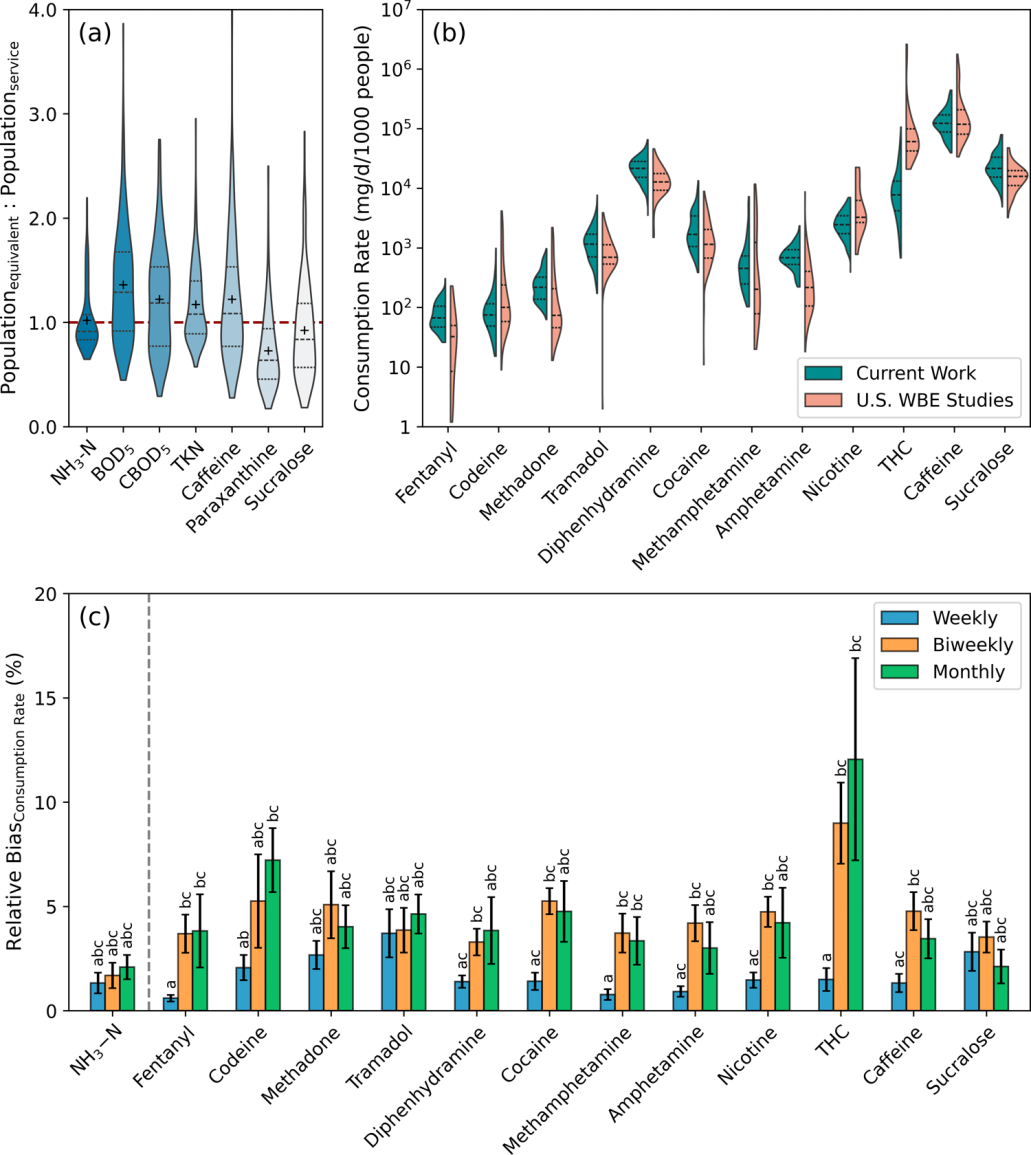
Consumption estimates of target substances detected in over 80% of wastewater samples. (a) Comparison of sewershed populations (i.e., population_equivalent_) estimated based on hydrochemical parameters (i.e., NH_3_-N, 5-day biochemical oxygen demand, 5-day carbonaceous BOD, and total Kjeldahl nitrogen) or high-consumption substances such as caffeine (and its metabolite paraxanthine) and sucralose with service populations (i.e., population_service_). On each violin plot, the dashed centerline marks the median, the “+” sign marks the mean, and the dotted lines bracket the interquartile range of population_equivalent_ to population_service_ ratios. The maroon dashed line marks a ratio of 1.0. (b) Consumption rates of substances (in mg/day/1000 people) estimated in this work compared with those reported by 15 WBE studies conducted in the U.S. between 2014 and 2024 (Table S31). On each grouped violin plot, the dashed centerline marks the median, and the dotted lines bracket the interquartile range of consumption rates. For this work, the consumption rates of six substances were estimated via their respective metabolites: methadone via EDDP, tramadol via *O*-desmethyltramadol, cocaine via benzoylecgonine, nicotine via cotinine, THC via THC–COOH, and caffeine via paraxanthine. Sewershed-specific substance consumption rates are summarized in Table S32. (c) Comparison of the relative bias for estimating the consumption rates of 12 target substances and the mass load of NH_3_-N for weekly, biweekly, and monthly monitoring scenarios, where bars sharing the same letters are not statistically different (Mann–Whitney *U* test *p* ≥ 0.05). Error bars represent the standard deviation of the relative bias.

Comparing the CRs of substances targeted in this work with literature data is challenging due to differences in sampling design, analytical methods, and assumptions made for back-calculations, each of which can introduce varying degrees of uncertainty into the final interpretation of results.^61^ For example, converting substance concentrations measured in WWTP influent samples into CRs requires estimating sewershed populations, which has long been perceived as a significant source of uncertainty among other parameters for back-calculations (e.g., excretion rates).^61^ Our analysis applied a normalization factor of 8.8 ± 1.3 g NH_3_-N/day/person to account for population dynamics in the sewersheds despite the known limitations of NH_3_-N loading for population normalization in the absence of more refined proxies (e.g., mobile network signals^62^ or concurrent census estimates^63^). Our normalization factor resembled those measured for sewersheds in New York City (i.e., 7.2 ± 0.7 g NH_3_-N/day/person)^64^ as well as those reported by WBE studies in Switzerland (i.e., 8.1 ± 0.4 g NH_3_-N/day/person)^65^ and China (i.e., 6.0–9.7 g NH_3_-N/day/person).^66,67^ On average, the ratio of NH_3_-N equivalent sewershed populations to the service populations of WWTPs was 1.02 ± 0.32 (Figure 3a), and the PNMLs and CRs of substances estimated based on NH_3_-N equivalent populations were not statistically different from those calculated using service populations (paired *t* test two-tailed *p* = 0.2905–0.4841). Three additional normalization factors were derived from BOD_5_ (i.e., 67 ± 10 g BOD_5_/day/person), CBOD_5_ (i.e., 60 ± 9 g CBOD_5_/day/person), and TKN (i.e., 12.2 ± 2.1 g TKN/day/person), but the ratios of BOD_5_, CBOD_5_, and TKN equivalent populations to service populations were more variable (i.e., 1.36 ± 0.57, 1.22 ± 0.53, and 1.17 ± 0.39, respectively) than those for NH_3_-N equivalent populations. Still, these normalization factors were comparable to those applied by previous WBE studies (e.g., 51–60 g of BOD_5_/day/person,^68,69^ 64–71 g of CBOD_5_/day/person,^64^ and 10.6–13.4 g of TKN/day/person;^64,67,69^Table S30). Given the consistent detection of caffeine, paraxanthine, and sucralose at elevated concentrations in samples, the applicability of these substances as population indicators was also evaluated for comparison to hydrochemical markers. With an average beverage caffeine intake of 165 mg/day/person^70^ and an estimated average consumption from all sources of 224 mg/day/person^41^ for the U.S. population, the ratios of caffeine equivalent populations to service populations were 0.90 ± 0.45 and 1.22 ± 0.62, respectively, which exceeded those (i.e., 0.54 ± 0.28 to 0.73 ± 0.38) calculated based on its metabolite paraxanthine (i.e., 139–188 mg/day/person assuming an 84% metabolic conversion from caffeine^71^). With an average consumption of sucralose at 18.5 to 26 mg/day/person,^19,72^ the ratios of sucralose equivalent populations to service populations ranged from 0.92 ± 0.48 to 1.30 ± 0.67. Taken together, while caffeine and sucralose loadings may serve as complementary metrics for population estimation, the analysis of NH_3_-N and other hydrochemical parameters is an integral component of WWTP operations for regulatory compliance and can readily be incorporated into the surveillance program.

Overall, the median CRs of the 12 most frequently detected target substances ranged from 66 to 1.20 × 10^5^ mg/day/1000 people (Figure 3b) and followed patterns compiled from 15 WBE studies conducted in the U.S. between 2014 and 2024 (Table S31). For codeine, tramadol, nicotine, caffeine, and sucralose, the median CRs were within ±30% of those measured for communities in other states (e.g., Arizona, Kentucky, Massachusetts, Nevada, and Washington). For fentanyl, methadone, diphenhydramine, cocaine, amphetamine, and methamphetamine, the CRs fell on the higher end of the ranges reported in the literature. Furthermore, the CR of fentanyl showed moderate to strong positive correlations with those of cocaine and methamphetamine (Spearman’s ρ = 0.552–0.830; *p* < 0.0001), which qualitatively agreed with the increasing co-consumption of fentanyl with these substances as revealed by nationwide urine drug testing.^73^ Compared to data from the literature, the median CR of THC was an order of magnitude lower, which might be attributed to nonstandardized sample preservation and extraction techniques,^74^ inconsistent excretion rates applied during back-calculations (e.g., ranging from 0.5% for THC–COOH via urinary excretion to 6.7% based on models that account for fecal excretion),^75−78^ or uncertainty resulting from the lack of measurements for particulate-bound metabolites.^40,79−81^

Over the study period, the CRs of substances varied across sewersheds (Table S32) as one might expect from shifts in the sociodemographic status, health conditions, and behavioral factors of contributing populations. For instance, the CRs of fentanyl, codeine, cocaine, methamphetamine, and THC were positively correlated with the percentages of low-income households and individuals in their early adulthood who have lower educational attainment and are unemployed but negatively correlated with the percentages of high-income households and married individuals with employment and higher educational attainment (Figure S15). No further attempts were made to interpret the covariations of CRs, if any, with the health indicators or opioid burdens of sewershed populations given the inconsistency in spatial resolutions among different data sets (e.g., sewershed level versus ZIP code level or county level). Concerted efforts to integrate wastewater-derived data with epidemiological modeling at the sewershed scale and other spatially comparable observations may overcome such limitations to identify meaningful relationships within the full-scale surveillance framework.

To investigate the effect of monitoring frequency on the relative bias in consumption estimates, the CRs of 12 target substances were calculated using reduced sample sets for three scenarios assuming weekly, biweekly, or monthly intervals and evaluated against baseline estimates derived from the complete sample sets. For eight out of the 12 substances (i.e., methadone, tramadol, diphenhydramine, cocaine, amphetamine, nicotine, caffeine, and sucralose), the relative bias in CRs derived from monthly monitoring (Figure 3c) and showed no significant difference compared to that derived from weekly and biweekly monitoring (Mann–Whitney *U* test *p* = 0.0539–0.9698), a pattern similar to that observed for NH_3_-N (Mann–Whitney *U* test *p* = 0.1859–0.7337). For fentanyl, codeine, methamphetamine, and THC, the relative bias in CRs derived from monthly monitoring was 80 ± 7% higher than that derived from weekly monitoring (Mann–Whitney *U* test *p* = 0.0028–0.0376) but did not differ significantly from those derived from biweekly monitoring (Mann–Whitney *U* test *p* = 0.2413–0.7913). Collectively, our simulations illustrated that for most of the 12 substances, the degree of deviation in CR estimates from baseline estimates was similar for weekly to monthly monitoring; however, the relative bias in CRs for fentanyl, codeine, methamphetamine, and THC exhibited a higher sensitivity to changes in monitoring frequency. Our approach did not seek to define an acceptable threshold of uncertainty or the minimum number of samples needed for representative CR estimates because quantifying the *true* loads of substances entering any WWTP requires high-frequency, longitudinal sampling.^14,82,83^ To maximize the information gained relative to the resources allocated for the surveillance program, future assessments should further quantify the effects of additional factors (e.g., weekdays versus weekends or special events, combined versus separate sewers under dry and wet weather conditions, or WWTP inlet sampling versus sewer network node sampling) on consumption estimates.

### PNML Ratios for Substances

**Figure 4:**
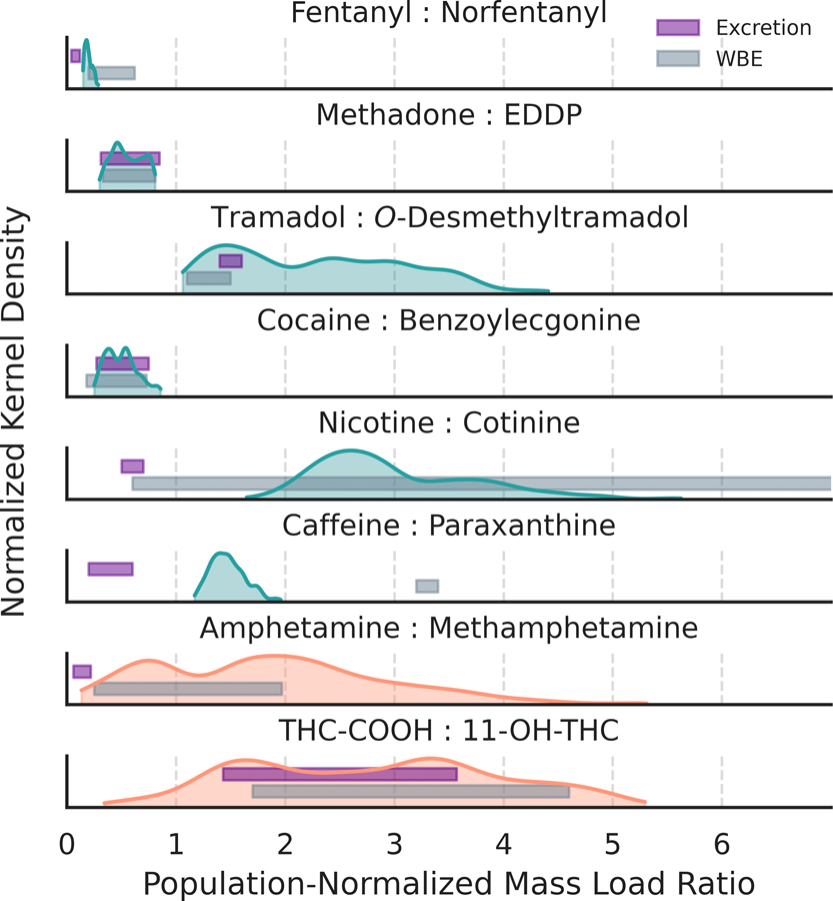
Comparison of population-normalized mass load (PNML) ratios for structurally related target substances. On each ridge plot, the indigo bar and the gray bar highlight the ranges of excretion ratios and the PNML ratios reported by previous WBE studies, respectively. Parent-to-metabolite (P:M) ratios were calculated for fentanyl (with its metabolite norfentanyl), methadone (with its metabolite EDDP), tramadol (with its metabolite *O*-desmethyltramadol), cocaine (with its metabolite benzoylecgonine), nicotine (with its metabolite cotinine), and caffeine (with its metabolite paraxanthine), respectively. Sewershed-specific ratios are summarized in Table S33.

Concurrent measurements of PNMLs for six parent–metabolite pairs of substances (i.e., fentanyl, methadone, tramadol, cocaine, nicotine, and caffeine) enabled the comparison of the P:M ratios measured in wastewater samples to those obtained from pharmacokinetic studies (Figure 4).^32^ On average, the P:M ratios for fentanyl:norfentanyl were relatively stable across sewersheds (Table S33) with a mean of 0.20 ± 0.04, which fell on the lower end of the range (i.e., 0.20–0.62) reported by a longitudinal WBE study conducted in the Midwestern U.S. communities^14^ but was higher than the urinary excretion ratio (i.e., 0.08 ± 0.04).^29^ The P:M ratios for methadone:EDDP converged at 0.54 ± 0.13 and matched the value (i.e., 0.57 ± 0.24) averaged from 24 WBE studies conducted in Europe, Australia, China, and U.S.A. as well as the excretion ratio (i.e., 0.58 ± 0.27) derived from urinary measurements.^84^ The P:M ratios for tramadol:*O*-desmethyltramadol ranged from 1.3 ± 0.2 to 2.9 ± 0.7, which encompassed the urinary excretion ratio (i.e., 1.5 ± 0.1)^29^ and the ratio (i.e., 1.3 ± 0.2) documented by a WBE study conducted in Leuven, Belgium.^85^ The P:M ratios for cocaine:benzoylecgonine varied from 0.41 ± 0.08 to 0.64 ± 0.10, consistent with those (i.e., 0.18–0.73) reported by WBE studies conducted in a Southwestern U.S. university campus^86^ and communities in Kentucky^16,87^ as well as the range of urinary excretion ratios (i.e., 0.27–0.75).^88,89^ The P:M ratios for nicotine:cotinine (i.e., 2.4 ± 0.4 to 4.2 ± 0.7) were less variable than the range (i.e., 0.6–9.2) observed by a WBE study conducted in communities with both separate and combined sewers in the Northeastern and Western U.S.A.^18^ but far exceeded the urinary excretion ratio (i.e., 0.6 ± 0.1).^66^ The P:M ratios for caffeine:paraxanthine (i.e., 1.5 ± 0.2) fell between the ratio (i.e., 3.3 ± 0.1) measured by a SARS-CoV-2 WBE study conducted in Missouri^90^ and the urinary excretion ratio (i.e., 0.4 ± 0.2).^91^ Overall, the P:M ratios for fentanyl, tramadol, nicotine, and caffeine provided an initial sign of nonconsumed parent compounds entering sewer systems through additional inputs; in contrast, the P:M ratios for methadone and cocaine suggested human excretion as the primary factor contributing to their prevalence at the sewershed level but in-sewer fate modeling would be required to assess the impacts of dynamic sewer conditions on P:M ratios.

Considering the possibility of amphetamine being excreted into sewer systems following methamphetamine consumption,^92^ the PNML ratios for amphetamine and methamphetamine were also calculated even though the amphetamine:methamphetamine ratio did not necessarily differentiate between excretion and other pathways like the P:M ratios above. On average, the amphetamine:methamphetamine ratios for five of the sewersheds ranged from 1.6 ± 0.2 to 4.1 ± 0.5, indicating a higher consumption of amphetamine than methamphetamine with reference to ratios (i.e., 0.20–3.4) observed in 17 WBE studies across five continents.^93^ Conversely, the ratios for the remaining half of sewersheds were within the range of 0.27 ± 0.03 to 0.83 ± 0.10, pointing to comparable consumption of amphetamine and methamphetamine.^93^ Taking into account the solid–liquid partitioning of cannabis biomarkers,^79,81^ the PNML ratios for THC–COOH and 11-OH-THC ranged from 1.7 ± 0.7 to 2.8 ± 0.6 and approached the values calculated for urine and feces (i.e., 2.5 ± 1.1)^81^ and unfiltered wastewater (i.e., 1.7–4.6).^80^ To what extent this ratio may serve as a measure of the primary route by which THC enters sewer systems requires more clinical research to refine the excretion profiles of THC metabolites (e.g., the amount of THC–COOH excreted across a range of product types, consumption methods and frequencies, and co-consumption effects),^92^ along with additional field and experimental investigation into their transformations and partitioning (e.g., the fraction of fecally excreted THC–COOH dissolved in wastewater) during in-sewer transit, sampling, and storage.^80^

### Implications and Limitations

This work demonstrated the potential of online SPE-LC-HRMS for high-throughput quantification and nontargeted analysis in support of substance use assessment through a statewide wastewater surveillance network. Our target screening method covered a panel of acidic, lipophilic, and basic hydrophilic compounds and is anticipated to meet the throughput requirements for weekly-to-monthly monitoring of influent samples from WWTPs participating in the NYS wastewater surveillance network. Going forward, targeted method development might consider combining mixed-bed multilayer online SPE^94^ or less-selective enrichment techniques (e.g., vacuum-assisted evaporative concentration^95^) with alternative chromatographic modes (e.g., hydrophilic interaction^96^ or mixed-mode^97^ liquid chromatography) to broaden the analytical coverage. Our nontargeted analysis applied filtering for diagnostic fragment ions and characteristic neutral losses to prioritize the identification of additional psychoactive substances of concern, and a logical next step would be to explore the use of mass defect filtering for the selective profiling of specific substance classes (e.g., synthetic cannabinoids^98^ and fentanyl analogs^99^) as well as the practicality of *in silico* mass spectral prediction models (e.g., domain-specific CFM-ID^100^) for high-confidence structural annotation of newly emerging or unknown psychoactive substances absent from mass spectral libraries. Complementary workflows, particularly those incorporating ion mobility separation, should also be implemented to remove mass spectral interferences and enhance structural elucidation when LC-HRMS alone cannot definitively resolve isobaric or isomeric substances in wastewater matrices.^101^ Our consumption estimates relied on back-calculations that were highly sensitive to uncertainties associated with the in-sample stability, in-sewer transformation, partitioning behavior, and the excretion profiles of substances, as well as the choice of population biomarkers.^61^ Furthermore, generating such estimates would likely be impractical for substances without known metabolic pathways and excretion rates, or those not excreted in detectable quantities in wastewater.^101^ Given the variability in parent-to-metabolite ratios, the application of diagnostic tools like enantiomeric analysis^102^ is warranted to differentiate the relative importance of human excretion versus other contributing sources to the presence of substances in sewer systems before performing consumption estimates. Overall, our study supports the operational feasibility of a statewide wastewater surveillance program for substance use assessment in New York and identifies several limitations and opportunities that could inform the implementation of similar initiatives in other regions of the U.S.

## Supporting Information Available

Characteristics of WWTPs and sewershed populations; online SPE-LC-HRMS method parameters for screening and quantification of substances; map of municipal WWTPs with an average design hydraulic flow of ≥1 MGD in NYS; stability factors, sorption data, and excretion rates of target substances from the literature; concentration ranges and detection frequencies of target substances; fragmentation information for substances identified at confidence levels 1, 2, and 3 through nontarget screening; population normalization factors; consumption rates of substances; and population-normalized mass load ratios for substances (PDF)
