# Supplementary material for: Target and Nontarget Screening to Support Capacity Scaling for Substance Use Assessment through a Statewide Wastewater Surveillance Network in New York
Source: Environ Sci Technol. 2024 May 1;58(19):8518–30. doi: 10.1021/acs.est.4c01251 (PMC11097395; doi:10.1021/acs.est.4c01251)
Supplement: Supplementary file 1 — es4c01251_si_001.pdf [file es4c01251_si_001.pdf]

Supporting Information for

Target and Nontarget Screening to Support Capacity Scaling for Substance Use Assessment through a Statewide Wastewater Surveillance Network in New York

Emily J. Vogel[1], Milagros Neyra[2], David A. Larsen[2], Teng Zeng[1]*

[1]Department of Civil and Environmental Engineering, Syracuse University, 151 Link Hall, Syracuse, New York 13244, United States

[2]Department of Public Health, Syracuse University, 444 White Hall, Syracuse, New York 13244, United States

*Corresponding Author: Teng Zeng: Email: tezeng@syr.edu; Phone: +1-315-443-1099

(Total 161 pages, 1 text, 33 tables, 16 figures)

Table of Contents

S1. Chemicals and materials S3

S2. Characteristics of WWTPs and sewershed populations S7

S3. Screening and quantification of substances by online SPE-LC-HRMS S11

S4. Stability factors, sorption data, and excretion rates of target substances S89

S5. Target screening of substances in wastewater samples S93

S6. Nontarget screening of substances in wastewater samples S99

S7. Population normalization factors S146

S8. Consumption rates of substances S147

S9. Population-normalized mass load (PNML) ratios for substances S153

References S156

S1. Chemicals and materials

Chemicals and reagents were used as received without further purification unless otherwise noted. Methanol (MeOH; HPLC and LC-MS grade), acetonitrile (HPLC and LC-MS grade), water (H_2O; HPLC and LC-MS grade), formic acid solution (FA; ≥99.0%; LC-MS grade), ammonium hydroxide solution (≥25%; LC-MS grade), and Pierce FlexMix calibration solutions (catalog number PIA39239) were purchased from Fisher Scientific. Mobile phases for LC-HRMS analysis were prepared using LC-MS grade water, methanol, and formic acid. High-purity reference standards and isotope-labeled internal standards were purchased from Sigma-Aldrich (Cerilliant), Toronto Research Chemicals, and C/D/N Isotopes as stock solutions (1.0 mg/mL or 100 µg/mL in MeOH or acetonitrile) or solids (**Table S1**). Multi-analyte working solutions of target substances and isotope-labeled internal standards (ILIS) were prepared by diluting native standards into LC-MS grade water before instrumental analysis. Sterile 50-mL conical polypropylene centrifuge tubes, polypropylene disposable syringes, 0.22-µm Millex-GP polyethersulfone syringe filters, and 18-mm screw top headspace vials and silicone/polytetrafluoroethylene-lined magnetic screw caps were purchased from Fisher Scientific.

**Table S1.** List of native reference standards and isotope-labeled internal standards				
Compound Name	CAS	Supplier	Catalog Number	Category
Fentanyl	437-38-7	Sigma-Aldrich (Cerilliant)	F-013-1ML	Parent
Norfentanyl	1609-66-1	Sigma-Aldrich (Cerilliant)	N-031-1ML	Metabolite (Fentanyl)
Meperidine	57-42-1	Sigma-Aldrich (Cerilliant)	M-035-1ML	Parent
Normeperidine	77-17-8	Sigma-Aldrich (Cerilliant)	N-017-1ML	Metabolite (Meperidine)
Codeine	76-57-3	Sigma-Aldrich (Cerilliant)	C-006-1ML	Parent
Norcodeine	467-15-2	Sigma-Aldrich (Cerilliant)	N-005-1ML	Metabolite (Codeine)
Hydrocodone	125-29-1	Sigma-Aldrich (Cerilliant)	H-003-1ML	Parent / Metabolite (Codeine)
Norhydrocodone	5083-62-5	Sigma-Aldrich (Cerilliant)	N-053-1ML	Metabolite (Hydrocodone)
Morphine	57-27-2	Sigma-Aldrich (Cerilliant)	M-005-1ML	Parent / Metabolite (Codeine, Heroin, 6-Acetylmorphine)
Hydromorphone	466-99-9	Sigma-Aldrich (Cerilliant)	H-004-1ML	Metabolite (Morphine, Hydrocodone)
Normorphine	466-97-7	Sigma-Aldrich (Cerilliant)	N-006-1ML	Metabolite (Morphine)
Morphine-3-glucuronide (M3G)	20290-09-9	Sigma-Aldrich (Cerilliant)	M-031-1ML	Metabolite (Morphine)
Heroin	561-27-3	Sigma-Aldrich (Cerilliant)	H-038-1ML	Parent
6-Monoacetylmorphine (6-MAM)	2784-73-8	Sigma-Aldrich (Cerilliant)	A-009-1ML	Metabolite (Heroin)
Oxycodone	76-42-6	Sigma-Aldrich (Cerilliant)	O-002-1ML	Parent
Noroxycodone	57664-96-7	Sigma-Aldrich (Cerilliant)	N-011-1ML	Metabolite (Oxycodone)
Oxymorphone	76-41-5	Sigma-Aldrich (Cerilliant)	O-004-1ML	Parent / Metabolite (Oxycodone)
Noroxymorphone	33522-95-1	Sigma-Aldrich (Cerilliant)	N-012-1ML	Metabolite (Oxycodone, Oxymorphone, Naloxone)
Dihydrocodeine	125-28-0	Sigma-Aldrich (Cerilliant)	D-019-1ML	Parent / Metabolite (Hydrocodone)
Dihydromorphine	509-60-4	Sigma-Aldrich (Cerilliant)	D-033-1ML	Parent / Metabolite (Dihydrocodeine)
Desomorphine	427-00-9	Sigma-Aldrich (Cerilliant)	D-083-1ML	Parent
Buprenorphine	52485-79-7	Sigma-Aldrich (Cerilliant)	B-044-1ML	Parent
Norbuprenorphine	78715-23-8	Sigma-Aldrich (Cerilliant)	N-912-1ML	Metabolite (Buprenorphine)
(±)-Methadone	76-99-3	Sigma-Aldrich (Cerilliant)	M-007-1ML	Parent
2-Ethylidene-1,5-dimethyl-3,3-diphenylpyrrolidine (EDDP)	30223-73-5	Sigma-Aldrich (Cerilliant)	E-022-1ML	Metabolite (Methadone)
(+)-Tramadol	27203-92-5	Sigma-Aldrich (Cerilliant)	T-027-1ML	Parent
(+)-*O*-Desmethyltramadol	144830-14-8	Sigma-Aldrich (Cerilliant)	T-035-1ML	Metabolite (Tramadol)
(+)-*N*-Desmethyltramadol	75377-45-6	Sigma-Aldrich (Cerilliant)	D-023-1ML	Metabolite (Tramadol)
Naloxone	465-65-6	Sigma-Aldrich (Cerilliant)	N-004-1ML	Parent
Diphenhydramine	58-73-1	Sigma-Aldrich (Cerilliant)	D-015-1ML	Parent
Alprazolam	28981-97-7	Sigma-Aldrich (Cerilliant)	A-903-1ML	Parent
α-Hydroxyalprazolam	37115-43-8	Sigma-Aldrich (Cerilliant)	A-907-1ML	Metabolite (Alprazolam)
Diazepam	439-14-5	Sigma-Aldrich (Cerilliant)	D-907-1ML	Parent
Nordiazepam	1088-11-5	Sigma-Aldrich (Cerilliant)	N-905-1ML	Metabolite (Diazepam)
Cocaine	50-36-2	Sigma-Aldrich (Cerilliant)	C-008-1ML	Parent
Benzoylecgonine	519-09-5	Sigma-Aldrich (Cerilliant)	B-004-1ML	Metabolite (Cocaine)
Ecgonine Methyl Ester	7143-09-1	Sigma-Aldrich (Cerilliant)	E-001-1ML	Metabolite (Cocaine)
(-)-Norcocaine	18717-72-1	Sigma-Aldrich (Cerilliant)	N-003-1ML	Metabolite (Cocaine)
(±)-3,4-Methylenedioxymethamphetamine ((±)-MDMA)	42542-10-9	Sigma-Aldrich (Cerilliant)	M-013-1ML	Parent
(±)-3,4-Methylenedioxyamphetamine ((±)-MDA)	4764-17-4	Sigma-Aldrich (Cerilliant)	M-012-1ML	Parent / Metabolite (MDMA)
(±)-Methamphetamine	7632-10-2	Sigma-Aldrich (Cerilliant)	M-009-1ML	Parent
(±)-Amphetamine	300-62-9	Sigma-Aldrich (Cerilliant)	A-007-1ML	Parent / Metabolite (Methamphetamine)

Table S1. List of native reference standards and isotope-labeled internal standards (continued)				
Compound Name	CAS	Supplier	Catalog Number	Category
(-)-Nicotine	54-11-5	Sigma-Aldrich (Cerilliant)	N-008-1ML	Parent
(-)-Cotinine	486-56-6	Sigma-Aldrich (Cerilliant)	C-016-1ML	Metabolite (Nicotine)
trans-3'-Hydroxycotinine	34834-67-8	Sigma-Aldrich (Cerilliant)	H-101-1ML	Metabolite (Nicotine)
Δ^9-Tetrahydrocannabinol (THC)	1972-08-3	Sigma-Aldrich (Cerilliant)	T-005-1ML	Parent
(±)-11-nor-9-Carboxy-Δ^9-tetrahydrocannabinol (THC-COOH)	104874-50-2	Sigma-Aldrich (Cerilliant)	T-006-1ML	Metabolite (THC)
(±)-11-Hydroxy-Δ^9-tetrahydrocannabinol (11-OH-THC)	34675-49-5	Sigma-Aldrich (Cerilliant)	H-026-1ML	Metabolite (THC)
Caffeine	58-08-2	Sigma-Aldrich	C0750-5G	Parent
Paraxanthine (1,7-Dimethylxanthine)	611-59-6	Sigma-Aldrich (Cerilliant)	IMPC-051-03-1ML	Metabolite (Caffeine)
Sucralose	56038-13-2	Toronto Research Chemicals	S692500	Parent
Fentanyl-d_5	118357-29-2	Sigma-Aldrich (Cerilliant)	F-001-1ML	Isotope-Labeled Internal Standard (ILIS)
Norfentanyl-d_5	1211527-23-9	Sigma-Aldrich (Cerilliant)	N-030-1ML	Isotope-Labeled Internal Standard (ILIS)
Meperidine-d_4	53484-73-4	Sigma-Aldrich (Cerilliant)	M-036-1ML	Isotope-Labeled Internal Standard (ILIS)
Normeperidine-d_4	160227-47-4	Sigma-Aldrich (Cerilliant)	N-020-1ML	Isotope-Labeled Internal Standard (ILIS)
Codeine-d_3	70420-71-2	Sigma-Aldrich (Cerilliant)	C-005-1ML	Isotope-Labeled Internal Standard (ILIS)
Norcodeine-d_3	NA	Sigma-Aldrich (Cerilliant)	N-082-1ML	Isotope-Labeled Internal Standard (ILIS)
Hydrocodone-d_3	136765-36-1	Sigma-Aldrich (Cerilliant)	H-005-1ML	Isotope-Labeled Internal Standard (ILIS)
Norhydrocodone-d_3	NA	Sigma-Aldrich (Cerilliant)	N-054-1ML	Isotope-Labeled Internal Standard (ILIS)
Morphine-d_3	67293-88-3	Sigma-Aldrich (Cerilliant)	M-003-1ML	Isotope-Labeled Internal Standard (ILIS)
Hydromorphone-d_3	136765-37-2	Sigma-Aldrich (Cerilliant)	H-006-1ML	Isotope-Labeled Internal Standard (ILIS)
Normorphine-d_3	NA	Sigma-Aldrich (Cerilliant)	N-144-1ML	Isotope-Labeled Internal Standard (ILIS)
Morphine-d_3-3-glucuronide	136765-44-1	Sigma-Aldrich (Cerilliant)	M-017-1ML	Isotope-Labeled Internal Standard (ILIS)
Heroin-d_9	1338713-49-7	Sigma-Aldrich (Cerilliant)	H-036-1ML	Isotope-Labeled Internal Standard (ILIS)
6-Monoacetylmorphine-d_6	152477-90-2	Sigma-Aldrich (Cerilliant)	A-026-1ML	Isotope-Labeled Internal Standard (ILIS)
Oxycodone-d_3	160227-46-3	Sigma-Aldrich (Cerilliant)	O-005-1ML	Isotope-Labeled Internal Standard (ILIS)
Noroxycodone-d_3	1007844-32-7	Sigma-Aldrich (Cerilliant)	N-032-1ML	Isotope-Labeled Internal Standard (ILIS)
Oxymorphone-d_3	145225-03-2	Sigma-Aldrich (Cerilliant)	O-003-1ML	Isotope-Labeled Internal Standard (ILIS)
Buprenorphine-d_4	136781-89-0	Sigma-Aldrich (Cerilliant)	B-901-1ML	Isotope-Labeled Internal Standard (ILIS)
Norbuprenorphine-d_3	350482-19-8	Sigma-Aldrich (Cerilliant)	N-920-1ML	Isotope-Labeled Internal Standard (ILIS)
(±)-Methadone-d_3	60263-63-0	Sigma-Aldrich (Cerilliant)	M-008-1ML	Isotope-Labeled Internal Standard (ILIS)
EDDP-d_3	136765-23-6	Sigma-Aldrich (Cerilliant)	E-021-1ML	Isotope-Labeled Internal Standard (ILIS)
(+)-Tramadol-^{13}C,d_3	NA	Sigma-Aldrich (Cerilliant)	T-029-1ML	Isotope-Labeled Internal Standard (ILIS)
Diphenhydramine-d_3	170082-18-5	Sigma-Aldrich (Cerilliant)	D-017-1ML	Isotope-Labeled Internal Standard (ILIS)
Alprazolam-d_5	125229-61-0	Sigma-Aldrich (Cerilliant)	A-902-1ML	Isotope-Labeled Internal Standard (ILIS)
α-Hydroxyalprazolam-d_5	136765-24-7	Sigma-Aldrich (Cerilliant)	A-904-1ML	Isotope-Labeled Internal Standard (ILIS)
Diazepam-d_5	65854-76-4	Sigma-Aldrich (Cerilliant)	D-902-1ML	Isotope-Labeled Internal Standard (ILIS)
Nordiazepam-d_5	65891-80-7	Sigma-Aldrich (Cerilliant)	N-903-1ML	Isotope-Labeled Internal Standard (ILIS)
Cocaine-d_3	138704-14-0	Sigma-Aldrich (Cerilliant)	C-004-1ML	Isotope-Labeled Internal Standard (ILIS)
Benzoylecgonine-d_3	115732-68-8	Sigma-Aldrich (Cerilliant)	B-001-1ML	Isotope-Labeled Internal Standard (ILIS)
Ecgonine Methyl Ester-d_3	136765-34-9	Sigma-Aldrich (Cerilliant)	E-002-1ML	Isotope-Labeled Internal Standard (ILIS)
(±)-MDMA-d_5	136765-43-0	Sigma-Aldrich (Cerilliant)	M-011-1ML	Isotope-Labeled Internal Standard (ILIS)
(±)-MDA-d_5	136765-42-9	Sigma-Aldrich (Cerilliant)	M-010-1ML	Isotope-Labeled Internal Standard (ILIS)
(±)-Methamphetamine-d_8	136765-40-7	Sigma-Aldrich (Cerilliant)	M-016-1ML	Isotope-Labeled Internal Standard (ILIS)

**Table S1.** List of native reference standards and isotope-labeled internal standards (continued)				
Compound Name	CAS	Supplier	Catalog Number	Category
(±)-Amphetamine-d_{10}	169565-17-7	Sigma-Aldrich (Cerilliant)	A-038-1ML	Isotope-Labeled Internal Standard (ILIS)
(±)-Nicotine-d_4	350818-69-8	Sigma-Aldrich (Cerilliant)	N-048-1ML	Isotope-Labeled Internal Standard (ILIS)
(±)-Cotinine-d_3	110952-70-0	Sigma-Aldrich (Cerilliant)	C-017-1ML	Isotope-Labeled Internal Standard (ILIS)
trans-3'-Hydroxycotinine-d_3	159956-78-2	Sigma-Aldrich (Cerilliant)	H-108-1ML	Isotope-Labeled Internal Standard (ILIS)
(-)-Δ^9-THC-d_3	81586-39-2	Sigma-Aldrich (Cerilliant)	T-003-1ML	Isotope-Labeled Internal Standard (ILIS)
(±)-11-nor-9-Carboxy-Δ^9-THC-d_3	136844-96-7	Sigma-Aldrich (Cerilliant)	T-004-1ML	Isotope-Labeled Internal Standard (ILIS)
(±)-11-Hydroxy-Δ^9-THC-d_3	362044-74-4	Sigma-Aldrich (Cerilliant)	H-041-1ML	Isotope-Labeled Internal Standard (ILIS)
Caffeine-d_9	72238-85-8	C/D/N Isotopes	D-5972	Isotope-Labeled Internal Standard (ILIS)
Sucralose-d_6	1459161-55-7	Toronto Research Chemicals	S692502	Isotope-Labeled Internal Standard (ILIS)

S2. Characteristics of WWTPs and sewershed populations

Table S2. Characteristics of WWTPs and sewershed populations

WWTP	A	B	C	D	E	F	G	H	I	J
Ave. design capacity (MGD)	84.2	9.0	7.0	3.0	10.0	6.5	3.75	2.8	2.5	4.8
Sewershed area (km^2)	322.3	125.6	40.5	80.7	155.7	127.4	27.2	10.7	29.8	36.6
Sewer transit time (h)[a]	2.5 ± 1.2	1.9 ± 0.8	1.4 ± 0.6	4.1 ± 2.8	3.5 ± 1.6	2.3 ± 1.1	1.5 ± 0.7	0.6 ± 0.3	1.5 ± 0.6	1.2 ± 0.4
Service population[b]	242377 ± 4917	30613 ± 1901	25965 ± 1693	16168 ± 1327	50727 ± 2124	39352 ± 1805	9207 ± 1075	6374 ± 713	3076 ± 642	10794 ± 921
Population density (km^{-2})	752 ± 15	244 ± 15	641 ± 42	200 ± 16	326 ± 14	309 ± 14	339 ± 40	596 ± 67	103 ± 22	295 ± 25
5-Year American Community Survey (Block Group)[b]										
**Gender**										
Male	47.7 ± 1.2%	48.4 ± 3.6%	49.1 ± 3.7%	48.5 ± 4.0%	50.0 ± 2.5%	50.0 ± 3.0%	49.6 ± 6.9%	45.1 ± 6.3%	69.2 ± 17.0%	48.0 ± 5.0%
Female	52.3 ± 1.2%	51.6 ± 3.6%	50.9 ± 3.8%	51.5 ± 5.1%	50.0 ± 2.3%	50.0 ± 2.6%	50.4 ± 6.0%	54.9 ± 7.5%	30.8 ± 8.0%	52.0 ± 5.5%
**Age Group (years)**										
<18	21.6 ± 1.8%	23.5 ± 6.3%	23.1 ± 6.1%	18.8 ± 6.1%	21.2 ± 3.9%	22.0 ± 4.3%	19.6 ± 9.8%	19.7 ± 9.8%	20.1 ± 16.9%	20.1 ± 8.3%
18–24	12.7 ± 1.4%	6.7 ± 3.3%	9.1 ± 3.6%	5.9 ± 4.0%	6.2 ± 2.1%	6.3 ± 2.5%	9.4 ± 6.3%	6.7 ± 6.6%	9.8 ± 10.1%	6.4 ± 5.2%
25–34	14.5 ± 1.1%	11.9 ± 2.9%	13.2 ± 3.6%	10.1 ± 3.0%	13.0 ± 2.0%	9.3 ± 2.2%	10.0 ± 4.6%	12.7 ± 5.4%	18.4 ± 12.7%	13.6 ± 4.8%
35–44	11.3 ± 0.9%	12.1 ± 2.9%	13.7 ± 3.0%	11.6 ± 3.8%	12.8 ± 2.0%	12.1 ± 2.4%	8.9 ± 4.5%	13.9 ± 6.9%	18.1 ± 9.3%	10.4 ± 3.9%
45–54	11.5 ± 0.9%	12.8 ± 3.2%	13.1 ± 2.8%	13.5 ± 3.8%	14.3 ± 2.1%	14.8 ± 2.5%	16.8 ± 7.0%	12.7 ± 5.6%	12.9 ± 9.6%	12.3 ± 4.0%
55–64	12.7 ± 1.1%	16.3 ± 3.8%	12.8 ± 3.3%	20.5 ± 5.6%	15.7 ± 2.5%	16.1 ± 3.1%	14.6 ± 6.2%	14.2 ± 6.5%	11.7 ± 9.6%	15.0 ± 5.2%
65–69	5.4 ± 0.6%	5.6 ± 1.6%	5.0 ± 1.5%	6.7 ± 2.4%	5.2 ± 1.1%	6.0 ± 1.4%	6.7 ± 3.1%	7.0 ± 4.1%	3.7 ± 4.2%	6.2 ± 2.9%
≥70	10.2 ± 1.1%	11.0 ± 3.1%	9.8 ± 3.3%	12.9 ± 5.5%	11.6 ± 2.5%	13.4 ± 3.1%	14.0 ± 6.6%	13.0 ± 7.9%	5.2 ± 8.1%	16.1 ± 6.3%
**Race/Ethnicity**										
White	68.6 ± 1.6%	92.8 ± 5.9%	84.5 ± 5.4%	95.7 ± 8.0%	91.1 ± 4.0%	86.0 ± 4.3%	96.3 ± 11.6%	67.1 ± 9.8%	75.9 ± 15.5%	96.4 ± 8.4%
Black or African American	17.5 ± 1.0%	1.4 ± 0.9%	3.8 ± 1.3%	0.3 ± 0.4%	1.7 ± 0.5%	3.9 ± 1.5%	0.4 ± 0.7%	17.0 ± 5.5%	14.6 ± 6.6%	0.2 ± 0.4%
American Indian and Alaska Native alone	0.6 ± 0.2%	0.3 ± 0.3%	0.1 ± 0.2%	0.0 ± 0.3%	0.1 ± 0.2%	0.2 ± 0.2%	1.0 ± 0.8%	0.0 ± 0.6%	0.2 ± 0.6%	0.6 ± 0.5%
Asian	5.2 ± 0.5%	1.0 ± 0.6%	3.1 ± 1.2%	1.4 ± 1.1%	2.1 ± 0.6%	5.6 ± 1.5%	0.7 ± 0.6%	9.6 ± 3.2%	0.0 ± 0.7%	0.2 ± 0.4%
Native Hawaiian and other Pacific Islander alone	0.0 ± 0.1%	0.2 ± 0.4%	0.0 ± 0.2%	0.0 ± 0.3%	0.0 ± 0.2%	0.0 ± 0.2%	0.0 ± 0.4%	0.0 ± 0.6%	0.0 ± 0.8%	0.0 ± 0.4%
Some other race alone	1.8 ± 0.3%	0.5 ± 0.4%	0.7 ± 0.5%	0.4 ± 0.7%	0.3 ± 0.3%	0.4 ± 0.3%	0.7 ± 0.7%	2.7 ± 1.9%	5.8 ± 5.2%	0.1 ± 0.4%
Non-Hispanic/Latino	93.2 ± 1.9%	96.2 ± 6.0%	93.5 ± 5.8%	98.7 ± 8.1%	96.9 ± 4.1%	98.1 ± 4.5%	98.8 ± 11.7%	95.1 ± 10.9%	87.7 ± 16.1%	99.2 ± 8.5%
Hispanic/Latino	6.8 ± 0.6%	3.8 ± 1.5%	6.5 ± 2.9%	1.3 ± 1.1%	3.1 ± 0.9%	1.9 ± 0.6%	1.2 ± 0.9%	4.9 ± 2.8%	12.3 ± 10.4%	0.8 ± 0.6%
**Marital Status**										
Never married	44.2 ± 1.7%	28.2 ± 4.3%	35.0 ± 5.2%	23.1 ± 4.6%	28.3 ± 2.8%	25.9 ± 3.4%	28.7 ± 6.4%	39.6 ± 8.8%	52.6 ± 20.2%	32.7 ± 6.8%
Now married	38.5 ± 1.2%	55.2 ± 4.2%	50.0 ± 3.8%	61.5 ± 6.1%	55.3 ± 2.9%	56.3 ± 3.3%	55.3 ± 8.6%	43.5 ± 8.3%	34.1 ± 10.9%	46.9 ± 6.0%
Widowed	5.9 ± 0.5%	5.4 ± 1.3%	4.2 ± 1.3%	5.3 ± 1.6%	5.0 ± 0.9%	6.6 ± 1.3%	6.2 ± 2.8%	6.1 ± 2.8%	2.5 ± 3.1%	8.2 ± 2.6%
Divorced	11.4 ± 0.8%	11.3 ± 2.3%	10.8 ± 2.3%	10.1 ± 2.9%	11.4 ± 1.6%	11.1 ± 1.9%	9.8 ± 3.8%	10.8 ± 4.5%	10.8 ± 6.7%	12.3 ± 3.8%

Table S2. Characteristics of WWTPs and sewershed populations (continued)										
Sewershed	A	B	C	D	E	F	G	H	I	J
**Education Attainment**										
No schooling completed	1.9 ± 0.4%	0.3 ± 0.6%	1.2 ± 0.9%	1.0 ± 1.0%	0.9 ± 0.6%	0.9 ± 0.7%	0.6 ± 1.2%	0.9 ± 1.6%	1.3 ± 3.2%	1.4 ± 1.4%
Elementary school	0.6 ± 0.7%	0.1 ± 1.7%	0.3 ± 2.0%	0.0 ± 2.2%	0.2 ± 1.3%	0.1 ± 1.5%	0.3 ± 3.2%	0.4 ± 4.7%	0.0 ± 7.2%	0.0 ± 3.4%
Middle/Some high school	9.5 ± 1.4%	4.6 ± 2.9%	5.2 ± 3.4%	4.7 ± 3.7%	5.6 ± 2.3%	4.6 ± 2.7%	5.9 ± 5.9%	12.2 ± 9.2%	21.8 ± 18.9%	8.0 ± 5.6%
High school	27.7 ± 1.3%	19.3 ± 2.9%	25.3 ± 3.7%	31.9 ± 5.2%	27.6 ± 2.5%	15.2 ± 2.4%	31.6 ± 7.8%	37.3 ± 9.1%	30.3 ± 13.4%	42.6 ± 7.2%
Some college	17.1 ± 1.0%	17.7 ± 3.1%	17.2 ± 3.3%	16.5 ± 3.7%	17.0 ± 2.0%	13.3 ± 2.4%	18.6 ± 5.5%	14.8 ± 5.0%	23.4 ± 12.1%	17.6 ± 4.5%
Associate’s	11.8 ± 0.6%	14.9 ± 2.2%	11.9 ± 1.9%	14.9 ± 3.4%	14.7 ± 1.7%	7.9 ± 1.1%	16.7 ± 4.2%	8.8 ± 3.4%	5.5 ± 3.5%	8.4 ± 2.1%
Bachelor’s	18.1 ± 0.8%	23.8 ± 2.8%	22.8 ± 2.4%	17.7 ± 2.7%	21.0 ± 1.7%	26.0 ± 2.1%	14.2 ± 3.3%	15.5 ± 4.1%	8.9 ± 4.6%	11.1 ± 2.4%
Master’s	9.7 ± 0.6%	13.8 ± 1.9%	13.4 ± 1.9%	10.9 ± 2.1%	10.7 ± 1.2%	19.5 ± 1.9%	8.6 ± 2.6%	6.9 ± 2.8%	8.0 ± 4.6%	8.6 ± 2.8%
Professional	2.1 ± 0.3%	3.4 ± 1.0%	1.5 ± 0.6%	1.9 ± 0.8%	1.6 ± 0.5%	8.0 ± 1.1%	1.6 ± 1.2%	2.6 ± 1.6%	0.8 ± 1.2%	1.8 ± 1.2%
Doctorate	1.4 ± 0.2%	1.9 ± 0.8%	1.1 ± 0.7%	0.3 ± 0.4%	0.7 ± 0.3%	4.5 ± 0.8%	1.9 ± 1.2%	0.7 ± 1.1%	0.0 ± 1.2%	0.6 ± 0.7%
**Household Income**										
Below/Near poverty	19.5 ± 1.6%	12.2 ± 3.9%	9.1 ± 3.7%	7.3 ± 3.8%	7.4 ± 2.0%	7.5 ± 2.8%	7.4 ± 5.0%	27.9 ± 12.0%	13.7 ± 18.3%	20.0 ± 7.6%
Low income	23.3 ± 2.2%	15.4 ± 5.5%	14.9 ± 5.8%	15.6 ± 7.3%	14.3 ± 3.6%	12.1 ± 4.4%	21.1 ± 10.5%	21.6 ± 12.4%	21.7 ± 27.6%	20.6 ± 8.6%
Middle class	47.1 ± 3.3%	49.5 ± 9.9%	59.2 ± 11.3%	59.8 ± 14.4%	62.2 ± 8.2%	47.2 ± 8.0%	56.5 ± 19.9%	41.2 ± 18.6%	49.3 ± 43.2%	54.3 ± 16.1%
High income	10.1 ± 0.9%	22.9 ± 4.2%	16.8 ± 3.8%	17.2 ± 4.6%	16.1 ± 2.4%	33.2 ± 4.1%	15.0 ± 7.8%	9.3 ± 5.5%	15.3 ± 12.2%	5.1 ± 3.0%
**Employment Status**										
In labor force	59.7 ± 1.5%	67.1 ± 4.8%	67.3 ± 4.7%	61.2 ± 5.4%	66.9 ± 3.2%	64.9 ± 3.7%	62.8 ± 9.2%	60.2 ± 8.5%	42.8 ± 10.9%	57.0 ± 6.2%
Civilian labor force	59.6 ± 1.5%	66.6 ± 4.8%	66.9 ± 4.7%	61.1 ± 5.4%	66.6 ± 3.2%	64.8 ± 3.7%	62.6 ± 9.2%	60.2 ± 8.5%	42.8 ± 10.9%	57.0 ± 6.2%
Employed	55.6 ± 1.4%	63.8 ± 4.6%	64.2 ± 4.6%	58.5 ± 5.2%	63.2 ± 3.0%	61.9 ± 3.4%	58.7 ± 8.3%	55.9 ± 8.1%	42.1 ± 10.9%	53.3 ± 5.9%
Unemployed	4.0 ± 0.4%	2.8 ± 0.9%	2.7 ± 0.8%	2.6 ± 1.2%	3.4 ± 1.0%	2.9 ± 1.0%	3.9 ± 2.2%	4.4 ± 2.3%	0.7 ± 1.4%	3.7 ± 1.7%
Armed forces	0.1 ± 0.1%	0.5 ± 0.5%	0.3 ± 0.4%	0.2 ± 0.4%	0.3 ± 0.2%	0.1 ± 0.2%	0.2 ± 0.5%	0.0 ± 0.7%	0.0 ± 1.0%	0.0 ± 0.5%
Not in labor force	40.3 ± 1.2%	32.9 ± 3.0%	32.7 ± 3.3%	38.8 ± 5.7%	33.1 ± 2.2%	35.1 ± 2.8%	37.2 ± 5.2%	39.8 ± 5.9%	57.2 ± 18.0%	43.0 ± 5.0%
New York State Opioid Data Dashboard (ZIP Code Level)[c]										
OASAS program admissions[d]	2776.9	348.2	348.2	546.9	190.6	591.2	995.6	755.0	316.6	516.4
Opioid burden[e]	862.0	176.7	176.7	229.6	141.6	272.6	110.1	491.3	80.4	136.8
Naloxone administrations[f]	15.1	3.1	3.1	4.0	3.6	5.5	3.9	5.7	3.4	3.8
New York State Expanded Behavioral Risk Factor Surveillance System (County Level)[g]										
2+ Adverse childhood experiences	38.7 ± 8.5	38.7 ± 8.5	38.7 ± 8.5	38.7 ± 8.5	38.7 ± 8.5	38.7 ± 8.5	42.7 ± 13.7	24.6 ± 8.6	29.5 ± 10.0	39.7 ± 12.6
Active transportation	53.2 ± 6.1	53.2 ± 6.1	53.2 ± 6.1	53.2 ± 6.1	53.2 ± 6.1	53.2 ± 6.1	60.9 ± 9.4	67.0 ± 7.8	65.3 ± 10.4	66.4 ± 6.6
Arthritis	27.5 ± 3.5	27.5 ± 3.5	27.5 ± 3.5	27.5 ± 3.5	27.5 ± 3.5	27.5 ± 3.5	33.4 ± 6.7	26.9 ± 5.1	24.6 ± 5.3	27.3 ± 4.7
Asthma	11.7 ± 3.1	11.7 ± 3.1	11.7 ± 3.1	11.7 ± 3.1	11.7 ± 3.1	11.7 ± 3.1	13.1 ± 6.4	6.4 ± 2.3	17.5 ± 6.9	12.5 ± 4.2
Asthma ever	15.6 ± 3.4	15.6 ± 3.4	15.6 ± 3.4	15.6 ± 3.4	15.6 ± 3.4	15.6 ± 3.4	16.7 ± 6.3	12.6 ± 4.6	25.1 ± 8.1	17.5 ± 5.1
Binge drinking	13.7 ± 3.5	13.7 ± 3.5	13.7 ± 3.5	13.7 ± 3.5	13.7 ± 3.5	13.7 ± 3.5	21.5 ± 6.5	21.2 ± 5.4	17.2 ± 5.9	20.7 ± 5.7
Breast cancer screening	78.0 ± 7.1	78.0 ± 7.1	78.0 ± 7.1	78.0 ± 7.1	78.0 ± 7.1	78.0 ± 7.1	79.5 ± 8.1	81.5 ± 7.8	66.1 ± 12.1	79.4 ± 9.3

**Table S2.** Characteristics of WWTPs and sewershed populations (continued)										
Sewershed	A	B	C	D	E	F	G	H	I	J
Cardiovascular disease	7.1 ± 2.0	7.1 ± 2.0	7.1 ± 2.0	7.1 ± 2.0	7.1 ± 2.0	7.1 ± 2.0	7.3 ± 3.1	4.8 ± 2.2	5.3 ± 1.7	7.3 ± 2.2
Cardiovascular disease disability	18.5 ± 7.0	18.5 ± 7.0	18.5 ± 7.0	18.5 ± 7.0	18.5 ± 7.0	18.5 ± 7.0	14.3 ± 7.3	7.9 ± 4.4	10.5 ± 4.5	15.5 ± 7.0
Cholesterol checked	89.5 ± 3.6	89.5 ± 3.6	89.5 ± 3.6	89.5 ± 3.6	89.5 ± 3.6	89.5 ± 3.6	87.4 ± 6.9	94.5 ± 2.9	89.8 ± 5.4	86.2 ± 5.3
Chronic disease self-management	8.1 ± 3.4	8.1 ± 3.4	8.1 ± 3.4	8.1 ± 3.4	8.1 ± 3.4	8.1 ± 3.4	3.9 ± 2.8	4.9 ± 3.9	9.3 ± 6.2	9.5 ± 6.5
COPD	6.2 ± 2.0	6.2 ± 2.0	6.2 ± 2.0	6.2 ± 2.0	6.2 ± 2.0	6.2 ± 2.0	7.1 ± 2.5	4.5 ± 1.7	8.1 ± 4.7	10.8 ± 3.5
Current cannabis use	23.6 ± 9.0	23.6 ± 9.0	23.6 ± 9.0	23.6 ± 9.0	23.6 ± 9.0	23.6 ± 9.0	14.6 ± 10.6	19.6 ± 10.3	20.1 ± 10.6	11.1 ± 6.5
Current smoking	17.3 ± 3.6	17.3 ± 3.6	17.3 ± 3.6	17.3 ± 3.6	17.3 ± 3.6	17.3 ± 3.6	20.1 ± 6.7	12.1 ± 5.2	16.0 ± 6.0	21.1 ± 5.3
Current smoking disability	29.1 ± 9.0	29.1 ± 9.0	29.1 ± 9.0	29.1 ± 9.0	29.1 ± 9.0	29.1 ± 9.0	35.8 ± 13.5	25.5 ± 12.8	33.6 ± 14.2	27.1 ± 9.7
Current smoking low income	38.8 ± 11.4	38.8 ± 11.4	38.8 ± 11.4	38.8 ± 11.4	38.8 ± 11.4	38.8 ± 11.4	42.5 ± 16.6	26.8 ± 14.2	22.2 ± 10.7	41.1 ± 20.8
Current smoking poor mental health	29.7 ± 9.9	29.7 ± 9.9	29.7 ± 9.9	29.7 ± 9.9	29.7 ± 9.9	29.7 ± 9.9	36.6 ± 17.8	21.1 ± 11.5	25.0 ± 11.2	20.6 ± 8.7
Depressive disorder	22.2 ± 3.8	22.2 ± 3.8	22.2 ± 3.8	22.2 ± 3.8	22.2 ± 3.8	22.2 ± 3.8	24.6 ± 6.9	17.0 ± 5.1	22.3 ± 7.2	25.4 ± 5.9
Diabetes	9.1 ± 2.0	9.1 ± 2.0	9.1 ± 2.0	9.1 ± 2.0	9.1 ± 2.0	9.1 ± 2.0	10.2 ± 4.2	9.8 ± 3.9	11.6 ± 5.1	11.9 ± 3.1
Diabetes and prediabetes testing	46.0 ± 5.4	46.0 ± 5.4	46.0 ± 5.4	46.0 ± 5.4	46.0 ± 5.4	46.0 ± 5.4	49.4 ± 8.9	37.7 ± 8.2	46.7 ± 11.1	47.4 ± 8.2
Disability	24.2 ± 4.0	24.2 ± 4.0	24.2 ± 4.0	24.2 ± 4.0	24.2 ± 4.0	24.2 ± 4.0	30.2 ± 7.3	21.8 ± 6.5	25.3 ± 7.3	31.4 ± 6.2
e-Cigarettes	7.0 ± 2.5	7.0 ± 2.5	7.0 ± 2.5	7.0 ± 2.5	7.0 ± 2.5	7.0 ± 2.5	10.4 ± 6.0	3.2 ± 2.4	4.5 ± 3.5	5.3 ± 3.5
Flu-shot	55.4 ± 4.8	55.4 ± 4.8	55.4 ± 4.8	55.4 ± 4.8	55.4 ± 4.8	55.4 ± 4.8	52.7 ± 8.1	43.2 ± 7.9	41.0 ± 7.8	38.3 ± 5.7
Flu-shot 65+	74.5 ± 6.7	74.5 ± 6.7	74.5 ± 6.7	74.5 ± 6.7	74.5 ± 6.7	74.5 ± 6.7	70.6 ± 7.9	79.9 ± 7.1	71.3 ± 8.7	63.6 ± 8.9
Food security	76.1 ± 6.1	76.1 ± 6.1	76.1 ± 6.1	76.1 ± 6.1	76.1 ± 6.1	76.1 ± 6.1	79.2 ± 9.1	86.1 ± 7.2	76.0 ± 9.2	86.4 ± 6.0
HCV ever tested	24.0 ± 9.6	24.0 ± 9.6	24.0 ± 9.6	24.0 ± 9.6	24.0 ± 9.6	24.0 ± 9.6	21.5 ± 11.3	25.9 ± 8.6	33.7 ± 14.1	28.6 ± 11.2
Health care coverage	97.1 ± 2.2	97.1 ± 2.2	97.1 ± 2.2	97.1 ± 2.2	97.1 ± 2.2	97.1 ± 2.2	91.8 ± 6.5	95.0 ± 3.9	94.6 ± 4.3	97.3 ± 2.6
Health care coverage female 18-64	98.8 ± 1.3	98.8 ± 1.3	98.8 ± 1.3	98.8 ± 1.3	98.8 ± 1.3	98.8 ± 1.3	97.4 ± 2.9	99.7 ± 0.5	96.4 ± 4.3	96.7 ± 4.2
Health care provider	89.7 ± 2.7	89.7 ± 2.7	89.7 ± 2.7	89.7 ± 2.7	89.7 ± 2.7	89.7 ± 2.7	86.7 ± 5.7	82.9 ± 6.6	86.3 ± 5.9	88.4 ± 4.4
High blood pressure	31.0 ± 3.6	31.0 ± 3.6	31.0 ± 3.6	31.0 ± 3.6	31.0 ± 3.6	31.0 ± 3.6	34.7 ± 7.2	25.1 ± 6.5	27.7 ± 4.9	34.0 ± 5.8
High blood pressure taking medication	80.2 ± 5.6	80.2 ± 5.6	80.2 ± 5.6	80.2 ± 5.6	80.2 ± 5.6	80.2 ± 5.6	81.3 ± 10.7	83.1 ± 9.8	76.6 ± 7.9	80.9 ± 10.9
Leisure-time physical activity	78.5 ± 3.7	78.5 ± 3.7	78.5 ± 3.7	78.5 ± 3.7	78.5 ± 3.7	78.5 ± 3.7	80.4 ± 6.0	85.9 ± 4.0	83.1 ± 4.8	74.8 ± 5.6
No fruits or vegetables	34.9 ± 6.0	34.9 ± 6.0	34.9 ± 6.0	34.9 ± 6.0	34.9 ± 6.0	34.9 ± 6.0	27.7 ± 8.0	31.4 ± 7.6	33.3 ± 9.4	35.7 ± 8.3
No medical care due to cost	5.8 ± 2.0	5.8 ± 2.0	5.8 ± 2.0	5.8 ± 2.0	5.8 ± 2.0	5.8 ± 2.0	8.1 ± 4.3	5.8 ± 3.4	9.5 ± 5.9	4.2 ± 2.6
Obesity	35.2 ± 4.8	35.2 ± 4.8	35.2 ± 4.8	35.2 ± 4.8	35.2 ± 4.8	35.2 ± 4.8	34.9 ± 7.5	26.9 ± 8.8	30.8 ± 7.9	44.6 ± 7.0
Obesity disability	46.3 ± 10.3	46.3 ± 10.3	46.3 ± 10.3	46.3 ± 10.3	46.3 ± 10.3	46.3 ± 10.3	56.1 ± 13.8	37.5 ± 11.6	54.2 ± 14.7	59.2 ± 12.7
Obesity low income	42.3 ± 12.6	42.3 ± 12.6	42.3 ± 12.6	42.3 ± 12.6	42.3 ± 12.6	42.3 ± 12.6	37.2 ± 14.0	29.1 ± 15.3	40.9 ± 12.9	43.1 ± 19.9
Overweight or obese	72.9 ± 4.3	72.9 ± 4.3	72.9 ± 4.3	72.9 ± 4.3	72.9 ± 4.3	72.9 ± 4.3	66.8 ± 7.2	59.3 ± 9.3	67.9 ± 7.9	76.6 ± 6.1
Pneumonia shot 65+	75.9 ± 6.6	75.9 ± 6.6	75.9 ± 6.6	75.9 ± 6.6	75.9 ± 6.6	75.9 ± 6.6	79.3 ± 6.9	77.0 ± 7.7	70.9 ± 8.9	73.7 ± 7.8

**Table S2.** Characteristics of WWTPs and sewershed populations (continued)										
Sewershed	A	B	C	D	E	F	G	H	I	J
Poor general health	3.1 ± 1.2	3.1 ± 1.2	3.1 ± 1.2	3.1 ± 1.2	3.1 ± 1.2	3.1 ± 1.2	3.5 ± 1.8	1.6 ± 1.2	3.1 ± 1.9	3.5 ± 1.8
Poor mental health	13.9 ± 3.1	13.9 ± 3.1	13.9 ± 3.1	13.9 ± 3.1	13.9 ± 3.1	13.9 ± 3.1	21.6 ± 7.3	13.5 ± 6.7	10.8 ± 5.1	18.7 ± 5.3
Poor physical health	10.2 ± 2.3	10.2 ± 2.3	10.2 ± 2.3	10.2 ± 2.3	10.2 ± 2.3	10.2 ± 2.3	11.0 ± 3.3	6.6 ± 2.8	8.6 ± 3.4	13.0 ± 4.8
Prediabetes	5.6 ± 2.4	5.6 ± 2.4	5.6 ± 2.4	5.6 ± 2.4	5.6 ± 2.4	5.6 ± 2.4	10.9 ± 4.5	6.0 ± 3.5	9.9 ± 5.2	8.4 ± 4.5
Provider discussed healthy pregnancy	43.5 ± 15.0	43.5 ± 15.0	43.5 ± 15.0	43.5 ± 15.0	43.5 ± 15.0	43.5 ± 15.0	0.0 ± 0.0	0.0 ± 0.0	0.0 ± 0.0	0.0 ± 0.0
Recent checkup	77.6 ± 4.5	77.6 ± 4.5	77.6 ± 4.5	77.6 ± 4.5	77.6 ± 4.5	77.6 ± 4.5	75.8 ± 8.9	74.5 ± 9.7	71.8 ± 7.7	79.2 ± 7.1
Recent checkup female 18-44	78.0 ± 8.3	78.0 ± 8.3	78.0 ± 8.3	78.0 ± 8.3	78.0 ± 8.3	78.0 ± 8.3	0.0 ± 0.0	57.9 ± 24.8	0.0 ± 0.0	0.0 ± 0.0
Sugar-sweetened beverages	19.7 ± 5.1	19.7 ± 5.1	19.7 ± 5.1	19.7 ± 5.1	19.7 ± 5.1	19.7 ± 5.1	23.7 ± 8.7	13.3 ± 5.2	20.0 ± 8.5	28.5 ± 7.6

[a] Calculated using the linear distance method as described by Kapo *et al.*[1] by dividing the average distances between the population density polygon centroids and the coordinate of a WWTP by an average sewage flow velocity of 0.6 m/s; [b] Calculated based on data extracted from the 2017-2021 American Community Survey at the census block group level as estimates ± margins of error.[2] [c] Extracted from the New York State Opioid Data Dashboard at the ZIP code level as estimates.[3] [d] Admissions to OASAS-certified substance use disorder treatment programs for any opioid (incl. heroin), crude rate per 100,000 population - Aged 12+ years. [e] Opioid burden (including outpatient ED visits and hospital discharges for non-fatal opioid overdose, abuse, dependence, and unspecified use; and opioid overdose deaths), crude rate per 100,000 population. [f] Unique naloxone administrations by EMS agencies, crude rate per 1,000 unique 911 EMS dispatches. [g] Extracted from the 2021 Expanded Behavioral Risk Factor Surveillance System (BRFSS) at the county level as estimates ± margins of error.[4]

S3. Screening and quantification of substances by online SPE-LC-HRMS

Table S3. Online SPE-LC-HRMS instrument settings

LC TriPlus RSH Autosampler and Liquid Handling System		
Sample Volume (µL)	1,000	
Bottom Sense Sample Vial	False	
Pre-Clean with Solvent 1	0	
Pre-Clean with Solvent 2	1	
Pre-Clean with Sample	0	
Sample Aspirate Flow Rate (µL/s)	100	
Filling Strokes	0	
Pullup Delay (ms)	1,000	
Transfer Time (min)	1.1	
Elution Time (min)	30.0	
Loop Bypass Enable	False	
Loop Bypass Start (min)	0.0	
Loop Bypass End (min)	0.0	
Inject Sample Flow Rate (µL/s)	500	
Air Volume (µL)	0	
Post-Clean with Solvent 1	3	
Post-Clean with Solvent 2	1	
Clean Valve with Solvent 1 (µL)	1,500	
Clean Valve with Solvent 2 (µL)	1,500	
Sample Vial Depth (mm)	44.0	
Height from Bottom of Sample Vial (mm)	0.5	
Clean Valve Flow Rate (µL/s)	80	
Wash Volume (%)	15	
Look Ahead	False	
Get Ready Delay (s)	5.0	
EQuan Tool	LS4	
Wait For Sample Temperature	False	
Sample Temperature (°C)	4.0	
Standby Temperature (°C)	4.0	
Look Ahead Delay (min)	0.0	
**Vanquish Horizon UHPLC System**		
Flow Rate: 0.2 mL/min<br>Time (min)	Mobile Phase A (H_2O + 0.1% FA)	Mobile Phase B (MeOH + 0.1% FA)
0.0	90%	10%
0.5	90%	10%
1.0	90%	10%
8.0	50%	50%
21.0	5%	95%
29.5	5%	95%
30.0	90%	10%
32.0	90%	10%
**Orbitrap Exploris 240 MS System**		
Application Mode: Small Molecule<br>Method Duration (min): 32	Positive H-ESI	Negative H-ESI
	Ion Source	
Spray Voltage (V) | Static	3,500	2,500
Sheath Gas (Arb) | Static	35	35
Auxiliary Gas (Arb) | Static	7	7
Sweep Gas (Arb) | Static	0	0
Ion Transfer Tube Temperature (°C)	320	320
Vaporizer Temperature (°C)	275	275
	MS Global Settings	
Infusion Mode	Liquid Chromatography	Liquid Chromatography

**Table S3.** Online SPE-LC-HRMS instrument settings (continued)		
Orbitrap Exploris 240 MS System		
Expected LC Peak Width (s)	3	3
Mild Trapping	False	False
Default Charge State	1	1
Enable Xcalibur AcquireX Method Modifications	False	False
Internal Mass Calibration	EASY-IC™	EASY-IC™
Mode	Run Start	Run Start
	Divert Valve A	
Time (min)	Position	Position
0	1 – 6	1 – 6
0.5	1 – 2	1 – 2
30	1 – 6	1 – 6
	Full Scan	
Start Time (min)	0.5	0.5
End Time (min)	30.0	30.0
Orbitrap Resolution FWHM at m/z 200	120,000	120,000
Scan Range (m/z)	100 – 1000	100 – 1000
RF Lens (%)	70	70
AGC Target	Standard	Standard
Maximum Injection Time Mode	Auto	Auto
Microscans	1	1
Data Type	Profile	Profile
Polarity	Positive	Negative
Source Fragmentation	Disabled	Disabled
	Filters	
Intensity Threshold	100,000	100,000
Include Charge State(s)	1 – 2	1 – 2
Include Undetermined Charge States	False	False
	Targeted Mass	
Mass List Type	m/z	m/z
Time Mode	Start/End Time	Start/End Time
Include Intensity Threshold	False	False
Mass Tolerance (ppm)	5	5
Set Collision Energy per Compound	False	False
Perform Dependent Scan on Most Intense Ion if No Targets are Found	True	True
	Targeted Mass Exclusion	
Mass List Type	m/z	m/z
Time Mode	Start/End Time	Start/End Time
Include Intensity Threshold	False	False
Exclusion Mass Width (ppm)	5	5
	Data Dependent ddMS^2 Scan	
Data Dependent Mode	Number of Scans	Number of Scans
Number of Dependent Scans	5	5
Multiplex Ions	False	False
Isolation Window (m/z)	1	1
Isolation Offset	Off	Off
Collision Energy Type	Normalized	Normalized
HCD Collision Energies (%)	15, 30, 45, 60, 75	15, 30, 45, 60, 75
Orbitrap Resolution FWHM at m/z 200	15,000	15,000
Scan Range Mode	Auto	Auto
AGC Target	Standard	Standard
Maximum Injection Time Mode	Auto	Auto
Microscans	1	1
Data Type	Centroid	Centroid

**Table S4.** Online SPE-LC-HRMS method parameters for target substances						
Compound Name	Molecular Formula	Adduct	*m/z*	Diagnostic Fragment Ion	RT (min)	ILIS
Fentanyl	$C_{22}H_{28}N_2O$	[M+H]+	337.2274	188.1433	10.55	Fentanyl-d_5
Norfentanyl	$C_{14}H_{20}N_2O$	[M+H]+	233.1647	84.0807	8.47	Norfentanyl-d_5
Meperidine	$C_{15}H_{21}NO_2$	[M+H]+	248.1644	220.1332	9.48	Meperidine-d_4
Normeperidine	$C_{14}H_{19}NO_2$	[M+H]+	234.1487	160.1119	9.74	Normeperidine-d_4
Codeine	$C_{18}H_{21}NO_3$	[M+H]+	300.1590	282.1487	5.50	Codeine-d_3
Norcodeine	$C_{17}H_{19}NO_3$	[M+H]+	286.1435	268.1329	5.68	Norcodeine-d_3
Hydrocodone	$C_{18}H_{21}NO_3$	[M+H]+	300.1592	199.0752	6.16	Hydrocodone-d_3
Norhydrocodone	$C_{17}H_{19}NO_3$	[M+H]+	286.1438	241.0859	6.36	Norhydrocodone-d_3
Morphine	$C_{17}H_{19}NO_3$	[M+H]+	286.1438	185.0598	3.26	Morphine-d_3
Hydromorphone	$C_{17}H_{19}NO_3$	[M+H]+	286.1436	199.0754	3.97	Hydromorphone-d_3
Normorphine	$C_{16}H_{17}NO_3$	[M+H]+	272.1283	199.0752	3.32	Normorphine-d_3
Morphine-3-glucuronide (M3G)	$C_{23}H_{27}NO_9$	[M+H]+	462.1759	286.1437	2.22	Morphine-d_3-3-glucuronide
Heroin	$C_{21}H_{23}NO_5$	[M+H]+	370.1647	268.1332	8.64	Heroin-d_9
6-Monoacetylmorphine (6-MAM)	$C_{19}H_{21}NO_4$	[M+H]+	328.1538	211.0751	6.36	6-Monoacetylmorphine-d_6
Oxycodone	$C_{18}H_{21}NO_4$	[M+H]+	316.1542	298.1436	5.89	Oxycodone-d_3
Noroxycodone	$C_{17}H_{19}NO_4$	[M+H]+	302.1368	284.1281	6.11	Noroxycodone-d_3
Oxymorphone	$C_{17}H_{19}NO_4$	[M+H]+	302.1390	284.1284	3.60	Oxymorphone-d_3
Noroxymorphone	$C_{16}H_{17}NO_4$	[M+H]+	288.1230	270.1122	3.69	Normorphine-d_3
Dihydrocodeine	$C_{18}H_{23}NO_3$	[M+H]+	302.1746	245.1170	5.37	Codeine-d_3
Dihydromorphine	$C_{17}H_{21}NO_3$	[M+H]+	288.1580	270.1498	3.14	(±)-Cotinine-d_3
Desomorphine	$C_{17}H_{21}NO2$	[M+H]+	272.1646	167.0855	6.41	6-Monoacetylmorphine-d_6
Buprenorphine	$C_{29}H_{41}NO_4$	[M+H]+	468.3104	414.2649	11.34	Buprenorphine-d_4
Norbuprenorphine	$C_{25}H_{35}NO_4$	[M+H]+	414.2636	396.2513	10.35	Norbuprenorphine-d_3
(±)-Methadone	$C_{21}H_{27}NO$	[M+H]+	310.2161	265.1586	13.35	(±)-Methadone-d_3
2-Ethylidene-1,5-dimethyl-3,3-diphenylpyrrolidine (EDDP)	$C_{20}H_{23}N$	[M+H]+	278.1903	234.1277	11.24	EDDP-d_3
(+)-Tramadol	$C_{16}H_{25}NO_2$	[M+H]+	264.1957	58.0651	8.60	(+)-Tramadol-^{13}C,d_3
(+)-*O*-Desmethyltramadol	$C_{15}H_{23}NO_2$	[M+H]+	250.1801	58.0651	6.82	(+)-Tramadol-^{13}C,d_3
(+)-*N*-Desmethyltramadol	$C_{15}H_{23}NO_2$	[M+H]+	250.1800	232.1694	9.10	(+)-Tramadol-^{13}C,d_3
Naloxone	$C_{19}H_{21}NO_4$	[M+H]+	328.1545	310.1435	5.37	Codeine-d_3
Diphenhydramine	$C_{17}H_{21}NO$	[M+H]+	256.1691	167.0854	11.41	Diphenhydramine-d_3
Alprazolam	$C_{17}H_{13}ClN_4$	[M+H]+	309.0903	281.0714	13.91	Alprazolam-d_5
α-Hydroxyalprazolam	$C_{17}H_{13}ClN_4O$	[M+H]+	325.0853	297.0666	13.28	α-Hydroxyalprazolam-d_5
Diazepam	$C_{16}H_{13}ClN_2O$	[M+H]+	285.0786	154.0416	15.29	Diazepam-d_5
Nordiazepam	$C_{15}H_{11}ClN_2O$	[M+H]+	271.0628	140.0259	14.62	Nordiazepam-d_5
Cocaine	$C_{17}H_{21}NO_4$	[M+H]+	304.1544	182.1175	8.95	Cocaine-d_3
Benzoylecgonine	$C_{16}H_{19}NO_4$	[M+H]+	290.1388	168.1019	8.20	Benzoylecgonine-d_3
Ecgonine Methyl Ester	$C_{10}H_{17}NO_3$	[M+H]+	200.1281	182.1175	2.08	Ecgonine Methyl Ester-d_3
(-)-Norcocaine	$C_{16}H_{19}NO_4$	[M+H]+	290.1388	168.1018	9.40	Cocaine-d_3
(±)-3,4-Methylenedioxymethamphetamine ((±)-MDMA)	$C_{11}H_{15}NO_2$	[M+H]+	194.1173	163.0752	7.00	(±)-MDMA-d_5
(±)-3,4-Methylenedioxyamphetamine ((±)-MDA)	$C_{10}H_{13}NO_2$	[M+H]+	180.1018	163.0752	6.88	(±)-MDA-d_5

**Table S4.** Online SPE-LC-HRMS method parameters for target substances (continued)						
Compound Name	Molecular Formula	Adduct	*m/z*	Diagnostic Fragment Ion	RT (min)	ILIS
(±)-Methamphetamine	$C_{10}H_{15}N$	[M+H]+	150.1276	91.0541	6.91	(±)-Methamphetamine-d_8
(±)-Amphetamine	$C_9H_{13}N$	[M+H]+	136.1119	91.0542	6.75	(±)-Amphetamine-d_{10}
(-)-Nicotine	$C_{10}H_{14}N_2$	[M+H]+	163.1228	132.0806	2.22	Nicotine-d_4
(-)-Cotinine	$C_{10}H_{12}N_2O$	[M+H]+	177.1020	80.0494	2.46	(±)-Cotinine-d_3
trans-3'-Hydroxycotinine	$C_{10}H_{12}N_2O_2$	[M+H]+	193.0972	134.0599	2.08	*trans*-3'-Hydroxycotinine-d_3
Δ^9-Tetrahydrocannabinol (THC)	$C_{21}H_{30}O_2$	[M+H]+	315.2319	193.1223	22.05	(-)-Δ^9-THC-d_3
(±)-11-nor-9-Carboxy-Δ^9-tetrahydrocannabinol (THC-COOH)	$C_{21}H_{28}O_4$	[M+H]+	345.2059	299.2005	20.37	(±)-11-nor-9-Carboxy-Δ^9-THC-d_3
(±)-11-Hydroxy-Δ^9-tetrahydrocannabinol (11-OH-THC)	$C_{21}H_{30}O_3$	[M+H]+	331.2271	313.2159	19.92	(±)-11-Hydroxy-Δ^9-THC-d_3
Caffeine	$C_8H_{10}N_4O_2$	[M+H]+	195.0875	138.0661	7.20	Caffeine-d_9
Paraxanthine	$C_7H_8N_4O_2$	[M+H]+	181.0719	124.0504	5.94	Caffeine-d_9
Sucralose	$C_{12}H_{19}Cl_3O_8$	[M+FA-H]-	441.0128	395.0078	7.69	Sucralose-d_6

**Table S5.** Online SPE-LC-HRMS method parameters for isotope-labeled internal standards				
Compound Name	Molecular Formula	Adduct	*m/z*	RT (min)
Fentanyl-d_5	$C_{22}H_{23}D_5N_2O$	[M+H]+	342.2588	10.53
Norfentanyl-d_5	$C_{14}H_{15}D_5N_2O$	[M+H]+	238.1962	8.42
Meperidine-d_4	$C_{15}H_{17}D_4NO_2$	[M+H]+	252.1896	9.46
Normeperidine-d_4	$C_{14}H_{15}D_4NO_2$	[M+H]+	238.1740	9.72
Codeine-d_3	$C_{18}H_{18}D_3NO_3$	[M+H]+	303.1783	5.37
Norcodeine-d_3	$C_{17}H_{16}D_3NO_3$	[M+H]+	289.1626	5.62
Hydrocodone-d_3	$C_{18}H_{18}D_3NO_3$	[M+H]+	303.1783	6.00
Norhydrocodone-d_3	$C_{17}H_{16}D_3NO_3$	[M+H]+	289.1626	6.33
Morphine-d_3	$C_{17}H_{16}D_3NO_3$	[M+H]+	289.1626	3.97
Hydromorphone-d_3	$C_{17}H_{16}D_3NO_3$	[M+H]+	289.1626	3.28
Normorphine-d_3	$C_{16}H_{14}D_3NO_3$	[M+H]+	275.1470	3.32
Morphine-d_3-3-glucuronide	$C_{23}H_{24}D_3NO_9$	[M+H]+	465.1947	2.25
Heroin-d_9	$C_{21}H_{14}D_9NO_5$	[M+H]+	379.2214	8.60
6-Monoacetylmorphine-d_6	$C_{19}H_{15}D_6NO_4$	[M+H]+	334.1920	6.35
Oxycodone-d_3	$C_{18}H_{18}D_3NO_4$	[M+H]+	319.1732	6.14
Noroxycodone-d_3	$C_{17}H_{16}D_3NO_4$	[M+H]+	305.1575	6.11
Oxymorphone-d_3	$C_{17}H_{16}D_3NO_4$	[M+H]+	305.1575	3.60
Buprenorphine-d_4	$C_{29}H_{37}D_4NO_4$	[M+H]+	472.3359	11.34
Norbuprenorphine-d_3	$C_{25}H_{32}D_3NO_4$	[M+H]+	417.2827	10.34
(±)-Methadone-d_3	$C_{21}H_{24}D_3NO$	[M+H]+	313.2354	13.32
EDDP-d_3	$C_{20}H_{21}D_3N^+$	[M]+	281.2092	11.23
(+)-Tramadol-^{13}C,d_3	$C_{15}{}^{13}CH_{22}D_3NO_2$	[M+H]+	268.2180	8.57
Diphenhydramine-d_3	$C_{17}H_{18}D_3NO$	[M+H]+	259.1884	11.42
Alprazolam-d_5	$C_{17}H_8D_5N_4Cl$	[M+H]+	314.1215	13.84
α-Hydroxyalprazolam-d_5	$C_{17}H_8ClD_5N_4O$	[M+H]+	330.1165	13.23
Diazepam-d_5	$C_{16}H_8ClD_5N_2O$	[M+H]+	290.1103	15.21
Nordiazepam-d_5	$C_{15}H_6ClD_5N_2O$	[M+H]+	276.0947	14.51
Cocaine-d_3	$C_{17}H_{18}D_3NO_4$	[M+H]+	307.1732	8.95
Benzoylecgonine-d_3	$C_{16}H_{16}D_3NO_4$	[M+H]+	293.1575	8.20
Ecgonine Methyl Ester-d_3	$C_{10}H_{14}D_3NO_3$	[M+H]+	203.1470	2.09
(±)-MDMA-d_5	$C_{11}H_{10}D_5NO_2$	[M+H]+	199.1489	6.96
(±)-MDA-d_5	$C_{10}H_8D_5NO_2$	[M+H]+	185.1333	6.82
(±)-Methamphetamine-d_8	$C_{10}H_7D_8N$	[M+H]+	158.1779	6.91
(±)-Amphetamine-d_{10}	$C_9D_{10}H_3N$	[M+H]+	146.1748	6.64
Nicotine-d_4	$C_{10}H_{10}D_4N_2$	[M+H]+	167.1481	2.20
(±)-Cotinine-d_3	$C_{10}H_9D_3N_2O$	[M+H]+	180.1211	2.51
trans-3'-Hydroxycotinine-d_3	$C_{10}H_9D_3N_2O_2$	[M+H]+	196.1160	2.08
(-)-Δ^9-THC-d_3	$C_{21}H_{27}D_3O_2$	[M+H]+	318.2507	22.05
(±)-11-nor-9-Carboxy-Δ^9-THC-d_3	$C_{21}H_{25}D_3O_4$	[M+H]+	348.2249	20.34
(±)-11-Hydroxy-Δ^9-THC-d_3	$C_{21}H_{27}D_3O_3$	[M+H]+	334.2456	19.89
Caffeine-d_9	$C_8HD_9N_4O_2$	[M+H]+	204.1441	7.12
Sucralose-d_6	$C_{12}H_{13}Cl_3D_6O_8$	[M+FA-H]-	447.0504	7.67

Table S6. Online SPE-LC-HRMS method performance for target substances

Compound Name	R^2	Intraday Precision	Intraday Accuracy	Interday Precision	Interday Accuracy	Recovery	$LOQ_{Wastewater}$ (ng/L)
Fentanyl	0.9996	3±2%	101±6%	6±1%	96±6%	89±4%	1.2
Norfentanyl	0.9992	5±3%	94±4%	5±1%	94±6%	95±3%	1.1
Meperidine	0.9995	5±2%	105±3%	6±3%	103±5%	84±4%	1.2
Normeperidine	0.9995	5±3%	102±4%	6±1%	99±5%	86±7%	1.2
Codeine	0.9992	3±1%	104±4%	3±0%	105±3%	93±7%	1.1
Norcodeine	0.9996	6±2%	100±7%	7±3%	100±6%	93±7%	1.1
Hydrocodone	0.9993	8±2%	104±7%	8±1%	107±7%	80±5%	1.3
Norhydrocodone	0.9989	6±3%	104±5%	9±1%	107±8%	81±7%	1.3
Morphine	0.9973	9±6%	102±11%	10±3%	105±11%	81±5%	26
Hydromorphone	0.9907	8±5%	105±3%	9±5%	101±9%	87±6%	24
Normorphine	0.9949	14±2%	107±14%	13±6%	105±12%	78±7%	27
Morphine-3-glucuronide (M3G)	0.9986	13±4%	111±9%	13±4%	113±12%	69±6%	31
Heroin	0.9996	7±3%	102±7%	8±1%	105±7%	85±5%	1.2
6-Monoacetylmorphine (6-MAM)	0.9996	8±4%	99±13%	12±1%	102±11%	81±5%	1.3
Oxycodone	0.9994	4±2%	109±3%	6±1%	104±5%	80±2%	1.3
Noroxycodone	0.9995	5±1%	103±5%	7±1%	103±6%	83±5%	1.3
Oxymorphone	0.9929	7±2%	106±6%	6±1%	106±7%	90±4%	5.8
Noroxymorphone	0.9950	10±6%	93±4%	8±2%	94±10%	89±6%	24
Dihydrocodeine	0.9994	11±3%	91±11%	7±3%	93±10%	86±7%	2.5
Dihydromorphine	0.9990	9±5%	103±4%	10±2%	99±9%	85±5%	12
Desomorphine	0.9987	4±0%	103±4%	7±1%	102±6%	87±3%	1.2
Buprenorphine	0.9993	4±2%	107±6%	5±1%	108±4%	83±5%	1.3
Norbuprenorphine	0.9986	4±3%	107±3%	7±2%	100±7%	84±8%	1.3
(±)-Methadone	0.9993	5±2%	102±6%	6±2%	101±6%	91±2%	1.1
2-Ethylidene-1,5-dimethyl-3,3-diphenylpyrrolidine (EDDP)	0.9990	7±3%	112±10%	11±4%	107±9%	89±11%	1.2
(+)-Tramadol	0.9998	5±1%	105±6%	5±1%	103±6%	83±2%	1.2
(+)-*O*-Desmethyltramadol	0.9968	5±2%	99±7%	6±1%	102±5%	93±5%	1.1
(+)-*N*-Desmethyltramadol	0.9985	5±1%	97±4%	5±1%	95±4%	87±4%	1.2
Naloxone	0.9989	6±6%	93±13%	8±2%	94±8%	87±6%	1.2
Diphenhydramine	0.9994	11±4%	109±8%	11±3%	95±15%	85±6%	1.2
Alprazolam	0.9994	5±2%	115±8%	10±3%	103±11%	86±6%	1.2
α-Hydroxyalprazolam	0.9993	6±2%	108±6%	7±1%	103±7%	81±7%	1.3
Diazepam	0.9977	5±3%	94±2%	7±3%	98±7%	87±3%	1.2
Nordiazepam	0.9978	4±1%	106±4%	5±1%	104±4%	86±4%	1.2
Cocaine	0.9996	3±1%	118±4%	3±1%	115±3%	82±2%	1.2
Benzoylecgonine	0.9992	8±2%	103±10%	10±6%	108±12%	89±4%	1.2
Ecgonine Methyl Ester	0.9987	14±5%	93±10%	12±6%	97±13%	70±6%	15
(-)-Norcocaine	0.9987	6±2%	98±7%	4±1%	97±6%	90±5%	1.2
(±)-3,4-Methylenedioxymethamphetamine ((±)-MDMA)	0.9989	6±2%	96±4%	9±1%	103±7%	83±4%	2.5
(±)-3,4-Methylenedioxyamphetamine ((±)-MDA)	0.9986	6±2%	109±5%	10±1%	101±8%	70±9%	3.1
(±)-Methamphetamine	0.9973	4±1%	110±4%	10±1%	106±8%	81±5%	1.3

Table S6. Online SPE-LC-HRMS method performance for target substances (continued)							
Compound Name	R^2	Intraday Precision	Intraday Accuracy	Interday Precision	Interday Accuracy	Recovery	$LOQ_{Wastewater}$ (ng/L)
(±)-Amphetamine	0.9959	5±2%	96±4%	10±1%	105±8%	77±7%	6.9
(-)-Nicotine	0.9975	14±12%	110±11%	17±9%	125±20%	81±2%	6.3
(-)-Cotinine	0.9950	17±10%	101±26%	11±4%	103±16%	87±2%	5.9
trans-3'-Hydroxycotinine	0.9977	8±3%	93±5%	11±3%	104±13%	73±4%	14
Δ^9-Tetrahydrocannabinol (THC)	0.9979	6±2%	110±5%	6±1%	108±7%	81±3%	13
(±)-11-nor-9-Carboxy-Δ^9-tetrahydrocannabinol (THC-COOH)	0.9972	8±5%	103±3%	12±3%	113±11%	74±7%	2.9
(±)-11-Hydroxy-Δ^9-tetrahydrocannabinol (11-OH-THC)	0.9991	8±7%	105±16%	11±3%	106±10%	74±7%	2.9
Caffeine	0.9993	11±3%	98±12%	6±3%	96±10%	82±3%	6.3
Paraxanthine	0.9981	15±2%	107±16%	7±5%	105±13%	94±3%	22
Sucralose	0.9985	8±4%	108±12%	7±2%	108±8%	71±4%	2.9

$$\text{Intraday Precision} = \left[\frac{\left(\frac{\text{Peak Area}_{\text{400 ng/L or 4000 ng/L Spiked in Pooled Wastewater Sample}}}{\text{Peak Area}_{\text{ILIS}}} - \frac{\text{Peak Area}_{\text{Non-Spiked Pooled Wastewater Sample}}}{\text{Peak Area}_{\text{ILIS}}}\right)}{\left(\frac{\text{Peak Area}_{\text{400 ng/L or 4000 ng/L Spiked in Deionized Water}}}{\text{Peak Area}_{\text{ILIS}}}\right)}\right]_{\text{Standard Deviation, Triplicate Analysis within 1 Day}} \times 100\%$$

$$\text{Intraday Accuracy} = \left[\frac{\left(\frac{\text{Peak Area}_{\text{400 ng/L or 4000 ng/L Spiked in Pooled Wastewater Sample}}}{\text{Peak Area}_{\text{ILIS}}} - \frac{\text{Peak Area}_{\text{Non-Spiked Pooled Wastewater Sample}}}{\text{Peak Area}_{\text{ILIS}}}\right)}{\left(\frac{\text{Peak Area}_{\text{400 ng/L or 4000 ng/L Spiked in Deionized Water}}}{\text{Peak Area}_{\text{ILIS}}}\right)}\right]_{\text{Mean, Triplicate Analysis within 1 Day}} \times 100\%$$

$$\text{Interday Precision} = \left[\frac{\left(\frac{\text{Peak Area}_{\text{400 ng/L or 4000 ng/L Spiked in Pooled Wastewater Sample}}}{\text{Peak Area}_{\text{ILIS}}} - \frac{\text{Peak Area}_{\text{Non-Spiked Pooled Wastewater Sample}}}{\text{Peak Area}_{\text{ILIS}}}\right)}{\left(\frac{\text{Peak Area}_{\text{400 ng/L or 4000 ng/L Spiked in Deionized Water}}}{\text{Peak Area}_{\text{ILIS}}}\right)}\right]_{\text{Standard Deviation, Triplicate Analysis over 3 Days}} \times 100\%$$

$$\text{Interday Accuracy} = \left[\frac{\left(\frac{\text{Peak Area}_{\text{400 ng/L or 4000 ng/L Spiked in Pooled Wastewater Sample}}}{\text{Peak Area}_{\text{ILIS}}} - \frac{\text{Peak Area}_{\text{Non-Spiked Pooled Wastewater Sample}}}{\text{Peak Area}_{\text{ILIS}}}\right)}{\left(\frac{\text{Peak Area}_{\text{400 ng/L or 4000 ng/L Spiked in Deionized Water}}}{\text{Peak Area}_{\text{ILIS}}}\right)}\right]_{\text{Mean, Triplicate Analysis over 3 Days}} \times 100\%$$

$$\text{Recovery}_{\text{Matching ILIS}} = \left[\frac{\text{Peak Area}_{\text{ILIS, 400 ng/L Spiked in Pooled Wastewater Sample}}}{\text{Peak Area}_{\text{ILIS, 400 ng/L Spiked in Deionized Water}}}\right]_{\text{Mean, Triplicate Analysis over 3 Days}} \times 100\%$$

$$\text{Recovery}_{\text{Non-Matching ILIS}} = \left[\frac{\text{Peak Area}_{\text{400 ng/L or 4000 ng/L Spiked in Pooled Wastewater Sample}} - \text{Peak Area}_{\text{Non-Spiked Pooled Wastewater Sample}}}{\text{Peak Area}_{\text{400 ng/L or 4000 ng/L Spiked in Deionized Water}}}\right]_{\text{Mean, Triplicate Analysis over 3 Days}} \times 100\%$$

$$\text{LOQ}_{\text{Wastewater}} = \frac{\text{LOQ}_{\text{Deionized Water}}\ \text{(defined as the lowest calibration standard containing at least five consecutive full scan data points)}}{\text{Recovery}}$$

**Table S7.** *TraceFinder 5.2 SP1* quantitation method settings	
Trace Selection	
Detector	MS
Trace	Mass range
Scan filter	FTMS + p ESI Full ms [100 - 1000]
Range type	*m/z*
Masses	Enable
Retention Times	
Detection type	Single - Detected
Expected RT	Compound-specific
Windows (sec)	30
View width (min)	2.0
Detection Algorithm (By Peak)	
Detection algorithm	ICIS
Peak detection strategy	Highest peak
Peak threshold type	Area
Threshold	1
Smoothing	9
Extraction window (min)	3.0
Area noise factor	5
Peak noise factor	10
Baseline window	40
Min peak height (S/N)	3
Noise method	Incos
Min peak width	3
Multiplet resolution	10
Area tail extension	5
Area scan window	0
mzVault (Enabled)	
Search type	Highchem
Prefilter type	Precursor
Scan filter (Global)	ms2
Precursor tolerance	10 ppm
Scan threshold	80
Passing value	80
Ignore precursor	Yes
Reverse search	Yes
Isotopes (Enabled; Global)	
Fit threshold %	90
Allowed mass deviation (ppm)	5
Allowed intensity deviation (%)	10
Suitability (Enabled; By Peak)	

**Table S8.** *Compound Discoverer 3.3 SP2* workflow node settings	
Select Spectra	
1. Spectrum Properties Filter	
Lower RT Limit	1.5
Upper RT Limit	29.5
2. Scan Event Filters	
Polarity Mode	Any
Align Retention Times	
1. General Settings	
Alignment Model	Adaptive curve
Maximum Shift [min]	2
Mass Tolerance	5 ppm
Detection Compounds	
1. General Settings	
Mass Tolerance	5 ppm
Min. Peak Intensity	100000
Use Most Intense Isotope Only	True
2. Peak Detection	
Chromatographic S/N Threshold	1.5
Remove Baseline	False
3. Isotope Pattern Detection	
Group Isotopes for	Br; Cl
4. Compound Detection	
Ions	[M+H]+1; [M+H-H2O]+1; [M+Na]+1; [M-H]-1; [M+FA-H]-1
Merge Features	
1. Peak Consolidation	
Mass Tolerance	5 ppm
RT Tolerance [min]	0.1
Group Compounds	
1. General Settings	
Mass Tolerance	5 ppm
RT Tolerance [min]	0.1
Align Peaks	False
Preferred Ions	[M+H]+1; [M-H]-1
Area Integration	Most Common Ion
2. Peak Rating Contributions	
Area Contribution	3
CV Contribution	10
FWHM to Base Contribution	5
Jaggedness Contribution	5
Modality Contribution	5
Zig-Zag Index Contribution	5
3. Peak Rating Filter	
Peak Rating Threshold	4
Number of Files	2
Fill Gaps	
1. General Settings	
Mass Tolerance	5 ppm
S/N Threshold	1.5
Apply SERRF QC Correction	
1. General Settings	
Min. QC Coverage [%]	30
Max. QC Area RSD [%]	30
Max. Corrected QC Area RSD [%]	25
# Batches	1
Interpolate Gap-filled QC Areas	True

**Table S8.** *Compound Discoverer 3.3 SP2* workflow node settings (continued)	
Mark Background Compounds	
1. General Settings	
Max. Sample/Blank	5
Max. Blank/Sample	0
Hide Background	True
Predict Compositions	
1. Prediction Settings	
Mass Tolerance	5 ppm
Min. Element Counts	C2 H4 N
Max. Element Counts	C40 H80 Br Cl3 F3 I N6 O10 S
Min. RDBE	0
Max. RDBE	40
Min. H/C	0.1
Max. H/C	3.5
Max. # Candidates	10
2. Pattern Matching	
Intensity Tolerance [%]	30
Intensity Threshold [%]	0.1
S/N Threshold	3
Use Dynamic Recalibration	True
3. Fragments Matching	
Use Fragments Matching	True
Mass Tolerance	5 ppm
S/N Threshold	3
Search mzVault	
1. Search Settings	
mzVault Library	\MassBank.db\CDC_FAS_EDP.db\HighResNPS.db
Compound Classes	All
Match Ion Activation Type	False
Match Ion Activation Energy	Any
Ion Activation Energy Tolerance	50
Match Ionization Method	False
Apply Intensity Threshold	False
Precursor Mass Tolerance	5 ppm
Match Analyzer Type	False
Search Algorithm	HighChem HighRes
Match Factor Threshold	30
RT Tolerance [min]	2
Use Retention Time	False
Search mzCloud	
1. General Settings	
Compound Classes	All
Library	Autoprocessed; Reference
Search MSn Tree	False
2. DDA Search	
Identity Search	HighChem HighRes
Match Ion Activation Type	False
Match Ion Activation Energy	Any
Ion Activation Energy Tolerance	50
Apply Intensity Threshold	False
Similarity Search	None
Match Factor Threshold	30
3. DIA Search	
Use DIA Scans for Search	False
Max. Isolation Width [Da]	500

**Table S8.** *Compound Discoverer 3.3 SP2* workflow node settings (continued)	
Search mzCloud (continued)	
Match Ion Activation Type	False
Match Ion Activation Energy	Any
Ion Activation Energy Tolerance	100
Apply Intensity Threshold	False
Match Factor Threshold	20
Assign Compound Annotations	
1. General Settings	
Mass Tolerance	5 ppm
2. Data Sources	
Data Source #1	mzCloud Search
Data Source #2	mzVault Search
Data Source #3	MassList Search
Data Source #4	ChemSpider Search
Data Source #5	Predicted Compositions
3. Scoring Rules	
Use mzLogic	True
Use Spectral Distance	True
SFit Threshold	90
SFit Range	20
4. Reprocessing	
Clear Names	False
Search Mass Lists	
1. Search Settings	
Mass Lists	\Import from csv\CDC_HighResNPS.massList (**Table S9**)
Use Retention Time	True
RT Tolerance [min]	3
Mass Tolerance	5 ppm
Search ChemSpider	
1. Search Settings	
Database(s)	Cayman Chemical; DrugBank; EPA DSSTox
Search Mode	By Formula Only
Mass Tolerance	5 ppm
Max. # of Results per Compound	10
Max. # of Predicted Compositions to be Searched per Compound	3
Calculate Mass Defect	
1. Mass Defect	
Fractional Mass	True
Standard Mass Defect	True
Relative Mass Defect	True
Kendrick Mass Defect	True
2. Kendrick Formula	
Formula 1	C2 F4
Formula 2	C2 F3 O
Formula 3	C2 H4
Formula 4	C3 H6
Formula 5	C8 H8
Apply Spectral Distance	
1. Pattern Matching	
Mass Tolerance	5 ppm
Intensity Tolerance [%]	30
Intensity Threshold [%]	0.1
S/N Threshold	3
Use Dynamic Recalibration	True

Table S8. *Compound Discoverer 3.3 SP2* workflow node settings (continued)	
Search Neutral Losses	
1. General Settings	
Neutral Losses	$C_{10}H_{10}O$, $C_{10}H_{11}NO$, $C_{11}H_{12}O$, $C_{13}H_{17}NO$, $C_{14}H_{17}NO$, $C_{14}H_{17}NO_2$, $C_{14}H_{20}N_2O$, $C_{16}H_{24}N_2O$, $C_2H_4O_3$, C_2H_5NO, C_2H_6O, C_2H_7N, C_2H_7NO, C_2H_7O, C_3H_7NO, C_3H_8O, C_3H_9N, C_3H_9NO, C_3H_9O, $C_4H_{10}O$, $C_4H_{11}N$, $C_4H_{11}O$, C_4H_9N, $C_5H_{11}NO$, C_5H_9NO, $C_6H_{11}NO$, $C_6H_{13}NO_2$, $C_6H_{14}N_2O$, $C_6H_{15}N$, $C_7H_{13}NO$, $C_7H_{15}NO_2$, $C_8H_{11}N$, $C_8H_{11}NO$, $C_8H_6O_3$, $C_9H_{11}NO$, CH_3NO, CH_4O_2, CH_5N, CH_5NO, CH_5O, CH_5O_2, CH_6NO, CH_6O_2, CH_7NO
High Acc. Mass Tolerance	5 ppm
Low Acc. Mass Tolerance	0.5 Da
S/N Threshold	10
Use DIA Scans for Search	True
Compound Class Scoring	
1. General Settings	
Compound Classes	**Table S10**
S/N Threshold	10
High Acc. Mass Tolerance	5 ppm
Low Acc. Mass Tolerance	0.5 Da
Use Full MS Tree	False
Allow DIA Scoring	True
Differential Analysis	
1. General Settings	
Log10 Transform Values	True
2. Peak Rating Contributions	
Update Peak Rating	True
Area Contribution	3
CV Contribution	10
FWHM to Base Contribution	5
Jaggedness Contribution	5
Modality Contribution	5
Zig-Zag Index Contribution	5
Descriptive Statistics	

**Table S9.** Compound database imported into the *Search Mass Lists* node			
Compound Name	Molecular Formula	Exact Mass	SMILES
Clonazolam	C17H12ClN5O2	353.0680	CC1=NN=C2N1C3=C(C=C(C=C3)[N+](=O)[O-])C(=NC2)C4=CC=CC=C4Cl
Etizolam	C17H15ClN4S	342.0706	CCC1=CC2=C(S1)N3C(=NN=C3CN=C2C4=CC=CC=C4Cl)C
Flualprazolam	C17H12ClFN4	326.0735	CC1=NN=C2N1C3=C(C=C(C=C3)Cl)C(=NC2)C4=CC=CC=C4F
Flubromazolam	C17H12BrFN4	370.0229	CC1=NN=C2N1C3=C(C=C(C=C3)Br)C(=NC2)C4=CC=CC=C4F
Desalkylflurazepam (Norfludiazepam)	C15H10ClFN2O	288.0466	C1C(=O)NC2=C(C=C(C=C2)Cl)C(=N1)C3=CC=CC=C3F
Bromazolam	C17H13BrN4	352.0324	CC1=NN=C2N1C3=C(C=C(C=C3)Br)C(=NC2)C4=CC=CC=C4
Adinazolam	C19H18ClN5	351.1251	CN(C)CC1=NN=C2N1C3=C(C=C(C=C3)Cl)C(=NC2)C4=CC=CC=C4
Deschloroetizolam	C17H16N4S	308.1096	CCC1=CC2=C(S1)N3C(=NN=C3CN=C2C4=CC=CC=C4)C
Meclonazepam	C16H12ClN3O3	329.0567	CC1C(=O)NC2=C(C=C(C=C2)[N+](=O)[O-])C(=N1)C3=CC=CC=C3Cl
Flubromazepam	C15H10BrFN2O	331.9961	C1C(=O)NC2=C(C=C(C=C2)Br)C(=N1)C3=CC=CC=C3F
Pyrazolam	C16H12BrN5	353.0276	CC1=NN=C2N1C3=C(C=C(C=C3)Br)C(=NC2)C4=CC=CC=N4
Diclazepam (2-Chlorodiazepam)	C16H12Cl2N2O	318.0327	CN1C(=O)CN=C(C2=C1C=CC(=C2)Cl)C3=CC=CC=C3Cl
Bromazepam	C14H10BrN3O	315.0007	C1C(=O)NC2=C(C=C(C=C2)Br)C(=N1)C3=CC=CC=N3
Metizolam (Desmethyletizolam)	C16H13ClN4S	328.0549	CCC1=CC2=C(S1)N3C=NN=C3CN=C2C4=CC=CC=C4Cl
8-Aminoclonazolam	C17H14ClN5	323.0938	CC1=NN=C2N1C3=C(C=C(C=C3)N)C(=NC2)C4=CC=CC=C4Cl
Phenazepam	C15H10BrClN2O	347.9665	C1C(=O)NC2=C(C=C(C=C2)Br)C(=N1)C3=CC=CC=C3Cl
Fluclotizolam	C15H10ClFN4S	332.0299	CC1=NN=C2N1C3=C(C=C(S3)Cl)C(=NC2)C4=CC=CC=C4F
N-Pyrrolidino Etonitazene (Etonitazepyne)	C22H26N4O3	394.2005	CCOC1=CC=C(C=C1)CC2=NC3=C(N2CCN4CCCC4)C=CC(=C3)[N+](=O)[O-]
Etodesnitazene	C22H29N3O	351.2311	CCN(CC)CCN1C2=CC=CC=C2N=C1CC3=CC=C(C=C3)OCC
Protonitazene	C23H30N4O3	410.2318	CCCOC1=CC=C(C=C1)CC2=NC3=C(N2CCN(CC)CC)C=CC(=C3)[N+](=O)[O-]
Dipyanone	C23H29NO	335.2249	CCC(=O)C(CC(C)N1CCCC1)(C2=CC=CC=C2)C3=CC=CC=C3
N-Piperidinyl Etonitazene (Etonitazepipne)	C23H28N4O3	408.2161	CCOC1=CC=C(C=C1)CC2=NC3=C(N2CCN4CCCCC4)C=CC(=C3)[N+](=O)[O-]
Butonitazene	C24H32N4O3	424.2474	CCCCOC1=CC=C(C=C1)CC2=NC3=C(N2CCN(CC)CC)C=CC(=C3)[N+](=O)[O-]
Eutylone	C13H17NO3	235.1208	CCC(C(=O)C1=CC2=C(C=C1)OCO2)NCC
α-Pyrrolidinoisohexanophenone (α-PiHP)	C16H23NO	245.1780	CC(C)CC(C(=O)C1=CC=CC=C1)N2CCCC2
Deschloroketamine	C13H17NO	203.1310	CNC1(CCCCC1=O)C2=CC=CC=C2
3-Hydroxy PCP (HO-PCP) (3-Hydroxyphencyclidine)	C17H25NO	259.1936	C1CCC(CC1)(C2=CC(=CC=C2)O)N3CCCCC3
2-Fluoro Deschloroketamine (Fluoroketamine)	C13H16FNO	221.1216	CNC1(CCCCC1=O)C2=CC=CC=C2F
3-Methoxy PCP	C18H27NO	273.2093	COC1=CC=CC(=C1)C2(CCCCC2)N3CCCCC3
3-Chloro PCP (Cl-PCP)	C17H24ClN	277.1597	C1CCC(CC1)(C2=CC(=CC=C2)Cl)N3CCCCC3
N-Ethylhexedrone (Hexen)	C14H21NO	219.1623	CCCCC(C(=O)C1=CC=CC=C1)NCC
Pentylone	C13H17NO3	235.1208	CCCC(C(=O)C1=CC2=C(C=C1)OCO2)NC
α-Pyrrolidinohexiophenone (α-PHP)	C16H23NO	245.1780	CCCCC(C(=O)C1=CC=CC=C1)N2CCCC2
N-Ethyl Pentylone	C14H19NO3	249.1365	CCCC(C(=O)C1=CC2=C(C=C1)OCO2)NCC
3,4-Methylenedioxy-N-Benzylcathinone (BDMP, Benzylone)	C17H17NO3	283.1208	CC(C(=O)C1=CC2=C(C=C1)OCO2)NCC3=CC=CC=C3
4-Methoxy PCP	C18H27NO	273.2093	COC1=CC=C(C=C1)C2(CCCCC2)N3CCCCC3
Xylazine	C12H16N2S	220.1034	CC1=C(C(=CC=C1)C)NC2=NCCCS2
N-Butyl Pentylone	C16H23NO3	277.1678	CCCCNC(CCC)C(=O)C1=CC2=C(C=C1)OCO2
3,4-Methylenedioxy PV8	C18H25NO3	303.1834	CCCCCC(C(=O)C1=CC2=C(C=C1)OCO2)N3CCCC3
N-Ethyl Pentedrone	C13H19NO	205.1467	CCCC(C(=O)C1=CC=CC=C1)NCC
N-Ethyl Deschloroketamine (Deschloro-N-ethyl Ketamine)	C14H19NO	217.1467	CCNC1(CCCCC1=O)C2=CC=CC=C2
4-Chloro-α-Pyrrolidinovalerophenone (4Cl-α-PVP)	C16H22ClNO	279.1390	CCCCC(C(=O)C1=CC=C(C=C1)Cl)N2CCCC2

Table S9. Compound database imported into the *Search Mass Lists* node (continued)

Compound Name	Molecular Formula	Exact Mass	SMILES
Pyrrolidinovalerophenone (α-PVP)	C15H21NO	231.1623	CCCC(C(=O)C1=CC=CC=C1)N2CCCC2
4-HO-DiPT (HO-DiPT)	C16H24N2O	260.1889	CC(C)N(CCC1=CNC2=C1C(=CC=C2)O)C(C)C
3-HO-PCE (HO-PCE)	C14H21NO	219.1623	CCNC1(CCCCC1)C2=CC(=CC=C2)O
5F-MDMB-PICA	C21H29FN2O3	376.2162	CC(C)(C)C(C(=O)OC)NC(=O)C1=CN(C2=CC=CC=C21)CCCCCF
MDMB-4en-PINACA	C20H27N3O3	357.2052	CC(C)(C)C(C(=O)OC)NC(=O)C1=NN(C2=CC=CC=C21)CCCC=C
4F-MDMB-BINACA (4F-MDMB-BUTINACA)	C19H26FN3O3	363.1958	CC(C)(C)C(C(=O)OC)NC(=O)C1=NN(C2=CC=CC=C21)CCCCF
ADB-BINACA	C21H24N4O2	364.1899	CC(C)(C)C(C(=O)N)NC(=O)C1=NN(C2=CC=CC=C21)CC3=CC=CC=C3
5F-MDMB-PINACA (5F-ADB)	C20H28FN3O3	377.2115	CC(C)(C)C(C(=O)OC)NC(=O)C1=NN(C2=CC=CC=C21)CCCCCF
4F-MDMB-BICA	C20H27FN2O3	362.2006	CC(C)(C)C(C(=O)OC)NC(=O)C1=CN(C2=CC=CC=C21)CCCCF
ADB-4en-PINACA	C19H26N4O2	342.2056	CC(C)(C)C(C(=O)N)NC(=O)C1=NN(C2=CC=CC=C21)CCCC=C
ADB-HEXINACA	C20H30N4O2	358.2369	CCCCCCN1C2=CC=CC=C2C(=N1)C(=O)NC(C(=O)N)C(C)(C)C
4F-ABINACA (4-Fluoro ABUTINACA)	C22H28FN3O	369.2216	C1C2CC3CC1CC(C2)(C3)NC(=O)C4=NN(C5=CC=CC=C54)CCCCF
ADB-BUTINACA	C18H26N4O2	330.2056	CCCCN1C2=CC=CC=C2C(=N1)C(=O)NC(C(=O)N)C(C)(C)C
ACHMINACA	C25H33N3O	391.2624	C1CCC(CC1)CN2C3=CC=CC=C3C(=N2)C(=O)NC45CC6CC(C4)CC(C6)C5
4-CN-CUMYL-BUTINACA	C22H24N4O	360.1950	CC(C)(C1=CC=CC=C1)NC(=O)C2=NN(C3=CC=CC=C32)CCCCC#N
FUB-AMB (MMB-FUBINACA)	C21H22FN3O3	383.1645	CC(C)C(C(=O)OC)NC(=O)C1=NN(C2=CC=CC=C21)CC3=CC=C(C=C3)F
5F-EDMB-PINACA	C21H30FN3O3	391.2271	CCOC(=O)C(C(C)(C)C)NC(=O)C1=NN(C2=CC=CC=C21)CCCCCF
5F-EDMB-PICA	C22H31FN2O3	390.2319	CCOC(=O)C(C(C)(C)C)NC(=O)C1=CN(C2=CC=CC=C21)CCCCCF
para-Methoxy Butyryl Fentanyl (4-Methoxybutyrfentanyl)	C24H32N2O2	380.2464	CCCC(=O)N(C1CCN(CC1)CCC2=CC=CC=C2)C3=CC=C(C=C3)OC
Acetyl Norfentanyl	C13H18N2O	218.1419	CC(=O)N(C1CCNCC1)C2=CC=CC=C2
α-Methyl Acetyl Fentanyl	C22H28N2O	336.2202	CC(CC1=CC=CC=C1)N2CCC(CC2)N(C3=CC=CC=C3)C(=O)C
Crotonyl Fentanyl	C23H28N2O	348.2202	CC=CC(=O)N(C1CCN(CC1)CCC2=CC=CC=C2)C3=CC=CC=C3
Acrylfentanyl	C22H26N2O	334.2045	C=CC(=O)N(C1CCN(CC1)CCC2=CC=CC=C2)C3=CC=CC=C3
Cyclopropyl Fentanyl	C23H28N2O	348.2202	C1CC1C(=O)N(C2CCN(CC2)CCC3=CC=CC=C3)C4=CC=CC=C4
Isobutyryl Fentanyl	C23H30N2O	350.2358	CC(C)C(=O)N(C1CCN(CC1)CCC2=CC=CC=C2)C3=CC=CC=C3
Methoxyacetyl Fentanyl	C22H28N2O2	352.2151	COCC(=O)N(C1CCN(CC1)CCC2=CC=CC=C2)C3=CC=CC=C3
Valeryl Fentanyl	C24H32N2O	364.2515	CCCCC(=O)N(C1CCN(CC1)CCC2=CC=CC=C2)C3=CC=CC=C3
(±)-cis-3-Methyl Fentanyl	C23H30N2O	350.2358	CCC(=O)N(C1CCN(CC1C)CCC2=CC=CC=C2)C3=CC=CC=C3
2'-Fluoro ortho-Fluorofentanyl	C22H26F2N2O	372.2013	CCC(=O)N(C1CCN(CC1)CCC2=CC=CC=C2F)C3=CC=CC=C3F
ortho-Fluorobutyryl Fentanyl	C23H29FN2O	368.2264	CCCC(=O)N(C1CCN(CC1)CCC2=CC=CC=C2)C3=CC=CC=C3F
ortho-Fluorofentanyl	C22H27FN2O	354.2107	CCC(=O)N(C1CCN(CC1)CCC2=CC=CC=C2)C3=CC=CC=C3F
ortho-Isopropyl Furanyl Fentanyl	C27H32N2O2	416.2464	CC(C)C1=CC=CC=C1N(C2CCN(CC2)CCC3=CC=CC=C3)C(=O)C4=CC=CO4
ortho-Methoxy Furanyl Fentanyl	C25H28N2O3	404.2100	COC1=CC=CC=C1N(C2CCN(CC2)CCC3=CC=CC=C3)C(=O)C4=CC=CO4
ortho-Methyl Furanyl Fentanyl	C25H28N2O2	388.2151	CC1=CC=CC=C1N(C2CCN(CC2)CCC3=CC=CC=C3)C(=O)C4=CC=CO4
meta-Fluorobutyryl Fentanyl	C23H29FN2O	368.2264	CCCC(=O)N(C1CCN(CC1)CCC2=CC=CC=C2)C3=CC(=CC=C3)F
(±)-cis-3-Methyl Butyryl Fentanyl	C24H32N2O	364.2515	CCCC(=O)N(C1CCN(CC1C)CCC2=CC=CC=C2)C3=CC=CC=C3
4'-Methyl Acetyl Fentanyl	C22H28N2O	336.2202	CC1=CC=C(C=C1)CCN2CCC(CC2)N(C3=CC=CC=C3)C(=O)C
para-Chloro Furanyl Fentanyl	C24H25ClN2O2	408.1605	C1CN(CCC1N(C2=CC=C(C=C2)Cl)C(=O)C3=CC=CO3)CCC4=CC=CC=C4
para-Methoxyfentanyl	C23H30N2O2	366.2307	CCC(=O)N(C1CCN(CC1)CCC2=CC=CC=C2)C3=CC=C(C=C3)OC
para-Methoxy Furanyl Fentanyl	C25H28N2O3	404.2100	COC1=CC=C(C=C1)N(C2CCN(CC2)CCC3=CC=CC=C3)C(=O)C4=CC=CO4
α-Methyl Butyryl Fentanyl	C24H32N2O	364.2515	CCCC(=O)N(C1CCN(CC1)C(C)CC2=CC=CC=C2)C3=CC=CC=C3
α-Methyl Thiofentanyl	C21H28N2OS	356.1922	CCC(=O)N(C1CCN(CC1)C(C)CC2=CC=CS2)C3=CC=CC=C3

**Table S9.** Compound database imported into the *Search Mass Lists* node (continued)			
Compound Name	Molecular Formula	Exact Mass	SMILES
Benzodioxole Fentanyl	C27H28N2O3	428.2100	C1CN(CCC1N(C2=CC=CC=C2)C(=O)C3=CC4=C(C=C3)OCO4)CCC5=CC=CC=C5
Phenyl Fentanyl	C26H28N2O	384.2202	C1CN(CCC1N(C2=CC=CC=C2)C(=O)C3=CC=CC=C3)CCC4=CC=CC=C4
Benzyl Fentanyl	C21H26N2O	322.2045	CCC(=O)N(C1CCN(CC1)CC2=CC=CC=C2)C3=CC=CC=C3
N-Benzyl Furanyl Norfentanyl	C23H24N2O2	360.1838	C1CN(CCC1N(C2=CC=CC=C2)C(=O)C3=CC=CO3)CC4=CC=CC=C4
Cyclohexyl Fentanyl	C26H34N2O	390.2671	C1CCC(CC1)C(=O)N(C2CCN(CC2)CCC3=CC=CC=C3)C4=CC=CC=C4
Cyclopentyl Fentanyl	C25H32N2O	376.2515	C1CCC(C1)C(=O)N(C2CCN(CC2)CCC3=CC=CC=C3)C4=CC=CC=C4
meta-Methylfentanyl	C23H30N2O	350.2358	CCC(=O)N(C1CCN(CC1)CCC2=CC=CC=C2)C3=CC=CC(=C3)C
N-Methyl Norcarfentanil	C17H24N2O3	304.1787	CCC(=O)N(C1=CC=CC=C1)C2(CCN(CC2)C)C(=O)OC
Norcarfentanil	C16H22N2O3	290.1630	CCC(=O)N(C1=CC=CC=C1)C2(CCNCC2)C(=O)OC
ortho-Methyl Acrylfentanyl	C23H28N2O	348.2202	CC1=CC=CC=C1N(C2CCN(CC2)CCC3=CC=CC=C3)C(=O)C=C
ortho-Methyl Methoxyacetyl Fentanyl	C23H30N2O2	366.2307	CC1=CC=CC=C1N(C2CCN(CC2)CCC3=CC=CC=C3)C(=O)COC
para-Chloroisobutyryl Fentanyl	C23H29ClN2O	384.1968	CC(C)C(=O)N(C1CCN(CC1)CCC2=CC=CC=C2)C3=CC=C(C=C3)Cl
Tetrahydrofuran Fentanyl	C24H30N2O2	378.2307	C1CC(OC1)C(=O)N(C2CCN(CC2)CCC3=CC=CC=C3)C4=CC=CC=C4
2,2,3,3-Tetramethyl-cyclopropyl Fentanyl	C27H36N2O	404.2828	CC1(C(C1(C)C)C(=O)N(C2CCN(CC2)CCC3=CC=CC=C3)C4=CC=CC=C4)C
Thienyl Fentanyl	C19H24N2OS	328.1609	CCC(=O)N(C1CCN(CC1)CC2=CC=CS2)C3=CC=CC=C3
Thiophene Fentanyl	C24H26N2OS	390.1766	C1CN(CCC1N(C2=CC=CC=C2)C(=O)C3=CC=CS3)CCC4=CC=CC=C4
Thiofentanyl	C20H26N2OS	342.1766	CCC(=O)N(C1CCN(CC1)CCC2=CC=CS2)C3=CC=CC=C3
ortho-Methyl Acetyl Fentanyl	C22H28N2O	336.2202	CC1=CC=CC=C1N(C2CCN(CC2)CCC3=CC=CC=C3)C(=O)C
α'-Methoxy Fentanyl	C23H30N2O2	366.2307	CC(C(=O)N(C1CCN(CC1)CCC2=CC=CC=C2)C3=CC=CC=C3)OC
α'-Methyl Butyryl Fentanyl	C24H32N2O	364.2515	CCC(C)C(=O)N(C1CCN(CC1)CCC2=CC=CC=C2)C3=CC=CC=C3
Cyclobutyl Fentanyl	C24H30N2O	362.2358	C1CC(C1)C(=O)N(C2CCN(CC2)CCC3=CC=CC=C3)C4=CC=CC=C4
meta-Fluorofentanyl	C22H27FN2O	354.2107	CCC(=O)N(C1CCN(CC1)CCC2=CC=CC=C2)C3=CC(=CC=C3)F
N-Methyl Cyclopropyl Norfentanyl	C16H22N2O	258.1732	CN1CCC(CC1)N(C2=CC=CC=C2)C(=O)C3CC3
(±)-trans-3-Methyl Thiofentanyl	C21H28N2OS	356.1922	CCC(=O)N(C1CCN(CC1C)CCC2=CC=CS2)C3=CC=CC=C3
4'-Fluoro, para-Fluoro (±)-trans-3-Methyl Fentanyl	C23H28F2N2O	386.2170	CCC(=O)N(C1CCN(CC1C)CCC2=CC=C(C=C2)F)C3=CC=C(C=C3)F
4'-Methyl Fentanyl	C23H30N2O	350.2358	CCC(=O)N(C1CCN(CC1)CCC2=CC=C(C=C2)C)C3=CC=CC=C3
Alfentanil	C21H32N6O3	416.2536	CCC(=O)N(C1=CC=CC=C1)C2(CCN(CC2)CCN3C(=O)N(N=N3)CC)COC
Benzyl Carfentanil	C23H28N2O3	380.2100	CCC(=O)N(C1=CC=CC=C1)C2(CCN(CC2)CC3=CC=CC=C3)C(=O)OC
Benzyl Acrylfentanyl	C21H24N2O	320.1889	C=CC(=O)N(C1CCN(CC1)CC2=CC=CC=C2)C3=CC=CC=C3
β'-Phenyl Fentanyl (3-Phenylpropanoylfentanyl)	C28H32N2O	412.2515	C1CN(CCC1N(C2=CC=CC=C2)C(=O)CCC3=CC=CC=C3)CCC4=CC=CC=C4
β-methyl Acetyl Fentanyl	C22H28N2O	336.2202	CC(CN1CCC(CC1)N(C2=CC=CC=C2)C(=O)C)C3=CC=CC=C3
Ethoxyacetyl Fentanyl	C23H30N2O2	366.2307	CCOCC(=O)N(C1CCN(CC1)CCC2=CC=CC=C2)C3=CC=CC=C3
Fentanyl Carbamate	C22H28N2O2	352.2151	CCOC(=O)N(C1CCN(CC1)CCC2=CC=CC=C2)C3=CC=CC=C3
Furanyl Fentanyl 3-Furancarboxamide	C24H26N2O2	374.1994	C1CN(CCC1N(C2=CC=CC=C2)C(=O)C3=COC=C3)CCC4=CC=CC=C4
Furanylethyl Fentanyl	C20H26N2O2	326.1994	CCC(=O)N(C1CCN(CC1)CCC2=CC=CO2)C3=CC=CC=C3
Isovaleryl Fentanyl	C24H32N2O	364.2515	CC(C)CC(=O)N(C1CCN(CC1)CCC2=CC=CC=C2)C3=CC=CC=C3
meta-Fluoroisobutyryl Fentanyl	C23H29FN2O	368.2264	CC(C)C(=O)N(C1CCN(CC1)CCC2=CC=CC=C2)C3=CC(=CC=C3)F
meta-Fluoro Methoxyacetyl Fentanyl	C22H27FN2O2	370.2057	COCC(=O)N(C1CCN(CC1)CCC2=CC=CC=C2)C3=CC(=CC=C3)F
meta-Methyl Furanyl Fentanyl	C25H28N2O2	388.2151	CC1=CC(=CC=C1)N(C2CCN(CC2)CCC3=CC=CC=C3)C(=O)C4=CC=CO4
meta-Methyl Methoxyacetyl Fentanyl	C23H30N2O2	366.2307	CC1=CC(=CC=C1)N(C2CCN(CC2)CCC3=CC=CC=C3)C(=O)COC
Methacrylfentanyl	C23H28N2O	348.2202	CC(=C)C(=O)N(C1CCN(CC1)CCC2=CC=CC=C2)C3=CC=CC=C3
ortho-Fluoro Acrylfentanyl	C22H25FN2O	352.1951	C=CC(=O)N(C1CCN(CC1)CCC2=CC=CC=C2)C3=CC=CC=C3F

Table S9. Compound database imported into the *Search Mass Lists* node (continued)

Compound Name	Molecular Formula	Exact Mass	SMILES
ortho-Fluoro Furanyl Fentanyl	C24H25FN2O2	392.1900	C1CN(CCC1N(C2=CC=CC=C2F)C(=O)C3=CC=CO3)CCC4=CC=CC=C4
ortho-Fluoroisobutyryl Fentanyl	C23H29FN2O	368.2264	CC(C)C(=O)N(C1CCN(CC1)CCC2=CC=CC=C2)C3=CC=CC=C3F
ortho-Methoxy Butyryl Fentanyl	C24H32N2O2	380.2464	CCCC(=O)N(C1CCN(CC1)CCC2=CC=CC=C2)C3=CC=CC=C3OC
ortho-Methyl Cyclopropyl Fentanyl	C24H30N2O	362.2358	CC1=CC=CC=C1N(C2CCN(CC2)CCC3=CC=CC=C3)C(=O)C4CC4
ortho-Methylfentanyl	C23H30N2O	350.2358	CCC(=O)N(C1CCN(CC1)CCC2=CC=CC=C2)C3=CC=CC=C3C
ortho-Methyl Phenyl Fentanyl	C27H30N2O	398.2358	CC1=CC=CC=C1N(C2CCN(CC2)CCC3=CC=CC=C3)C(=O)C4=CC=CC=C4
para-Chlorofentanyl	C22H27ClN2O	370.1812	CCC(=O)N(C1CCN(CC1)CCC2=CC=CC=C2)C3=CC=C(C=C3)Cl
para-Chloro Acrylfentanyl	C22H25ClN2O	368.1655	C=CC(=O)N(C1CCN(CC1)CCC2=CC=CC=C2)C3=CC=C(C=C3)Cl
para-Chlorobutyryl Fentanyl	C23H29ClN2O	384.1968	CCCC(=O)N(C1CCN(CC1)CCC2=CC=CC=C2)C3=CC=C(C=C3)Cl
para-Chloro Cyclobutyl Fentanyl	C24H29ClN2O	396.1968	C1CC(C1)C(=O)N(C2CCN(CC2)CCC3=CC=CC=C3)C4=CC=C(C=C4)Cl
para-Chloro Cyclopentyl Fentanyl	C25H31ClN2O	410.2125	C1CCC(C1)C(=O)N(C2CCN(CC2)CCC3=CC=CC=C3)C4=CC=C(C=C4)Cl
para-Chloro Methoxyacetyl Fentanyl	C22H27ClN2O2	386.1761	COCC(=O)N(C1CCN(CC1)CCC2=CC=CC=C2)C3=CC=C(C=C3)Cl
para-Chloro Valeryl Fentanyl	C24H31ClN2O	398.2125	CCCCC(=O)N(C1CCN(CC1)CCC2=CC=CC=C2)C3=CC=C(C=C3)Cl
para-Chloro Cyclopropyl Fentanyl	C23H27ClN2O	382.1812	C1CC1C(=O)N(C2CCN(CC2)CCC3=CC=CC=C3)C4=CC=C(C=C4)Cl
para-Fluoro Acrylfentanyl	C22H25FN2O	352.1951	C=CC(=O)N(C1CCN(CC1)CCC2=CC=CC=C2)C3=CC=C(C=C3)F
para-Fluoro Crotonyl Fentanyl	C23H27FN2O	366.2107	CC=CC(=O)N(C1CCN(CC1)CCC2=CC=CC=C2)C3=CC=C(C=C3)F
para-Fluoro Cyclopentyl Fentanyl	C25H31FN2O	394.2420	C1CCC(C1)C(=O)N(C2CCN(CC2)CCC3=CC=CC=C3)C4=CC=C(C=C4)F
para-Fluoro Cyclopropyl Fentanyl	C23H27FN2O	366.2107	C1CC1C(=O)N(C2CCN(CC2)CCC3=CC=CC=C3)C4=CC=C(C=C4)F
para-Fluoro Furanyl Fentanyl 3-Furancarboxamide	C24H25FN2O2	392.1900	C1CN(CCC1N(C2=CC=C(C=C2)F)C(=O)C3=COC=C3)CCC4=CC=CC=C4
para-Fluoro Furanyl Fentanyl	C24H25FN2O2	392.1900	C1CN(CCC1N(C2=CC=C(C=C2)F)C(=O)C3=CC=CO3)CCC4=CC=CC=C4
para-Fluoro Methoxyacetyl Fentanyl	C22H27FN2O2	370.2057	COCC(=O)N(C1CCN(CC1)CCC2=CC=CC=C2)C3=CC=C(C=C3)F
para-Fluoro Tetrahydrofuran Fentanyl	C24H29FN2O2	396.2213	C1CC(OC1)C(=O)N(C2CCN(CC2)CCC3=CC=CC=C3)C4=CC=C(C=C4)F
para-Fluoro Valeryl Fentanyl	C24H31FN2O	382.2420	CCCCC(=O)N(C1CCN(CC1)CCC2=CC=CC=C2)C3=CC=C(C=C3)F
para-Fluoroacetyl Fentanyl	C21H25FN2O	340.1951	CC(=O)N(C1CCN(CC1)CCC2=CC=CC=C2)C3=CC=C(C=C3)F
para-Methoxy Acrylfentanyl	C23H28N2O2	364.2151	COC1=CC=C(C=C1)N(C2CCN(CC2)CCC3=CC=CC=C3)C(=O)C=C
para-Methoxy Tetrahydrofuran Fentanyl	C25H32N2O3	408.2413	COC1=CC=C(C=C1)N(C2CCN(CC2)CCC3=CC=CC=C3)C(=O)C4CCCO4
para-Methoxy Valeryl Fentanyl	C25H34N2O2	394.2620	CCCCC(=O)N(C1CCN(CC1)CCC2=CC=CC=C2)C3=CC=C(C=C3)OC
para-Methylfentanyl	C23H30N2O	350.2358	CCC(=O)N(C1CCN(CC1)CCC2=CC=CC=C2)C3=CC=C(C=C3)C
para-Methyl Acetyl Fentanyl	C22H28N2O	336.2202	CC1=CC=C(C=C1)N(C2CCN(CC2)CCC3=CC=CC=C3)C(=O)C
para-Methyl Acrylfentanyl	C23H28N2O	348.2202	CC1=CC=C(C=C1)N(C2CCN(CC2)CCC3=CC=CC=C3)C(=O)C=C
para-Methyl Cyclopropyl Fentanyl	C24H30N2O	362.2358	CC1=CC=C(C=C1)N(C2CCN(CC2)CCC3=CC=CC=C3)C(=O)C4CC4
para-Methyl Furanyl Fentanyl	C25H28N2O2	388.2151	CC1=CC=C(C=C1)N(C2CCN(CC2)CCC3=CC=CC=C3)C(=O)C4=CC=CO4
para-Methyl Isobutyryl Fentanyl	C24H32N2O	364.2515	CC1=CC=C(C=C1)N(C2CCN(CC2)CCC3=CC=CC=C3)C(=O)C(C)C
para-Methyl Methoxyacetyl Fentanyl	C23H30N2O2	366.2307	CC1=CC=C(C=C1)N(C2CCN(CC2)CCC3=CC=CC=C3)C(=O)COC
para-Methyl Tetrahydrofuran Fentanyl	C25H32N2O2	392.2464	CC1=CC=C(C=C1)N(C2CCN(CC2)CCC3=CC=CC=C3)C(=O)C4CCCO4
Phenylacetyl Fentanyl	C27H30N2O	398.2358	C1CN(CCC1N(C2=CC=CC=C2)C(=O)CC3=CC=CC=C3)CCC4=CC=CC=C4
Pivaloyl Fentanyl	C24H32N2O	364.2515	CC(C)(C)C(=O)N(C1CCN(CC1)CCC2=CC=CC=C2)C3=CC=CC=C3
Remifentanil	C20H28N2O5	376.1998	CCC(=O)N(C1=CC=CC=C1)C2(CCN(CC2)CCC(=O)OC)C(=O)OC
Sufentanil	C22H30N2O2S	386.2028	CCC(=O)N(C1=CC=CC=C1)C2(CCN(CC2)CCC3=CC=CS3)COC
Tetrahydrofuran Fentanyl 3-Tetrahydrofurancarboxamide	C24H30N2O2	378.2307	C1CN(CCC1N(C2=CC=CC=C2)C(=O)C3CCOC3)CCC4=CC=CC=C4
(±)-cis-3-Methyl Thiofentanyl	C21H28N2OS	356.1922	CCC(=O)N(C1CCN(CC1C)CCC2=CC=CS2)C3=CC=CC=C3
β-Hydroxythiofentanyl	C20H26N2O2S	358.1715	CCC(=O)N(C1CCN(CC1)CC(C2=CC=CS2)O)C3=CC=CC=C3

Table S9. Compound database imported into the *Search Mass Lists* node (continued)

Compound Name	Molecular Formula	Exact Mass	SMILES
4-ANPP (Despropionylfentanyl)	C19H24N2	280.1939	C1CN(CCC1NC2=CC=CC=C2)CCC3=CC=CC=C3
β-Methyl Fentanyl	C23H30N2O	350.2358	CCC(=O)N(C1CCN(CC1)CC(C)C2=CC=CC=C2)C3=CC=CC=C3
(±)-trans-3-Methyl Fentanyl	C23H30N2O	350.2358	CCC(=O)N(C1CCN(CC1C)CCC2=CC=CC=C2)C3=CC=CC=C3
para-Fluorobutyryl Fentanyl	C23H29FN2O	368.2264	CCCC(=O)N(C1CCN(CC1)CCC2=CC=CC=C2)C3=CC=C(C=C3)F
FIBF (4-Fluoroisobutyryl Fentanyl)	C23H29FN2O	368.2264	CC(C)C(=O)N(C1CCN(CC1)CCC2=CC=CC=C2)C3=CC=C(C=C3)F
α-Methyl Fentanyl	C23H30N2O	350.2358	CCC(=O)N(C1CCN(CC1)C(C)CC2=CC=CC=C2)C3=CC=CC=C3
Ocfentanil	C22H27FN2O2	370.2057	COCC(=O)N(C1CCN(CC1)CCC2=CC=CC=C2)C3=CC=CC=C3F
para-Fluorofentanyl	C22H27FN2O	354.2107	CCC(=O)N(C1CCN(CC1)CCC2=CC=CC=C2)C3=CC=C(C=C3)F
Butyryl Norfentanyl	C15H22N2O	246.1732	CCCC(=O)N(C1CCNCC1)C2=CC=CC=C2
Butyryl Fentanyl	C23H30N2O	350.2358	CCCC(=O)N(C1CCN(CC1)CCC2=CC=CC=C2)C3=CC=CC=C3
Furanyl Fentanyl	C24H26N2O2	374.1994	C1CN(CCC1N(C2=CC=CC=C2)C(=O)C3=CC=CO3)CCC4=CC=CC=C4
Norsufentanil	C16H24N2O2	276.1838	CCC(=O)N(C1=CC=CC=C1)C2(CCNCC2)COC
Acetyl Fentanyl	C21H26N2O	322.2045	CC(=O)N(C1CCN(CC1)CCC2=CC=CC=C2)C3=CC=CC=C3
4’-Fluorofentanyl	C22H27FN2O	354.2107	CCC(=O)N(C1CCN(CC1)CCC2=CC=C(C=C2)F)C3=CC=CC=C3
4-Phenyl Fentanyl	C28H32N2O	412.2515	CCC(=O)N(C1=CC=CC=C1)C2(CCN(CC2)CCC3=CC=CC=C3)C4=CC=CC=C4
Cyclopentenyl Fentanyl	C25H30N2O	374.2358	C1CC=C(C1)C(=O)N(C2CCN(CC2)CCC3=CC=CC=C3)C4=CC=CC=C4
para-Methyl Cyclopentyl Fentanyl	C26H34N2O	390.2671	CC1=CC=C(C=C1)N(C2CCN(CC2)CCC3=CC=CC=C3)C(=O)C4CCCC4
meta-Methyl Cyclopropyl Fentanyl	C24H30N2O	362.2358	CC1=CC(=CC=C1)N(C2CCN(CC2)CCC3=CC=CC=C3)C(=O)C4CC4
para-Methyl Butyryl Fentanyl	C24H32N2O	364.2515	CCCC(=O)N(C1CCN(CC1)CCC2=CC=CC=C2)C3=CC=C(C=C3)C
para-Methoxy Acetyl Fentanyl	C22H28N2O2	352.2151	CC(=O)N(C1CCN(CC1)CCC2=CC=CC=C2)C3=CC=C(C=C3)OC
para-Methoxy Methoxyacetyl Fentanyl	C23H30N2O3	382.2256	COCC(=O)N(C1CCN(CC1)CCC2=CC=CC=C2)C3=CC=C(C=C3)OC
Tetrahydrothiophene Fentanyl	C24H30N2OS	394.2079	C1CC(SC1)C(=O)N(C2CCN(CC2)CCC3=CC=CC=C3)C4=CC=CC=C4
N,N-Dimethylamido-Despropionyl Fentanyl	C22H29N3O	351.2311	CN(C)C(=O)N(C1CCN(CC1)CCC2=CC=CC=C2)C3=CC=CC=C3
Hexanoyl Fentanyl	C25H34N2O	378.2671	CCCCCC(=O)N(C1CCN(CC1)CCC2=CC=CC=C2)C3=CC=CC=C3
Heptanoyl Fentanyl	C26H36N2O	392.2828	CCCCCCC(=O)N(C1CCN(CC1)CCC2=CC=CC=C2)C3=CC=CC=C3
β-Hydroxythioacetylfentanyl	C19H24N2O2S	344.1558	CC(=O)N(C1CCN(CC1)CC(C2=CC=CS2)O)C3=CC=CC=C3
2,3-seco-Fentanyl (Secofentanyl)	C22H30N2O	338.2358	CCC(=O)N(C1=CC=CC=C1)C(C)CCN(C)CCC2=CC=CC=C2
Senecioylfentanyl	C24H30N2O	362.2358	CC(=CC(=O)N(C1CCN(CC1)CCC2=CC=CC=C2)C3=CC=CC=C3)C
Phenoxyacetyl Fentanyl	C27H30N2O2	414.2307	C1CN(CCC1N(C2=CC=CC=C2)C(=O)COC3=CC=CC=C3)CCC4=CC=CC=C4
Fentanyl Methyl Carbamate	C21H26N2O2	338.1994	COC(=O)N(C1CCN(CC1)CCC2=CC=CC=C2)C3=CC=CC=C3
β-Hydroxy Fentanyl	C22H28N2O2	352.2151	CCC(=O)N(C1CCN(CC1)CC(C2=CC=CC=C2)O)C3=CC=CC=C3
N-(3-Ethylindole) Norfentanyl	C24H29N3O	375.2311	CCC(=O)N(C1CCN(CC1)CCC2=CNC3=CC=CC=C32)C4=CC=CC=C4
U-47700	C16H22Cl2N2O	328.1109	CN(C)C1CCCCC1N(C)C(=O)C2=CC(=C(C=C2)Cl)Cl
U-49900	C18H26Cl2N2O	356.1422	CCN(CC)C1CCCCC1N(C)C(=O)C2=CC(=C(C=C2)Cl)Cl
U-48800	C17H24Cl2N2O	342.1266	CN(C)C1CCCCC1N(C)C(=O)CC2=C(C=C(C=C2)Cl)Cl
Isopropyl U-47700	C18H26Cl2N2O	356.1422	CC(C)N(C1CCCCC1N(C)C)C(=O)C2=CC(=C(C=C2)Cl)Cl
AH 7921	C16H22Cl2N2O	328.1109	CN(C)C1(CCCCC1)CNC(=O)C2=CC(=C(C=C2)Cl)Cl
MT-45	C24H32N2	348.2565	C1CCC(CC1)N2CCN(CC2)C(CC3=CC=CC=C3)C4=CC=CC=C4
2-Fluoro MT-45	C24H31FN2	366.2471	C1CCC(CC1)N2CCN(CC2)C(CC3=CC=CC=C3)C4=CC=CC=C4F
Furanyl Norfentanyl	C16H18N2O2	270.1368	C1CNCCC1N(C2=CC=CC=C2)C(=O)C3=CC=CO3
U-48520	C16H23ClN2O	294.1499	CN(C)C1CCCCC1N(C)C(=O)C2=CC=C(C=C2)Cl
U-50488	C19H26Cl2N2O	368.1422	CN(C1CCCCC1N2CCCC2)C(=O)CC3=CC(=C(C=C3)Cl)Cl

**Table S9.** Compound database imported into the *Search Mass Lists* node (continued)			
Compound Name	Molecular Formula	Exact Mass	SMILES
U-51754	C17H24Cl2N2O	342.1266	CN(C)C1CCCCC1N(C)C(=O)CC2=CC(=C(C=C2)Cl)Cl
U-47931E (Bromadoline)	C15H21BrN2O	324.0837	CN(C)C1CCCCC1NC(=O)C2=CC=C(C=C2)Br
U-48753E (Eclanamine)	C16H22Cl2N2O	328.1109	CCC(=O)N(C1CCCC1N(C)C)C2=CC(=C(C=C2)Cl)Cl
meta-Fluoro Furanyl Fentanyl	C24H25FN2O2	392.1900	C1CN(CCC1N(C2=CC(=CC=C2)F)C(=O)C3=CC=CO3)CCC4=CC=CC=C4
3,4-Methylenedioxy U-47700	C17H24N2O3	304.1787	CN(C)C1CCCCC1N(C)C(=O)C2=CC3=C(C=C2)OCO3
3,4-Ethylenedioxy U-51754	C19H28N2O3	332.2100	CN(C)C1CCCCC1N(C)C(=O)CC2=CC3=C(C=C2)OCCO3
3,4-Ethylenedioxy U-47700	C18H26N2O3	318.1943	CN(C)C1CCCCC1N(C)C(=O)C2=CC3=C(C=C2)OCCO3
N-Methyl U-47931E	C16H23BrN2O	338.0994	CN(C)C1CCCCC1N(C)C(=O)C2=CC=C(C=C2)Br
Propyl U-47700	C18H26Cl2N2O	356.1422	CCCN(C1CCCCC1N(C)C)C(=O)C2=CC(=C(C=C2)Cl)Cl
4-Phenyl U-51754	C23H30N2O	350.2358	CN(C)C1CCCCC1N(C)C(=O)CC2=CC=C(C=C2)C3=CC=CC=C3
N-Desmethyl U-47700	C15H20Cl2N2O	314.0953	CNC1CCCCC1N(C)C(=O)C2=CC(=C(C=C2)Cl)Cl
N,N-Didesmethyl U-47700	C14H18Cl2N2O	300.0796	CN(C1CCCCC1N)C(=O)C2=CC(=C(C=C2)Cl)Cl
3'-Methyl Acetyl Fentanyl	C22H28N2O	336.2202	CC1=CC(=CC=C1)CCN2CCC(CC2)N(C3=CC=CC=C3)C(=O)C
2'-Methyl Acetyl Fentanyl	C22H28N2O	336.2202	CC1=CC=CC=C1CCN2CCC(CC2)N(C3=CC=CC=C3)C(=O)C
meta-Methyl Acetyl Fentanyl	C22H28N2O	336.2202	CC1=CC(=CC=C1)N(C2CCN(CC2)CCC3=CC=CC=C3)C(=O)C
2'-Methyl Fentanyl	C23H30N2O	350.2358	CCC(=O)N(C1CCN(CC1)CCC2=CC=CC=C2C)C3=CC=CC=C3
3'-Methyl Fentanyl	C23H30N2O	350.2358	CCC(=O)N(C1CCN(CC1)CCC2=CC=CC(=C2)C)C3=CC=CC=C3
Remifentanil Acid	C19H26N2O5	362.1842	CCC(=O)N(C1=CC=CC=C1)C2(CCN(CC2)CCC(=O)O)C(=O)OC
(±)-cis-3-Methyl Norfentanyl	C15H22N2O	246.1732	CCC(=O)N(C1CCNCC1C)C2=CC=CC=C2
N-Benzyl Phenyl Norfentanyl	C25H26N2O	370.2045	C1CN(CCC1N(C2=CC=CC=C2)C(=O)C3=CC=CC=C3)CC4=CC=CC=C4
para-Fluoro 4-ANBP	C18H21FN2	284.1689	C1CN(CCC1NC2=CC=C(C=C2)F)CC3=CC=CC=C3
Despropionyl para-Fluorofentanyl	C19H23FN2	298.1845	C1CN(CCC1NC2=CC=C(C=C2)F)CCC3=CC=CC=C3
Despropionyl meta-Methylfentanyl	C20H26N2	294.2096	CC1=CC(=CC=C1)NC2CCN(CC2)CCC3=CC=CC=C3
N-Benzyl para-Fluoro Norfentanyl	C21H25FN2O	340.1951	CCC(=O)N(C1CCN(CC1)CC2=CC=CC=C2)C3=CC=C(C=C3)F
Despropionyl 2'-Fluoro ortho-Fluorofentanyl	C19H22F2N2	316.1751	C1CN(CCC1NC2=CC=CC=C2F)CCC3=CC=CC=C3F
(±)-cis-Isofentanyl	C22H28N2O	336.2202	CCC(=O)N(C1CCN(CC1C)CC2=CC=CC=C2)C3=CC=CC=C3
N-Methyl Norfentanyl	C15H22N2O	246.1732	CCC(=O)N(C1CCN(CC1)C)C2=CC=CC=C2
N-Benzyl para-Fluoro Cyclopropyl Norfentanyl	C22H25FN2O	352.1951	C1CC1C(=O)N(C2CCN(CC2)CC3=CC=CC=C3)C4=CC=C(C=C4)F
para-Bromofentanyl	C22H27BrN2O	414.1307	CCC(=O)N(C1CCN(CC1)CCC2=CC=CC=C2)C3=CC=C(C=C3)Br
para-Toluoyl Fentanyl	C27H30N2O	398.2358	CC1=CC=C(C=C1)C(=O)N(C2CCN(CC2)CCC3=CC=CC=C3)C4=CC=CC=C4
N-(MDA) Fentanyl	C24H30N2O3	394.2256	CCC(=O)N(C1CCN(CC1)C(C)CC2=CC3=C(C=C2)OCO3)C4=CC=CC=C4
N-(Phentermine) Fentanyl	C24H32N2O	364.2515	CCC(=O)N(C1CCN(CC1)C(C)(C)CC2=CC=CC=C2)C3=CC=CC=C3
N-(6-APB) Fentanyl	C25H30N2O2	390.2307	CCC(=O)N(C1CCN(CC1)C(C)CC2=CC3=C(C=C2)C=CO3)C4=CC=CC=C4
N-(6-APDB) Fentanyl	C25H32N2O2	392.2464	CCC(=O)N(C1CCN(CC1)C(C)CC2=CC3=C(CCO3)C=C2)C4=CC=CC=C4
N-(2-APB) Fentanyl	C25H30N2O2	390.2307	CCC(=O)N(C1CCN(CC1)C(C)CC2=CC3=CC=CC=C3O2)C4=CC=CC=C4
2',5'-Dimethoxy Fentanyl	C24H32N2O3	396.2413	CCC(=O)N(C1CCN(CC1)CCC2=C(C=CC(=C2)OC)OC)C3=CC=CC=C3
N-(2C-E) Fentanyl	C26H36N2O3	424.2726	CCC1=CC(=C(C=C1OC)CCN2CCC(CC2)N(C3=CC=CC=C3)C(=O)CC)OC
N-(2C-B) Fentanyl	C24H31BrN2O3	474.1518	CCC(=O)N(C1CCN(CC1)CCC2=CC(=C(C=C2OC)Br)OC)C3=CC=CC=C3
N-(2C-C) Fentanyl	C24H31ClN2O3	430.2023	CCC(=O)N(C1CCN(CC1)CCC2=CC(=C(C=C2OC)Cl)OC)C3=CC=CC=C3
N-(2C-P) Fentanyl	C27H38N2O3	438.2882	CCCC1=CC(=C(C=C1OC)CCN2CCC(CC2)N(C3=CC=CC=C3)C(=O)CC)OC
N-(2C-T-7) Fentanyl	C27H38N2O3S	470.2603	CCCSC1=C(C=C(C(=C1)OC)CCN2CCC(CC2)N(C3=CC=CC=C3)C(=O)CC)OC
N-(2C-D) Fentanyl	C25H34N2O3	410.2569	CCC(=O)N(C1CCN(CC1)CCC2=C(C=C(C(=C2)OC)C)OC)C3=CC=CC=C3

**Table S9.** Compound database imported into the *Search Mass Lists* node (continued)			
Compound Name	Molecular Formula	Exact Mass	SMILES
N-(2C-G) Fentanyl	C26H36N2O3	424.2726	CCC(=O)N(C1CCN(CC1)CCC2=CC(=C(C(=C2OC)C)C)OC)C3=CC=CC=C3
N-(2C-I) Fentanyl	C24H31IN2O3	522.1379	CCC(=O)N(C1CCN(CC1)CCC2=CC(=C(C=C2OC)I)OC)C3=CC=CC=C3
N-(2C-iP) Fentanyl	C27H38N2O3	438.2882	CCC(=O)N(C1CCN(CC1)CCC2=CC(=C(C=C2OC)C(C)C)OC)C3=CC=CC=C3
N-(2C-N) Fentanyl	C24H31N3O5	441.2264	CCC(=O)N(C1CCN(CC1)CCC2=CC(=C(C=C2OC)[N+](=O)[O-])OC)C3=CC=CC=C3
N-(2C-T) Fentanyl	C25H34N2O3S	442.2290	CCC(=O)N(C1CCN(CC1)CCC2=CC(=C(C=C2OC)SC)OC)C3=CC=CC=C3
N-(2C-T-2) Fentanyl	C26H36N2O3S	456.2447	CCC(=O)N(C1CCN(CC1)CCC2=CC(=C(C=C2OC)SCC)OC)C3=CC=CC=C3
N-(2C-T-4) Fentanyl	C27H38N2O3S	470.2603	CCC(=O)N(C1CCN(CC1)CCC2=CC(=C(C=C2OC)SC(C)C)OC)C3=CC=CC=C3
N-(2C-TFM) Fentanyl	C25H31F3N2O3	464.2287	CCC(=O)N(C1CCN(CC1)CCC2=CC(=C(C=C2OC)C(F)(F)F)OC)C3=CC=CC=C3
N-(2C-B-fly) Fentanyl	C26H31BrN2O3	498.1518	CCC(=O)N(C1CCN(CC1)CCC2=C3CCOC3=C(C4=C2OCC4)Br)C5=CC=CC=C5
N-(3C-B-fly) Fentanyl	C27H33BrN2O3	512.1675	CCC(=O)N(C1CCN(CC1)C(C)CC2=C3CCOC3=C(C4=C2OCC4)Br)C5=CC=CC=C5
N-(3,4,5-TMA) Fentanyl	C26H36N2O4	440.2675	CCC(=O)N(C1CCN(CC1)C(C)CC2=CC(=C(C(=C2)OC)OC)OC)C3=CC=CC=C3
N-(2,5-DMA) Fentanyl	C25H34N2O3	410.2569	CCC(=O)N(C1CCN(CC1)C(C)CC2=C(C=CC(=C2)OC)OC)C3=CC=CC=C3
N-(DOC) Fentanyl	C25H33ClN2O3	444.2180	CCC(=O)N(C1CCN(CC1)C(C)CC2=CC(=C(C=C2OC)Cl)OC)C3=CC=CC=C3
N-(DOB) Fentanyl	C25H33BrN2O3	488.1675	CCC(=O)N(C1CCN(CC1)C(C)CC2=CC(=C(C=C2OC)Br)OC)C3=CC=CC=C3
N-(DOI) Fentanyl	C25H33IN2O3	536.1536	CCC(=O)N(C1CCN(CC1)C(C)CC2=CC(=C(C=C2OC)I)OC)C3=CC=CC=C3
N-(DOM) Fentanyl	C26H36N2O3	424.2726	CCC(=O)N(C1CCN(CC1)C(C)CC2=C(C=C(C(=C2)OC)C)OC)C3=CC=CC=C3
N-(DOET) Fentanyl	C27H38N2O3	438.2882	CCC1=CC(=C(C=C1OC)CC(C)N2CCC(CC2)N(C3=CC=CC=C3)C(=O)CC)OC
N-(DOBU) Fentanyl	C29H42N2O3	466.3195	CCCCC1=CC(=C(C=C1OC)CC(C)N2CCC(CC2)N(C3=CC=CC=C3)C(=O)CC)OC
Despropionyl ortho-Fluorofentanyl	C19H23FN2	298.1845	C1CN(CCC1NC2=CC=CC=C2F)CCC3=CC=CC=C3
4-Piperidone	C5H9NO	99.0684	C1CNCCC1=O
2’,4’-Dimethoxy Fentanyl	C24H32N2O3	396.2413	CCC(=O)N(C1CCN(CC1)CCC2=C(C=C(C=C2)OC)OC)C3=CC=CC=C3
3’,4’-Dimethoxy Fentanyl	C24H32N2O3	396.2413	CCC(=O)N(C1CCN(CC1)CCC2=CC(=C(C=C2)OC)OC)C3=CC=CC=C3
2-Methyl AP-237	C18H26N2O	286.2045	CCCC(=O)N1CCN(CC1C)CC=CC2=CC=CC=C2
Tianeptine	C21H25ClN2O4S	436.1224	CN1C2=CC=CC=C2C(C3=C(S1(=O)=O)C=C(C=C3)Cl)NCCCCCCC(=O)O
Piperidylthiambutene	C17H21NS2	303.1115	CC(C=C(C1=CC=CS1)C2=CC=CS2)N3CCCCC3
Isotonitazene	C23H30N4O3	410.2318	CCN(CC)CCN1C2=C(C=C(C=C2)[N+](=O)[O-])N=C1CC3=CC=C(C=C3)OC(C)C
meta-Fluoro Valeryl Fentanyl	C24H31FN2O	382.2420	CCCCC(=O)N(C1CCN(CC1)CCC2=CC=CC=C2)C3=CC(=CC=C3)F
meta-Fluoro Acrylfentanyl	C22H25FN2O	352.1951	C=CC(=O)N(C1CCN(CC1)CCC2=CC=CC=C2)C3=CC(=CC=C3)F
Metonitazene	C21H26N4O3	382.2005	CCN(CC)CCN1C2=C(C=C(C=C2)[N+](=O)[O-])N=C1CC3=CC=C(C=C3)OC
4-Anilino-1-Benzylpiperidine	C18H22N2	266.1783	C1CN(CCC1NC2=CC=CC=C2)CC3=CC=CC=C3
4-Anilino-1-Boc-Piperidine	C16H24N2O2	276.1838	CC(C)(C)OC(=O)N1CCC(CC1)NC2=CC=CC=C2
para-Chloro Furanyl Fentanyl 3-Furancarboxamide	C24H25ClN2O2	408.1605	C1CN(CCC1N(C2=CC=C(C=C2)Cl)C(=O)C3=COC=C3)CCC4=CC=CC=C4
Thiophene Fentanyl 3-Thiophenecarboxamide	C24H26N2OS	390.1766	C1CN(CCC1N(C2=CC=CC=C2)C(=O)C3=CSC=C3)CCC4=CC=CC=C4
3’-Fluorofentanyl	C22H27FN2O	354.2107	CCC(=O)N(C1CCN(CC1)CCC2=CC(=CC=C2)F)C3=CC=CC=C3
ortho-Fluoro Valeryl Fentanyl	C24H31FN2O	382.2420	CCCCC(=O)N(C1CCN(CC1)CCC2=CC=CC=C2)C3=CC=CC=C3F
4-Methyl Fentanyl	C23H30N2O	350.2358	CCC(=O)N(C1=CC=CC=C1)C2(CCN(CC2)CCC3=CC=CC=C3)C
Cyclopropaneacetyl Fentanyl	C24H30N2O	362.2358	C1CC1CC(=O)N(C2CCN(CC2)CCC3=CC=CC=C3)C4=CC=CC=C4
Etonitazene	C22H28N4O3	396.2161	CCN(CC)CCN1C2=C(C=C(C=C2)[N+](=O)[O-])N=C1CC3=CC=C(C=C3)OCC
para-Chloroacetyl Fentanyl	C21H25ClN2O	356.1655	CC(=O)N(C1CCN(CC1)CCC2=CC=CC=C2)C3=CC=C(C=C3)Cl
2-Fluoro Viminol	C21H31FN2O	346.2420	CCC(C)N(CC(C1=CC=CN1CC2=CC=CC=C2F)O)C(C)CC
para-Hydroxy Butyryl Fentanyl	C23H30N2O2	366.2307	CCCC(=O)N(C1CCN(CC1)CCC2=CC=CC=C2)C3=CC=C(C=C3)O
2’-Fluorofentanyl	C22H27FN2O	354.2107	CCC(=O)N(C1CCN(CC1)CCC2=CC=CC=C2F)C3=CC=CC=C3

**Table S9.** Compound database imported into the *Search Mass Lists* node (continued)			
Compound Name	Molecular Formula	Exact Mass	SMILES
AP-237	C17H24N2O	272.1889	CCCC(=O)N1CCN(CC1)CC=CC2=CC=CC=C2
meta-Methoxy Furanyl Fentanyl	C25H28N2O3	404.2100	COC1=CC=CC(=C1)N(C2CCN(CC2)CCC3=CC=CC=C3)C(=O)C4=CC=CO4
Brorphine	C20H22BrN3O	399.0946	CC(C1=CC=C(C=C1)Br)N2CCC(CC2)N3C4=CC=CC=C4NC3=O
2,3-Benzodioxole Fentanyl	C27H28N2O3	428.2100	C1CN(CCC1N(C2=CC=CC=C2)C(=O)C3=C4C(=CC=C3)OCO4)CCC5=CC=CC=C5
3'-Fluoro ortho-Fluorofentanyl	C22H26F2N2O	372.2013	CCC(=O)N(C1CCN(CC1)CCC2=CC(=CC=C2)F)C3=CC=CC=C3F
Tigloyl Fentanyl	C24H30N2O	362.2358	CC=C(C)C(=O)N(C1CCN(CC1)CCC2=CC=CC=C2)C3=CC=CC=C3
2',3'-Dimethoxy Fentanyl	C24H32N2O3	396.2413	CCC(=O)N(C1CCN(CC1)CCC2=C(C(=CC=C2)OC)OC)C3=CC=CC=C3
N-Benzyl-4-Piperidone	C12H15NO	189.1154	C1CN(CCC1=O)CC2=CC=CC=C2
2',6'-Dimethoxy Fentanyl	C24H32N2O3	396.2413	CCC(=O)N(C1CCN(CC1)CCC2=C(C=CC=C2OC)OC)C3=CC=CC=C3
NPP (1-Phenethyl-4-piperidone)	C13H17NO	203.1310	C1CN(CCC1=O)CCC2=CC=CC=C2
3',5'-Dimethoxy Fentanyl	C24H32N2O3	396.2413	CCC(=O)N(C1CCN(CC1)CCC2=CC(=CC(=C2)OC)OC)C3=CC=CC=C3
AP-238	C18H26N2O	286.2045	CCC(=O)N1C(CN(CC1C)CC=CC2=CC=CC=C2)C
4-Anilinopiperidine	C11H16N2	176.1313	C1CNCCC1NC2=CC=CC=C2
Fentanyl	C22H28N2O	336.2202	CCC(=O)N(C1CCN(CC1)CCC2=CC=CC=C2)C3=CC=CC=C3
Norfentanyl	C14H20N2O	232.1576	CCC(=O)N(C1CCNCC1)C2=CC=CC=C2
Meperidine	C15H21NO2	247.1572	CCOC(=O)C1(CCN(CC1)C)C2=CC=CC=C2
Normeperidine	C14H19NO2	233.1416	CCOC(=O)C1(CCNCC1)C2=CC=CC=C2
Codeine	C18H21NO3	299.1521	CN1CCC23C4C1CC5=C2C(=C(C=C5)OC)OC3C(C=C4)O
Norcodeine	C17H19NO3	285.1365	COC1=C2C3=C(CC4C5C3(CCN4)C(O2)C(C=C5)O)C=C1
Hydrocodone	C18H21NO3	299.1521	CN1CCC23C4C1CC5=C2C(=C(C=C5)OC)OC3C(=O)CC4
Norhydrocodone	C17H19NO3	285.1365	COC1=C2C3=C(CC4C5C3(CCN4)C(O2)C(=O)CC5)C=C1
Morphine	C17H19NO3	285.1365	CN1CCC23C4C1CC5=C2C(=C(C=C5)O)OC3C(C=C4)O
Hydromorphone	C17H19NO3	285.1365	CN1CCC23C4C1CC5=C2C(=C(C=C5)O)OC3C(=O)CC4
Normorphine	C16H17NO3	271.1208	C1CNC2CC3=C4C15C2C=CC(C5OC4=C(C=C3)O)O
Morphine-3-glucuronide (Morphine-3-β-D-glucuronide)	C23H27NO9	461.1686	CN1CCC23C4C1CC5=C2C(=C(C=C5)OC6C(C(C(C(O6)C(=O)O)O)O)O)OC3C(C=C4)O
Heroin	C21H23NO5	369.1576	CC(=O)OC1C=CC2C3CC4=C5C2(C1OC5=C(C=C4)OC(=O)C)CCN3C
6-Monoacetylmorphine (6-MAM; 6-Acetylmorphine)	C19H21NO4	327.1471	CC(=O)OC1C=CC2C3CC4=C5C2(C1OC5=C(C=C4)O)CCN3C
Oxycodone	C18H21NO4	315.1471	CN1CCC23C4C(=O)CCC2(C1CC5=C3C(=C(C=C5)OC)O4)O
Noroxycodone	C17H19NO4	301.1314	COC1=C2C3=C(CC4C5(C3(CCN4)C(O2)C(=O)CC5)O)C=C1
Oxymorphone	C17H19NO4	301.1314	CN1CCC23C4C(=O)CCC2(C1CC5=C3C(=C(C=C5)O)O4)O
Noroxymorphone	C16H17NO4	287.1158	C1CC2(C3CC4=C5C2(CCN3)C(C1=O)OC5=C(C=C4)O)O
Dihydrocodeine	C18H23NO3	301.1678	CN1CCC23C4C1CC5=C2C(=C(C=C5)OC)OC3C(CC4)O
Dihydromorphine	C17H21NO3	287.1521	CN1CCC23C4C1CC5=C2C(=C(C=C5)O)OC3C(CC4)O
Desomorphine	C17H21NO2	271.1572	CN1CCC23C4C1CC5=C2C(=C(C=C5)O)OC3CCC4
Buprenorphine	C29H41NO4	467.3036	CC(C)(C)C(C)(C1CC23CCC1(C4C25CCN(C3CC6=C5C(=C(C=C6)O)O4)CC7CC7)OC)O
Norbuprenorphine	C25H35NO4	413.2566	CC(C)(C)C(C)(C1CC23CCC1(C4C25CCNC3CC6=C5C(=C(C=C6)O)O4)OC)O
(±)-Methadone	C21H27NO	309.2093	CCC(=O)C(CC(C)N(C)C)(C1=CC=CC=C1)C2=CC=CC=C2
2-Ethylidene-1,5-dimethyl-3,3-diphenylpyrrolidine (EDDP)	C20H23N	278.1903	C/C=C\1/C(CC(N1C)C)(C2=CC=CC=C2)C3=CC=CC=C3
(+)-Tramadol	C16H25NO2	263.1885	CN(C)CC1CCCCC1(C2=CC(=CC=C2)OC)O
(+)-O-Desmethyltramadol	C15H23NO2	249.1729	CN(C)CC1CCCCC1(C2=CC(=CC=C2)O)O
(+)-N-Desmethyltramadol	C15H23NO2	249.1729	CNCC1CCCCC1(C2=CC(=CC=C2)OC)O
Naloxone	C19H21NO4	327.1471	C=CCN1CCC23C4C(=O)CCC2(C1CC5=C3C(=C(C=C5)O)O4)O

Table S9. Compound database imported into the *Search Mass Lists* node (continued)

Compound Name	Molecular Formula	Exact Mass	SMILES
Diphenhydramine	C17H21NO	255.1623	CN(C)CCOC(C1=CC=CC=C1)C2=CC=CC=C2
Alprazolam	C17H13ClN4	308.0829	CC1=NN=C2N1C3=C(C=C(C=C3)Cl)C(=NC2)C4=CC=CC=C4
α-Hydroxyalprazolam	C17H13ClN4O	324.0778	C1C2=NN=C(N2C3=C(C=C(C=C3)Cl)C(=N1)C4=CC=CC=C4)CO
Diazepam	C16H13ClN2O	284.0716	CN1C(=O)CN=C(C2=C1C=CC(=C2)Cl)C3=CC=CC=C3
Nordiazepam	C15H11ClN2O	270.0560	C1C(=O)NC2=C(C=C(C=C2)Cl)C(=N1)C3=CC=CC=C3
Cocaine	C17H21NO4	303.1471	CN1C2CCC1C(C(C2)OC(=O)C3=CC=CC=C3)C(=O)OC
Benzoylecgonine	C16H19NO4	289.1314	CN1C2CCC1C(C(C2)OC(=O)C3=CC=CC=C3)C(=O)O
Ecgonine Methyl Ester	C10H17NO3	199.1208	CN1C2CCC1C(C(C2)O)C(=O)OC
(-)-Norcocaine	C16H19NO4	289.1314	COC(=O)C1C2CCC(N2)CC1OC(=O)C3=CC=CC=C3
(±)-3,4-Methylenedioxymethamphetamine ((±)-MDMA)	C11H15NO2	193.1103	CC(CC1=CC2=C(C=C1)OCO2)NC
(±)-3,4-Methylenedioxyamphetamine ((±)-MDA)	C10H13NO2	179.0946	CC(CC1=CC2=C(C=C1)OCO2)N
(±)-Methamphetamine	C10H15N	149.1204	CC(CC1=CC=CC=C1)NC
(±)-Amphetamine	C9H13N	135.1048	CC(CC1=CC=CC=C1)N
(-)-Nicotine	C10H14N2	162.1157	CN1CCCC1C2=CN=CC=C2
(-)-Cotinine	C10H12N2O	176.0950	CN1C(CCC1=O)C2=CN=CC=C2
trans-3'-Hydroxycotinine	C10H12N2O2	192.0899	CN1C(CC(C1=O)O)C2=CN=CC=C2
Δ9-Tetrahydrocannabinol (THC)	C21H30O2	314.2246	CCCCCC1=CC(=C2C3C=C(CCC3C(OC2=C1)(C)C)C)O
(±)-11-nor-9-Carboxy-Δ9-tetrahydrocannabinol (THC-COOH)	C21H28O4	344.1988	CCCCCC1=CC(=C2C3C=C(CCC3C(OC2=C1)(C)C)C(=O)O)O
(±)-11-Hydroxy-Δ9-tetrahydrocannabinol (11-OH-THC)	C21H30O3	330.2195	CCCCCC1=CC(=C2C3C=C(CCC3C(OC2=C1)(C)C)CO)O
Caffeine	C8H10N4O2	194.0804	CN1C=NC2=C1C(=O)N(C(=O)N2C)C
Paraxanthine (1,7-Dimethylxanthine)	C7H8N4O2	180.0647	CN1C=NC2=C1C(=O)N(C(=O)N2)C
Sucralose	C12H19Cl3O8	396.0146	C(C1C(C(C(C(O1)OC2(C(C(C(O2)CCl)O)O)CCl)O)O)Cl)O
Levamisole	C11H12N2S	204.0721	C1CSC2=NC(CN21)C3=CC=CC=C3
Cocaethylene	C18H23NO4	317.1627	CCOC(=O)C1C2CCC(N2C)CC1OC(=O)C3=CC=CC=C3
Ecgonine	C9H15NO3	185.1052	CN1C2CCC1C(C(C2)O)C(=O)O
Norlofentanil	C17H24N2O3	304.1787	CCC(=O)N(C1=CC=CC=C1)C2(CCNCC2C)C(=O)OC
Lofentanil	C25H32N2O3	408.2413	CCC(=O)N(C1=CC=CC=C1)C2(CCN(CC2C)CCC3=CC=CC=C3)C(=O)OC
(Iso)butyryl-F-fentanyl N-Benzyl Analogue	C22H27FN2O	354.2102	C(C1=CC=CC=C1)N1CCC(CC1)N(C(C(C)C)=O)C1=CC=C(C=C1)F
1-(1,3-Diphenylpropan-2-yl)pyrrolidine	C19H23N	265.1825	C1CCN(C1)C(CC2=CC=CC=C2)CC3=CC=CC=C3
1-(1-Phenylcyclohexyl)azepane	C18H27N	257.2138	C1(=CC=CC=C1)C1(CCCCC1)N1CCCCCC1
1-(1-Phenylcyclohexyl)azetidine	C15H21N	215.1669	C1(=CC=CC=C1)C1(CCCCC1)N1CCC1
1-(2,3,4-Trimethoxybenzyl)piperazine	C14H22N2O3	266.1625	COC1=CC=C(C(OC)=C1OC)CN2CCNCC2
1-(2,3-Dichlorophenyl)-piperazine	C10H12Cl2N2	230.0372	ClC1=C(Cl)C(N2CCNCC2)=CC=C1
1-(2,3-Dihydro-1H-inden-5-yl)-2-phenyl-2-(pyrrolidinyl-1-yl)ethan-1-one	C21H23NO	305.1774	O=C(C1=CC2=C(CCC2)C=C1)C(C3=CC=CC=C3)N4CCCC4
1-(2,5-Dimethoxybenzyl)piperazine	C13H20N2O2	236.1519	COC1=CC=C(OC)C(CN2CCNCC2)=C1
1-(2-Chloro-3-fluorophenyl)piperazine	C10H12ClFN2	214.0668	ClC1=C(C=CC=C1F)N1CCNCC1
1-(2-Methoxyphenyl)piperazine	C11H16N2O	192.1257	COC1=C(C=CC=C1)N1CCNCC1
1-(3-Methoxyphenyl)piperazine	C11H16N2O	192.1257	COC1=CC(N2CCNCC2)=CC=C1
1-(3-Methylbenzyl)piperazine	C12H18N2	190.1465	CC1=CC(CN2CCNCC2)=CC=C1
1-(4-Bromo-2,5-dimethoxyphenyl)ethanamine	C10H14BrNO2	259.0203	BrC1=CC(=C(C=C1OC)C(C)N)OC
1-(4-Fluorophenyl)-2-(piperidin-1-yl)pentan-1-one	C16H22FNO	263.1680	CCCC(N1CCCCC1)C(C2=CC=C(F)C=C2)=O
1-(4-Methylbenzyl) Piperazine	C12H18N2	190.1465	CC1=CC=C(C=C1)CN2CCNCC2

**Table S9.** Compound database imported into the *Search Mass Lists* node (continued)			
Compound Name	Molecular Formula	Exact Mass	SMILES
1-(4-Methylphenyl)-2-(piperidin-1-yl)propan-1-one	C15H21NO	231.1618	CC(N1CCCCC1)C(C2=CC=C(C)C=C2)=O
1-(5-Methyl-2-thienyl)-propan-2-amine	C8H13NS	155.0763	CC1=CC=C(S1)CC(C)N
1,3-Dimethylbutylamine	C6H15N	101.1199	CC(CC(C)N)C
1,3-Indanedione	C9H6O2	146.0362	O=C1CC(C2=C1C=CC=C2)=O
1,4-Butanediol	C4H10O2	90.0675	OCCCCO
1,4-DMAA	C7H17N	115.1356	CC(CCC(C)N)C
1-Aminoindan	C9H11N	133.0886	NC1CCC2=C1C=CC=C2
1B-LSD	C24H31N3O2	393.2411	C(CCC)(=O)N1C=C2CC3N(CC(C=C3C3=CC=CC1=C32)C(=O)N(CC)CC)C
1cP-AL-LAD	C26H31N3O2	417.2411	C1(CC1)C(=O)N1C=C2C[C@H]3N(C[C@@H](C=C3C=3C=CC=C1C32)C(=O)N(CC)CC)CC=C
1cP-LSD	C24H29N3O2	391.2254	CN1[C@](C2=C[C@@H](C(N(CC)CC)=O)C1)([H])CC3=CN(C(C4CC4)=O)C5=C3C2=CC=C5
1-Cyclohexylmethylindole	C15H19N	213.1512	N1(CC2CCCCC2)C=CC3=C1C=CC=C3
1-Cyclohexyl-x-methoxybenzene	C13H18O	190.1352	COC1=CC(C2CCCCC2)=CC=C1
1-Demethylphenazolam	C16H10BrClN4	371.9772	ClC1=CC=CC=C1C2=NCC3=NN=CN3C4=CC=C(Br)C=C24
1-Ethynyl-cyclohexanol (ECX)	C8H12O	124.0883	OC1(C#C)CCCCC1
1-Naphthoyl indole	C19H13NO	271.0992	O=C(C1=CNC2=C1C=CC=C2)C3=C4C=CC=CC4=CC=C3
1-Naphthyl U-47700	C20H26N2O	310.2040	CN(C)[C@@H]1CCCC[C@H]1N(C)C(C2=C(C=CC=C3)C3=CC=C2)=O
1-Naphthyl-CP	C21H27N	293.2138	C1(=CC=CC2=CC=CC=C12)C1(CCCCC1)N1CCCCC1
1-PEA	C8H11N	121.0886	CC(C1=CC=CC=C1)N
1-Pentylindole	C13H17N	187.1356	CCCCCN1C=CC2=C1C=CC=C2
1P-ETH-LAD	C24H31N3O2	393.2411	O=C([C@H](C=C12)CN(CC)[C@]2([H])CC3=CN(C(CC)=O)C4=C3C1=CC=C4)N(CC)CC
1-Phenethyl-4-hydroxypiperidene	C13H19NO	205.1461	OC1CCN(CCC2=CC=CC=C2)CC1
1-Phenyl-1-propanamine	C9H13N	135.1043	CCC(C1=CC=CC=C1)N
1-Phenyl-2-(piperidin-1-yl)butan-1-one	C15H21NO	231.1618	CCC(N1CCCCC1)C(C2=CC=CC=C2)=O
1-Phenylbutan-2-amine	C10H15N	149.1199	CCC(N)CC1=CC=CC=C1
1p-LSD	C23H29N3O2	379.2254	C(C)N(C(=O)[C@H]1CN([C@@H]2CC=3C4=C(C2=C1)C=CC=C4N(C3)C(CC)=O)C)CC
1V-LSD	C25H33N3O2	407.2567	C(C)N(C(=O)[C@H]1CN(C)[C@@H]2CC3=CN(C4=CC=CC(C2=C1)=C34)C(CCCC)=O)CC
2-(1H-Indol-3-yl)-N,N-dipropylacetamide	C16H22N2O	258.1727	O=C(N(CCC)CCC)CC1=CNC2=C1C=CC=C2
2-(2,3-Dimethoxyphenyl)-N-(3,4,5-trimethoxybenzyl)ethanamine	C20H27NO5	361.1884	COC1=C(OC)C(OC)=CC(CNCCC2=CC=CC(OC)=C2OC)=C1
2-(2-Chlorophenyl)-2-hydroxycyclohexanone	C12H13ClO2	224.0599	ClC1=C(C=CC=C1)C1(C(CCCC1)=O)O
2-(4-Fluorophenyl)piperazine	C10H13FN2	180.1057	FC1=CC=C(C2NCCNC2)C=C1
2-(4-Methylpiperazin-1-yl)-1-phenylpropan-1-one	C14H20N2O	232.1570	CN1CCN(CC1)C(C(=O)C1=CC=CC=C1)C
2,2-Dibromo-1-phenylhexan-1-one	C12H14Br2O	331.9406	BrC(C(=O)C1=CC=CC=C1)(CCCC)Br
2,3,4-TMA	C12H19NO3	225.1360	NC(C)CC1=CC=C(OC)C(OC)=C1OC
2,3,5-Trimethoxyamphetamine	C12H19NO3	225.1360	COC1=C(C=C(C=C1OC)OC)CC(N)C
2,3,6-Trimethoxyamphetamine	C12H19NO3	225.1360	COC1=C(C(=CC=C1OC)OC)CC(N)C
2,3-Dimethylethcathinone	C13H19NO	205.1461	CC1=C(C=CC=C1C)C(C(C)NCC)=O
2,3-DMMC	C12H17NO	191.1305	CC(NC)C(C1=CC=CC(C)=C1C)=O
2,3-MDMA	C11H15NO2	193.1097	CNC(CC1=CC=CC=2OCOC21)C
2,3-Methylenedioxy-alpha-ethylaminobutiophenone	C13H17NO3	235.1203	O1COC2=C1C=CC=C2C(C(CC)NCC)=O
2,4,5-TMMC	C13H19NO	205.1461	CC(NC)C(C1=CC(C)=C(C)C=C1C)=O
2,4-Difluoro U-48800	C17H24F2N2O	310.1851	FC1=C(C=CC(=C1)F)CC(=O)N(C)C1C(CCCC1)N(C)C
2,4-DMA	C11H17NO2	195.1254	CC(N)CC1=CC=C(OC)C=C1OC

Table S9. Compound database imported into the *Search Mass Lists* node (continued)

Compound Name	Molecular Formula	Exact Mass	SMILES
2,4-DMEC	C13H19NO	205.1461	CC(NCC)C(C1=CC=C(C)C=C1C)=O
2,4-DMMC	C12H17NO	191.1305	CC(NC)C(C1=CC=C(C)C=C1C)=O
2,4-DMPPP	C15H21NO	231.1618	CC(N1CCCC1)C(C2=CC=C(C)C=C2C)=O
2,5-Dimethoxy-4-ethylamphetamine	C13H21NO2	223.1567	CC(N)CC1=CC(OC)=C(CC)C=C1OC
2,5-Dimethyl-alpha-PVP	C17H25NO	259.1931	CC1=C(C=C(C=C1)C)C(C(CCC)N1CCCC1)=O
2,5-DMA	C11H17NO2	195.1254	CC(N)CC1=CC(OC)=CC=C1OC
2,5-DMMA	C12H19NO2	209.1410	COC1=C(C=C(C=C1)OC)CC(NC)C
2-[4-(2-Methoxyphenyl)piperazin-1-yl]-1,2-diphenylethanol	C25H28N2O2	388.2145	COC1=C(C=CC=C1)N1CCN(CC1)C(C(O)C1=CC=CC=C1)C1=CC=CC=C1
23b-PVP	C17H23NO2	273.1723	O1CCC2=C1C=C(C=C2)C(C(CCC)N2CCCC2)=O
25B-N(BOMe)2	C26H30BrNO4	499.1353	COC1=CC=CC=C1CN(CC2=CC=CC=C2OC)CCC3=CC(OC)=C(Br)C=C3OC
25B-NBF	C17H19BrFNO2	367.0578	FC1=CC=CC=C1CNCCC2=CC(OC)=C(Br)C=C2OC
25B-NBOH	C17H20BrNO3	365.0621	OC1=CC=CC=C1CNCCC2=CC(OC)=C(Br)C=C2OC
25B-NBOMe	C18H22BrNO3	379.0778	BrC1=CC(=C(C=C1OC)CCNCC1=C(C=CC=C1)OC)OC
25C-NB3OMe	C18H22ClNO3	335.1283	COC1=CC(CNCCC2=CC(OC)=C(Cl)C=C2OC)=CC=C1
25C-NBF	C17H19ClFNO2	323.1083	FC1=CC=CC=C1CNCCC2=CC(OC)=C(Cl)C=C2OC
25C-NBOH	C17H20ClNO3	321.1126	OC1=CC=CC=C1CNCCC2=CC(OC)=C(Cl)C=C2OC
25C-NBOMe	C18H22ClNO3	335.1283	ClC1=CC(=C(C=C1OC)CCNCC1=C(C=CC=C1)OC)OC
25CN-NBOH	C18H20N2O3	312.1468	N#CC1=CC(OC)=C(CCNCC2=CC=CC=C2O)C=C1OC
25CN-NBOMe	C19H22N2O3	326.1625	COC1=C(C#N)C=C(C(=C1)CCNCC1=C(C=CC=C1)OC)OC
25D-NBOMe	C19H25NO3	315.1829	COC1=C(C=C(C(=C1)C)OC)CCNCC1=C(C=CC=C1)OC
25E-NBOH	C19H25NO3	315.1829	C(C)C1=CC(=C(C=C1OC)CCNCC1=C(C=CC=C1)O)OC
25E-NBOMe	C20H27NO3	329.1986	COC1=CC=CC=C1CNCCC2=CC(OC)=C(CC)C=C2OC
25G-NBOMe	C20H27NO3	329.1986	COC1=CC=CC=C1CNCCC2=CC(OC)=C(C)C(C)=C2OC
25H-NB4OMe	C18H23NO3	301.1673	COC1=CC=C(CNCCC2=CC(OC)=CC=C2OC)C=C1
25H-NBMD	C18H21NO4	315.1465	COC1=CC=C(OC)C(CCNCC2=C3OCOC3=CC=C2)=C1
25H-NBOH	C17H21NO3	287.1516	COC1=CC=C(OC)C(CCNCC2=CC=CC=C2O)=C1
25H-NBOMe	C18H23NO3	301.1673	COC1=C(C=C(C=C1)OC)CCNCC1=C(C=CC=C1)OC
25I-NB34MD	C18H20INO4	441.0432	COC1=C(I)C=C(OC)C(CCNCC2=CC=C(OCO3)C3=C2)=C1
25I-NB4OMe	C18H22INO3	427.0639	COC1=C(I)C=C(OC)C(CCNCC2=CC=C(OC)C=C2)=C1
25I-NBF	C17H19FINO2	415.0439	COC1=C(I)C=C(OC)C(CCNCC2=CC=CC=C2F)=C1
25I-NBMD	C18H20INO4	441.0432	COC1=C(I)C=C(OC)C(CCNCC2=C3OCOC3=CC=C2)=C1
25I-NBOH	C17H20INO3	413.0482	IC1=CC(=C(C=C1OC)CCNCC1=C(C=CC=C1)O)OC
25I-NBOMe	C18H22INO3	427.0639	IC1=CC(=C(C=C1OC)CCNCC1=C(C=CC=C1)OC)OC
25I-NBOMe-M (2-Desmethyl-)	C17H20INO3	413.0482	OC1=CC(I)=C(OC)C=C1CCNCC2=CC=CC=C2OC
25I-NBOMe-M (5-Desmethyl-)	C17H20INO3	413.0482	OC1=CC(CCNCC2=CC=CC=C2OC)=C(OC)C=C1I
25I-NBOMe-M (para-HO)	C18H22INO4	443.0588	OC1=CC=C(CNCCC2=CC(OC)=C(I)C=C2OC)C(OC)=C1
25IP-NBOMe	C21H29NO3	343.2142	COC1=C(C(C)C)C=C(OC)C(CCNCC2=CC=CC=C2OC)=C1
25N-NBOMe	C18H22N2O5	346.1523	COC1=CC=CC=C1CNCCC2=CC(OC)=C([N+]([O-])=O)C=C2OC
25P-NBOMe	C21H29NO3	343.2142	COC1=CC=CC=C1CNCCC2=CC(OC)=C(CCC)C=C2OC
25T2-NBOMe	C20H27NO3S	361.1706	COC1=CC=CC=C1CNCCC2=CC(OC)=C(SCC)C=C2OC
25T4-NBOMe	C21H29NO3S	375.1863	COC1=CC=CC=C1CNCCC2=CC(OC)=C(SC(C)C)C=C2OC
25T7-NBOMe	C21H29NO3S	375.1863	COC1=CC=CC=C1CNCCC2=CC(OC)=C(SCCC)C=C2OC

**Table S9.** Compound database imported into the *Search Mass Lists* node (continued)			
Compound Name	Molecular Formula	Exact Mass	SMILES
25T-NBOMe	C19H25NO3S	347.1550	CSC1=CC(=C(C=C1OC)CCNCC1=C(C=CC=C1)OC)OC
2-AI	C9H11N	133.0886	NC1CC2=C(C=CC=C2)C1
2-Amino-3-(2-chlorobenzoyl)-5-ethylthiophene	C13H12ClNOS	265.0323	NC=1SC(=CC1C(=O)C1=C(C=CC=C1)Cl)CC
2-APB	C11H13NO	175.0992	CC(N)CC(O1)=CC2=C1C=CC=C2
2-Benzylamino-1-(3,4-methylenedioxyphenyl)butan-1-one	C18H19NO3	297.1360	CCC(NCC1=CC=CC=C1)C(C2=CC=C(OCO3)C3=C2)=O
2-Bromo LSD	C20H24BrN3O	401.1097	O=C(N(CC)CC)[C@H]1CN(C)[C@](C2=C1)([H])CC3=C(Br)NC4=C3C2=CC=C4
2-Bromo-4,5-dimethoxyphenethylamine	C10H14BrNO2	259.0203	BrC1=C(CCN)C=C(C(=C1)OC)OC
2-Bromoamphetamine	C9H12BrN	213.0148	NC(C)CC1=CC=CC=C1Br
2-Bromo-deschloroketamine	C13H16BrNO	281.0410	BrC1=C(C=CC=C1)C1(C(CCCC1)=O)NC
2-Bromomethamphetamine	C10H14BrN	227.0304	CC(NC)CC1=CC=CC=C1Br
2C-B	C10H14BrNO2	259.0203	BrC1=CC(=C(C=C1OC)CCN)OC
2C-B Aminorex	C11H13BrN2O3	300.0104	NC1=NCC(C2=CC(OC)=C(Br)C=C2OC)O1
2C-B-BZP	C13H19BrN2O2	314.0625	COC1=C(Br)C=C(OC)C(CN2CCNCC2)=C1
2C-B-FLY	C12H14BrNO2	283.0203	BrC1=C2C(=C(C=3CCOC31)CCN)OCC2
2C-B-FLY-NBOMe	C20H22BrNO3	403.0778	BrC1=C(OCC2)C2=C(CCNCC3=CC=CC=C3OC)C4=C1CCO4
2C-C	C10H14ClNO2	215.0708	NCCC1=CC(OC)=C(Cl)C=C1OC
2C-D	C11H17NO2	195.1254	NCCC1=CC(OC)=C(C)C=C1OC
2-CDMC	C11H14ClNO	211.0759	ClC1=C(C=CC=C1)C(C(C)N(C)C)=O
2C-E	C12H19NO2	209.1410	C(C)C1=CC(=C(C=C1OC)CCN)OC
2-CEC	C11H14ClNO	211.0759	CC(NCC)C(C1=CC=CC=C1Cl)=O
2C-E-FLY	C14H19NO2	233.1410	NCCC1=C2C(OCC2)=C(CC)C3=C1OCC3
2C-F	C10H14FNO2	199.1003	NCCC1=CC(OC)=C(F)C=C1OC
2C-G	C12H19NO2	209.1410	NCCC1=CC(OC)=C(C)C(C)=C1OC
2C-G-4	C14H21NO2	235.1567	COC1=C2CCCCC2=C(C(=C1)CCN)OC
2C-G-N	C14H17NO2	231.1254	COC1=CC(=C(C2=CC=CC=C21)OC)CCN
2C-H	C10H15NO2	181.1097	COC1=C(C=C(C=C1)OC)CCN
2-Chloro-4,5-MDMA	C11H14ClNO2	227.0708	CC(NC)CC1=C(Cl)C=C(OCO2)C2=C1
2-Chloro-MDA	C10H12ClNO2	213.0551	ClC1=C(CC(N)C)C=C2C(=C1)OCO2
2C-I	C10H14INO2	307.0064	COC1=C(I)C=C(OC)C(CCN)=C1
2C-iP	C13H21NO2	223.1567	NCCC1=CC(OC)=C(C(C)C)C=C1OC
2-Cl-2'-Oxo-PCP	C17H22ClNO	291.1385	ClC1=C(C=CC=C1)C1(C(CCCC1)=O)N1CCCCC1
2-Cl-PCP	C17H24ClN	277.1592	ClC1=C(C=CC=C1)C1(CCCCC1)N1CCCCC1
2-CMC	C10H12ClNO	197.0602	ClC1=C(C=CC=C1)C(C(C)NC)=O
2C-N	C10H14N2O4	226.0948	NCCC1=CC(OC)=C([N+]([O-])=O)C=C1OC
2C-O-4	C13H21NO3	239.1516	CC(C)OC1=C(C=C(C=C1OC)CCN)OC
2C-P	C13H21NO2	223.1567	COC1=C(CCN)C=C(OC)C(CCC)=C1
2C-T	C11H17NO2S	227.0975	CSC1=C(OC)C=C(CCN)C(OC)=C1
2C-T-2	C12H19NO2S	241.1131	C(C)SC1=CC(=C(C=C1OC)CCN)OC
2C-T-21	C12H18FNO2S	259.1037	FCCSC1=C(OC)C=C(CCN)C(OC)=C1
2C-T-4	C13H21NO2S	255.1288	COC1=C(C=C(C(=C1)SC(C)C)OC)CCN
2C-T-7	C13H21NO2S	255.1288	COC1=C(C=C(C(=C1)SCCC)OC)CCN
2C-TFM	C11H14F3NO2	249.0971	NCCC1=CC(OC)=C(C(F)(F)F)C=C1OC

**Table S9.** Compound database imported into the *Search Mass Lists* node (continued)			
Compound Name	Molecular Formula	Exact Mass	SMILES
2-Diphenylmethylpyrrolidine	C17H19N	237.1512	C1(C(C2=CC=CC=C2)C3=CC=CC=C3)NCCC1
2-EAPB	C13H17NO	203.1305	CC(NCC)CC(O1)=CC2=C1C=CC=C2
2-Ethylethcathinone	C13H19NO	205.1461	C(C)NC(C(=O)C1=C(C=CC=C1)CC)C
2'-Et-PCP	C19H29N	271.2295	C(C)C1C(CCCC1)(C1=CC=CC=C1)N1CCCCC1
2-FEC	C11H14FNO	195.1054	C(C)NC(C(=O)C1=C(C=CC=C1)F)C
2-Fluoro ADB	C20H28FN3O3	377.2109	CC(C)(C)[C@H](NC(C1=NN(CC(F)CCC)C2=C1C=CC=C2)=O)C(OC)=O
2-Fluoro AMB	C19H26FN3O3	363.1953	CC(C)[C@@H](C(OC)=O)NC(C1=NN(CC(F)CCC)C2=C1C=CC=C2)=O
2-Fluoro Deschloronorketamine	C12H14FNO	207.1054	NC1(C(CCCC1)=O)C1=C(C=CC=C1)F
2-Fluoro NNEI	C24H23FN2O	374.1789	FC(CN1C=C(C2=CC=CC=C12)C(=O)NC1=CC=CC2=CC=CC=C12)CCC
2'-Fluoro-2-oxo PCE	C14H18FNO	235.1367	O=C1CCCCC1(NCC)C2=CC=CC=C2F
2-Fluoroacetyl Fentanyl	C21H25FN2O	340.1946	FC1=C(C=CC=C1)N(C(C)=O)C1CCN(CC1)CCC1=CC=CC=C1
2-Fluoroamphetamine	C9H12FN	153.0948	CC(N)CC1=CC=CC=C1F
2-Fluoroethamphetamine	C11H16FN	181.1261	C(C)NC(CC1=C(C=CC=C1)F)C
2-Fluorofentanyl	C22H27FN2O	354.2102	FC1N(CCC(C1)N(C(CC)=O)C1=CC=CC=C1)CCC1=CC=CC=C1
2-Fluoro-JWH-019	C25H24FNO	373.1837	FC(CN1C=C(C2=CC=CC=C12)C(=O)C1=CC=CC2=CC=CC=C12)CCCC
2-Fluoromethamphetamine	C10H14FN	167.1105	CC(NC)CC1=CC=CC=C1F
2-Fluoropentylindole	C13H16FN	205.1261	FC(CN1C=CC2=CC=CC=C12)CCC
2-Fluoroviminol	C21H31FN2O	346.2415	OC(C1=CC=CN1CC2=CC=CC=C2F)CN(C(CC)C)C(CC)C
2-FMC	C10H12FNO	181.0898	FC1=C(C=CC=C1)C(C(C)NC)=O
2F-PBCHA	C13H16FN	205.1261	FC1=C(C=CC=C1)C1(C2CCC(C1)C2)N
2F-PBCHPy	C17H22FN	259.1731	FC1=C(C=CC=C1)C1(C2CCC(C1)C2)N2CCCC2
2F-Phenmetrazine	C11H14FNO	195.1054	CC1NCCOC1C2=CC=CC=C2F
2-FPP	C10H13FN2	180.1057	FC1=CC=CC=C1N2CCNCC2
2-FPPP	C18H20FN	269.1574	FC1=CC=CC=C1C(N2CCCC2)CC3=CC=CC=C3
2-HO-PCP	C17H25NO	259.1931	N1(CCCCC1)C1(CCCCC1)C1=C(C=CC=C1)O
2-Hydroxyethylflurazepam	C17H14ClFN2O2	332.0722	ClC=1C=CC2=C(C(=NCC(N2CCO)=O)C2=C(C=CC=C2)F)C1
2-MABB	C13H17NO	203.1305	CCC(NC)CC(O1)=CC2=C1C=CC=C2
2-MAPB	C12H15NO	189.1148	CC(NC)CC(O1)=CC2=C1C=CC=C2
2-MEC	C12H17NO	191.1305	CC(NCC)C(C1=CC=CC=C1C)=O
2-MeO-Ketamine	C14H19NO2	233.1410	O=C1C(NC)(C2=CC=CC=C2OC)CCCC1
2-MeO-PCMo	C17H25NO2	275.1880	COC1=C(C=CC=C1)C1(CCCCC1)N1CCOCC1
2'-MeO-PCP	C18H27NO	273.2087	COC1C(CCCC1)(C1=CC=CC=C1)N1CCCCC1
2-MeO-PCP	C18H27NO	273.2087	COC1=C(C=CC=C1)C1(CCCCC1)N1CCCCC1
2-Me-PCE	C15H23N	217.1825	C(C)NC1(CCCCC1)C1=C(C=CC=C1)C
2-Me-PCP	C18H27N	257.2138	CC1=C(C=CC=C1)C1(CCCCC1)N1CCCCC1
2'-Me-PVP	C16H23NO	245.1774	CC1=C(C=CC=C1)C(C(CCC)N1CCCC1)=O
2-Methoxyamphetamine	C10H15NO	165.1148	CC(N)CC1=CC=CC=C1OC
2-Methyl Carfentanil	C25H32N2O3	408.2407	CC1=C(C=CC=C1)N(C1(CCN(CC1)CCC1=CC=CC=C1)C(=O)OC)C(CC)=O
2-Methyl DMT	C13H18N2	202.1465	CC(N1)=C(CCN(C)C)C2=C1C=CC=C2
2-Methyl-α-PiHP	C17H25NO	259.1931	CC(CC(C(=O)C1=C(C=CC=C1)C)N1CCCC1)C
2-Methylamphetamine	C10H15N	149.1199	CC(N)CC1=CC=CC=C1C
2-Methylethylbuphedrone	C13H19NO	205.1461	C(C)NC(C(=O)C1=C(C=CC=C1)C)CC

**Table S9.** Compound database imported into the *Search Mass Lists* node (continued)			
Compound Name	Molecular Formula	Exact Mass	SMILES
2-Methylfentanyl	C23H30N2O	350.2353	CC1N(CCC(C1)N(C(CC)=O)C1=CC=CC=C1)CCC1=CC=CC=C1
2-MMC	C11H15NO	177.1148	CNC(C(=O)C1=C(C=CC=C1)C)C
2-Naphthyl U-47700	C20H26N2O	310.2040	CN([C@H]1[C@@H](CCCC1)N(C(=O)C1=CC2=CC=CC=C2C=C1)C)C
2-NMC	C12H17NO	191.1305	CN(C(C(C)C)=O)C1=CC=C(C=C1)C
2-Oxo-3-hydroxy-LSD	C20H25N3O3	355.1890	C(C)N(C(=O)[C@H]1CN([C@@H]2CC3(C4=C(C2=C1)C=CC=C4NC3=O)O)C)CC
2’-Oxo-PCP	C17H23NO	257.1774	C1(=CC=CC=C1)C1(C(CCCC1)=O)N1CCCCC1
2’-Oxo-PCPr	C15H21NO	231.1618	C1(=CC=CC=C1)C1(C(CCCC1)=O)NCCC
2PYCP	C16H24N2	244.1934	N1(CCCCC1)C1(CCCCC1)C1=NC=CC=C1
3-(2,3-Dimethylphenyl)-2-methyl-4-(3H)-quinazolinone	C17H16N2O	264.1257	CC1=C(C=CC=C1C)N1C(=NC2=CC=CC=C2C1=O)C
3-(4-Hydroxymethylbenzoyl)-1-pentylindole	C21H23NO2	321.1723	O=C(C1=CC=C(CO)C=C1)C2=CN(CCCCC)C3=C2C=CC=C3
3-(p-Methoxybenzoyl)-N-methylindole	C17H15NO2	265.1097	O=C(C1=CC=C(OC)C=C1)C2=CN(C)C3=C2C=CC=C3
3’,3’,5’-TriMe-PCP	C20H31N	285.2451	CC1(CC(CC(C1)C)(C1=CC=CC=C1)N1CCCCC1)C
3,3-Dimethylfentanyl	C24H32N2O	364.2509	CC1(CN(CCC1N(C(CC)=O)C1=CC=CC=C1)CCC1=CC=CC=C1)C
3,4,5-Trimethoxyamphetamine	C12H19NO3	225.1360	CC(N)CC1=CC(OC)=C(OC)C(OC)=C1
3,4-CFP	C10H12ClFN2	214.0668	FC1=CC=C(N2CCNCC2)C=C1Cl
3,4-Dichloro-alpha-pyrrolidinoisohexanophenone	C16H21Cl2NO	313.0995	ClC=1C=C(C=CC1Cl)C(C(CC(C)C)N1CCCC1)=O
3,4-Dichloromethylphenidate	C14H17Cl2NO2	301.0631	ClC=1C=C(C=CC1Cl)C(C(=O)OC)C1NCCCC1
3,4-Dichloro-N,N-dimethcathinone	C11H13Cl2NO	245.0369	ClC=1C=C(C=CC1Cl)C(C(C)N(C)C)=O
3,4-Dichloro-N,N-cyclohexylmethylmethcathinone	C16H21Cl2NO	313.0995	CC(N(C1CCCCC1)C)C(C2=CC=C(Cl)C(Cl)=C2)=O
3,4-Difluoro Isopropyl U-47700	C18H26F2N2O	324.2008	FC=1C=C(C(=O)N(C(C)C)[C@H]2[C@@H](CCCC2)N(C)C)C=CC1F
3,4-Difluoro Propyl U-47700	C18H26F2N2O	324.2008	CN(C1C(CCCC1)N(C(C1=CC(=C(C=C1)F)F)=O)CCC)C
3,4-Difluoro U-49900	C18H26F2N2O	324.2008	C(C)N([C@H]1[C@@H](CCCC1)N(C(C1=CC(=C(C=C1)F)F)=O)C)CC
3,4-Difluoro U-50488	C19H26F2N2O	336.2008	FC=1C=C(C=CC1F)CC(=O)N([C@H]1[C@@H](CCCC1)N1CCCC1)C
3,4-Difluoro-N,N-didesmethyl U-47700	C14H18F2N2O	268.1382	N[C@H]1[C@@H](CCCC1)N(C(C1=CC(=C(C=C1)F)F)=O)C
3,4-Difluoro-U-47700	C16H22F2N2O	296.1695	CN(C1C(CCCC1)N(C(C1=CC(=C(C=C1)F)F)=O)C)C
3,4-Dihydroxyamphetamine	C9H13NO2	167.0941	NC(CC=1C=C(C(=CC1)O)O)C
3,4-Dimethoxy-α-PHP	C18H27NO3	305.1986	CCCCC(N1CCCC1)C(C2=CC=C(OC)C(OC)=C2)=O
3,4-Dimethoxy-α-PVP	C17H25NO3	291.1829	CCCC(N1CCCC1)C(C2=CC=C(OC)C(OC)=C2)=O
3,4-Dimethyl Alpha-PVP	C17H25NO	259.1931	CC=1C=C(C=CC1C)C(C(CCC)N1CCCC1)=O
3,4-Dimethylethcathinone (3,4-DMEC)	C13H19NO	205.1461	CC(NCC)C(C1=CC=C(C)C(C)=C1)=O
3,4-Dimethylmethcathinone Norepinephrine Metabolite	C11H17NO	179.1305	CC(N)C(C1=CC=C(C)C(C)=C1)O
3,4-DMA	C11H17NO2	195.1254	CC(N)CC1=CC=C(OC)C(OC)=C1
3,4-DMA NBOMe	C19H25NO3	315.1829	CC(NCC1=CC=CC=C1OC)CC2=CC=C(OC)C(OC)=C2
3,4-DMAR	C11H14N2O	190.1101	N=C1OC(C2=CC=CC=C2)C(C)N1C
3,4-DMMC	C12H17NO	191.1305	CC(NC)C(C1=CC=C(C)C(C)=C1)=O
3,4-EDMA	C12H17NO2	207.1254	O1CCOC2=C1C=C(C=C2)CC(C)NC
3,4-EDMC	C12H15NO3	221.1047	O1CCOC2=C1C=CC(=C2)C(C(C)NC)=O
3,4-Ethylenedioxy U-47700	C18H26N2O3	318.1938	CN(C1C(CCCC1)N(C(=O)C1=CC2=C(OCCO2)C=C1)C)C
3,4-Ethylenedioxy U-51754	C19H28N2O3	332.2094	O1CCOC2=C1C=CC(=C2)CC(=O)N(C)C2C(CCCC2)N(C)C
3,4-HHMA	C10H15NO2	181.1097	OC1=CC=C(CC(NC)C)C=C1O
3,4-MDMA Methylene Homologue	C12H17NO2	207.1254	CNCC(CC1=CC2=C(OCO2)C=C1)C
3,4-MDPA	C13H19NO2	221.1410	CCCNC(C)CC1=CC=C(OCO2)C2=C1

**Table S9.** Compound database imported into the *Search Mass Lists* node (continued)			
Compound Name	Molecular Formula	Exact Mass	SMILES
3,4-MD-PCP	C18H25NO2	287.1880	O1COC2=C1C=CC(=C2)C2(CCCCC2)N2CCCCC2
3’,4’-Methylenedioxy alpha-Methyl Fentanyl	C24H30N2O3	394.2251	O1COC2=C1C=CC(=C2)CC(C)N2CCC(CC2)N(C(CC)=O)C2=CC=CC=C2
3,4-Methylenedioxy U-47700	C17H24N2O3	304.1781	CN(C1C(CCCC1)N(C(=O)C1=CC2=C(OCO2)C=C1)C)C
3,4-Methylenedioxy-α-butylaminopropiophenone	C14H19NO3	249.1360	O1COC2=C1C=CC(=C2)C(C(C)NCCCC)=O
3,4-Methylenedioxy-5-methylethcathinone	C13H17NO3	235.1203	C(C)NC(C(=O)C1=CC2=C(OCO2)C(=C1)C)C
3,4-Methylenedioxy-alpha-isopropylaminobutiophenone	C14H19NO3	249.1360	O1COC2=C1C=CC(=C2)C(C(CC)NC(C)C)=O
3,4-Methylenedioxy-alpha-methylPPP	C15H19NO3	261.1360	CC(N1CCCC1)(C)C(C2=CC=C(OCO3)C3=C2)=O
3,4-Methylenedioxy-PV9	C19H27NO3	317.1986	CCCCCCC(N1CCCC1)C(C2=CC=C(OCO3)C3=C2)=O
3,4-Pr-PipVP	C19H27NO	285.2087	C1CCC2=CC(=CC=C12)C(C(CCC)N1CCCCC1)=O
3,4-Trimethylene-alpha-PVP	C18H25NO	271.1931	CCCC(N1CCCC1)C(C2=CC3=C(CCC3)C=C2)=O
3,4-Trimethylenepentedrone	C15H21NO	231.1618	CCCC(NC)C(C1=CC2=C(CCC2)C=C1)=O
3,5-ADB-4en-PFUPPYCA	C21H27FN4O2	386.2113	NC(C(C(C)(C)C)NC(=O)C1=CC(=NN1CCCC=C)C1=CC=C(C=C1)F)=O
3,5-Dimethyl-cyclopentylfentanyl	C27H36N2O	404.2822	CC1CN(CC(C1N(C(=O)C1CCCC1)C1=CC=CC=C1)C)CCC1=CC=CC=C1
3,5-Dimethylfentanyl	C24H32N2O	364.2509	CC1CN(CC(C1N(C(CC)=O)C1=CC=CC=C1)C)CCC1=CC=CC=C1
3,6-DMPM	C12H17NO	191.1305	CC1OC(C2=CC=CC=C2)C(C)NC1
30C-NBOMe	C20H26ClNO5	395.1494	COC1=C(OC)C(OC)=CC(CNCCC2=CC(OC)=C(Cl)C=C2OC)=C1
3’-4’-Dichloro-3”-fluorofentanyl	C22H25Cl2FN2O	422.1323	ClC=1C=C(C=CC1Cl)N(C(CC)=O)C1CCN(CC1)CCC1=CC(=CC=C1)F
3-4-MD-α-PBP	C16H21NO3	275.1516	O1COC2=C1C=CC(=C2)C(CC(CC)N2CCCC2)=O
3’-4’-Methylenedioxyfentanyl	C23H28N2O3	380.2094	O1COC2=C1C=CC(=C2)N(C(CC)=O)C2CCN(CC2)CCC2=CC=CC=C2
3-AcO-PCP	C19H27NO2	301.2036	C(C)(=O)OC1=CC(=CC=C1)C1(CCCCC1)N1CCCCC1
3-Allylfentanyl	C25H32N2O	376.2509	CCC(N([C@H]1[C@@H](CC=C)CN(CCC2=CC=CC=C2)CC1)C3=CC=CC=C3)=O
3-Amino-1-phenyl-butane	C10H15N	149.1199	CC(N)CCC1=CC=CC=C1
3-Anilinopiperidine	C11H16N2	176.1308	C1(=CC=CC=C1)NC1CNCCC1
3-Bromoamphetamine	C9H12BrN	213.0148	NC(C)CC1=CC=CC(Br)=C1
3-Bromomethamphetamine	C10H14BrN	227.0304	CC(NC)CC1=CC=CC(Br)=C1
3-Bromomethcathinone	C10H12BrNO	241.0097	CC(NC)C(C1=CC=CC(Br)=C1)=O
3-Br-PCP	C17H24BrN	321.1087	BrC=1C=C(C=CC1)C1(CCCCC1)N1CCCCC1
3-CAF	C24H15FN2O2	382.1112	O=C(C1=NN(C2=CC=CC=C2F)C3=C1C=CC=C3)OC4=CC=C5C=CC=CC5=C4
3C-B-fly	C13H16BrNO2	297.0359	BrC1=C2OCCC2=C(C=2OCCC21)CC(N)C
3-CDMC	C11H14ClNO	211.0759	ClC=1C=C(C=CC1)C(C(C)N(C)C)=O
3C-E	C13H21NO3	239.1516	C(C)OC1=C(C=C(C=C1OC)CC(C)N)OC
3-CF3O-PCP	C18H24F3NO	327.1805	FC(OC=1C=C(C=CC1)C1(CCCCC1)N1CCCCC1)(F)F
3-Chlorocathinone	C9H10ClNO	183.0446	NC(C(=O)C1=CC(=CC=C1)Cl)C
3-Chloroethcathinone	C11H14ClNO	211.0759	CC(NCC)C(C1=CC=CC(Cl)=C1)=O
3-Chloromethcathinone	C10H12ClNO	197.0602	ClC=1C=C(C=CC1)C(C(C)NC)=O
3-Cl-PCE	C14H20ClN	237.1279	ClC=1C=C(C=CC1)C1(CCCCC1)NCC
3C-P	C14H23NO3	253.1673	COC=1C=C(C=C(C1OCCC)OC)CC(C)N
3-CPM	C11H14ClNO	211.0759	ClC=1C=C(C=CC1)C1C(NCCO1)C
3-EMC	C12H17NO	191.1305	CCC1=CC(=CC=C1)C(=O)C(C)NC
3-Ethoxy-4-methoxy N,N-Diethylhexedrone	C19H31NO3	321.2299	C(C)N(C(C(=O)C1=CC(=C(C=C1)OC)OCC)CCCC)CC
3-Ethoxy-4-methoxy N,N-Diethylpentedrone	C18H29NO3	307.2142	C(C)N(C(C(=O)C1=CC(=C(C=C1)OC)OCC)CCC)CC
3-Ethylethcathinone	C13H19NO	205.1461	C(C)NC(C(=O)C1=CC(=CC=C1)CC)C

**Table S9.** Compound database imported into the *Search Mass Lists* node (continued)			
Compound Name	Molecular Formula	Exact Mass	SMILES
3-Ethylfentanyl	C24H32N2O	364.2509	C(C)C1CN(CCC1N(C(CC)=O)C1=CC=CC=C1)CCC1=CC=CC=C1
3-EtO-PCA	C14H21NO	219.1618	C(C)OC=1C=C(C=CC1)C1(CCCCC1)N
3-EtO-PCP	C19H29NO	287.2244	C(C)OC=1C=C(C=CC1)C1(CCCCC1)N1CCCCC1
3F-4-ANBP	C18H21FN2	284.1683	FC1=CC(NC2CCN(CC3=CC=CC=C3)CC2)=CC=C1
3-FA	C9H12FN	153.0948	FC=1C=C(C=CC1)CC(N)C
3F-alpha-PHP	C16H22FNO	263.1680	FC=1C=C(C=CC1)C(C(CCCC)N1CCCC1)=O
3F-alpha-PiHP	C16H22FNO	263.1680	FC=1C=C(C=CC1)C(C(CC(C)C)N1CCCC1)=O
3F-alpha-PVP	C15H20FNO	249.1524	CCCC(N1CCCC1)C(C2=CC=CC(F)=C2)=O
3-FEA	C11H16FN	181.1261	C(C)NC(CC1=CC(=CC=C1)F)C
3-FEC	C11H14FNO	195.1054	C(C)NC(C(=O)C1=CC(=CC=C1)F)C
3-Fluoro ADB	C20H28FN3O3	377.2109	CC(C)(C)[C@H](NC(C1=NN(CCC(F)CC)C2=C1C=CC=C2)=O)C(OC)=O
3-Fluoro AMB	C19H26FN3O3	363.1953	CCC(F)CCn1nc(C(=O)NC(C(=O)OC)C(C)C)c2ccccc21
3-Fluoro Deschloroketamine	C13H16FNO	221.1211	FC=1C=C(C=CC1)C1(C(CCCC1)=O)NC
3-Fluoro NNEI	C24H23FN2O	374.1789	O=C(C1=CN(CCC(F)CC)C2=C1C=CC=C2)NC3=C4C=CC=CC4=CC=C3
3-Fluoro-4-methyl-alpha-pyrrolidinovalerophenone	C16H22FNO	263.1680	FC=1C=C(C=CC1C)C(C(CCC)N1CCCC1)=O
3-Fluoroacetyl Fentanyl	C21H25FN2O	340.1946	FC=1C=C(C=CC1)N(C(C)=O)C1CCN(CC1)CCC1=CC=CC=C1
3-Fluoro-isomethcathinone	C10H12FNO	181.0898	CC(C(C1=CC=CC(F)=C1)NC)=O
3-Fluorophenmetrazine	C11H14FNO	195.1054	FC=1C=C(C=CC1)C1C(NCCO1)C
3-Fluropentylindole	C13H16FN	205.1261	CCC(F)CCn1ccc2ccccc21
3-FMA	C10H14FN	167.1105	FC=1C=C(C=CC1)CC(C)NC
3-FMC	C10H12FNO	181.0898	FC=1C=C(C=CC1)C(C(C)NC)=O
3F-MT-45	C24H31FN2	366.2466	FC1=CC(C(N2CCN(C3CCCCC3)CC2)CC4=CC=CC=C4)=CC=C1
3F-NEB	C12H16FNO	209.1211	C(C)NC(C(=O)C1=CC(=CC=C1)F)CC
3F-N-Ethylhexedrone	C14H20FNO	237.1524	C(C)NC(C(=O)C1=CC(=CC=C1)F)CCCC
3F-PBCHA	C13H16FN	205.1261	FC=1C=C(C=CC1)C1(C2CCC(C1)C2)N
3F-PBCHP	C18H24FN	273.1887	FC=1C=C(C=CC1)C1(C2CCC(C1)C2)N2CCCCC2
3F-PBCHPy	C17H22FN	259.1731	FC=1C=C(C=CC1)C1(C2CCC(C1)C2)N2CCCC2
3-F-PCA	C12H16FN	193.1261	FC=1C=C(C=CC1)C1(CCCCC1)N
3F-PCHEPy	C17H24FN	261.1887	FC=1C=C(C=CC1)C1(CCCCCC1)N1CCCC1
3F-PCP	C17H24FN	261.1887	FC=1C=C(C=CC1)C1(CCCCC1)N1CCCCC1
3F-Phenetrazine	C12H16FNO	209.1211	C(C)C1NCCOC1C1=CC(=CC=C1)F
3-FPM-M,O,N-bis-dealkyl-	C9H12FNO	169.0898	CC(N)C(C1=CC=CC(F)=C1)O
3-FPP	C10H13FN2	180.1057	FC1=CC(N2CCNCC2)=CC=C1
3-Hydroxybromazepam	C14H10BrN3O2	330.9951	C1=CC=NC(=C1)C2=NC(C(=O)NC3=C2C=C(C=C3)Br)O
3-Hydroxyflubromazepam	C15H10BrFN2O2	347.9904	O=C1NC2=CC=C(Br)C=C2C(C3=CC=CC=C3F)=NC1O
3-Hydroxyphenazepam	C15H10BrClN2O2	363.9609	C1=CC=C(C(=C1)C2=NC(C(=O)NC3=C2C=C(C=C3)Br)O)Cl
3-MAPB	C12H15NO	189.1148	O1C=C(C2=C1C=CC=C2)CC(C)NC
3'-Me-4F-iBF	C24H31FN2O	382.2415	FC1=CC=C(C=C1)N(C(C(C)C)=O)C1CCN(CC1)CCC1=CC(=CC=C1)C
3-MEC	C12H17NO	191.1305	CC(NCC)C(C1=CC=CC(C)=C1)=O
3-MeOMA	C11H17NO	179.1305	CNC(C)CC1=CC=CC(OC)=C1
3-MeO-MC	C11H15NO2	193.1097	CC(NC)C(C1=CC=CC(OC)=C1)=O
3-MeO-PCE	C15H23NO	233.1774	C(C)NC1(CCCCC1)C1=CC(=CC=C1)OC

**Table S9.** Compound database imported into the *Search Mass Lists* node (continued)			
Compound Name	Molecular Formula	Exact Mass	SMILES
3-MeO-PCMMo	C18H27NO2	289.2036	COC1=CC(C2(CN3CCOCC3)CCCCC2)=CC=C1
3-MeO-PCMo	C17H25NO2	275.1880	COC1=CC(C2(N3CCOCC3)CCCCC2)=CC=C1
3-MeO-PCPr	C16H25NO	247.1931	COC=1C=C(C=CC1)C1(CCCCC1)NCCC
3-MeO-PCPy	C17H25NO	259.1931	COC=1C=C(C=CC1)C1(CCCCC1)N1CCCC1
3-Me-PBCHP	C19H27N	269.2138	CC=1C=C(C=CC1)C1(C2CCC(C1)C2)N2CCCCC2
3-Me-PBCHPr	C17H25N	243.1982	CC=1C=C(C=CC1)C1(C2CCC(C1)C2)NCCC
3-Me-PBCHPy	C18H25N	255.1982	CC=1C=C(C=CC1)C1(C2CCC(C1)C2)N2CCCC2
3-Me-PCA	C13H19N	189.1512	CC=1C=C(C=CC1)C1(CCCCC1)N
3-Me-PCBzl	C20H25N	279.1982	C(C1=CC=CC=C1)NC1(CCCCC1)C1=CC(=CC=C1)C
3-Me-PCE	C15H23N	217.1825	C(C)NC1(CCCCC1)C1=CC(=CC=C1)C
3’-Me-PCP	C18H27N	257.2138	CC1CC(CCC1)(C1=CC=CC=C1)N1CCCCC1
3-Me-PCP	C18H27N	257.2138	CC=1C=C(C=CC1)C1(CCCCC1)N1CCCCC1
3-Me-PCPr	C16H25N	231.1982	CC=1C=C(C=CC1)C1(CCCCC1)NCCC
3-Me-PCPy	C17H25N	243.1982	CC=1C=C(C=CC1)C1(CCCCC1)N1CCCC1
3-Methoxy-4-ethoxy N,N-Diethylhexedrone	C19H31NO3	321.2299	C(C)N(C(C(=O)C1=CC(=C(C=C1)OCC)OC)CCCC)CC
3-Methoxy-4-ethoxy N,N-Diethylpentedrone	C18H29NO3	307.2142	C(C)N(C(C(=O)C1=CC(=C(C=C1)OCC)OC)CCC)CC
3-Methoxyfentanyl	C23H30N2O2	366.2302	COC1CN(CCC1N(C(CC)=O)C1=CC=CC=C1)CCC1=CC=CC=C1
3-Methoxyphenmetrazine	C12H17NO2	207.1254	COC=1C=C(C=CC1)C1C(NCCO1)C
3-Methyl Fentanyl	C23H30N2O	350.2353	CCC(N(C1C(C)CN(CCC2=CC=CC=C2)CC1)C3=CC=CC=C3)=O
3-Methyl Phenoxy Acetylfentanil	C22H28N2O2	352.2145	CC1CN(CCC1N(C(C)=O)C1=CC=CC=C1)CCOC1=CC=CC=C1
3-Methylamphetamine	C10H15N	149.1199	CC(N)CC1=CC=CC(C)=C1
3-Methylbuphedrone	C12H17NO	191.1305	CCC(C(=O)C1=CC=CC(=C1)C)NC
3-Methylflephedrone	C11H14FNO	195.1054	CC(NC)C(C1=CC=C(F)C(C)=C1)=O
3-Methyl-furanylfentanyl	C25H28N2O2	388.2145	CC1CN(CCC1N(C(=O)C=1OC=CC1)C1=CC=CC=C1)CCC1=CC=CC=C1
3-Methylmethamphetamine	C11H17N	163.1356	CNC(CC1=CC(=CC=C1)C)C
3-Methylnordiazepam	C16H13ClN2O	284.0711	ClC=1C=CC2=C(C(=NC(C(N2)=O)C)C2=CC=CC=C2)C1
3-Methyl-N-propyl-cathinone	C13H19NO	205.1461	C(CC)NC(C(=O)C1=CC(=CC=C1)C)C
3-Methylphenmetrazine	C12H17NO	191.1305	CC1=CC(C2C(C)NCCO2)=CC=C1
3-Methylthio-fentanyl	C23H30N2OS	382.2073	CSC1CN(CCC1N(C(CC)=O)C1=CC=CC=C1)CCC1=CC=CC=C1
3-MMA	C10H15NO	165.1148	NC(C)CC1=CC=CC(OC)=C1
3-MMC	C11H15NO	177.1148	CC(NC)C(C1=CC=CC(C)=C1)=O
3-NH2-PCP	C17H26N2	258.2091	N1(CCCCC1)C1(CCCCC1)C=1C=C(N)C=CC1
3PYCP	C16H24N2	244.1934	N1(CCCCC1)C1(CCCCC1)C=1C=NC=CC1
4’-Hydroxy Nitazene	C20H24N4O3	368.1843	OC(C=C1)=CC=C1CC2=NC3=CC([N+]([O-])=O)=CC=C3N2CCN(CC)CC
4-(m-Hydroxyphenyl)fentanyl	C28H32N2O2	428.2458	OC=1C=C(C=CC1)C1(CCN(CC1)CCC1=CC=CC=C1)N(C(CC)=O)C1=CC=CC=C1
4,4-Dimethyl-1-phenyl-1-pyrrolidin-1-yl-pentan-3-one	C17H25NO	259.1931	CC(C(CC(N1CCCC1)C1=CC=CC=C1)=O)(C)C
4,4’-Methylenedianiline	C13H14N2	198.1152	C(C1=CC=C(N)C=C1)C1=CC=C(N)C=C1
4,5-MDAI	C10H11NO2	177.0784	NC1CC2=CC=C(OCO3)C3=C2C1
4-Acetoxy MiPT	C16H22N2O2	274.1676	CC(OC1=CC=CC2=C1C(CCN(C(C)C)C)=CN2)=O
4-Acetoxy PiPT	C18H26N2O2	302.1989	C(C)(=O)OC1=C2C(=CNC2=CC=C1)CCN(CCC)C(C)C
4-Acetoxy-MALT	C16H20N2O2	272.1519	CC(OC1=CC=CC2=C1C(CCN(CC=C)C)=CN2)=O
4-AcO-DALT	C18H22N2O2	298.1676	CC(OC1=CC=CC2=C1C(CCN(CC=C)CC=C)=CN2)=O

**Table S9.** Compound database imported into the *Search Mass Lists* node (continued)			
Compound Name	Molecular Formula	Exact Mass	SMILES
4-AcO-DET	C16H22N2O2	274.1676	C(C)(=O)OC1=C2C(=CNC2=CC=C1)CCN(CC)CC
4-AcO-DIPT	C18H26N2O2	302.1989	C(C)(=O)OC1=C2C(=CNC2=CC=C1)CCN(C(C)C)C(C)C
4-AcO-DMT	C14H18N2O2	246.1363	CC(OC1=CC=CC2=C1C(CCN(C)C)=CN2)=O
4-AcO-DPT	C18H26N2O2	302.1989	C(C)(=O)OC1=C2C(=CNC2=CC=C1)CCN(CCC)CCC
4-ACO-EPT	C17H24N2O2	288.1832	C(C)(=O)OC1=C2C(=CNC2=CC=C1)CCN(CCC)CC
4-AcO-MET	C15H20N2O2	260.1519	C(C)(=O)OC1=C2C(=CNC2=CC=C1)CCN(C)CC
4-AcO-MPT	C16H22N2O2	274.1676	CC(OC1=CC=CC2=C1C(CCN(C)CCC)=CN2)=O
4"-AcO-TCP	C17H25NO2S	307.1601	C(C)(=O)OC1CCN(CC1)C1(CCCCC1)C=1SC=CC1
4-APB	C11H13NO	175.0992	CC(N)CC1=CC=CC2=C1C=CO2
4-APDB	C11H15NO	177.1148	NC(CC1=CC=CC2=C1CCO2)C
4-BEC	C11H14BrNO	255.0253	CC(NCC)C(C1=CC=C(Br)C=C1)=O
4"-Benzoyloxy-PCP	C24H29NO2	363.2193	C(C1=CC=CC=C1)(=O)OC1CCN(CC1)C1(CCCCC1)C1=CC=CC=C1
4-Benzylpiperidine	C12H17N	175.1356	C1(CC2=CC=CC=C2)CCNCC1
4-BPD	C12H16BrNO	269.0410	BrC1=CC=C(C=C1)C(C(CCC)NC)=O
4Br-alpha-PPP	C13H16BrNO	281.0410	CC(N1CCCC1)C(C2=CC=C(Br)C=C2)=O
4Br-alpha-PVP	C15H20BrNO	309.0723	CCCC(N1CCCC1)C(C2=CC=C(Br)C=C2)=O
4Br-MAR	C10H11BrN2O	254.0049	BrC1=CC=C(C=C1)C1C(N=C(O1)N)C
4-Bromo-2,5-DMMA	C12H18BrNO2	287.0516	BrC1=CC(=C(C=C1OC)CC(NC)C)OC
4-Bromoamphetamine	C9H12BrN	213.0148	CC(N)CC1=CC=C(Br)C=C1
4-Bromomethamphetamine	C10H14BrN	227.0304	CC(NC)CC1=CC=C(Br)C=C1
4-Bromomethcathinone	C10H12BrNO	241.0097	BrC1=CC=C(C=C1)C(C(C)NC)=O
4"-Bromo-ohmefentanyl	C23H29BrN2O2	444.1407	BrC1=CC=C(C=C1)C(CN1CC(C(CC1)N(C(CC)=O)C1=CC=CC=C1)C)O
4-Br-PCP	C17H24BrN	321.1087	BrC1=CC=C(C=C1)C1(CCCCC1)N1CCCCC1
4-CAB	C10H14ClN	183.0809	ClC1=CC=C(C=C1)CC(N)CC
4-CBC	C13H18ClNO	239.1072	C(CCC)NC(C(=O)C1=CC=C(C=C1)Cl)C
4C-D	C13H21NO2	223.1567	CCC(N)CC1=CC(OC)=C(C)C=C1OC
4-Chloro CUMYL-PINACA	C22H26ClN3O	383.1759	CCCCCn1nc(C(=O)NC(C)(C)c2ccc(Cl)cc2)c2ccccc21
4-Chloro MDMB-BUTICA	C20H27ClN2O3	378.1705	ClCCCCN1C=C(C2=CC=CC=C12)C(=O)N[C@H](C(=O)OC)C(C)(C)C
4-Chloro-3-methylmethcathinone	C11H14ClNO	211.0759	CNC(C(=O)C1=CC(=C(C=C1)C)Cl)C
4-Chloroamphetamine	C9H12ClN	169.0653	ClC1=CC=C(C=C1)CC(C)N
4-Chloro-Buphedrone	C11H14ClNO	211.0759	ClC1=CC=C(C=C1)C(C(CC)NC)=O
4-Chlorocathinone	C9H10ClNO	183.0446	NC(C(=O)C1=CC=C(C=C1)Cl)C
4'-Chloro-deschloroalprazolam	C17H13ClN4	308.0823	ClC1=CC=C(C=C1)C1=NCC=2N(C3=C1C=CC=C3)C(=NN2)C
4-Chloroethcathinone	C11H14ClNO	211.0759	CC(NCC)C(C1=CC=C(Cl)C=C1)=O
4-Chloromethcathinone	C10H12ClNO	197.0602	ClC1=CC=C(C=C1)C(C(C)NC)=O
4-Chloro-N,N-dimethylcathinone	C11H14ClNO	211.0759	CC(N(C)C)C(C1=CC=C(Cl)C=C1)=O
4-CIC	C12H16ClNO	225.0915	ClC1=CC=C(C=C1)C(C(C)NC(C)C)=O
4-Cl-3-MMC	C11H14ClNO	211.0759	ClC1=C(C=C(C=C1)C(C(C)NC)=O)C
4-Cl-alpha-PPP	C13H16ClNO	237.0915	CC(N1CCCC1)C(C2=CC=C(Cl)C=C2)=O
4Cl-MAR	C10H11ClN2O	210.0555	ClC1=CC=C(C=C1)C1C(N=C(O1)N)C
4-Cl-PCDM	C16H24ClN	265.1592	ClC1=CC=C(C=C1)C1(CCCCC1)N(CC)CC
4-Cl-PCE	C14H20ClN	237.1279	ClC1=CC=C(C=C1)C1(CCCCC1)NCC

**Table S9.** Compound database imported into the *Search Mass Lists* node (continued)			
Compound Name	Molecular Formula	Exact Mass	SMILES
4-Cl-PCP	C17H24ClN	277.1592	ClC1=CC=C(C=C1)C1(CCCCC1)N1CCCCC1
4-Cl-Pentedrone	C12H16ClNO	225.0915	CCCC(NC)C(C1=CC=C(Cl)C=C1)=O
4Cl-PVP	C15H20ClNO	265.1228	CCCC(N1CCCC1)C(C2=CC=C(Cl)C=C2)=O
4-CMA	C10H14ClN	183.0809	CC(NC)CC1=CC=C(Cl)C=C1
4CN-AB-BUTICA	C19H24N4O2	340.1894	O=C(N[C@@H](C(C)C)C(N)=O)C1=CN(CCCCC#N)C2=C1C=CC=C2
4-Cyano ADB-BUTINACA	C19H25N5O2	355.2003	NC(C(C(C)(C)C)NC(=O)C1=NN(C2=CC=CC=C12)CCCCC#N)=O
4-Cyano CUMYL-BUT7AICA	C22H24N4O	360.1945	O=C(C1=CN(CCCCC#N)C2=NC=CC=C21)NC(C3=CC=CC=C3)(C)C
4-Cyano CUMYL-BUTCZCA	C27H27N3O	409.2149	C(#N)CCCCN1C2=CC=CC=C2C=2C=C(C=CC12)C(=O)NC(C)(C)C1=CC=CC=C1
4-Cyano CUMYL-BUTINACA N-Butanoic Acid	C21H23N3O3	365.1734	C1(=CC=CC=C1)C(C)(C)NC(=O)C1=NN(C2=CC=CC=C12)CCCC(=O)O
4-Cyano MDMB-BUTINACA	C20H26N4O3	370.1999	COC(=O)C(NC(=O)c1nn(CCCCC#N)c2ccccc12)C(C)(C)C
4-Cyano MMB-BUTINACA N-Butanoic Acid 3-Methylbutanoic Acid	C17H21N3O5	347.1476	C(=O)(O)CCCN1N=C(C2=CC=CC=C12)C(=O)N[C@@H](C(C)C)C(=O)O
4-Cyano-AMB-BUTINACA	C19H24N4O3	356.1843	O=C(N[C@@H](C(C)C)C(OC)=O)C1=NN(CCCCC#N)C2=C1C=CC=C2
4-DEA-PCE	C18H30N2	274.2404	C(C)N(C1=CC=C(C=C1)C1(CCCCC1)NCC)CC
4-Desoxymescaline	C11H17NO2	195.1254	NCCC1=CC(OC)=C(C)C(OC)=C1
4-DHBF-PCA	C14H19NO	217.1461	O1CCC2=C1C=CC(=C2)C2(CCCCC2)N
4-DHBF-PCP	C19H27NO	285.2087	O1CCC2=C1C=CC(=C2)C2(CCCCC2)N2CCCCC2
4-EA NBOMe	C19H25NO	283.1931	CC(NCC1=CC=CC=C1OC)CC2=CC=C(CC)C=C2
4-EAPB	C13H17NO	203.1305	CC(NCC)CC1=CC=CC2=C1C=CO2
4-EEC	C13H19NO	205.1461	CCNC(C)C(=O)C1=CC=C(CC)C=C1
4-EPD	C14H21NO	219.1618	C(C)C1=CC=C(C=C1)C(C(CCC)NC)=O
4'-Ethyl-alpha-PVP	C17H25NO	259.1931	C(C)C1=CC=C(C=C1)C(C(CCC)N1CCCC1)=O
4-Ethylamphetamine	C11H17N	163.1356	CC(N)CC1=CC=C(CC)C=C1
4-Ethylephedrine	C12H19NO	193.1461	CC(NC)C(C1=CC=C(CC)C=C1)O
4-Ethylmethcathinone	C12H17NO	191.1305	CC(NC)C(C1=CC=C(CC)C=C1)=O
4-Ethyl-N,N-DMC	C13H19NO	205.1461	CC(N(C)C)C(C1=CC=C(CC)C=C1)=O
4-F MDMB-BICA N-(4-Hydroxybutyl) Metabolite	C20H28N2O4	360.2044	OCCCCN1C=C(C2=CC=CC=C12)C(=O)N[C@H](C(=O)OC)C(C)(C)C
4F-alpha-PHiP	C16H22FNO	263.1680	FC1=CC=C(C=C1)C(C(CC(C)C)N1CCCC1)=O
4F-alpha-PVP	C15H20FNO	249.1524	CCCC(N1CCCC1)C(C2=CC=C(F)C=C2)=O
4F-alpha-PBP	C14H18FNO	235.1367	CCC(N1CCCC1)C(C2=CC=C(F)C=C2)=O
4F-Buphedrone	C11H14FNO	195.1054	CCC(NC)C(C1=CC=C(F)C=C1)=O
4-FEC	C11H14FNO	195.1054	CCNC(C)C(=O)C1=CC=C(C=C1)F
4F-Ephedrine	C10H14FNO	183.1054	C[C@H](NC)[C@@H](C1=CC=C(F)C=C1)O
4-Fluoro ABUTINACA N-(4-Hydroxybutyl) Metabolite	C22H29N3O2	367.2254	C12(CC3CC(CC(C1)C3)C2)NC(=O)C2=NN(C3=CC=CC=C23)CCCCO
4-Fluoro ADB	C20H28FN3O3	377.2109	CC(C)(C)[C@H](NC(C1=NN(CCCC(F)C)C2=C1C=CC=C2)=O)C(OC)=O
4-Fluoro AMB	C19H26FN3O3	363.1953	COC(=O)C(NC(=O)c1nn(CCCC(C)F)c2ccccc12)C(C)C
4'-Fluoro Diazepam	C16H12ClFN2O	302.0617	ClC=1C=CC2=C(C(=NCC(N2C)=O)C2=CC=C(C=C2)F)C1
4-Fluoro EDMB-BUTINACA	C20H28FN3O3	377.2109	FCCCCN1N=C(C2=CC=CC=C12)C(=O)N[C@H](C(=O)OCC)C(C)(C)C
4-Fluoro EMB-BUTICA	C20H27FN2O3	362.2000	FCCCCN1C=C(C2=CC=CC=C12)C(=O)N[C@@H](C(C)C)C(=O)OCC
4-Fluoro MDMB-BUTICA Butanoic Acid Metabolite	C19H25FN2O3	348.1844	O=C(N[C@H](C(O)=O)C(C)(C)C)C1=CN(CCCCF)C2=C1C=CC=C2
4-Fluoro MDMB-BUTINACA 2'-Indazole Isomer	C19H26FN3O3	363.1953	CC(C)(C)[C@H](NC(C1=C2C=CC=CC2=NN1CCCCF)=O)C(OC)=O
4-Fluoro MDMB-BUTINACA 2'-Isomer Butanoic Acid	C18H24FN3O3	349.1796	FCCCCN1N=C2C=CC=CC2=C1C(=O)N[C@H](C(=O)O)C(C)(C)C
4-Fluoro MDMB-BUTINACA 3-Carboxy-2'-indazole Metabolite	C12H13FN2O2	236.0956	O=C(O)C1=C2C(C=CC=C2)=NN1CCCCF

**Table S9.** Compound database imported into the *Search Mass Lists* node (continued)			
Compound Name	Molecular Formula	Exact Mass	SMILES
4-Fluoro MDMB-BUTINACA 3-Carboxyindazole Metabolite	C12H13FN2O2	236.0956	O=C(O)c1nn(CCCCF)c2ccccc12
4-Fluoro MDMB-BUTINACA Butanoic Acid	C18H24FN3O3	349.1796	O=C(N[C@H](C(O)=O)C(C)(C)C)C1=NN(CCCCF)C2=C1C=CC=C2
4-Fluoro MDMB-BUTINACA Butanoic Acid Metabolite	C18H24FN3O3	349.1796	CC(C)(C)C(NC(=O)c1nn(CCCCF)c2ccccc12)C(=O)O
4-Fluoro MDMB-BUTINACA N-(4-hydroxybutyl) Metabolite	C19H27N3O4	361.1996	CC(C)(C)[C@H](NC(C1=NN(CCCCO)C2=C1C=CC=C2)=O)C(OC)=O
4-Fluoro-3-methyl alpha-PVP	C16H22FNO	263.1680	FC1=C(C=C(C=C1)C(C(CCC)N1CCCC1)=O)C
4-Fluoroamphetamine	C9H12FN	153.0948	CC(N)CC1=CC=C(F)C=C1
4-Fluorocathinone	C9H10FNO	167.0741	CC(N)C(C1=CC=C(F)C=C1)=O
4-Fluoro-CUMYL-5-fluoro-PICA	C23H26F2N2O	384.2008	CC(C)(NC(=O)c1cn(CCCCCF)c2ccccc12)c1ccc(F)cc1
4-Fluoro-CUMYL-5-fluoro-PINACA	C22H25F2N3O	385.1960	CC(C)(NC(=O)c1nn(CCCCCF)c2ccccc12)c1ccc(F)cc1
4-Fluoroethamphetamine	C11H16FN	181.1261	CC(NCC)CC1=CC=C(F)C=C1
4-Fluoroethylphenidate	C15H20FNO2	265.1473	FC1=CC=C(C=C1)C(C(=O)OCC)C1NCCCC1
4-Fluorofentanyl	C22H27FN2O	354.2102	FC1(CCN(CC1)CCC1=CC=CC=C1)N(C(CC)=O)C1=CC=CC=C1
4-Fluoromethamphetamine	C10H14FN	167.1105	CC(NC)CC1=CC=C(F)C=C1
4-Fluoromethylphenidate	C14H18FNO2	251.1316	FC1=CC=C(C=C1)C(C(=O)OC)C2CCCCN2
4-Fluoro-N-ethyl-pentedrone	C13H18FNO	223.1367	C(C)NC(C(=O)C1=CC=C(C=C1)F)CCC
4”-Fluoro-ohmefentanyl	C23H29FN2O2	384.2208	FC1=CC=C(C=C1)C(CN1CC(C(CC1)N(C(CC)=O)C1=CC=CC=C1)C)O
4-Fluoropentedrone	C12H16FNO	209.1211	FC1=CC=C(C=C1)C(C(CCC)NC)=O
4-Fluorophenibut	C10H12FNO2	197.0847	O=C(O)CC(C1=CC=C(F)C=C1)CN
4-Fluorophenmetrazine	C11H14FNO	195.1054	FC1=CC=C(C=C1)C1C(NCCO1)C
4-Fluorophenylacetone	C9H9FO	152.0632	FC1=CC=C(C=C1)CC(C)=O
4-Fluorotropacocaine	C15H18FNO2	263.1316	FC1=CC=C(C(=O)OC2C[C@H]3CC[C@@H](C2)N3C)C=C1
4F-MBZP	C12H17FN2	208.1370	FC1=CC=C(C=C1)CN1CCN(CC1)C
4-FMC	C10H12FNO	181.0898	FC1=CC=C(C=C1)C(C(C)NC)=O
4F-MDMB-BUTICA N-(Butanoic Acid) Metabolite	C20H26N2O5	374.1836	COC([C@H](C(C)(C)C)NC(=O)C1=CN(C2=CC=CC=C12)CCCC(=O)O)=O
4F-MDMB-BUTINACA N-Butanoic Acid Metabolite	C19H25N3O5	375.1789	O=C(N[C@H](C(OC)=O)C(C)(C)C)C1=NN(CCCC(O)=O)C2=C1C=CC=C2
4F-NEB	C12H16FNO	209.1211	CCC(NCC)C(C1=CC=C(F)C=C1)=O
4F-NNEI	C24H23FN2O	374.1789	O=C(C1=CN(CCCC(F)C)C2=C1C=CC=C2)NC3=C4C=CC=CC4=CC=C3
4F-NPP	C14H20FNO	237.1524	FC1=CC=C(C=C1)C(C(CCC)NC(C)C)=O
4F-PBCHA	C13H16FN	205.1261	FC1=CC=C(C=C1)C1(C2CCC(C1)C2)N
4F-PBCHP	C18H24FN	273.1887	FC1=CC=C(C=C1)C1(C2CCC(C1)C2)N2CCCCC2
4F-PBCHPy	C17H22FN	259.1731	FC1=CC=C(C=C1)C1(C2CCC(C1)C2)N2CCCC2
4F-PCHEPy	C17H24FN	261.1887	FC1=CC=C(C=C1)C1(CCCCCC1)N1CCCC1
4F-PCP	C17H24FN	261.1887	FC1=CC=C(C=C1)C1(CCCCC1)N1CCCCC1
4F-PHP	C16H22FNO	263.1680	CCCCC(N1CCCC1)C(C2=CC=C(F)C=C2)=O
4F-PV8	C17H24FNO	277.1837	CCCCCC(N1CCCC1)C(C2=CC=C(F)C=C2)=O
4F-PV8 Piperidine Analogue	C18H26FNO	291.1993	FC1=CC=C(C=C1)C(C(CCCCC)N1CCCCC1)=O
4F-PV9	C18H26FNO	291.1993	CCCCCCC(N1CCCC1)C(C2=CC=C(F)C=C2)=O
4-HO-DBT	C18H28N2O	288.2196	CCCCN(CCCC)CCC1=CNC2=C1C(O)=CC=C2
4-HO-DET	C14H20N2O	232.1570	C(C)N(CCC1=CNC=2C=CC=C(C12)O)CC
4-HO-MET	C13H18N2O	218.1414	C(C)N(CCC1=CNC=2C=CC=C(C12)O)C
4-HO-MiPT	C14H20N2O	232.1570	C(C)(C)N(CCC1=CNC=2C=CC=C(C12)O)C
4-HO-MPMI	C14H18N2O	230.1414	CN1C(CCC1)CC1=CNC=2C=CC=C(C12)O

Table S9. Compound database imported into the *Search Mass Lists* node (continued)			
Compound Name	Molecular Formula	Exact Mass	SMILES
4-HO-MPT	C14H20N2O	232.1570	CN(CCC1=CNC=2C=CC=C(C12)O)CCC
4-HO-Pyr-T	C14H18N2O	230.1414	OC1=CC=CC2=C1C(CCN3CCCC3)=CN2
4-HTMPIPO	C21H31NO2	329.2349	CC(C)(O)C(C)(C)CC(C1=CN(CCCCC)C2=C1C=CC=C2)=O
4-Hydroxy Amphetamine	C9H13NO	151.0992	NC(CC1=CC=C(C=C1)O)C
4-Hydroxy DPT	C16H24N2O	260.1883	OC1=CC=CC2=C1C(CCN(CCC)CCC)=CN2
4-Hydroxy EPT	C15H22N2O	246.1727	OC=1C=CC=C2NC=C(CCN(CCC)CC)C12
4-Hydroxy MALT	C14H18N2O	230.1414	CN(CCC1=CNC=2C=CC=C(C12)O)CC=C
4-Hydroxy McPT	C14H18N2O	230.1414	OC1=CC=CC2=C1C(CCN(C3CC3)C)=CN2
4'-Hydroxyclobazam	C16H13ClN2O3	316.0609	ClC1=CC2=C(N(C(CC(N2C2=CC=C(C=C2)O)=O)=O)C)C=C1
4-Hydroxymethamphetamine	C10H15NO	165.1148	CNC(CC1=CC=C(C=C1)O)C
4-Hydroxymethcathinone	C10H13NO2	179.0941	CC(NC)C(C1=CC=C(O)C=C1)=O
4-Hydroxymethyl PCP	C18H27NO	273.2087	N1(CCCCC1)C1(CCCCC1)C1=CC=C(C=C1)CO
4-MA-NBOMe	C18H23NO	269.1774	CC(NCC1=CC=CC=C1OC)CC2=CC=C(C)C=C2
4-MAPB	C12H15NO	189.1148	CNC(CC1=CC=CC2=C1C=CO2)C
4-MEC	C12H17NO	191.1305	C(C)NC(C(=O)C1=CC=C(C=C1)C)C
4-MeO-alpha-PBP	C15H21NO2	247.1567	CCC(N1CCCC1)C(C2=CC=C(OC)C=C2)=O
4-MeO-alpha-PEP	C18H27NO2	289.2036	CCCCCC(N1CCCC1)C(C2=CC=C(OC)C=C2)=O
4-MeO-PCMo	C17H25NO2	275.1880	COC1=CC=C(C=C1)C1(CCCCC1)N1CCOCC1
4-MeOPP	C11H16N2O	192.1257	COC1=CC=C(N2CCNCC2)C=C1
4-MeO-PV9	C19H29NO2	303.2193	COC1=CC=C(C=C1)C(C(CCCCCC)N1CCCC1)=O
4-MeO-PVP	C16H23NO2	261.1723	CCCC(N1CCCC1)C(C2=CC=C(OC)C=C2)=O
4-Me-PCDM	C17H27N	245.2138	C(C)N(C1(CCCCC1)C1=CC=C(C=C1)C)CC
4-Me-PCMo	C17H25NO	259.1931	CC1=CC=C(C=C1)C1(CCCCC1)N1CCOCC1
4'-Me-PCP	C18H27N	257.2138	CC1CCC(CC1)(C1=CC=CC=C1)N1CCCCC1
4-Methoxy DiPT	C17H26N2O	274.2040	CC(N(CCC1=CNC2=C1C(OC)=CC=C2)C(C)C)C
4-Methoxy DMT	C13H18N2O	218.1414	COC1=CC=CC2=C1C(CCN(C)C)=CN2
4-Methoxy MiPT	C15H22N2O	246.1727	COC1=C2C(=CNC2=CC=C1)CCN(C(C)C)C
4-Methoxy PCE	C15H23NO	233.1774	C(C)NC1(CCCCC1)C1=CC=C(C=C1)OC
4''-Methoxyfentanyl	C23H30N2O2	366.2302	COC1=CC=C(C=C1)CCN1CCC(CC1)N(C(CC)=O)C1=CC=CC=C1
4-Methoxy-N,N-Dimethylcathinone	C12H17NO2	207.1254	CN(C(C(=O)C1=CC=C(C=C1)OC)C)C
4-Methyl AET	C13H18N2	202.1465	CC1=C2C(NC=C2CC(N)CC)=CC=C1
4'-Methyl Hexedrone	C14H21NO	219.1618	CNC(C(=O)C1=CC=C(C=C1)C)CCCC
4'-Methyl-alpha-PiHP	C17H25NO	259.1931	CC(CC(C(=O)C1=CC=C(C=C1)C)N1CCCC1)C
4-Methylaminoantipyrine	C12H15N3O	217.1210	CN1N(C(C(=C1C)NC)=O)C1=CC=CC=C1
4-Methylaminorex	C10H12N2O	176.0944	NC1=NC(C)C(C2=CC=CC=C2)O1
4-Methylamphetamine	C10H15N	149.1199	CC(N)CC1=CC=C(C)C=C1
4-Methylbuphedrone	C12H17NO	191.1305	CCC(NC)C(C1=CC=C(C)C=C1)=O
4-Methylbuphedrone, N-Benzyl Derivative	C18H21NO	267.1618	CCC(NCC1=CC=CC=C1)C(C2=CC=C(C)C=C2)=O
4-Methyldiethcathinone	C14H21NO	219.1618	CC(N(CC)CC)C(C1=CC=C(C)C=C1)=O
4-Methyl-N,N-dimethylcathinone	C12H17NO	191.1305	CC(N(C)C)C(C1=CC=C(C)C=C1)=O
4-Methyl-N-rthylhexanophenone	C15H23NO	233.1774	C(C)NC(C(=O)C1=CC=C(C=C1)C)CCCC
4-Methyl-N-ethyl-norephedrine	C12H19NO	193.1461	CC(NCC)C(C1=CC=C(C)C=C1)O

**Table S9.** Compound database imported into the *Search Mass Lists* node (continued)			
Compound Name	Molecular Formula	Exact Mass	SMILES
4-Methyl-N-ethyl-pentedrone	C14H21NO	219.1618	CCCC(NCC)C(C1=CC=C(C)C=C1)=O
4’-Methyl-NiPP	C15H23NO	233.1774	CC1=CC=C(C=C1)C(C(CCC)NC(C)C)=O
4-Methyl-N-propylcathinone	C13H19NO	205.1461	CC1=CC=C(C=C1)C(C(C)NCCC)=O
4-Methyl-PCP	C18H27N	257.2138	CC1=CC=C(C=C1)C1(CCCCC1)N1CCCCC1
4-Methylpentedrone	C13H19NO	205.1461	CNC(C(=O)C1=CC=C(C=C1)C)CCC
4-Methylphendimetrazine	C13H19NO	205.1461	CC1=CC=C(C2C(C)N(C)CCO2)C=C1
4’-Methylpropiophenone	C10H12O	148.0883	CC1=CC=C(C=C1)C(CC)=O
4-Methyl-PV8	C18H27NO	273.2087	CCCCCC(N1CCCC1)C(C2=CC=C(C)C=C2)=O
4-Methylthio-N-benzylcathinone	C17H19NOS	285.1182	C(C1=CC=CC=C1)NC(C(=O)C1=CC=C(C=C1)SC)C
4-MeTMP	C15H21NO2	247.1567	O=C(OC)[C@H](C1=CC=C(C)C=C1)[C@@H]2NCCCC2
4-MMA	C11H17N	163.1356	CC(NC)CC1=CC=C(C)C=C1
4-MMA NBOMe	C19H25NO	283.1931	CC(N(CC1=CC=CC=C1OC)C)CC2=CC=C(C)C=C2
4-MPH	C12H17NO	191.1305	CC1=CC=C(C2C(C)NCCO2)C=C1
4-MTA	C10H15NS	181.0920	CSC1=CC=C(C=C1)CC(C)N
4”-Nitrofentanyl	C22H27N3O3	381.2047	[N+](=O)([O-])C1=CC=C(C=C1)CCN1CCC(CC1)N(C(CC)=O)C1=CC=CC=C1
4-OH-DALT	C16H20N2O	256.1570	OC1=CC=CC2=C1C(CCN(CC=C)CC=C)=CN2
4’-Oxo-PCP	C17H23NO	257.1774	C1(=CC=CC=C1)C1(CCC(CC1)=O)N1CCCCC1
4-PhO-PCP	C23H29NO	335.2244	O(C1=CC=CC=C1)C1=CC=C(C=C1)C1(CCCCC1)N1CCCCC1
4-PrO-DMT	C15H20N2O2	260.1519	CCC(OC1=CC=CC2=C1C(CCN(C)C)=CN2)=O
4”-PrO-TCP	C18H27NO2S	321.1757	C(CC)(=O)OC1CCN(CC1)C1(CCCCC1)C=1SC=CC1
4PYCP	C16H24N2	244.1934	N1(CCCCC1)C1(CCCCC1)C1=CC=NC=C1
4’-tert-Butyl-PCP	C21H33N	299.2608	C(C)(C)(C)C1CCC(CC1)(C1=CC=CC=C1)N1CCCCC1
4-TFM U-47700	C17H23F3N2O	328.1757	CN([C@H]1[C@@H](CCCC1)N(C(C1=CC=C(C=C1)C(F)(F)F)=O)C)C
5,3-AB-CHMFUPPYCA	C22H29FN4O2	400.2269	CC(C)C(NC(=O)c1cc(-c2ccc(F)cc2)n(CC2CCCCC2)n1)C(N)=O
5,3-ADB-4en-PFUPPYCA	C21H27FN4O2	386.2113	NC([C@H](C(C)(C)C)NC(=O)C1=NN(C(=C1)C1=CC=C(C=C1)F)CCCC=C)=O
5,6-MeO-MiPT	C16H24N2O2	276.1832	COC1=C(C=C2NC=C(CCN(C(C)C)C)C2=C1)OC
5,7-Dichloro Tryptamine	C10H10Cl2N2	228.0216	ClC=1C=C2C(=CNC2=C(C1)Cl)CCN
5-AEDB	C10H13NO	163.0992	NCCC1=CC2=C(OCC2)C=C1
5-Aminoisotonitazene	C23H32N4O	380.2571	NC1=CC=C2C(N=C(CC3=CC=C(OC(C)C)C=C3)N2CCN(CC)CC)=C1
5-APB	C11H13NO	175.0992	CC(N)CC1=CC=C(OC=C2)C2=C1
5-APB NBOMe	C19H21NO2	295.1567	CC(NCC1=CC=CC=C1OC)CC2=CC3=C(OC=C3)C=C2
5-APDB	C11H15NO	177.1148	CC(N)CC1=CC=C(OCC2)C2=C1
5-APDI	C12H17N	175.1356	CC(N)CC1=CC2=C(CCC2)C=C1
5-BPDi	C19H27NO	285.2087	CCCCC(N1CCCC1)C(C2=CC3=C(CCC3)C=C2)=O
5-Br-DMT	C12H15BrN2	266.0413	BrC=1C=C2C(=CNC2=CC1)CCN(C)C
5-Bromo APINACA	C23H30BrN3O	443.1567	O=C(NC12CC3CC(CC(C3)C1)C2)c1nn(CCCCCBr)c2ccccc12
5-Bromo THJ 018	C23H21BrN2O	420.0832	O=C(C1=NN(CCCCCBr)C2=C1C=CC=C2)C3=C4C=CC=CC4=CC=C3
5-Chloro AB-PINACA	C18H25ClN4O2	364.1661	O=C(C1=NN(CCCCCCl)C2=C1C=CC=C2)NC(C(N)=O)C(C)C
5-Chloro AKB48	C23H30ClN3O	399.2072	O=C(C1=NN(CCCCCCl)C2=C1C=CC=C2)NC34CC5CC(C4)CC(C5)C3
5-Chloro DMT	C12H15ClN2	222.0918	ClC1=CC=C2NC=C(CCN(C)C)C2=C1
5-Chloro MDMB-PICA	C21H29ClN2O3	392.1861	ClCCCCCN1C=C(C2=CC=CC=C12)C(=O)N[C@H](C(=O)OC)C(C)(C)C
5-Chloro NNEI	C24H23ClN2O	390.1494	O=C(C1=CN(CCCCCCl)C2=C1C=CC=C2)NC3=C4C=CC=CC4=CC=C3

**Table S9.** Compound database imported into the *Search Mass Lists* node (continued)			
Compound Name	Molecular Formula	Exact Mass	SMILES
5-Chloro THJ 018	C23H21ClN2O	376.1337	O=C(c1cccc2ccccc12)c1nn(CCCCCCl)c2ccccc12
5-Chloro Tryptamine	C10H11ClN2	194.0605	ClC=1C=C2C(=CNC2=CC1)CCN
5Cl-alpha MT	C11H13ClN2	208.0762	ClC=1C=C2C(=CNC2=CC1)CC(C)N
5Cl-bk-MPA	C8H10ClNOS	203.0166	CC(NC)C(C1=CC=C(Cl)S1)=O
5Cl-MDMB-PINACA	C20H28ClN3O3	393.1814	CC(C)(C)C(NC(C1=NN(CCCCCCl)C2=C1C=CC=C2)=O)C(OC)=O
5-DBFPV	C17H23NO2	273.1723	CCCC(N1CCCC1)C(C2=CC=C(OCC3)C3=C2)=O
5-EAPB	C13H17NO	203.1305	O1C=CC2=C1C=CC(=C2)CC(C)NCC
5F-AB-001	C24H30FNO	367.2306	C12(CC3CC(CC(C1)C3)C2)C(=O)C2=CN(C3=CC=CC=C23)CCCCCF
5F-AB-FUPPYCA	C20H26F2N4O2	392.2018	O=C(C1=NN(CCCCCF)C(C2=CC=C(F)C=C2)=C1)NC(C(C)C)C(N)=O
5F-ABICA	C19H26FN3O2	347.2004	O=C(C1=CN(CCCCCF)C2=C1C=CC=C2)N[C@H](C(N)=O)C(C)C
5F-ADB	C20H28FN3O3	377.2109	CC(C)(C)[C@H](C(OC)=O)NC(C1=NN(CCCCCF)C2=C1C=CC=C2)=O
5F-ADB Metabolite 2	C20H29N3O4	375.2153	COC(=O)C(NC(=O)c1nn(CCCCCO)c2ccccc12)C(C)(C)C
5F-ADBICA	C20H28FN3O2	361.2160	O=C(C1=CN(CCCCCF)C2=C1C=CC=C2)NC(C(N)=O)C(C)(C)C
5F-AEB	C20H28FN3O3	377.2109	O=C(N[C@H](C(OCC)=O)C(C)C)C1=NN(CCCCCF)C2=CC=CC=C21
5F-AKB48	C23H30FN3O	383.2367	O=C(C1=NN(CCCCCF)C2=C1C=CC=C2)NC34CC5CC(C4)CC(C5)C3
5F-AMB	C19H26FN3O3	363.1953	CC(C)C(NC(C1=NN(CCCCCF)C2=C1C=CC=C2)=O)C(OC)=O
5F-A-P7AICA	C23H30FN3O	383.2367	C12(CC3CC(CC(C1)C3)C2)NC(=O)C2=CN(C3=NC=CC=C32)CCCCCF
5F-APINAC	C23H29FN2O2	384.2208	O=C(C1=NN(CCCCCF)C2=C1C=CC=C2)OC34CC5CC(C4)CC(C5)C3
5F-BZO-POXIZID	C20H20FN3O2	353.1534	FCCCCCN1C(C(C2=CC=CC=C12)=NNC(C1=CC=CC=C1)=O)=O
5F-CUMYL-P7AICA	C22H26FN3O	367.2054	O=C(C1=CN(CCCCCF)C2=NC=CC=C12)NC(C)(C3=CC=CC=C3)C
5F-Cumyl-PeGACLONE	C25H27FN2O	390.2102	FCCCCCN1C2=C(C=3C=CC=CC13)C(N(C=C2)C(C)(C)C2=CC=CC=C2)=O
5F-CUMYL-PICA	C23H27FN2O	366.2102	CC(C)(C1=CC=CC=C1)NC(=O)C2=CN(C3=CC=CC=C32)CCCCCF
5F-CUMYL-PINACA	C22H26FN3O	367.2054	O=C(C1=NN(CCCCCF)C2=C1C=CC=C2)NC(C)(C3=CC=CC=C3)C
5F-EDMB-PICA	C22H31FN2O3	390.2313	FCCCCCN1C=C(C2=CC=CC=C12)C(=O)NC(C(=O)OCC)C(C)(C)C
5F-EMB-PICA	C21H29FN2O3	376.2157	FCCCCCN1C=C(C2=CC=CC=C12)C(=O)NC(C(=O)OCC)C(C)C
5-Fluoro 7-QUPAIC	C22H20FN3O2	377.1534	O=C(Oc1cccc2cccnc12)c1cn(CCCCCF)c2ncccc12
5-Fluoro AB-7-PAICA	C18H25FN4O2	348.1956	O=C(C1=CN(CCCCCF)C2=NC=CC=C21)NC(C(C)C)C(N)=O
5-Fluoro AB-PINACA	C18H25FN4O2	348.1956	O=C(C1=NN(CCCCCF)C2=C1C=CC=C2)N[C@@H](C(C)C)C(N)=O
5-Fluoro AB-PINACA 3-Carboxyindazole Metabolite	C13H15FN2O2	250.1112	O=C(O)c1nn(CCCCCF)c2ccccc12
5-Fluoro AB-PINACA N-(4-Hydroxypentyl) Metabolite	C18H25FN4O3	364.1905	O=C(C1=NN(CCCC(O)CF)C2=C1C=CC=C2)NC(C(C)C)C(N)=O
5-Fluoro ADB 2’-Indazole Isomer	C20H28FN3O3	377.2109	FCCCCCN1N=C2C=CC=CC2=C1C(=O)N[C@H](C(=O)OC)C(C)(C)C
5-Fluoro ADB 2’-Indazole Isomer Butanoic Acid	C19H26FN3O3	363.1953	FCCCCCN1N=C2C=CC=CC2=C1C(=O)N[C@H](C(=O)O)C(C)(C)C
5-Fluoro ADB-P7AICA	C19H27FN4O2	362.2113	NC([C@H](C(C)(C)C)NC(=O)C1=CN(C2=NC=CC=C21)CCCCCF)=O
5-Fluoro ADB-PINACA	C19H27FN4O2	362.2113	CC(C)(C)C(NC(=O)c1nn(CCCCCF)c2ccccc12)C(N)=O
5-Fluoro ADB-PINACA Isomer 2	C19H27FN4O2	362.2113	O=C(C1=NN(CCCCCF)C2=C1C=CC=C2)N[C@@H]([C@@H](C)CC)C(N)=O
5-Fluoro AMB Metabolite 2	C19H27N3O4	361.1996	CC(C)[C@H](NC(C1=NN(CCCCCO)C2=C1C=CC=C2)=O)C(OC)=O
5-Fluoro AMB Metabolite 3	C19H25N3O5	375.1789	COC(=O)C(NC(=O)c1nn(CCCCC(=O)O)c2ccccc12)C(C)C
5-Fluoro AMB Metabolite 5	C18H25N3O4	347.1840	CC(C)[C@@H](C(O)=O)NC(C1=NN(CCCCCO)C2=C1C=CC=C2)=O
5-Fluoro AMB Metabolite 6	C18H23N3O5	361.1632	C(=O)(O)CCCCN1N=C(C2=CC=CC=C12)C(=O)N[C@@H](C(C)C)C(=O)O
5-Fluoro AMB Metabolite 7	C18H24FN3O3	349.1796	CC(C)[C@@H](C(O)=O)NC(C1=NN(CCCCCF)C2=C1C=CC=C2)=O
5-Fluoro AMT	C11H13FN2	192.1057	FC=1C=C2C(=CNC2=CC1)CC(N)C
5-Fluoro BEPIRAPIM	C25H30FN3O	407.2367	O=C(c1cn(CCCCCF)c2ccccc12)N1CCN(Cc2ccccc2)CC1

**Table S9.** Compound database imported into the *Search Mass Lists* node (continued)			
Compound Name	Molecular Formula	Exact Mass	SMILES
5-Fluoro CUMYL-P7AICA N-Pentanoic Acid Metabolite	C22H25N3O3	379.1890	C1(=CC=CC=C1)C(C)(C)NC(=O)C1=CN(C2=NC=CC=C21)CCCCC(=O)O
5-Fluoro CUMYL-PeGACLONE N-(5-Hydroxypentyl) Metabolite	C25H28N2O2	388.2145	OCCCCCN1C2=C(C=3C=CC=CC13)C(N(C=C2)C(C)(C2=CC=CC=C2)C)=O
5-Fluoro CUMYL-PeGACLONE N-Pentanoic Acid Metabolite	C25H26N2O3	402.1938	O=C1N(C=CC=2N(C=3C=CC=CC3C21)CCCCC(=O)O)C(C)(C)C2=CC=CC=C2
5-Fluoro CYPPICA	C18H23FN2O	302.1789	O=C(C1=CN(CCCCCF)C2=C1C=CC=C2)NCC3CC3
5-Fluoro EMB-PICA N-(5-Hydroxypentyl) Metabolite	C21H30N2O4	374.2200	OCCCCCN1C=C(C2=CC=CC=C12)C(=O)N[C@@H](C(C)C)C(=O)OCC
5-Fluoro Ethylbenzyl-PICA	C23H27FN2O	366.2102	CCC(NC(=O)c1cn(CCCCCF)c2ccccc12)c1ccccc1
5-Fluoro MDMB-7-PAICA	C20H28FN3O3	377.2109	COC(=O)C(NC(=O)c1cn(CCCCCF)c2ncccc12)C(C)(C)C
5-Fluoro MDMB-7-PAICA Butanoic Acid Metabolite	C19H26FN3O3	363.1953	CC(C)(C)C(NC(=O)c1cn(CCCCCF)c2ncccc12)C(=O)O
5-Fluoro MDMB-PICA Metabolite 2	C21H30N2O4	374.2200	COC(=O)C(NC(=O)c1cn(CCCCCO)c2ccccc12)C(C)(C)C
5-Fluoro MDMB-PICA Metabolite 4	C20H27FN2O4	378.1949	CC(C)(C)C(NC(=O)c1cn(CCCC(O)CF)c2ccccc12)C(=O)O
5-Fluoro MDMB-PICA Metabolite 7	C20H27FN2O3	362.2000	FCCCCCN1C=C(C2=CC=CC=C12)C(=O)N[C@H](C(=O)O)C(C)(C)C
5-Fluoro MDMB-PICA Metabolite 8	C19H24N2O5	360.1680	COC(=O)C(NC(=O)c1cn(CCC(=O)O)c2ccccc12)C(C)(C)C
5-Fluoro MDMB-PICA Metabolite 9	C21H29FN2O4	392.2106	COC(=O)C(NC(=O)c1cn(CCCCCF)c2cc(O)ccc12)C(C)(C)C
5-Fluoro MMB-P7AICA	C18H24FN3O3	349.1796	FCCCCN1C=C(C=2C1=NC=CC2)C(=O)N[C@@H](C(C)C)C(=O)OC
5-Fluoro MN-18	C23H22FN3O	375.1741	O=C(C1=NN(CCCCCF)C2=C1C=CC=C2)NC3=C4C=CC=CC4=CC=C3
5-Fluoro MPP-PICA Phenylpropionic Acid Metabolite	C23H25FN2O3	396.1844	O=C(N[C@H](C(O)=O)CC1=CC=CC=C1)C2=CN(CCCCCF)C3=C2C=CC=C3
5-Fluoro NNEI	C24H23FN2O	374.1789	O=C(C1=CN(CCCCCF)C2=C1C=CC=C2)NC3=C4C=CC=CC4=CC=C3
5-Fluoro PB-22 3-Carboxyindole Metabolite	C14H16FNO2	249.1160	O=C(C1=CN(CCCCCF)C2=C1C=CC=C2)O
5-Fluoro Phenyl-PICA	C20H21FN2O	324.1633	O=C(Nc1ccccc1)c1cn(CCCCCF)c2ccccc12
5-Fluoro PY-PICA	C18H23FN2O	302.1789	O=C(C1=CN(CCCCCF)C2=C1C=CC=C2)N3CCCC3
5-Fluoro SDB-005	C23H21FN2O2	376.1582	O=C(Oc1cccc2ccccc12)c1nn(CCCCCF)c2ccccc12
5-Fluoro-2-ADB-PINACA Isomer 2	C19H27FN4O2	362.2113	O=C(C1=C2C=CC=CC2=NN1CCCCCF)N[C@@H]([C@@H](C)CC)C(N)=O
5-Fluoro-3,5-AB-PFUPPYCA	C20H26F2N4O2	392.2018	O=C(C1=CC(C2=CC=C(F)C=C2)=NN1CCCCCF)N[C@@H](C(C)C)C(N)=O
5-Fluoro-3,5-ADB-PFUPPYCA	C21H28F2N4O2	406.2175	CC(C)(C)C(NC(=O)c1cc(-c2ccc(F)cc2)nn1CCCCCF)C(N)=O
5-Fluoro-AKB48 N-(4-Hydroxypentyl) Metabolite	C23H30FN3O2	399.2317	O=C(C1=NN(CCCC(O)CF)C2=C1C=CC=C2)NC3(C4)CC5CC4CC(C5)C3
5-Fluoropentyl-3-pyridinoylindole	C19H19FN2O	310.1476	O=C(C1=CN(CCCCCF)C2=C1C=CC=C2)C3=CC=CN=C3
5-Fluoropentylindole	C13H16FN	205.1261	FCCCCCn1ccc2ccccc21
5-Fluoro-tert-butylbenzyl-PINACA	C24H30FN3O	395.2367	CC(C)(C)C(NC(=O)c1nn(CCCCCF)c2ccccc12)c1ccccc1
5F-MDMB-P4AICA	C20H28FN3O3	377.2109	CC(C)(C)C(NC(C1=CN(CCCCCF)C2=CC=CN=C21)=O)C(OC)=O
5F-MDMB-PINACA COOH Metabolite	C19H26FN3O3	363.1953	CC(C)(C)[C@@H](C(O)=O)NC(C1=NN(CCCCCF)C2=C1C=CC=C2)=O
5F-NNEI-2	C24H23FN2O	374.1789	O=C(C1=CN(CCCCCF)C2=C1C=CC=C2)NC3=CC=C4C=CC=CC4=C3
5F-NPB-22	C22H20FN3O2	377.1534	O=C(C1=NN(CCCCCF)C2=C1C=CC=C2)OC3=C4N=CC=CC4=CC=C3
5F-PB-22	C23H21FN2O2	376.1582	O=C(C1=CN(CCCCCF)C2=C1C=CC=C2)OC3=C4N=CC=CC4=CC=C3
5F-PCN	C23H22FN3O	375.1741	O=C(C1=CN(CCCCCF)C2=C1C=NC=C2)NC3=C4C=CC=CC4=CC=C3
5F-PY-PINACA	C17H22FN3O	303.1741	FCCCCCN1N=C(C(=O)N2CCCC2)C2=CC=CC=C12
5F-SDB-006	C21H23FN2O	338.1789	FCCCCCN1C=C(C(NCC2=CC=CC=C2)=O)C3=C1C=CC=C3
5F-THJ	C22H21FN4O	376.1694	O=C(NC1=C(N=CC=C2)C2=CC=C1)C3=NN(CCCCCF)C4=C3C=CC=C4
5-HTP	C11H12N2O3	220.0842	NC(C(=O)O)CC1=CNC2=CC=C(C(=C12)O
5-Hydroxy Tryptamine	C10H12N2O	176.0944	NCCC1=CNC2=CC=C(C(=C12)O
5-Hydroxy-BZO-POXIZID	C20H21N3O3	351.1577	OCCCCCN1C(C(C2=CC=CC=C12)=NNC(C1=CC=CC=C1)=O)=O
5-Hydroxy-N-methyl Tryptamine	C11H14N2O	190.1101	CNCCC1=CNC2=CC=C(C(=C12)O
5-IAI	C9H10IN	258.9853	NC1CC2=C(C=C(I)C=C2)C1

Table S9. Compound database imported into the *Search Mass Lists* node (continued)

Compound Name	Molecular Formula	Exact Mass	SMILES
5-IT	C11H14N2	174.1152	CC(N)CC1=CC2=C(NC=C2)C=C1
5-MAPB	C12H15NO	189.1148	CC(NC)CC1=CC2=C(OC=C2)C=C1
5-MAPDB	C12H17NO	191.1305	CC(NC)CC1=CC=C(OCC2)C2=C1
5-MAPDI	C13H19N	189.1512	CC(NC)CC1=CC2=C(CCC2)C=C1
5-MBPB	C13H17NO	203.1305	CCC(NC)CC1=CC2=C(OC=C2)C=C1
5MDMB-FUBICA Metabolite 3	C22H23FN2O3	382.1687	CC(C)(C)[C@H](NC(C1=CN(CC2=CC=C(F)C=C2)C3=C1C=CC=C3)=O)C(O)=O
5-MeO-ADMT	C13H18N2O	218.1414	COC=1C=C2C(=CNC2=CC1)CC(C)NC
5-MeO-Amt	C12H16N2O	204.1257	COC1=CC2=C(NC=C2CC(C)N)C=C1
5-MeO-DALT	C17H22N2O	270.1727	C=CCN(CC=C)CCC1=CNC2=C1C=C(OC)C=C2
5-MeO-DiBF	C17H25NO2	275.1880	CC(N(C(C)C)CCC1=COC2=C1C=C(OC)C=C2)C
5-MeO-DIPT	C17H26N2O	274.2040	CC(N(C(C)C)CCC1=CNC2=C1C=C(OC)C=C2)C
5-MeO-DMT	C13H18N2O	218.1414	COC=1C=C2C(=CNC2=CC1)CCN(C)C
5-MeO-MALT	C15H20N2O	244.1570	C=CCN(CCC1=CNC2=C1C=C(OC)C=C2)C
5-MeO-MiPT	C15H22N2O	246.1727	CN(C(C)C)CCC1=CNC2=CC=C(OC)C=C12
5-MeO-MPMI	C15H20N2O	244.1570	COC=1C=C2C(=CNC2=CC1)C[C@@H]1N(CCC1)C
5-MeO-NiPT	C14H20N2O	232.1570	CC(NCCC1=CNC2=C1C=C(OC)C=C2)C
5-MeO-NMT	C12H16N2O	204.1257	COC=1C=C2C(=CNC2=CC1)CCNC
5-MeO-pyr-T	C15H20N2O	244.1570	COC1=CC2=C(NC=C2CCN3CCCC3)C=C1
5-MeO-TMT	C14H20N2O	232.1570	COC=1C=C2C(=C(NC2=CC1)C)CCN(C)C
5-MeO-tryptamine	C11H14N2O	190.1101	NCCC1=CNC2=C1C=C(OC)C=C2
5-Methoxy AET	C13H18N2O	218.1414	NC(CC)CC1=CNC2=CC=C(OC)C=C21
5-Methoxy DET	C15H22N2O	246.1727	COC1=CC2=C(NC=C2CCN(CC)CC)C=C1
5-Methoxy DPT	C17H26N2O	274.2040	CCCN(CCC1=CNC2=C1C=C(OC)C=C2)CCC
5-Methoxy EiPT	C16H24N2O	260.1883	CC(N(CC)CCC1=CNC2=C1C=C(OC)C=C2)C
5-Methoxy EPT	C16H24N2O	260.1883	CCCN(CC)CCC1=CNC2=C1C=C(OC)C=C2
5-Methoxy MET	C14H20N2O	232.1570	CN(CC)CCC1=CNC2=C1C=C(OC)C=C2
5-Methoxy N,N-Diethyl Hexylone	C18H27NO4	321.1935	C(C)N(C(C(=O)C1=CC2=C(OCO2)C(=C1)OC)CCCC)CC
5-Methoxy-Methylone	C12H15NO4	237.0996	CC(NC)C(C1=CC(OC)=C(OCO2)C2=C1)=O
5-Methoxy-N,N-Dibutyl Tryptamine	C19H30N2O	302.2353	C(CCC)N(CCCC)CCC1=CNC2=CC=C(C=C12)OC
5-Methoxy-N,N-Diethylpentylone	C17H25NO4	307.1778	CCCC(N(CC)CC)C(C1=CC(OC)=C(OCO2)C2=C1)=O
5-Methoxy-N,N-Diisobutyl Tryptamine	C19H30N2O	302.2353	C(C(C)C)N(CC(C)C)CCC1=CNC2=CC=C(C=C12)OC
5-Methyl Etodesnitazene	C23H31N3O	365.2462	C(C)OC1=CC=C(C=C1)CC1=NC2=C(N1CCN(CC)CC)C=CC(=C2)C
5-Methyl-MDA	C11H15NO2	193.1097	CC(N)CC1=CC(C)=C(OCO2)C2=C1
5-PPDi	C17H23NO	257.1774	CCC(N1CCCC1)C(C2=CC3=C(CCC3)C=C2)=O
6-(2-Aminopropyl)indole	C11H14N2	174.1152	CC(N)CC1=CC2=C(C=C1)C=CN2
6-Acetylcodeine	C20H23NO4	341.1622	[H][C@@]12OC3=C(OC)C=CC4=C3[C@]11CCN(C)[C@]([H])(C4)[C@]1([H])C=C[C@]2([H])OC(C)=O
6-APB	C11H13NO	175.0992	CC(N)CC1=CC2=C(C=C1)C=CO2
6-APDB	C11H15NO	177.1148	CC(N)CC1=CC2=C(C=C1)CCO2
6-Bromo-MDMA	C11H14BrNO2	271.0203	CC(NC)CC1=C(Br)C=C(OCO2)C2=C1
6-EAPB	C13H17NO	203.1305	O1C=CC2=C1C=C(C=C2)CC(C)NCC
6-Fluoro DET	C14H19FN2	234.1527	CCN(CC)CCC1=CNC2=CC(F)=CC=C21
6-MAPB	C12H15NO	189.1148	O1C=CC2=C1C=C(C=C2)CC(C)NC

**Table S9.** Compound database imported into the *Search Mass Lists* node (continued)			
Compound Name	Molecular Formula	Exact Mass	SMILES
6-Methoxy DiPT	C17H26N2O	274.2040	CC(N(CCC1=CNC2=C1C=CC(OC)=C2)C(C)C)C
6-Methoxy DMT	C13H18N2O	218.1414	COC=1C=C2NC=C(CCN(C)C)C2=CC1
6-Methoxy-N,N-diethylpentylone	C17H25NO4	307.1778	C(C)N(C(C(=O)C1=CC2=C(OCO2)C=C1OC)CCC)CC
6-Methyl-MDA	C11H15NO2	193.1097	CC=1C(=CC2=C(OCO2)C1)CC(C)N
7-Aminoclonazepam	C15H12ClN3O	285.0663	C1C(=O)NC2=C(C=C(C=C2)N)C(=N1)C3=CC=CC=C3Cl
7-Aminoflunitrazepam	C16H14FN3O	283.1115	NC=1C=CC2=C(C(=NCC(N2C)=O)C2=C(C=CC=C2)F)C1
7-Aminonitrazepam	C15H13N3O	251.1053	C1C(=O)NC2=C(C=C(C=C2)N)C(=N1)C3=CC=CC=C3
7-APB	C11H13NO	175.0992	NC(CC1=CC=CC=2C=COC21)C
7-APDB	C11H15NO	177.1148	NC(CC1=CC=CC=2CCOC21)C
7-Fluoro Tryptamine	C10H11FN2	178.0901	FC=1C=CC=C2C(=CNC12)CCN
7-Hydroxymitragynine	C23H30N2O5	414.2149	O=C(OC)/C([C@@H](C[C@]12[H])[C@H](CC)CN1CC[C@@]3(O)C2=NC4=C3C(OC)=CC=C4)=C/OC
7-Methoxy DiPT	C17H26N2O	274.2040	CC(N(CCC1=CNC2=C1C=CC=C2OC)C(C)C)C
7’-Methoxy NABUTIE	C25H25NO2	371.1880	CCCCn1cc(C(=O)Cc2cccc3ccccc23)c2cccc(OC)c21
8-Aminoflunitrazolam	C17H14FN5	307.1228	FC1=C(C=CC=C1)C1=NCC=2N(C3=C1C=C(C=C3)N)C(=NN2)C
8-Chlorotheophylline	C7H7ClN4O2	214.0252	ClC1=NC=2N(C(N(C(C2N1)=O)C)=O)C
A03	C16H23FN2O	278.1789	FC1=CC=C(C(=O)NCC2(CCCCC2)N(C)C)C=C1
A04	C17H23F3N2O	328.1757	FC(C1=CC=C(C(=O)NCC2(CCCCC2)N(C)C)C=C1)(F)F
A05	C17H26N2O2	290.1989	COC1=CC=C(C(=O)NCC2(CCCCC2)N(C)C)C=C1
A06	C16H22F2N2O	296.1695	FC=1C=C(C(=O)NCC2(CCCCC2)N(C)C)C=CC1F
A07	C15H22Cl2N2O2S	364.0774	ClC=1C=C(C=CC1Cl)S(=O)(=O)NCC1(CCCCC1)N(C)C
A08	C16H23BrN2O	338.0988	BrC1=CC=C(C(=O)NCC2(CCCCC2)N(C)C)C=C1
A09	C15H22ClN3O	295.1446	ClC1=C(C(=O)NCC2(CCCCC2)N(C)C)C=CC=N1
A10	C20H26N2O	310.2040	C1(=CC=CC2=CC=CC=C12)C(=O)NCC1(CCCCC1)N(C)C
A11	C15H23N3O	261.1836	C(C1=CN=CC=C1)(=O)NCC1(CCCCC1)N(C)C
A12	C15H23N3O	261.1836	C(C1=CC=NC=C1)(=O)NCC1(CCCCC1)N(C)C
A14	C20H34N2O	318.2666	C12(CC3CC(CC(C1)C3)C2)C(=O)NCC2(CCCCC2)N(C)C
A16	C16H23FN2O	278.1789	FC=1C=C(C(=O)NCC2(CCCCC2)N(C)C)C=CC1
A-3665	C25H31FN6O2	466.2487	C(C)N1N=NN(C1=O)CCN1CCC(CC1)(C1=CC=CC=C1)N(C(CC)=O)C1=C(C=CC=C1)F
A-796,260	C22H30N2O2	354.2302	O=C(C1=CN(CCN2CCOCC2)C3=C1C=CC=C3)C4C(C)(C)C4(C)C
A-796260 Degradant	C22H30N2O2	354.2302	C=C(C)C(C)(C)CC(C1=CN(CCN2CCOCC2)C3=C1C=CC=C3)=O
A-834735	C22H29NO2	339.2193	CC1(C)C(C(=O)C2=CN(CC3CCOCC3)C3=CC=CC=C23)C1(C)C
A-834735 Degradant	C22H29NO2	339.2193	C=C(C)C(C)(C)CC(C1=CN(CC2CCOCC2)C3=C1C=CC=C3)=O
A-836339	C16H26N2O2S	310.1710	O=C(C1C(C)(C)C1(C)C)/N=C2SC(C)=C(C)N\2CCOC
AB-001	C24H31NO	349.2400	O=C(C1=CN(CCCCC)C2=C1C=CC=C2)C34CC5CC(C4)CC(C5)C3
AB-005	C23H32N2O	352.2509	O=C(C1=CN(CC2N(C)CCCC2)C3=C1C=CC=C3)C4C(C)(C)C4(C)C
AB-005 Azepane Isomer 2	C23H32N2O	352.2509	O=C(C1=CN(C2N(C)CCCCC2)C3=C1C=CC=C3)C4C(C)(C)C4(C)C
AB-005-Azepan	C23H32N2O	352.2509	O=C(C1=CN(C2CN(C)CCCC2)C3=C1C=CC=C3)C4C(C)(C)C4(C)C
AB-7-FUBAICA	C20H21FN4O2	368.1643	CC(C)C(NC(=O)c1cn(Cc2ccc(F)cc2)c2ncccc12)C(N)=O
AB-BICA	C21H23N3O2	349.1785	O=C(C1=CN(CC2=CC=CC=C2)C3=C1C=CC=C3)N[C@@H](C(C)C)C(N)=O
AB-CHFUPYCA	C22H29FN4O2	400.2269	CC(C)[C@@H](C(N)=O)NC(C1=CC(C2=CC=C(F)C=C2)=NN1CC3CCCCC3)=O
AB-CHMICA	C21H29N3O2	355.2254	O=C(C1=CN(CC2CCCCC2)C3=C1C=CC=C3)N[C@@H](C(C)C)C(N)=O
AB-CHMINACA	C20H28N4O2	356.2207	O=C(C1=NN(CC2CCCCC2)C3=C1C=CC=C3)N[C@@H](C(C)C)C(N)=O

**Table S9.** Compound database imported into the *Search Mass Lists* node (continued)			
Compound Name	Molecular Formula	Exact Mass	SMILES
AB-CHMINACA (M1B(3-OH-Cyclohexyl))	C20H28N4O3	372.2156	O=C(C1=NN(CC2CC(O)CCC2)C3=C1C=CC=C3)N[C@@H](C(C)C)C(N)=O
AB-CHMINACA (M5A (3-Carboxyindole-4-OH-cyclohexyl))	C15H18N2O3	274.1312	O=C(C1=NN(CC2CCC(O)CC2)C3=C1C=CC=C3)O
AB-CHMINACA 2'-Indazole Isomer	C20H28N4O2	356.2207	O=C(N[C@H](C(N)=O)C(C)C)C1=C2C(C=CC=C2)=NN1CC3CCCCC3
AB-CHMINACA Metabolite M1A	C20H28N4O3	372.2156	O=C(C1=NN(CC2CCC(O)CC2)C3=C1C=CC=C3)N[C@@H](C(C)C)C(N)=O
AB-CHMINACA Metabolite M2	C20H27N3O3	357.2047	CC(C)[C@@H](C(O)=O)NC(C1=NN(CC2CCCCC2)C3=C1C=CC=C3)=O
AB-CHMINACA Metabolite M3A	C20H27N3O4	373.1996	CC(C)[C@@H](C(O)=O)NC(C1=NN(CC2CCC(O)CC2)C3=C1C=CC=C3)=O
AB-CHMINACA Metabolite M4	C15H18N2O2	258.1363	O=C(C1=NN(CC2CCCCC2)C3=C1C=CC=C3)O
AB-CHMINACA Metabolite M6	C20H26N4O4	386.1948	O=C(O)C(C)C(NC(C1=NN(CC2CCCCC2)C3=C1C=CC=C3)=O)C(N)=O
AB-CHMINACA Metabolite M7	C20H25N3O5	387.1789	CC(C(=O)O)C(NC(=O)c1nn(CC2CCCCC2)c2ccccc12)C(=O)O
AB-FUBICA	C21H22FN3O2	367.1691	O=C(N[C@H](C(N)=O)C(C)C)C1=CN(CC2=CC=C(F)C=C2)C3=CC=CC=C31
AB-FUBINACA	C20H21FN4O2	368.1643	CC(C)C(NC(=O)c1nn(Cc2ccc(F)cc2)c2ccccc12)C(N)=O
AB-FUBINACA Metabolite 2A	C20H19FN4O4	398.1385	O=C(O)C(C)C(NC(C1=NN(CC2=CC=C(F)C=C2)C3=C1C=CC=C3)=O)C(N)=O
AB-FUBINACA Metabolite 3	C20H20FN3O3	369.1483	CC(C)[C@@H](C(O)=O)NC(C1=NN(CC2=CC=C(F)C=C2)C3=C1C=CC=C3)=O
AB-FUBINACA Metabolite 4	C15H11FN2O2	270.0799	O=C(O)c1nn(Cc2ccc(F)cc2)c2ccccc12
AB-FUBINACA-2-fluorbenzyl	C20H21FN4O2	368.1643	O=C(C1=NN(CC2=CC=CC=C2F)C3=C1C=CC=C3)N[C@@H](C(C)C)C(N)=O
AB-FUBINACA-3-fluorbenzyl	C20H21FN4O2	368.1643	NC([C@H](C(C)C)NC(=O)C1=NN(C2=CC=CC=C12)CC1=CC(=CC=C1)F)=O
ABO-4en-PINACA	C17H22N4O2	314.1737	NC(C(CC)NC(=O)C1=NN(C2=CC=CC=C12)CCCC=C)=O
AB-PICA	C19H27N3O2	329.2098	NC(=O)[C@H](C(C)C)NC(=O)C1=CN(C2=CC=CC=C12)CCCCC
AB-PINACA	C18H26N4O2	330.2050	O=C(C1=NN(CCCCC)C2=C1C=CC=C2)N[C@H](C(N)=O)C(C)C
AB-PINACA (5-OH-Pentyl)	C18H26N4O3	346.1999	NC(C(C(C)C)NC(=O)C1=NN(C2=CC=CC=C12)CCCCCO)=O
AB-PINACA 3-Carboxyindazole Metabolite	C13H16N2O2	232.1206	O=C(O)C1=NN(CCCCC)C2=C1C=CC=C2
AB-PINACA Carboxylic Acid	C18H25N3O3	331.1890	CC([C@@H](C(=O)O)NC(=O)C1=NN(C2=CC=CC=C12)CCCCC)C
AB-PINACA N-(2-Fluoropentyl) Isomer	C18H25FN4O2	348.1956	O=C(C1=NN(CC(F)CCC)C2=C1C=CC=C2)NC(C(C)C)C(N)=O
AB-PINACA N-(4-Hydroxypentyl) Metabolite	C18H26N4O3	346.1999	CC(O)CCCn1nc(C(=O)NC(C(N)=O)C(C)C)c2ccccc21
AB-PINACA Pentanoic Acid	C18H24N4O4	360.1792	O=C(O)CCCCN1N=C(C(NC(C(N)=O)C(C)C)=O)C2=C1C=CC=C2
Acefylline	C9H10N4O4	238.0696	CN1C(N(C=2N=CN(C2C1=O)CC(=O)O)C)=O
Acetildenafil	C25H34N6O3	466.2687	O=C1C(N(C)N=C2CCC)=C2N=C(C3=CC(C(CN4CCN(CC)CC4)=O)=CC=C3OCC)N1
Acetylamphetamine	C11H15NO	177.1148	CC(NC(C)CC1=CC=CC=C1)=O
Acetylbenzylfentanyl	C20H24N2O	308.1883	CC(N(C1CCN(CC2=CC=CC=C2)CC1)C3=CC=CC=C3)=O
Acetyl-carfentanil	C23H28N2O3	380.2094	C(C)(=O)N(C1(CCN(CC1)CCC1=CC=CC=C1)C(=O)OC)C1=CC=CC=C1
Aconitine	C34H47NO11	645.3143	CCN1CC2(C(CC(C34C2C(C(C31)C5(C6C4CC(C6OC(=O)C7=CC=CC=C7)(C(C5O)OC)O)OC(=O)C)OC)OC)O)COC
ADB-4en-P-5Br-INACA	C19H25BrN4O2	420.1155	NC(C(C(C)(C)C)NC(=O)C1=NN(C2=CC=C(C=C12)Br)CCCC=C)=O
ADB-4en-PINACA	C19H26N4O2	342.2050	O=C(NC(C(C)(C)C)C(N)=O)C1=NN(CCCC=C)C2=C1C=CC=C2
ADB-5'Br-BINACA	C18H25BrN4O2	408.1155	BrC=1C=C2C(=NN(C2=CC1)CCCC)C(=O)NC(C(C)(C)C)C(N)=O
ADB-5'Br-DECINACA	C24H37BrN4O2	492.2094	NC(C(C(C)(C)C)NC(=O)C1=NN(C2=CC=C(C=C12)Br)CCCCCCCCCC)=O
ADB-5Br-INACA	C14H17BrN4O2	352.0529	BrC=1C=C2C(=NNC2=CC1)C(=O)NC(C(C)(C)C)C(N)=O
ADB-BICA	C22H25N3O2	363.1941	CC(C)(C)C(NC(C1=CN(CC2=CC=CC=C2)C3=C1C=CC=C3)=O)C(N)=O
ADB-BINACA	C21H24N4O2	364.1894	O=C(C1=NN(CC2=CC=CC=C2)C3=C1C=CC=C3)NC(C(C)(C)C)C(N)=O
ADB-BUT-5'F-INACA	C18H25FN4O2	348.1956	NC(C(C(C)(C)C)NC(=O)C1=NN(C2=CC=C(C=C12)F)CCCC)=O
ADB-BUTINACA	C18H26N4O2	330.2050	CCCCn1nc(C(=O)NC(C(N)=O)C(C)(C)C)c2ccccc21
ADB-BUTINACA N-(4-Hydroxybutyl) Metabolite	C18H26N4O3	346.1999	NC([C@H](C(C)(C)C)NC(=O)C1=NN(C2=CC=CC=C12)CCCCO)=O
ADB-BUTINACA N-Butanoic Acid Metabolite	C18H24N4O4	360.1792	NC([C@H](C(C)(C)C)NC(=O)C1=NN(C2=CC=CC=C12)CCCC(=O)O)=O

Table S9. Compound database imported into the *Search Mass Lists* node (continued)			
Compound Name	Molecular Formula	Exact Mass	SMILES
ADB-BUTINAATA	C19H28N4O2	344.2207	C(CCC)N1N=C(C2=CC=CC=C12)CC(=O)N[C@H](C(=O)N)C(C)(C)C
ADB-CHMICA	C22H31N3O2	369.2411	O=C(C1=CN(CC2CCCCC2)C3=C1C=CC=C3)NC(C(C)(C)C)C(N)=O
ADB-CHMINACA (M1(4-OH-Cyclohexyl))	C21H30N4O3	386.2312	O=C(C1=NN(CC2CCC(O)CC2)C3=C1C=CC=C3)NC(C(C)(C)C)C(N)=O
ADB-CHMINACA (M10(Lacton)	C21H27N3O3	369.2047	O=C(C1=NN(CC2CCCCC2)C3=C1C=CC=C3)NC4C(OCC4(C)C)=O
ADB-CHMINACA (M11(OH-t-Butyl)	C21H30N4O4	402.2261	O=C(C1=NN(CC2CCC(O)CC2)C3=C1C=CC=C3)NC(C(C)(C)CO)C(N)=O
ADB-CHMINACA (M2(Valin))	C21H29N3O3	371.2203	C1(CCCCC1)CN1N=C(C2=CC=CC=C12)C(=O)N[C@H](C(=O)O)C(C)(C)C
ADB-CHMINACA (M3(Valin-4-OH-cyclohexyl))	C21H29N3O4	387.2153	CC(C)(C)[C@H](NC(C1=NN(CC2CCC(O)CC2)C3=C1C=CC=C3)=O)C(O)=O
ADB-FUBHQUCA	C23H26FN3O2	395.2004	NC(C(C(C)(C)C)NC(=O)C1=CN(C2=CC=CC=C2C1)CC1=CC=C(C=C1)F)=O
ADB-FUBIATA	C23H26FN3O2	395.2004	FC1=CC=C(C=C1)CN1C=C(C2=CC=CC=C12)CC(=O)NC(C(=O)N)C(C)(C)C
ADB-FUBIATA 2-Hydroxybenzyl Metabolite	C23H26FN3O3	411.1953	FC1=CC(=C(CN2C=C(C3=CC=CC=C23)CC(=O)NC(C(=O)N)C(C)(C)C)C=C1)O
ADB-FUBIATA 6-Hydroxyindole Metabolite	C23H26FN3O4	427.1902	FC1=CC(=C(CN2C=C(C3=CC=C(C=C23)O)CC(=O)NC(C(=O)N)C(C)(C)C)C=C1)O
ADB-FUBICA	C22H24FN3O2	381.1847	O=C(C1=CN(CC2=CC=C(F)C=C2)C3=C1C=CC=C3)NC(C(C)(C)C)C(N)=O
ADB-FUBINACA	C21H23FN4O2	382.1800	O=C(NC(C(N)=O)C(C)(C)C)C1=NN(CC2=CC=C(F)C=C2)C3=C1C=CC=C3
ADB-HEXINACA	C20H30N4O2	358.2363	C(N)(=O)C(C(C)(C)C)NC(=O)C1=NN(C2=CC=CC=C12)CCCCCC
ADB-HEXINACA 3,3-Dimethylbutanoic Acid	C20H29N3O3	359.2203	C(CCCCC)N1N=C(C2=CC=CC=C12)C(=O)N[C@H](C(=O)O)C(C)(C)C
ADB-HEXINACA N-(6-Hydroxyhexyl) Metabolite	C20H30N4O3	374.2312	NC([C@H](C(C)(C)C)NC(=O)C1=NN(C2=CC=CC=C12)CCCCCCO)=O
ADB-HEXINACA N-Hexanoic Acid Metabolite	C20H28N4O4	388.2105	NC([C@H](C(C)(C)C)NC(=O)C1=NN(C2=CC=CC=C12)CCCCCC(=O)O)=O
ADB-IATA	C16H21N3O2	287.1628	N1C=C(C2=CC=CC=C12)CC(=O)NC(C(=O)N)C(C)(C)C
ADBICA	C20H29N3O2	343.2254	O=C(C1=CN(CCCCC)C2=C1C=CC=C2)NC(C(C)(C)C)C(N)=O
ADBICA (N-5-OH-pentyl)	C20H29N3O3	359.2203	NC(C(C(C)(C)C)NC(=O)C1=CN(C2=CC=CC=C12)CCCCCO)=O
ADBICA N-(4-Hydroxypentyl) Metabolite	C20H29N3O3	359.2203	CC(O)CCCn1cc(C(=O)NC(C(N)=O)C(C)(C)C)c2ccccc21
ADBICA N-Pentanoic Acid	C20H27N3O4	373.1996	O=C(O)CCCCN1C=C(C(NC(C(C)(C)C)C(N)=O)=O)C2=C1C=CC=C2
ADB-INACA	C14H18N4O2	274.1424	C(N)(=O)C(C(C)(C)C)NC(=O)C1=NNC2=CC=CC=C12
ADB-P-5Br-INACA	C19H27BrN4O2	422.1312	NC(C(C(C)(C)C)NC(=O)C1=NN(C2=CC=C(C=C12)Br)CCCCC)=O
ADB-P7AICA	C19H28N4O2	344.2207	O=C(N[C@@H](C(C)(C)C)C(N)=O)C1=CN(CCCCC)C2=C1C=CC=N2
ADB-PHETINACA	C22H26N4O2	378.2050	C(N)(=O)C(C(C)(C)C)NC(=O)C1=NN(C2=CC=CC=C12)CCC1=CC=CC=C1
ADB-PINACA	C19H28N4O2	344.2207	O=C(C1=NN(CCCCC)C2=C1C=CC=C2)NC(C(C)(C)C)C(N)=O
ADB-PINACA (N-5-OH-Pentyl)	C19H28N4O3	360.2156	NC(C(C(C)(C)C)NC(=O)C1=NN(C2=CC=CC=C12)CCCCCO)=O
ADB-PINACA N-(4-Hydroxypentyl) Metabolite	C19H28N4O3	360.2156	CC(O)CCCn1nc(C(=O)NC(C(N)=O)C(C)(C)C)c2ccccc21
ADB-PINACA Pentanoic Acid Metabolite	C19H26N4O4	374.1948	CC(C)(C)C(NC(=O)c1nn(CCCCC(=O)O)c2ccccc12)C(N)=O
ADB-PINACA-Isomer-1	C19H28N4O2	344.2207	O=C(C1=NN(CCCCC)C2=C1C=CC=C2)NC(C(C)C)(C)C(N)=O
ADB-PINACA-Isomer-2	C19H28N4O2	344.2207	O=C(C1=NN(CCCCC)C2=C1C=CC=C2)N[C@@H]([C@@H](C)CC)C(N)=O
ADB-PINACA-Isomer-3	C19H28N4O2	344.2207	O=C(C1=NN(CCCCC)C2=C1C=CC=C2)N[C@@H](CCCC)C(N)=O
Adrafinil	C15H15NO3S	289.0767	ONC(=O)CS(=O)C(c1ccccc1)c1ccccc1
AEP-PINACA	C18H26N4O2	330.2050	C(CCCC)N1N=C(C2=CC=CC=C12)C(=O)N[C@H](C(=O)N)CCC
Afloqualone	C16H14FN3O	283.1115	O=C1N(C2=CC=CC=C2C)C(CF)=NC3=C1C=C(N)C=C3
A-FUB7AICA	C25H26FN3O	403.2054	C12(CC3CC(CC(C1)C3)C2)NC(=O)C2=CN(C3=NC=CC=C32)CC3=CC=C(C=C3)F
AFUB7AICA 7'-Azaindole Isomer	C25H26FN3O	403.2054	FC1=CC=C(C=C1)CN1C=2C(=CC=C1)C(=CN2)C(=O)NC21CC3CC(CC(C2)C3)C1
AFUBIATA	C27H29FN2O	416.2259	O=C(NC1(C[C@H](C2)C3)C[C@H]3C[C@H]2C1)CC4=CN(CC5=CC=C(F)C=C5)C6=C4C=CC=C6
AH-7563	C16H24N2O	260.1883	CN(C1(CCCCC1)CNC(C1=CC=CC=C1)=O)C
AH-7959	C19H26Cl2N2O	368.1417	ClC=1C=C(C(=O)NCC2(CCCCC2)N2CCCCC2)C=CC1Cl
AH-8507	C19H27Cl2N3O	383.1526	ClC=1C=C(C(=O)NCC2(CCCCC2)N2CCN(CC2)C)C=CC1Cl

**Table S9.** Compound database imported into the *Search Mass Lists* node (continued)			
Compound Name	Molecular Formula	Exact Mass	SMILES
AH-8529	C16H23ClN2O	294.1494	ClC1=CC=C(C(=O)NCC2(CCCCC2)N(C)C)C=C1
AH-8532	C16H23ClN2O	294.1494	ClC=1C=C(C(=O)NCC2(CCCCC2)N(C)C)C=CC1
AH-8533	C16H23ClN2O	294.1494	ClC1=C(C(=O)NCC2(CCCCC2)N(C)C)C=CC=C1
AKB48	C23H31N3O	365.2462	O=C(C1=NN(CCCCC)C2=C1C=CC=C2)NC34CC5CC(C4)CC(C5)C3
AKB48 N-(4-Fluorobenzyl) Analog	C25H26FN3O	403.2054	O=C(C1=NN(CC2=CC=C(F)C=C2)C3=C1C=CC=C3)NC45C[C@H]6C[C@](C5)([H])C[C@@](C6)([H])C4
AKB48 N-(4-Hydroxypentyl) Metabolite	C23H31N3O2	381.2411	O=C(C1=NN(CCCC(O)C)C2=C1C=CC=C2)NC3(C4)CC5CC4CC(C5)C3
AKB48 N-(5-Hydroxypentyl) Metabolite	C23H31N3O2	381.2411	C12(CC3CC(CC(C1)C3)C2)NC(=O)C2=NN(C3=CC=CC=C23)CCCCCO
AKB48 N-Pentanoic Acid Metabolite	C23H29N3O3	395.2203	O=C(O)CCCCn1nc(C(=O)NC23CC4CC(CC(C4)C2)C3)c2ccccc21
AKB-57	C23H30N2O2	366.2302	O=C(C1=NN(CCCCC)C2=C1C=CC=C2)OC34CC5CC(C4)CC(C5)C3
AKB-N1	C25H34N4O	406.2727	O=C(C1=NN(CCN2CCCCC2)C3=C1C=CC=C3)NC45CC6CC(C5)CC(C6)C4
ALD-52	C22H27N3O2	365.2098	O=C([C@H](C=C12)CN(C)[C@]2([H])CC3=CN(C(C)=O)C4=C3C1=CC=C4)N(CC)CC
ALEPH-2	C13H21NO2S	255.1288	COC1=C(CC(N)C)C=C(C(=C1)SCC)OC
ALEPH-4	C14H23NO2S	269.1444	COC1=C(CC(N)C)C=C(C(=C1)SC(C)C)OC
AL-LAD	C22H27N3O	349.2149	[H][C@@]1(CN(CC=C)[C@]2([H])CC3=CNC4=CC=CC(=C34)C2=C1)C(=O)N(CC)CC
Allylescaline	C13H19NO3	237.1360	NCCC1=CC(OC)=C(OCC=C)C(OC)=C1
alpha,3-Dimethylfentanyl	C24H32N2O	364.2509	CC1CN(CCC1N(C(CC)=O)C1=CC=CC=C1)C(CC1=CC=CC=C1)C
alpha-D2PV	C18H19NO	265.1461	C1(=CC=CC=C1)C(C(N1CCCC1)C1=CC=CC=C1)=O
alpha-Dimethylaminohexanophenone	C14H21NO	219.1618	CN(C(C(=O)C1=CC=CC=C1)CCCC)C
alpha-Ethyl Tryptamine	C12H16N2	188.1308	CCC(N)CC1=CNC2=C1C=CC=C2
alpha-Ethylamino-3,3-dimethylbutyrophenone	C14H21NO	219.1618	CC(C)(C)C(NCC)C(C1=CC=CC=C1)=O
alpha-Hydroxy Clonazolam	C17H12ClN5O3	369.0623	ClC1=C(C=CC=C1)C1=NCC=2N(C3=C1C=C(C=C3)[N+](=O)[O-])C(=NN2)CO
alpha-Hydroxy Flubromazolam	C17H12BrFN4O	386.0173	BrC=1C=CC2=C(C(=NCC=3N2C(=NN3)CO)C3=C(C=CC=C3)F)C1
alpha-Hydroxy Midazolam	C18H13ClFN3O	341.0726	C1C2=CN=C(N2C3=C(C=C(C=C3)Cl)C(=N1)C4=CC=CC=C4F)CO
alpha-Hydroxyetizolam	C17H15ClN4OS	358.0650	ClC1=C(C=CC=C1)C=1C=2C=C(SC2N2C(=NN=C2CN1)C)C(C)O
alpha-Hydroxyflualprazolam	C17H12ClFN4O	342.0678	ClC1=CC=2C(=NCC3=NN=C(N3C2C=C1)CO)C1=C(C=CC=C1)F
Alpha-Hydroxytriazolam	C17H12Cl2N4O	358.0383	C1C2=NN=C(N2C3=C(C=C(C=C3)Cl)C(=N1)C4=CC=CC=C4Cl)CO
alpha-Isobutylaminohexanophenone	C16H25NO	247.1931	CCCCC(NCC(C)C)C(C1=CC=CC=C1)=O
alpha-Methyl Butyryl Fentanyl	C24H32N2O	364.2509	CCCC(N(C1=CC=CC=C1)C2CCN(C(C)CC3=CC=CC=C3)CC2)=O
alpha-Methyl-acrylfentanyl	C23H28N2O	348.2196	C1(=CC=CC=C1)N(C(C=C)=O)C1CCN(CC1)C(CC1=CC=CC=C1)C
alpha-Methylephedrine	C11H17NO	179.1305	CNC(C(C)(O)C1=CC=CC=C1)C
Alpha-Methylfentanyl Butanamide Analogue	C24H32N2O	364.2509	CC(C)C(N(C1=CC=CC=C1)C2CCN(C(C)CC3=CC=CC=C3)CC2)=O
alpha-Methyl-p-fluorofentanyl	C23H29FN2O	368.2259	FC1=CC=C(C=C1)N(C(CC)=O)C1CCN(CC1)C(CC1=CC=CC=C1)C
alpha-PBP	C14H19NO	217.1461	C1(=CC=CC=C1)C(C(CC)N1CCCC1)=O
alpha-PBT	C12H17NOS	223.1026	CCC(N1CCCC1)C(C2=CC=CS2)=O
alpha-PCYP	C18H25NO	271.1931	C1(CCCCC1)C(C(=O)C1=CC=CC=C1)N1CCCC1
alpha-PHiP	C16H23NO	245.1774	CC(C)CC(N1CCCC1)C(C2=CC=CC=C2)=O
alpha-PPP	C13H17NO	203.1305	C1(=CC=CC=C1)C(C(C)N1CCCC1)=O
alpha-PPP-MeO	C14H19NO2	233.1410	O=C(C1=CC=CC=C1)C(N2CCCC2)COC
alpha-PVT	C13H19NOS	237.1182	CCCC(N1CCCC1)C(C2=CC=CS2)=O
alpha-TMT	C13H18N2	202.1465	CC(N(C)C)CC1=CNC2=C1C=CC=C2
Alprazolam Triazolobenzophenone Serivative	C17H15ClN4O	326.0929	O=C(C1=CC(Cl)=CC=C1N2C(CN)=NN=C2C)C3=CC=CC=C3
AM-1220	C26H26N2O	382.2040	O=C(C1=CN(C[C@@H]2N(C)CCCC2)C3=C1C=CC=C3)C4=C5C=CC=CC5=CC=C4

**Table S9.** Compound database imported into the *Search Mass Lists* node (continued)			
Compound Name	Molecular Formula	Exact Mass	SMILES
AM1220 Azepane Isomer	C26H26N2O	382.2040	O=C(C1=CN(C2CN(C)CCCC2)C3=C1C=CC=C3)C4=C5C=CC=CC5=CC=C4
AM1235	C24H21FN2O3	404.1531	FCCCCCN1C=C(C(=O)C2=CC=CC3=CC=CC=C23)C2=C1C=C(C=C2)N(=O)=O
AM-1241	C22H22IN3O3	503.0700	CN1CCCCC1CN1C=C(C(=O)C2=C(I)C=CC(=C2)N(=O)=O)C2=CC=CC=C12
AM-1248	C26H34N2O	390.2666	O=C(C12CC3CC(C2)CC(C3)C1)C4=CN(CC5N(C)CCCC5)C6=C4C=CC=C6
AM-1248-Azepane	C26H34N2O	390.2666	O=C(C12CC3CC(C2)CC(C3)C1)C4=CN(C5CN(C)CCCC5)C6=C4C=CC=C6
AM-2201	C24H22FNO	359.1680	O=C(C1=CN(CCCCCF)C2=C1C=CC=C2)C3=C4C=CC=CC4=CC=C3
AM-2201 (2-OH-Indole)	C24H22FNO2	375.1629	FCCCCCN1C(=C(C2=CC=CC=C12)C(=O)C1=CC=CC2=CC=CC=C12)O
AM-2201 (5-OH-Indole)	C24H22FNO2	375.1629	O=C(C1=CN(CCCCCF)C2=C1C=C(O)C=C2)C3=C4C=CC=CC4=CC=C3
AM-2201 (6-OH-Indole)	C24H22FNO2	375.1629	FCCCCCN1C=C(C2=CC=C(C=C12)O)C(=O)C1=CC=CC2=CC=CC=C12
AM-2201 7-Hydroxyindole Metabolite	C24H22FNO2	375.1629	FCCCCCN1C=C(C2=CC=CC(=C12)O)C(=O)C1=CC=CC2=CC=CC=C12
AM-2201 8-Quinolinyl Carboxamide	C23H22FN3O	375.1741	O=C(C1=CN(CCCCCF)C2=C1C=CC=C2)NC3=C4N=CC=CC4=CC=C3
AM-2201 N-(4-Hydroxypentyl)	C24H22FNO2	375.1629	O=C(C1=CN(CCCC(O)CF)C2=C1C=CC=C2)C3=C4C=CC=CC4=CC=C3
AM-2201-4-Methoxynaphthyl-analogue	C25H24FNO2	389.1786	O=C(C1=CN(CCCCCF)C2=C1C=CC=C2)C3=C4C=CC=CC4=C(OC)C=C3
AM-2232	C24H20N2O	352.1570	N#CCCCCN1C=C(C(C2=C3C=CC=CC3=CC=C2)=O)C4=C1C=CC=C4
AM-2233	C22H23IN2O	458.0850	O=C(C1=CC=CC=C1I)C2=CN(CC3N(C)CCCC3)C4=C2C=CC=C4
AM-2233-Azepane	C22H23IN2O	458.0850	IC1=C(C=CC=C1)C(=O)C1=CN(C2=CC=CC=C12)C1CN(CCCC1)C
AM-3102	C21H41NO2	339.3132	OC[C@@H](C)NC(CCCCCCC/C=C\CCCCCCCC)=O
AM-630	C23H25IN2O3	504.0904	O=C(C1=C(C)N(CCN2CCOCC2)C3=C1C=CC(I)=C3)C4=CC=C(OC)C=C4
AM-679	C20H20INO	417.0584	O=C(C1=CC=CC=C1I)C2=CN(CCCCC)C3=C2C=CC=C3
AM-694	C20H19FINO	435.0490	O=C(C1=CN(CCCCCF)C2=C1C=CC=C2)C3=CC=CC=C3I
AM-694 Chloro Derivative	C20H19ClINO	451.0195	O=C(C1=CN(CCCCCCl)C2=C1C=CC=C2)C3=CC=CC=C3I
AM-694 Ethyl Substituted for Iodine	C22H24FNO	337.1837	O=C(C1=CC=CC=C1CC)C2=CN(CCCCCF)C3=C2C=CC=C3
AM-694 Methyl Substituted for Iodine	C21H22FNO	323.1680	O=C(C1=CN(CCCCCF)C2=C1C=CC=C2)C3=CC=CC=C3C
AM694 N-(5-Hydroxypentyl) Metabolite	C20H20INO2	433.0533	O=C(c1ccccc1I)c1cn(CCCCCO)c2ccccc12
AM694 N-Pentanoic Acid Metabolite	C20H18INO3	447.0326	O=C(O)CCCCn1cc(C(=O)c2ccccc2I)c2ccccc21
Amantadine	C10H17N	151.1356	C1C2CC3CC1CC(C2)(C3)N
AMAPN	C14H15NO	213.1148	CC(NC)C(C1=C2C=CC=CC2=CC=C1)=O
AMB	C19H27N3O3	345.2047	CC(C)[C@@H](C(OC)=O)NC(C1=NN(CCCCC)C2=C1C=CC=C2)=O
AMB-CHMICA (Valin)	C21H28N2O3	356.2094	C1(CCCCC1)CN1C=C(C2=CC=CC=C12)C(=O)N[C@H](C(=O)O)C(C)C
AMB-FUBICA	C22H23FN2O3	382.1687	CC(C)[C@@H](C(OC)=O)NC(C1=CN(CC2=CC=C(F)C=C2)C3=C1C=CC=C3)=O
AMB-PICA (Valin)	C19H26N2O3	330.1938	CC([C@@H](C(=O)O)NC(=O)C1=CN(C2=CC=CC=C12)CCCCC)C
Amfepramone	C13H19NO	205.1461	CC(N(CC)CC)C(C1=CC=CC=C1)=O
Aminorex	C9H10N2O	162.0788	C1(=CC=CC=C1)C1CN=C(O1)N
Amphetamine-N-propyl	C12H19N	177.1512	CCCNC(C)CC1=CC=CC=C1
AMT	C11H14N2	174.1152	CC(N)CC1=CNC2=C1C=CC=C2
α-Naphyrone	C19H23NO	281.1774	CCCC(N1CCCC1)C(C2=C3C=CC=CC3=CC=C2)=O
AP-237	C17H24N2O	272.1883	CCCC(=O)N1CCN(CC1)C/C=C/C2=CC=CC=C2
AP-238	C18H26N2O	286.2040	C(C=CC1=CC=CC=C1)N1CC(N(C(C1)C)C(CC)=O)C
APICA	C24H32N2O	364.2509	O=C(C1=CN(CCCCC)C2=C1C=CC=C2)NC34CC5CC(C4)CC(C5)C3
APICA (N-4-OH-Pentyl)	C24H32N2O2	380.2458	O=C(C1=CN(CCCC(O)C)C2=C1C=CC=C2)NC34CC5CC(C4)CC(C5)C3
α-PNP	C19H29NO	287.2244	CCCCCCCC(N1CCCC1)C(C2=CC=CC=C2)=O
A-PONASA	C25H33NO3S	427.2176	C12(CC3CC(CC(C1)C3)C2)NS(=O)(=O)C2=CC=C(C3=CC=CC=C23)OCCCCC

**Table S9.** Compound database imported into the *Search Mass Lists* node (continued)			
Compound Name	Molecular Formula	Exact Mass	SMILES
APP-BINACA	C21H24N4O2	364.1894	O=C(C1=NN(CCCC)C2=C1C=CC=C2)NC(CC3=CC=CC=C3)C(N)=O
APP-BUTINACA Phenylpropanoic Acid Metabolite	C21H23N3O3	365.1734	C(CCC)N1N=C(C2=CC=CC=C12)C(=O)N[C@@H](CC1=CC=CC=C1)C(=O)O
APP-CHMINACA	C24H28N4O2	404.2207	O=C(C1=NN(CC2CCCCC2)C3=C1C=CC=C3)N[C@@H](CC4=CC=CC=C4)C(N)=O
APP-FUBINACA	C24H21FN4O2	416.1643	O=C(C1=NN(CC2=CC=C(F)C=C2)C3=C1C=CC=C3)NC(CC4=CC=CC=C4)C(N)=O
APP-PICA	C23H27N3O2	377.2098	O=C(C1=CN(CCCCC)C2=C1C=CC=C2)N[C@@H](CC3=CC=CC=C3)C(N)=O
APP-PICA Phenylpropanoic Acid Metabolite	C23H26N2O3	378.1938	C(CCCC)N1C=C(C2=CC=CC=C12)C(=O)N[C@@H](CC1=CC=CC=C1)C(=O)O
APAA	C10H11NO2	177.0784	CC(C(C1=CC=CC=C1)C(N)=O)=O
APAAN	C10H9NO	159.0679	O=C(C(C#N)C1=CC=CC=C1)C
Arecoline	C8H13NO2	155.0941	O=C(C1=CCCN(C)C1)OC
Arfendazam	C18H17ClN2O3	344.0922	ClC1=CC2=C(N(CCC(N2C2=CC=CC=C2)=O)C(=O)OCC)C=C1
ATHPINACA	C24H31N3O2	393.2411	C12(CC3CC(CC(C1)C3)C2)NC(=O)C2=NN(C3=CC=CC=C23)CC2CCOCC2
ATHPINACA Isomer 2	C24H31N3O2	393.2411	O=C(C1=NN(CC2CCOCC2)C3=C1C=CC=C3)NC4C5CC6CC(C5)CC4C6
Atomoxetine	C17H21NO	255.1618	CC1=CC=CC=C1O[C@@H](C2=CC=CC=C2)CCNC
Atropine	C17H23NO3	289.1673	[H][C@@](CO)(C(=O)O[C@]1([H])C[C@]2([H])CC[C@]([H])(C1)N2C)C1=CC=CC=C1
AZEFUBIM	C19H17FN2O	308.1320	O=C(c1cn(Cc2ccc(F)cc2)c2ccccc12)N1CCC1
Azidoindolene 1	C21H28FN3O2	373.2160	CC1(C)C(C(=O)NN=C2C(=O)N(CCCCCF)c3ccccc32)C1(C)C
Baclofen	C10H12ClNO2	213.0551	O=C(O)CC(C1=CC=C(Cl)C=C1)CN
BAY 38-7271	C20H21F3O5S	430.1056	FC(CCCS(=O)(=O)OC1=CC(=CC=C1)OC1=C2C[C@@H](CC2=CC=C1)CO)(F)F
BAY-59-3074	C18H13F6NO4S	453.0464	O=S(CCCC(F)(F)F)(OC1=CC=CC(OC2=CC=CC(C(F)(F)F)=C2C#N)=C1)=O
BB-22	C25H24N2O2	384.1832	O=C(C1=CN(CC2CCCCC2)C3=C1C=CC=C3)OC4=C5N=CC=CC5=CC=C4
BB-22 3-Carboxyindole Metabolite	C16H19NO2	257.1410	O=C(C1=CN(CC2CCCCC2)C3=C1C=CC=C3)O
BDB	C11H15NO2	193.1097	CCC(N)CC1=CC=C(OCO2)C2=C1
BDMT	C24H30N4	374.2465	N1C(=C(C2=CC=CC=C12)CCN(C)C)C=C1NC2=CC=CC=C2C1CCN(C)C
Benocyclidine	C19H25NS	299.1702	S1C(=CC2=C1C=CC=C2)C2(CCCCC2)N2CCCCC2
Bentazepam	C17H16N2OS	296.0978	C1(=CC=CC=C1)C1=C2C(=NC(CN1)=O)SC1=C2CCCC1
Bentazepam	C17H16N2OS	296.0978	O=C1CN=C(C2=CC=CC=C2)C(C3=C(CCCC3)S4)=C4N1
Benzatropine	C21H25NO	307.1931	[H][C@]12CC[C@]([H])(C[C@@]([H])(C1)OC(C1=CC=CC=C1)C1=CC=CC=C1)N2C
Benzedrone	C17H19NO	253.1461	CC(NCC1=CC=CC=C1)C(C2=CC=C(C)C=C2)=O
Benzofuranyl-fentanyl	C24H28N2O2	376.2145	O1C=CC2=C1C=CC(=C2)CCN2CCC(CC2)N(C(CC)=O)C2=CC=CC=C2
Benzoylcyclohexylpiperidine	C18H25NO	271.1931	C1(=CC=CC=C1)C(=O)C1(CCCCC1)N1CCCCC1
Benzphetamine	C17H21N	239.1669	C(C1=CC=CC=C1)N([C@H](CC1=CC=CC=C1)C)C
Benzydamine	C19H23N3O	309.1836	CN(C)CCCOC1=NN(CC2=CC=CC=C2)C3=C1C=CC=C3
Benzyl-4-CN BUTINACA	C20H20N4O	332.1632	C(C1=CC=CC=C1)NC(=O)C1=NN(C2=CC=CC=C12)CCCCC#N
Benzylpiperazine	C11H16N2	176.1308	C(C1=CC=CC=C1)N1CCNCC1
beta-Hydroxy Acetyl Thiofentanyl	C19H24N2O2S	344.1553	CC(N(C1=CC=CC=C1)C2CCN(CC(C3=CC=CS3)O)CC2)=O
beta-Hydroxy-3-methyl-thienylfentanyl	C21H28N2O2S	372.1866	OC(CN1CC(C(CC1)N(C(CC)=O)C1=CC=CC=C1)C)C=1SC=CC1
beta-Hydroxy-carfentanil	C24H30N2O4	410.2200	OC(CN1CCC(CC1)(C(=O)OC)N(C(CC)=O)C1=CC=CC=C1)C1=CC=CC=C1
beta-Hydroxy-p-fluorofentanyl	C22H27FN2O2	370.2051	FC1=CC=C(C=C1)N(C(CC)=O)C1CCN(CC1)CC(C1=CC=CC=C1)O
beta-Hydroxy-PHP	C16H25NO	247.1931	C1(=CC=CC=C1)C(C(CCCC)N1CCCC1)O
beta-Hydroxy-sufentanil	C22H30N2O3S	402.1972	OC(CN1CCC(CC1)(COC)N(C(CC)=O)C1=CC=CC=C1)C=1SC=CC1
beta-Methoxy-2C-B	C11H16BrNO3	289.0308	BrC1=CC(=C(C=C1OC)C(CN)OC)OC
beta-Methyl Acetyl Fentanyl	C22H28N2O	336.2196	C1(=CC=CC=C1)N(C(C)=O)C1CCN(CC1)CC(C)C1=CC=CC=C1

**Table S9.** Compound database imported into the *Search Mass Lists* node (continued)			
Compound Name	Molecular Formula	Exact Mass	SMILES
Bisfluoromodafinil	C15H13F2NO2S	309.0630	O=C(N)CS(C(C1=CC=C(F)C=C1)C2=CC=C(F)C=C2)=O
bk-2C-B	C10H12BrNO3	272.9995	COC1=C(Br)C=C(OC)C(C(CN)=O)=C1
bk-2C-I	C10H12INO3	320.9856	NCC(=O)C1=C(C=C(C(=C1)OC)I)OC
bk-IMP	C13H17NO	203.1305	CC(NC)C(C1=CC2=C(CCC2)C=C1)=O
bk-IVP	C16H23NO	245.1774	CCCC(NCC)C(C1=CC2=C(CCC2)C=C1)=O
bk-MAPB	C13H17NO	203.1305	O1C=C(C2=C1C=CC=C2)CC(C)NCC
bk-MDA	C10H11NO3	193.0734	CC(N)C(C1=CC=C(OCO2)C2=C1)=O
bk-MDDMA	C12H15NO3	221.1047	CC(N(C)C)C(C1=CC=C(OCO2)C2=C1)=O
BMPEA	C9H13N	135.1043	CC(C1=CC=CC=C1)CN
BOD	C12H19NO3	225.1360	NCC(OC)C1=CC(OC)=C(C)C=C1OC
BOH-2C-B	C10H14BrNO3	275.0152	NCC(O)C1=C(C=C(C(=C1)OC)Br)OC
Boldenone	C19H26O2	286.1927	O=C\1\C=C/[C@]4(/C(=C/1)CC[C@@H]2[C@@H]4CC[C@@]3([C@@H](O)CC[C@@H]23)C)C
Bretazenil	C19H20BrN3O3	417.0683	BrC1=CC=CC2=C1C(N1C(C=3N2C=NC3C(=O)OC(C)(C)C)CCC1)=O
Brifentanyl	C20H29FN6O3	420.2280	O=C(N([C@@H]1[C@H](C)CN(CCN2N=NN(CC)C2=O)CC1)C3=CC=CC=C3F)COC
Bromadol	C22H28BrNO	401.1349	CN(C)C1(CCC(CC1)(CCC2=CC=CC=C2)O)C3=CC=C(C=C3)Br
Bromantane	C16H20BrN	305.0774	BrC1=CC=C(NC2C3CC4CC(C3)CC2C4)C=C1
Bromazepam Cleavage Product	C12H9BrN2O	275.9893	C1=CC=NC(=C1)C(=O)C2=C(C=CC(=C2)Br)N
Bromo-DragonFLY	C13H12BrNO2	293.0046	CC(N)CC1=C2C(OC=C2)=C(Br)C3=C1OC=C3
Brorphine	C20H22BrN3O	399.0941	BrC1=CC=C(C=C1)C(C)N1CCC(CC1)N1C(NC2=C1C=CC=C2)=O
Brotizolam	C15H10BrClN4S	391.9493	CC1=NN=C2N1C3=C(C=C(S3)Br)C(=NC2)C4=CC=CC=C4Cl
BTCPy	C18H23NS	285.1546	S1C(=CC2=C1C=CC=C2)C2(CCCCC2)N2CCCC2
Bufotenine	C12H16N2O	204.1257	CN(CCC1=CNC2=CC=C(C=C12)O)C
Buphedrone	C11H15NO	177.1148	CCC(NC)C(C1=CC=CC=C1)=O
Bupivacaine	C18H28N2O	288.2196	O=C(C1N(CCCC1)CCCC)NC2=C(C)C=CC=C2C
Bupropion	C13H18ClNO	239.1072	C(C)(C)(C)NC(C(=O)C1=CC(=CC=C1)Cl)C
Butorphanol	C21H29NO2	327.2193	OC1=CC=C2C[C@]3([H])[C@]4(O)CCCC[C@]4(CCN3CC5CCC5)C2=C1
Butylone	C12H15NO3	221.1047	O1COC2=C1C=CC(=C2)C(C(CC)NC)=O
Butyryl-carfentanyl	C25H32N2O3	408.2407	C(CCC)(=O)N(C1(CCN(CC1)CCC1=CC=CC=C1)C(=O)OC)C1=CC=CC=C1
Butyrylfentanyl Carboxy Metabolite	C23H28N2O3	380.2094	O=C(CCC(O)=O)N(C1CCN(CCC2=CC=CC=C2)CC1)C3=CC=CC=C3
Butyrylremifentanil	C21H30N2O5	390.2149	C(CCC)(=O)N(C1(CCN(CC1)CCC(=O)OC)C(=O)OC)C1=CC=CC=C1
BZO-4en-POXIZID	C20H19N3O2	333.1472	O=C\1N(C2=CC=CC=C2/C1=N/NC(C1=CC=CC=C1)=O)CCCC=C
BZO-CHMOXIZID	C22H23N3O2	361.1785	C1(CCCCC1)CN1C(\C(\C2=CC=CC=C12)=N/NC(C1=CC=CC=C1)=O)=O
BZO-HEPOXIZID	C22H25N3O2	363.1941	C(CCCCCC)N1C(\C(\C2=CC=CC=C12)=N/NC(C1=CC=CC=C1)=O)=O
BZO-HEXOXIZID	C21H23N3O2	349.1785	C(CCCCC)N1C(\C(\C2=CC=CC=C12)=N/NC(C1=CC=CC=C1)=O)=O
BZO-POXIZID	C20H21N3O2	335.1628	C(CCCC)N1C(C(C2=CC=CC=C12)=NNC(C1=CC=CC=C1)=O)=O
Camazepam	C19H18ClN3O3	371.1031	CN(C(OC1C(N(C2=C(C(=N1)C1=CC=CC=C1)C=C(C=C2)Cl)C)=O)=O)C
Camfetamine	C14H19N	201.1512	CNC1C2CCC(C1C1=CC=CC=C1)C2
Carbonyl-bromadol	C23H28BrNO2	429.1298	BrC1=CC=C(C=C1)C(=O)C1(CCC(CC1)(CCC1=CC=CC=C1)O)N(C)C
Carburazepam	C17H16ClN3O2	329.0926	ClC=1C=CC2=C(C(N(CC(N2C)=O)C(=O)N)C2=CC=CC=C2)C1
Carfentanil	C24H30N2O3	394.2251	C1(=CC=CC=C1)CCN1CCC(CC1)(C(=O)OC)N(C(CC)=O)C1=CC=CC=C1
Cathinone	C9H11NO	149.0835	C[C@H](N)C(C1=CC=CC=C1)=O
CB-13	C26H24O2	368.1771	O=C(C1=C2C=CC=CC2=CC=C1)C3=C4C=CC=CC4=C(OCCCCC)C=C3

Table S9. Compound database imported into the *Search Mass Lists* node (continued)

Compound Name	Molecular Formula	Exact Mass	SMILES
CB-25	C25H41NO3	403.3081	O=C(NC1CC1)CCCCCCCCCCOC2=CC(CCCCC)=CC(O)=C2
CB-52	C26H43NO3	417.3238	O=C(NC1CC1)CCCCCCCCCCOC2=CC(O)=CC=C2CCCCCC
CB-86	C26H43NO3	417.3238	CCCCCCC(C)(C)C1=CC(O)=CC(OCCCCCCCC(O)=NC2CC2)=C1
CBL-018	C24H23NO2	357.1723	O=C(C1=CN(CCCCC)C2=C1C=CC=C2)OC3=C4C=CC=CC4=CC=C3
Cephaeline	C28H38N2O4	466.2826	[H][C@]1(C[C@@]2([H])C[C@]3([H])N(CCC4=CC(OC)=C(OC)C=C34)C[C@]2([H])CC)NCCC2=CC(O)=C(OC)C=C12
CH-FUBBMPDORA	C20H22BrFN2O2	420.0843	BrC=1C(=C(C(N(C1)CC1=CC=C(C=C1)F)=O)NC(=O)C1CCCCC1)C
CH-FUBIATA	C23H25FN2O	364.1946	C1(CCCCC1)NC(CC1=CN(C2=CC=CC=C12)CC1=CC=C(C=C1)F)=O
CH-IACA	C16H20N2O	256.1570	C1(CCCCC1)NC(CC1=CNC2=CC=CC=C12)=O
Chlordiazepoxide	C16H14ClN3O	299.0820	ClC=1C=CC2=C(C(=[N+](CC(=N2)NC)[O-])C2=CC=CC=C2)C1
Chlorphine	C20H22ClN3O	355.1446	ClC1=CC=C(C=C1)C(C)N1CCC(CC1)N1C(NC2=C1C=CC=C2)=O
CHM-122	C27H27NO	381.2087	O=C(C1=CN(CC2CCCCC2)C3=C1C=CC=C3)C4=C5C=CC=CC5=C(C)C=C4
CHM-FUBIATA	C24H27FN2O	378.2102	C1(CCCCC1)CNC(CC1=CN(C2=CC=CC=C12)CC1=CC=C(C=C1)F)=O
CHM-MDMB-CHMINACA	C28H41N3O3	467.3142	C1(CCCCC1)CN1N=C(C2=CC=CC=C12)C(=O)NC(C(=O)OCC1CCCCC1)C(C)(C)C
CH-PIATA	C21H30N2O	326.2353	C1(CCCCC1)NC(CC1=CN(C2=CC=CC=C12)CCCCC)=O
Ciclotizolam	C20H18BrClN4S	460.0119	BrC1=CC=2C(=NCC=3N(C2S1)C(=NN3)C3CCCCC3)C3=C(C=CC=C3)Cl
Cinazepam	C19H14BrClN2O5	463.9769	O=C(O)CCC(OC1N=C(C2=CC=CC=C2Cl)C3=CC(Br)=CC=C3NC1=O)=O
Cinolazepam	C18H13ClFN3O2	357.0675	FC1=CC=CC=C1C2=NC(C(N(CCC#N)C3=C2C=C(C=C3)Cl)=O)O
cis-3-Methyl Butyryl Fentanyl	C24H32N2O	364.2509	CCCC(N([C@H]1[C@H](C)CN(CCC2=CC=CC=C2)CC1)C3=CC=CC=C3)=O
Citicoline	C14H26N4O11P2	488.1067	P(=O)(OP(=O)(O)OCC1OC(C(C1O)O)N1C(N=C(C=C1)N)=O)(OCC[N+](C)(C)C)[O-]
Cl2201	C24H21ClFNO	393.1290	O=C(C1=C2C=CC=CC2=C(Cl)C=C1)C3=CN(CCCCCF)C4=C3C=CC=C4
Clazolam	C18H17ClN2O	312.1024	ClC=1C=CC2=C(C3N(CC(N2C)=O)CCC2=CC=CC=C23)C1
Climazolam	C18H13Cl2N3	341.0481	ClC=1C=CC2=C(C(=NCC=3N2C(=NC3)C)C3=C(C=CC=C3)Cl)C1
Clobazam	C16H13ClN2O2	300.0660	ClC1=CC2=C(N(C(CC(N2C2=CC=CC=C2)=O)=O)C)C=C1
Clobromazolam	C17H12BrClN4	385.9928	CC1=NN=C2N1C3=C(C=C(C=C3)Br)C(=NC2)C4=CC=CC=C4Cl
Clonazepam	C15H10ClN3O3	315.0405	[O-][N+](C1=CC2=C(C=C1)NC(CN=C2C3=CC=CC=C3Cl)=O)=O
Cloniprazepam	C19H16ClN3O3	369.0875	ClC1=C(C=CC=C1)C=1C2=C(N(C(CN1)=O)CC1CC1)C=CC(=C2)[N+](=O)[O-]
Clonitazene	C20H23ClN4O2	386.1504	ClC1=CC=C(C=C1)CC1=NC2=C(N1CCN(CC)CC)C=CC(=C2)[N+](=O)[O-]
Clotiazepam	C16H15ClN2OS	318.0588	ClC1=C(C=CC=C1)C=1C2=C(N(C(CN1)=O)C)SC(=C2)CC
Cloxazolam	C17H14Cl2N2O2	348.0427	ClC=1C=CC2=C(C3(N(CC(N2)=O)CCO3)C3=C(C=CC=C3)Cl)C1
Clozapine	C18H19ClN4	326.1293	ClC=1C=CC2=C(N=C(C3=C(N2)C=CC=C3)N3CCN(CC3)C)C1
Cl-Pseudoephedrine	C10H14ClN	183.0809	ClC(C(NC)C)C1=CC=CC=C1
Coluracetam	C19H23N3O3	341.1734	CC1=C(C=2C(=NC=3CCCCC3C2NC(CN2C(CCC2)=O)=O)O1)C
CP 55,244	C26H42O3	402.3129	O[C@H]1C[C@@]2([H])CC[C@@H](CO)C[C@]2([H])[C@@H](C3=CC=C(C(CCCCCC)(C)C)C=C3O)C1
CP-47,497	C21H34O2	318.2553	OC1=CC(C(CCCCCC)(C)C)=CC=C1[C@H]2C[C@@H](O)CCC2
CP-47,497-C8-homolog	C22H36O2	332.2710	OC1=CC(C(CCCCCCC)(C)C)=CC=C1[C@@H]2C[C@@H](O)CCC2
CP-55,940	C24H40O3	376.2972	O[C@H]1C[C@@H](C2=CC=C(C(C)(C)CCCCCC)C=C2O)[C@H](CCCO)CC1
CPCPP	C13H18Cl2N2	272.0842	ClCCCN1CCN(C2=CC=CC(Cl)=C2)CC1
CPE	C24H28N2O2	376.2145	CN1C(CN2C=C(C(CC3=CC=CC=C3OC)=O)C4=C2C=CC=C4)CCCC1
CUMYL CBMICA	C23H26N2O	346.2040	C1(CCC1)CN1C=C(C2=CC=CC=C12)C(=O)NC(C)(C)C1=CC=CC=C1
Cumyl-BC-HpMeGaClone-221	C28H30N2O	410.2353	C12C(CC(CC1)C2)CN2C1=C(C=3C=CC=CC23)C(N(C=C1)C(C)(C)C1=CC=CC=C1)=O
Cumyl-BICA	C22H26N2O	334.2040	O=C(C1=CN(CCCC)C2=C1C=CC=C2)NC(C)(C3=CC=CC=C3)C
CUMYL-CBEICA	C24H28N2O	360.2196	C1(CCC1)CCN1C=C(C2=CC=CC=C12)C(=O)NC(C)(C)C1=CC=CC=C1

**Table S9.** Compound database imported into the *Search Mass Lists* node (continued)			
Compound Name	Molecular Formula	Exact Mass	SMILES
Cumyl-Cb-MeGaClone	C25H26N2O	370.2040	C1(CCC1)CN1C2=C(C=3C=CC=CC13)C(N(C=C2)C(C)(C2=CC=CC=C2)C)=O
CUMYL-CBMINACA	C22H25N3O	347.1992	C1(CCC1)CN1N=C(C2=CC=CC=C12)C(=O)NC(C)(C)C1=CC=CC=C1
Cumyl-CH-MeGaClone	C27H30N2O	398.2353	C1(CCCCC1)CN1C2=C(C=3C=CC=CC13)C(N(C=C2)C(C)(C2=CC=CC=C2)C)=O
Cumyl-CHSINACA	C23H27N3O3S	425.1768	C1(CCCCC1)S(=O)(=O)N1N=C(C2=CC=CC=C12)C(=O)NC(C)(C)C1=CC=CC=C1
Cumyl-ClCHSINACA	C23H26ClN3O3S	459.1378	ClC1(CCCCC1)S(=O)(=O)N1N=C(C2=CC=CC=C12)C(=O)NC(C)(C)C1=CC=CC=C1
CUMYL-CPEICA	C23H26N2O	346.2040	C1(CC1)CCN1C=C(C2=CC=CC=C12)C(=O)NC(C)(C)C1=CC=CC=C1
Cumyl-INACA	C17H17N3O	279.1366	C1(=CC=CC=C1)C(C)(C)NC(=O)C1=NNC2=CC=CC=C12
CUMYL-NBMICA	C26H30N2O	386.2353	C12C(CC(CC1)C2)CN2C=C(C1=CC=CC=C21)C(=O)NC(C)(C)C2=CC=CC=C2
CUMYL-NBMINACA	C25H29N3O	387.2305	C12C(CC(CC1)C2)CN2N=C(C1=CC=CC=C21)C(=O)NC(C)(C)C2=CC=CC=C2
Cumyl-PeGACLONE	C25H28N2O	372.2196	CC(C)(C1=CC=CC=C1)N1C(C2=C(N(C=3C=CC=CC23)CCCCC)C=C1)=O
CUMYL-PICA	C23H28N2O	348.2196	O=C(C1=CN(CCCCC)C2=C1C=CC=C2)NC(C)(C3=CC=CC=C3)C
CUMYL-PICA N-Pentanoic Acid Metabolite	C23H26N2O3	378.1938	CC(C)(NC(=O)c1cn(CCCCC(=O)O)c2ccccc12)c1ccccc1
CUMYL-PINACA	C22H27N3O	349.2149	O=C(C1=NN(CCCCC)C2=C1C=CC=C2)NC(C)(C3=CC=CC=C3)C
CUMYL-PIPETINACA	C24H30N4O	390.2414	O=C(C1=NN(CCN2CCCCC2)C3=C1C=CC=C3)NC(C)(C4=CC=CC=C4)C
CUMYL-THPINACA	C23H27N3O2	377.2098	O=C(C1=NN(CC2CCOCC2)C3=C1C=CC=C3)NC(C)(C4=CC=CC=C4)C
CUMYL-TsINACA	C24H23N3O3S	433.1455	CC(C)(C1=CC=CC=C1)NC(=O)C1=NN(C2=CC=CC=C12)S(=O)(=O)C1=CC=C(C=C1)C
Cyclazodone	C12H12N2O2	216.0893	C1CC1N=C2NC(=O)C(O2)C3=CC=CC=C3
Cyclohexalamine	C12H17N	175.1356	C1(=CC=CC=C1)C1(CCCCC1)N
Cyclopropyl Norfentanyl	C15H20N2O	244.1570	C1(=CC=CC=C1)N(C(=O)C1CC1)C1CCNCC1
Cyclopropyl U-47700	C18H24Cl2N2O	354.1260	ClC=1C=C(C(=O)N([C@@H]2[C@H](CCCC2)N(C)C)C2CC2)C=CC1Cl
Cyclopropylmescaline	C14H21NO3	251.1516	C1(CC1)COC1=C(C=C(C=C1OC)CCN)OC
Cyprazepam	C19H18ClN3O	339.1133	ClC=1C=CC2=C(C(=N(CC(=N2)NCC2CC2)=O)C2=CC=CC=C2)C1
Cyproheptadine	C21H21N	287.1669	C1=CC=CC=2C(C3=C(C=CC21)C=CC=C3)=C3CCN(CC3)C
D2PM	C17H19NO	253.1461	OC(C1=CC=CC=C1)(C2=CC=CC=C2)C3NCCC3
DALT	C16H20N2	240.1621	C=CCN(CCC1=CNC2=C1C=CC=C2)CC=C
DB-MDBP	C12H14F2N2O2	256.1018	FC1(F)OC2=CC=C(CN3CCNCC3)C=C2O1
Dehydro-deschloroketamine	C13H15NO	201.1148	CNC1(C(C=CCC1)=O)C1=CC=CC=C1
DehydroMethoxetamine	C15H19NO2	245.1410	C(C)NC1(C(C=CCC1)=O)C1=CC(=CC=C1)OC
Dehydronorketamine	C12H12ClNO	221.0602	NC1(CCC=CC1=O)C1=C(C=CC=C1)Cl
Delorazepam	C15H10Cl2N2O	304.0165	O=C1NC2=CC=C(Cl)C=C2C(C3=CC=CC=C3Cl)=NC1
Demoxepam	C15H11ClN2O2	286.0504	ClC=1C=CC2=C(C(=N(CC(N2)=O)=O)C2=CC=CC=C2)C1
Desalkylgidazepam	C15H11BrN2O	314.0049	BrC=1C=CC2=C(C(=NCC(N2)=O)C2=CC=CC=C2)C1
Deschloro W-19	C19H23N3O2S	357.1506	O=S(C1=CC=CC=C1)(/N=C2N(CCC3=CC=C(N)C=C3)CCCC\2)=O
Deschloroclotizolam	C15H11ClN4S	314.0388	ClC1=CC=2C(=NCC=3N(C2S1)C(=NN3)C)C3=CC=CC=C3
Deschloroketamine	C13H17NO	203.1305	CNC1(C(CCCC1)=O)C1=CC=CC=C1
Deschloro-N-ethyl-ketamine	C14H19NO	217.1461	C(C)NC1(C(CCCC1)=O)C1=CC=CC=C1
Deschloronorketamine	C12H15NO	189.1148	O=C1C(C2=CC=CC=C2)(N)CCCC1
Desmethylclotiazepam	C15H13ClN2OS	304.0432	O=C1CN=C(C2=CC=CC=C2Cl)C(C=C(CC)S3)=C3N1
Desmethylflutiazepam	C15H13FN2OS	288.0727	C(C)C1=CC2=C(NC(CN=C2C2=C(C=CC=C2)F)=O)S1
Desmethylmoramide	C24H30N2O2	378.2302	N1(CCOCC1)CCC(C(=O)N1CCCC1)(C1=CC=CC=C1)C1=CC=CC=C1
Desmethylnitrazolam	C16H11N5O2	305.0907	[N+](=O)([O-])C=1C=CC2=C(C(=NCC=3N2C=NN3)C3=CC=CC=C3)C1
Desmethylrilmazolam	C18H13Cl2N5O	385.0492	ClC=1C=CC2=C(C(=NCC=3N2N=C(N3)C(=O)NC)C3=C(C=CC=C3)Cl)C1

**Table S9.** Compound database imported into the *Search Mass Lists* node (continued)			
Compound Name	Molecular Formula	Exact Mass	SMILES
Desmethyltriazolam	C16H10Cl2N4	328.0277	ClC=1C=CC2=C(C(=NCC=3N2C=NN3)C3=C(C=CC=C3)Cl)C1
Desoxypipradrol	C18H21N	251.1669	C1(C(C2=CC=CC=C2)C3=CC=CC=C3)NCCCC1
Despropionyl meta-Chlorofentanyl	C19H23ClN2	314.1544	ClC=1C=C(C=CC1)NC1CCN(CC1)CCC1=CC=CC=C1
Despropionyl meta-Fluoro Fentanyl	C19H23FN2	298.1840	FC1=CC(NC2CCN(CCC3=CC=CC=C3)CC2)=CC=C1
Despropionyl meta-Methyl Fentanyl	C20H26N2	294.2091	CC1CN(CCC2=CC=CC=C2)CCC1NC3=CC=CC=C3
Despropionyl ortho-Chlorofentanyl	C19H23ClN2	314.1544	ClC1=C(C=CC=C1)NC1CCN(CC1)CCC1=CC=CC=C1
Despropionyl ortho-Methyl Fentanyl	C20H26N2	294.2091	CC1=CC=CC=C1NC2CCN(CCC3=CC=CC=C3)CC2
Despropionyl para-Chlorofentanyl	C19H23ClN2	314.1544	ClC1=CC=C(C=C1)NC1CCN(CC1)CCC1=CC=CC=C1
Despropionyl Remifentanil	C17H24N2O4	320.1731	COC(=O)C1(CCN(CC1)CCC(=O)OC)NC1=CC=CC=C1
DET	C14H20N2	216.1621	CCN(CC)CCC1=CNC2=CC=CC=C12
Dextromethorphan	C18H25NO	271.1931	[H][C@]12CC3=C(C=C(OC)C=C3)[C@]3(CCCC[C@]13[H])CCN2C
Di(beta-phenylisopropyl)amine	C18H23N	253.1825	CC(CC1=CC=CC=C1)NC(C)CC2=CC=CC=C2
Dibenzylpiperazine	C18H22N2	266.1778	N1(CC2=CC=CC=C2)CCN(CC3=CC=CC=C3)CC1
Dibutylone	C13H17NO3	235.1203	O1COC2=C1C=CC(=C2)C(C(CC)N(C)C)=O
Dichloroethcathinone	C11H13Cl2NO	245.0369	CC(NCC)C(C1=CC=C(Cl)C(Cl)=C1)=O
Diclofensine	C17H17Cl2NO	321.0682	ClC=1C=C(C=CC1Cl)C1CN(CC2=CC(=CC=C12)OC)C
Didesmethylrilmazolam	C17H11Cl2N5O	371.0335	ClC=1C=CC2=C(C(=NCC=3N2N=C(N3)C(=O)N)C3=C(C=CC=C3)Cl)C1
Didesmethylsibutramine	C15H22ClN	251.1435	CC(C)CC(N)C1(CCC1)C1=CC=C(Cl)C=C1
Diethylone	C14H19NO3	249.1360	CC(N(CC)CC)C(C1=CC=C(OCO2)C2=C1)=O
Diethylpentylone	C16H23NO3	277.1673	CCCC(N(CC)CC)C(C1=CC=C(OCO2)C2=C1)=O
Dieticyclidine	C16H25N	231.1982	C(C)N(C1(CCCCC1)C1=CC=CC=C1)CC
Dimetamine	C15H21NO	231.1618	CN(C1(CCC(CC1)=O)C1=CC=C(C=C1)C)C
Dimethocaine	C16H26N2O2	278.1989	O=C(OCC(C)(C)CN(CC)CC)C1=CC=C(N)C=C1
Diphenidine	C19H23N	265.1825	C1(=CC=CC=C1)C(CC1=CC=CC=C1)N1CCCCC1
Dipipanone	C24H31NO	349.2400	C1(=CC=CC=C1)C(C(CC)=O)(CC(C)N1CCCCC1)C1=CC=CC=C1
DiPT	C16H24N2	244.1934	CC(N(CCC1=CNC2=C1C=CC=C2)C(C)C)C
Dipyanone	C23H29NO	335.2244	C1(=CC=CC=C1)C(C(CC)=O)(CC(C)N1CCCC1)C1=CC=CC=C1
DL-4662	C15H23NO3	265.1673	CCCC(NCC)C(C1=CC=C(OC)C(OC)=C1)=O
DMAR	C11H14N2O	190.1101	NC1=NC(C)C(C2=CC=C(C)C=C2)O1
DMCPA	C12H17NO2	207.1254	COC1=C(C=C(C(=C1)C)OC)C1CC(C1)N
DMMA	C12H19NO2	209.1410	CC(NC)CC1=CC=C(OC)C(OC)=C1
DMPEA	C10H15NO2	181.1097	NCCC1=CC=C(OC)C(OC)=C1
DMT	C12H16N2	188.1308	N1C=C(C2=CC=CC=C12)CCN(C)C
DMXE	C15H21NO	231.1618	CCNC1(CCCCC1=O)c1cccc(C)c1
DOAM	C16H27NO2	265.2036	CC(N)CC1=CC(OC)=C(CCCCC)C=C1OC
DOB	C11H16BrNO2	273.0359	CC(N)CC1=CC(OC)=C(Br)C=C1OC
DOBU	C15H25NO2	251.1880	CC(N)CC1=CC(OC)=C(CCCC)C=C1OC
DOC	C11H16ClNO2	229.0864	CC(N)CC1=CC(OC)=C(Cl)C=C1OC
DOC Theophylline Derivative	C20H26ClN5O4	435.1668	O=C(N1C)N(C)C2=C(N(CCNC(C)CC3=CC(OC)=C(Cl)C=C3OC)C=N2)C1=O
DOF	C11H16FNO2	213.1160	CC(N)CC1=CC(OC)=C(F)C=C1OC
DOI	C11H16INO2	321.0220	CC(N)CC1=CC(OC)=C(I)C=C1OC
DOIP	C14H23NO2	237.1723	CC(N)CC1=CC(OC)=C(C(C)C)C=C1OC

Table S9. Compound database imported into the *Search Mass Lists* node (continued)

Compound Name	Molecular Formula	Exact Mass	SMILES
DOM	C12H19NO2	209.1410	CC(N)CC1=CC(OC)=C(C)C=C1OC
DON	C11H16N2O4	240.1105	COC1=C(C=C(C(=C1)[N+](=O)[O-])OC)CC(C)N
DOPR	C14H23NO2	237.1723	CC(N)CC1=CC(OC)=C(CCC)C=C1OC
DOT	C12H19NO2S	241.1131	CC(N)CC1=CC(OC)=C(SC)C=C1OC
Doxefazepam	C17H14ClFN2O3	348.0671	ClC=1C=CC2=C(C(=NC(C(N2CCO)=O)O)C2=C(C=CC=C2)F)C1
DPT	C16H24N2	244.1934	CCCN(CCC1=CNC2=C1C=CC=C2)CCC
EADB-FUBINACA	C23H27FN4O2	410.2113	CCNC(=O)C(NC(=O)c1nn(Cc2ccc(F)cc2)c2ccccc12)C(C)(C)C
EAM-2201	C26H26FNO	387.1993	O=C(C1=C2C=CC=CC2=C(CC)C=C1)C3=CN(CCCCCF)C4=C3C=CC=C4
EDMB-4en-PINACA	C21H29N3O3	371.2203	CC(C(C(=O)OCC)NC(=O)C1=NN(C2=CC=CC=C12)CCCC=C)(C)C
EDMB-CHMICA	C24H34N2O3	398.2564	CCOC(=O)C(NC(=O)c1cn(CC2CCCCC2)c2ccccc12)C(C)(C)C
EDMB-PINACA	C21H31N3O3	373.2360	CC([C@@H](C(=O)OCC)NC(=O)C1=NN(C2=CC=CC=C12)CCCCC)(C)C
EFLEA	C12H17NO3	223.1203	ON(C(C)CC1=CC=C2OCCOC2=C1)C
EG-018	C28H25NO	391.1931	O=C(C1=C2C=CC=CC2=CC=C1)C3=CC4=C(C=C3)N(CCCCC)C5=C4C=CC=C5
EG2201	C28H24FNO	409.1837	FCCCCCN1C2=CC=CC=C2C2=C1C=CC(=C2)C(=O)C1=CC=CC2=CC=CC=C12
Elfazepam	C19H18ClFN2O3S	408.0705	ClC=1C=CC2=C(C(=NCC(N2CCS(=O)(=O)CC)=O)C2=C(C=CC=C2)F)C1
Eluxadoline	C32H35N5O5	569.2633	O=C(O)C1=CC(CN(C([C@@H](N)CC2=C(C)C=C(C(N)=O)C=C2C)=O)[C@H](C3=NC(C4=CC=CC=C4)=CN3)C)=CC=C1OC
EMB-FUBINACA	C22H24FN3O3	397.1796	CC(C)[C@@H](C(OCC)=O)NC(C1=NN(CC2=CC=C(F)C=C2)C3=C1C=CC=C3)=O
Embutramide	C17H27NO3	293.1986	O=C(NCC(C1=CC=CC(OC)=C1)(CC)CC)CCCO
Emetine	C29H40N2O4	480.2983	[H][C@]1(C[C@@]2([H])C[C@]3([H])N(CCC4=CC(OC)=C(OC)C=C34)C[C@]2([H])CC)NCCC2=CC(OC)=C(OC)C=C12
Ephedrine	C10H15NO	165.1148	C[C@H](NC)[C@@H](C1=CC=CC=C1)O
Ephenidine	C16H19N	225.1512	CCNC(CC1=CC=CC=C1)C2=CC=CC=C2
Ephinazone	C16H14N2O	250.1101	C(C)C1=NC2=CC=CC=C2C(N1C1=CC=CC=C1)=O
Epirocaine	C14H21NO2	235.1567	O=C(OCC(NCCC)(C)C)C1=CC=CC=C1
Ergocristine	C35H39N5O5	609.2946	C(C1=CC=CC=C1)[C@@H]1N2C([C@](O[C@]2([C@@H]2CCCN2C1=O)O)(C(C)C)NC(=O)[C@H]1CN([C@@H]2CC=3C4=C(C2=C1)C=CC=C4NC3)C)=O
Ergonovine	C19H23N3O2	325.1785	OC[C@H](C)NC(=O)[C@H]1CN([C@@H]2CC=3C4=C(C2=C1)C=CC=C4NC3)C
Ergotamine	C33H35N5O5	581.2633	C(C1=CC=CC=C1)[C@H]1C(N2[C@H]([C@]3(N1C([C@](O3)(C)NC(=O)[C@H]3CN([C@@H]1CC=4C5=C(C1=C3)C=CC=C5NC4)C)=O)O)CCC2)=O
Escaline	C12H19NO3	225.1360	NCCC1=CC(OC)=C(OCC)C(OC)=C1
Estazolam	C16H11ClN4	294.0667	ClC=1C=CC2=C(C(=NCC=3N2C=NN3)C3=CC=CC=C3)C1
Etaqualone	C17H16N2O	264.1257	O=C1N(C2=CC=CC=C2CC)C(C)=NC3=C1C=CC=C3
Ethcathinone	C11H15NO	177.1148	CC(NCC)C(C1=CC=CC=C1)=O
Ethiopropamine	C9H15NS	169.0920	C(C)NC(CC=1SC=CC1)C
ETH-LAD	C21H27N3O	337.2149	O=C([C@H](C=C12)CN(CC)[C@]2([H])CC3=CNC4=C3C1=CC=C4)N(CC)CC
Ethyl-(1-phenylbutan-2-yl)amine	C12H19N	177.1512	CCC(NCC)CC1=CC=CC=C1
Ethylbenzyl-CYBINACA	C22H24N4O	360.1945	O=C(C1=NN(CCCCC#N)C2=C1C=CC=C2)NC(C3=CC=CC=C3)CC
Ethyleneoxynitazene	C22H26N4O3	394.1999	O1CCC2=C1C=CC(=C2)CC2=NC1=C(N2CCN(CC)CC)C=CC(=C1)[N+](=O)[O-]
Ethylmorphine	C19H23NO3	313.1673	C(C)OC1=CC=C2C[C@H]3N(CC[C@]45[C@@H](OC1=C52)[C@H](C=C[C@@H]34)O)C
Ethylone	C12H15NO3	221.1047	CC(NCC)C(C1=CC=C(OCO2)C2=C1)=O
Ethylphenidate	C15H21NO2	247.1567	CCOC(=O)C(C1CCCCN1)C2=CC=CC=C2
Ethyphenethyl-FUBICA	C26H25FN2O	400.1946	CCC(Cc1ccccc1)NC(=O)c1cn(Cc2ccc(F)cc2)c2ccccc12
Eticyclidine	C14H21N	203.1669	CCNC1(CCCCC1)C2=CC=CC=C2
Etorphine	C25H33NO4	411.2404	O[C@](C)(CCC)[C@H]1C[C@@]23[C@]45CCN([C@@H]2CC=2C=CC(=C(O[C@H]4[C@]1(C=C3)OC)C25)O)C
F2201	C24H21F2NO	377.1586	O=C(C1=C2C=CC=CC2=C(F)C=C1)C3=CN(CCCCCF)C4=C3C=CC=C4

**Table S9.** Compound database imported into the *Search Mass Lists* node (continued)			
Compound Name	Molecular Formula	Exact Mass	SMILES
FAB-144	C20H27FN2O	330.2102	O=C(C1=NN(CCCCCF)C2=C1C=CC=C2)C3C(C)(C)C3(C)C
FDU-NNEI	C26H19FN2O	394.1476	O=C(C1=CN(CC2=CC=C(F)C=C2)C3=C1C=CC=C3)NC4=C5C=CC=CC5=CC=C4
FDU-PB-22	C26H18FNO2	395.1316	O=C(C1=CN(CC2=CC=C(F)C=C2)C3=C1C=CC=C3)OC4=C5C=CC=CC5=CC=C4
Fenethylline	C18H23N5O2	341.1846	O=C(N1C)N(C)C2=C(N(CCNC(C)CC3=CC=CC=C3)C=N2)C1=O
Fenozolone	C11H12N2O2	204.0893	C(C)NC=1OC(C(N1)=O)C1=CC=CC=C1
Fenproporex	C12H16N2	188.1308	C1(=CC=CC=C1)CC(C)NCCC#N
Fentranyl	C23H28N2O	348.2196	C1(=CC=CC=C1)N(C(CC)=O)C1CCN(CC1)C1C(C1)C1=CC=CC=C1
Fladrafinil	C15H13F2NO3S	325.0579	O=C(NO)CS(C(C1=CC=C(F)C=C1)C2=CC=C(F)C=C2)=O
Fletazepam	C17H13ClF4N2	356.0698	ClC=1C=CC2=C(C(=NCCN2CC(F)(F)F)C2=C(C=CC=C2)F)C1
Flibanserin	C20H21F3N4O	390.1662	O=C1N(CCN2CCN(C3=CC=CC(C(F)(F)F)=C3)CC2)C4=CC=CC=C4N1
Fluadinazolam	C19H17ClFN5	369.1151	ClC=1C=CC2=C(C(=NCC=3N2C(=NN3)CN(C)C)C3=C(C=CC=C3)F)C1
Flubromazepam Isomer	C15H10BrFN2O	331.9955	O=C1NC2=CC=C(F)C=C2C(C3=CC=CC=C3Br)=NC1
Flubrometazepam	C16H12BrFN2O	346.0112	BrC=1C=CC2=C(C(=NCC(N2C)=O)C2=C(C=CC=C2)F)C1
Flubrotizolam	C15H10BrFN4S	375.9788	BrC1=CC=2C(=NCC=3N(C2S1)C(=NN3)C)C3=C(C=CC=C3)F
Fludiazepam	C16H12ClFN2O	302.0617	CN1C(=O)CN=C(C2=C1C=CC(=C2)Cl)C3=CC=CC=C3F
Fluetizolam	C17H15FN4S	326.0996	C(C)C=1SC=2N3C(=NN=C3CN=C(C2C1)C1=C(C=CC=C1)F)C
Fluloprazolam	C23H21FN6O3	448.1654	FC1=C(C=CC=C1)C1=NCC=2N(C3=C1C=C(C=C3)[N+](=O)[O-])C(/C(/N2)=C/N2CCN(CC2)C)=O
Flunitazene	C20H23FN4O2	370.1800	C(C)N(CCN1C(=NC2=C1C=CC(=C2)[N+](=O)[O-])CC2=CC=C(C=C2)F)CC
Flunitrazepam	C16H12FN3O3	313.0857	O=C1N(C)C2=CC=C([N+]([O-])=O)C=C2C(C3=CC=CC=C3F)=NC1
Flunitrazolam	C17H12FN5O2	337.0969	FC1=C(C=CC=C1)C1=NCC2=NN=C(N2C2=CC=C(C=C12)[N+](=O)[O-])C
Fluorexetamine	C14H18FNO	235.1367	C(C)NC1(C(CCCC1)=O)C1=CC(=CC=C1)F
Fluoropentyl-norcarfentanil	C21H31FN2O3	378.2313	FCCCCCN1CCC(CC1)(C(=O)OC)N(C(CC)=O)C1=CC=CC=C1
Fluorphine	C20H22FN3O	339.1741	FC1=CC=C(C=C1)C(C)N1CCC(CC1)N1C(NC2=C1C=CC=C2)=O
Flurazepam	C21H23ClFN3O	387.1508	ClC=1C=CC2=C(C(=NCC(N2CCN(CC)CC)=O)C2=C(C=CC=C2)F)C1
Flutazolam	C19H18ClFN2O3	376.0984	ClC=1C=CC2=C(C3(N(CC(N2CCO)=O)CCO3)C3=C(C=CC=C3)F)C1
Flutemazepam	C16H12ClFN2O2	318.0566	ClC=1C=CC2=C(C(=NC(C(N2C)=O)O)C2=C(C=CC=C2)F)C1
Flutoprazepam	C19H16ClFN2O	342.0930	ClC=1C=CC2=C(C(=NCC(N2CC2CC2)=O)C2=C(C=CC=C2)F)C1
Fonazepam	C15H10FN3O3	299.0701	O=C1CN=C(C2=CC=CC=C2F)C3=CC([N+]([O-])=O)=CC=C3N1
Formetorex	C10H13NO	163.0992	O=CNC(C)CC1=CC=CC=C1
Fosazepam	C18H18ClN2O2P	360.0789	O=C1N(CP(C)(C)=O)C2=CC=C(Cl)C=C2C(C3=CC=CC=C3)=NC1
FUB-144	C23H24FNO	349.1837	FC1=CC=C(CN2C=C(C3=CC=CC=C23)C(=O)C2C(C2(C)C)(C)C)C=C1
FUBIAT	C17H14FNO2	283.1003	FC1=CC=C(C=C1)CN1C=C(C2=CC=CC=C12)CC(=O)O
FUBIMINA	C23H21FN2O	360.1633	O=C(C1=NC2=CC=CC=C2N1CCCCCF)C3=C4C=CC=CC4=CC=C3
FUBIMINA N-(5-Hydroxypentyl) Metabolite	C23H22N2O2	358.1676	O=C(C1=NC2C=CC=CC2N1CCCCCO)c1cccc2ccccc12
FUBIMINA N-Pentanoic Acid	C23H20N2O3	372.1468	O=C(O)CCCCN1C(C(C2=C3C=CC=CC3=CC=C2)=O)=NC4=CC=CC=C14
FUB-JWH-018	C26H18FNO	379.1367	O=C(C1=CC=CC2=C1C=CC=C2)C3=CN(CC4=CC=C(F)C=C4)C5=CC=CC=C53
FUB-NPB-22	C24H16FN3O2	397.1221	O=C(C1=NN(CC2=CC=C(F)C=C2)C3=C1C=CC=C3)OC4=C5N=CC=CC5=CC=C4
FUB-PB-22	C25H17FN2O2	396.1269	O=C(C1=CN(CC2=CC=C(F)C=C2)C3=C1C=CC=C3)OC4=C5N=CC=CC5=CC=C4
FUB-PB-22 3-Carboxyindole Metabolite	C16H12FNO2	269.0847	O=C(O)c1cn(Cc2ccc(F)cc2)c2ccccc12
Furanyl UF-17	C19H24N2O2	312.1832	O=C(C1=CC=CO1)N(C2C(N(C)C)CCCC2)C3=CC=CC=C3
G-130	C12H17NO	191.1305	CC1(C)COC(C2=CC=CC=C2)CN1
Gabapentin	C9H17NO2	171.1254	O=C(O)CC1(CN)CCCCC1

Table S9. Compound database imported into the *Search Mass Lists* node (continued)			
Compound Name	Molecular Formula	Exact Mass	SMILES
Gacyclidine	C16H25NS	263.1702	C[C@@H]1[C@](CCCC1)(C=1SC=CC1)N1CCCCC1
Gidazepam	C17H15BrN4O2	386.0373	BrC1=CC2=C(C=C1)N(CC(NN)=O)C(CN=C2C3=CC=CC=C3)=O
Glaucine	C21H25NO4	355.1778	CN1CCC2=CC(OC)=C(OC)C3=C2[C@]1([H])CC4=CC(OC)=C(OC)C=C34
Glycinexylidide	C10H14N2O	178.1101	CC1=C(C(=CC=C1)C)NC(=O)CN
GTS-21	C19H20N2O2	308.1519	COC1=CC=C(/C=C2C(C3=CC=CN=C3)=NCCC/2)C(OC)=C1
Halazepam	C17H12ClF3N2O	352.0585	ClC=1C=CC2=C(C(=NCC(N2CC(F)(F)F)=O)C2=CC=CC=C2)C1
Haloxazolam	C17H14BrFN2O2	376.0217	BrC=1C=CC2=C(C3(N(CC(N2)=O)CCO3)C3=C(C=CC=C3)F)C1
Harmaline	C13H14N2O	214.1101	CC1=NCCC2=C1NC3=C2C=CC(OC)=C3
Harmine	C13H12N2O	212.0944	CC1=NC=CC2=C1NC3=C2C=CC(OC)=C3
HDEP-28	C19H23NO2	297.1723	O=C(OCC)C(C1=CC=C2C=CC=CC2=C1)C3NCCCC3
HDMP-28	C18H21NO2	283.1567	O=C(OC)C(C1=CC=C2C=CC=CC2=C1)C3NCCCC3
Hexahydrocannabiphorol	C23H36O2	344.2710	C(CCCCCC)C=1C=C(C(=2[C@H]3[C@H](C(OC2C1)(C)C)CC[C@@H](C3)C)O
Hexedrone	C13H19NO	205.1461	CCCCC(NC)C(C1=CC=CC=C1)=O
HMA	C10H15NO2	181.1097	OC1=CC=C(CC(N)C)C=C1OC
Homarylamine	C10H13NO2	179.0941	CNCCC1=CC=C(OCO2)C2=C1
Hordenine	C10H15NO	165.1148	OC1=CC=C(CCN(C)C)C=C1
HOT-2	C12H19NO3S	257.1080	C(C)SC1=CC(=C(C=C1OC)CCNO)OC
HOT-7	C13H21NO3S	271.1237	COC1=C(C=C(C(=C1)SCCC)OC)CCNO
HU-210	C25H38O3	386.2816	OC1=CC(C(CCCCCC)(C)C)=CC2=C1[C@@](CC(CO)=CC3)([H])[C@]3([H])C(C)(C)O2
HU-308	C27H42O3	414.3129	OCC1=C[C@@H](C2=C(OC)C=C(C(CCCCCC)(C)C)C=C2OC)[C@@]3([H])C[C@]1([H])C3(C)C
HU-331	C21H28O3	328.2033	O=C(C(CCCCC)=C1)C(O)=C([C@@H]2C=C(C)CCC2C(C)=C)C1=O
Hydroxetamine	C14H19NO2	233.1410	C(C)NC1(C(CCCC1)=O)C1=CC(=CC=C1)O
Hydroxybupropion	C13H18ClNO2	255.1021	CC(NC(C)(C)CO)C(=O)C1=CC(Cl)=CC=C1
Hydroxy-THC	C21H30O3	330.2190	CCCCCC1=CC(=C2C3C=C(CCC3C(OC2=C1)(C)C)CO)O
Ibogaine	C20H26N2O	310.2040	COC1=CC2=C(NC3=C2CCN4C5C3CC(C4)CC5CC)C=C1
Iclazepam	C21H21ClN2O2	368.1286	ClC=1C=CC2=C(C(=NCC(N2CCOCC2CC2)=O)C2=CC=CC=C2)C1
IDRA-21	C8H9ClN2O2S	232.0068	CC(NC1=CC=C(Cl)C=C12)NS2(=O)=O
Indane	C9H10	118.0777	C12=C(CCC2)C=CC=C1
Iodorphine	C20H22IN3O	447.0802	CC(C1=CC=C(I)C=C1)N2CCC(N3C(NC4=C3C=CC=C4)=O)CC2
iPDMB-FUBINACA	C24H28FN3O3	425.2109	FC1=CC=C(CN2N=C(C3=CC=CC=C23)C(=O)N[C@H](C(=O)OC(C)C)C(C)(C)C)C=C1
IPO-33	C20H22N2O	306.1727	C(CCCC)N1N=C(C2=CC=CC=C12)C(CC1=CC=CC=C1)=O
iPRONE	C13H17NO3	235.1203	CC(NC(C)C)C(C1=CC=C(OCO2)C2=C1)=O
Iso-(meta-methyl-propcathinone)	C13H19NO	205.1461	CC=1C=C(C=CC1)C(C(C)=O)NCCC
Iso-(metamethyl-propcathinone)	C13H19NO	205.1461	NC(C(=O)C=1C=C(C=CC1)C)CCCC
Iso-3-CMC	C10H12ClNO	197.0602	ClC=1C=C(C=CC1)C(C(C)=O)NC
Iso-butonitazene	C24H32N4O3	424.2469	C(C)N(CCN1C(=NC2=C1C=CC(=C2)[N+](=O)[O-])CC2=CC=C(C=C2)OCC(C)C)CC
Isobutyl-pentyl-1H-indazole-3-carboxylate	C17H24N2O2	288.1832	CCCCCn1nc(C(=O)OCC(C)C)c2ccccc21
Isobutyryl Norfentanyl	C15H22N2O	246.1727	CC(C)C(N(C1=CC=CC=C1)C2CCNCC2)=O
Isocarfentanil	C24H30N2O3	394.2251	C1(=CC=CC=C1)CCN1CC(C(CC1)N(C(CC)=O)C1=CC=CC=C1)C(=O)OC
Iso-ethcathinone	C11H15NO	177.1148	CC(C(NCC)C1=CC=CC=C1)=O
Isohexedrone	C13H19NO	205.1461	CC(CC(C(=O)C1=CC=CC=C1)NC)C
Isohexylone	C14H19NO3	249.1360	CC(C)CC(NC)C(C1=CC=C(OCO2)C2=C1)=O

Table S9. Compound database imported into the *Search Mass Lists* node (continued)

Compound Name	Molecular Formula	Exact Mass	SMILES
Isopentedrone	C12H17NO	191.1305	CCCC(C(NC)C1=CC=CC=C1)=O
Isophenmetrazine	C11H15NO	177.1148	CC1COC(C2=CC=CC=C2)CN1
Isopropyl U-47700	C18H26Cl2N2O	356.1417	ClC=1C=C(C(=O)N(C(C)C)C2C(CCCC2)N(C)C)C=CC1Cl
Isopropylphenidate	C16H23NO2	261.1723	C1(=CC=CC=C1)C(C(=O)OC(C)C)C2CCCCN2
Isotodesnitazene	C23H31N3O	365.2462	C(C)N(CCN1C(=NC2=C1C=CC=C2)CC2=CC=C(C=C2)OC(C)C)CC
JTE-907	C24H26N2O6	438.1785	O=C(C1=CC2=C(NC1=O)C(OCCCCC)=C(OC)C=C2)NCC3=CC=C(OCO4)C4=C3
JWH 018 N-(5-Hydroxypentyl) Metabolite	C24H23NO2	357.1723	O=C(C1=CN(CCCCCO)C2=C1C=CC=C2)C3=C4C=CC=CC4=CC=C3
JWH 019 N-(5-Hydroxyhexyl) Metabolite	C25H25NO2	371.1880	O=C(c1cccc2ccccc12)c1cn(CCCCCCO)c2ccccc12
JWH 073 4-Hydroxyindole Metabolite	C23H21NO2	343.1567	O=C(C1=CN(CCCC)C2=C1C(O)=CC=C2)C3=C4C=CC=CC4=CC=C3
JWH 073 5-Hydroxyindole Metabolite	C23H21NO2	343.1567	O=C(C1=CN(CCCC)C2=C1C=C(O)C=C2)C3=C4C=CC=CC4=CC=C3
JWH 073 N-(3-Hydroxybutyl) Metabolite	C23H21NO2	343.1567	O=C(C1=CN(CCC(O)C)C2=C1C=CC=C2)C3=C4C=CC=CC4=CC=C3
JWH 073 N-(4-Hydroxybutyl) Metabolite	C23H21NO2	343.1567	O=C(C1=CN(CCCCO)C2=C1C=CC=C2)C3=C4C=CC=CC4=CC=C3
JWH 073 N-Butanoic Acid Metabolite	C23H19NO3	357.1360	O=C(O)CCCN1C=C(C(C2=C3C=CC=CC3=CC=C2)=O)C4=C1C=CC=C4
JWH 122 N-(5-Hydroxypentyl) Metabolite	C25H25NO2	371.1880	O=C(C1=CN(CCCCCO)C2=C1C=CC=C2)C3=C4C=CC=CC4=C(C)C=C3
JWH 184	C25H27N	341.2138	CC1=CC=C(C2=CC=CC=C12)CC1=CN(C2=CC=CC=C12)CCCCC
JWH 210 N-(4-Hydroxypentyl) Metabolite	C26H27NO2	385.2036	CCc1ccc(C(=O)c2cn(CCCC(C)O)c3ccccc23)c2ccccc12
JWH 250 N-(4-Hydroxypentyl) Metabolite	C22H25NO3	351.1829	OC(CCCN1C=C(C2=CC=CC=C12)C(CC1=C(C=CC=C1)OC)=O)C
JWH 307 3-Isomer	C26H24FNO	385.1837	O=C(C1=CN(CCCCC)C=C1C2=CC=CC=C2F)C3=C4C=CC=CC4=CC=C3
JWH 412 N-(5-Hydroxypentyl) Metabolite	C24H22FNO2	375.1629	O=C(C1=C2C=CC=CC2=C(F)C=C1)C3=CN(CCCCCO)C4=C3C=CC=C4
JWH-004	C26H27NO	369.2087	O=C(C1=C(C)N(CCCCCC)C2=C1C=CC=C2)C3=C4C=CC=CC4=CC=C3
JWH-007	C25H25NO	355.1931	CC=1N(C2=CC=CC=C2C1C(=O)C1=CC=CC2=CC=CC=C12)CCCCC
JWH-007 (5-OH-Pentyl)	C25H25NO2	371.1880	O=C(C1=C(C)N(CCCCCO)C2=C1C=CC=C2)C3=C4C=CC=CC4=CC=C3
JWH-007 (N-Pentanoic Acid)	C25H23NO3	385.1673	C1(=CC=CC2=CC=CC=C12)C(=O)C1=C(N(C2=CC=CC=C12)CCCCC(=O)O)C
JWH-011	C27H29NO	383.2244	CCCCCC(C)N1C(C)=C(C(=O)C2=CC=CC3=CC=CC=C23)C2=CC=CC=C12
JWH-015	C23H21NO	327.1618	O=C(C1=C(C)N(CCC)C2=C1C=CC=C2)C3=C4C=CC=CC4=CC=C3
JWH-016	C24H23NO	341.1774	O=C(C1=C(C)N(CCCC)C2=C1C=CC=C2)C3=C4C=CC=CC4=CC=C3
JWH-018	C24H23NO	341.1774	CCCCCN1C=C(C(C2=C3C=CC=CC3=CC=C2)=O)C4=C1C=CC=C4
JWH-018 (2-OH-Indole)	C24H23NO2	357.1723	O=C(C1=C(O)N(CCCCC)C2=C1C=CC=C2)C3=C4C=CC=CC4=CC=C3
JWH-018 (2-OH-Pentyl)	C24H23NO2	357.1723	OC(CN1C=C(C2=CC=CC=C12)C(=O)C1=CC=CC2=CC=CC=C12)CCC
JWH-018 (3-OH-Pentyl)	C24H23NO2	357.1723	OC(CCN1C=C(C2=CC=CC=C12)C(=O)C1=CC=CC2=CC=CC=C12)CC
JWH-018 (4-OH-Indole)	C24H23NO2	357.1723	O=C(C1=CN(CCCCC)C2=C1C(O)=CC=C2)C3=C4C=CC=CC4=CC=C3
JWH-018 (4-oxo-Pentyl)	C24H21NO2	355.1567	C1(=CC=CC2=CC=CC=C12)C(=O)C1=CN(C2=CC=CC=C12)CCCC(C)=O
JWH-018 (5-OH-Indole)	C24H23NO2	357.1723	O=C(C1=CN(CCCCC)C2=C1C=C(O)C=C2)C3=C4C=CC=CC4=CC=C3
JWH-018 (5-OH-Pentyl-glucuronide)	C30H31NO8	533.2044	C1(=CC=CC2=CC=CC=C12)C(=O)C1=CN(C2=CC=CC=C12)CCCCCO[C@H]1[C@@H]([C@H]([C@@H]([C@H](O1)C(=O)O)O)O)O
JWH-018 (6-OH-Indole)	C24H23NO2	357.1723	O=C(C1=CN(CCCCC)C2=C1C=CC(O)=C2)C3=C4C=CC=CC4=CC=C3
JWH-018 (7-OH-Indole)	C24H23NO2	357.1723	O=C(C1=CN(CCCCC)C2=C1C=CC=C2O)C3=C4C=CC=CC4=CC=C3
JWH-018 6-Methoxyindole Analogue	C25H25NO2	371.1880	CCCCCN1C2=CC(OC)=CC=C2C(C(C3=CC=CC4=C3C=CC=C4)=O)=C1
JWH-018 8-Quinolinyl Carboxamide	C23H23N3O	357.1836	CCCCCN1C=C(C(O)=NC2=CC=CC3=C2N=CC=C3)C2=CC=CC=C12
JWH-018 Benzimidazole Analogue	C23H22N2O	342.1727	O=C(C1=C(C=CC=C2)C2=CC=C1)C3=NC4=C(C=CC=C4)N3CCCCC
JWH-018 Methylderivate (2-Methylnaphthalene)	C25H25NO	355.1931	O=C(C1=C2C=CC=CC2=CC=C1C)C3=CN(CCCCC)C4=C3C=CC=C4
JWH-018 N-(1,1-Dimethylpropyl) Isomer	C24H23NO	341.1774	O=C(C1=C2C=CC=CC2=CC=C1)C3=CN(C(C)(C)CC)C4=C3C=CC=C4
JWH-018 N-(4,5-Epoxypentyl) Analogue	C24H21NO2	355.1567	O=C(C1=CN(CCCC2OC2)C3=C1C=CC=C3)C4=C5C(C=CC=C5)=CC=C4

**Table S9.** Compound database imported into the *Search Mass Lists* node (continued)			
Compound Name	Molecular Formula	Exact Mass	SMILES
JWH-018 N-(4-Hydroxypentyl) Metabolite	C24H23NO2	357.1723	O=C(C1=CN(CCCC(O)C)C2=C1C=CC=C2)C3=C4C=CC=CC4=CC=C3
JWH-018 N-(5-Bromopentyl) Analogue	C24H22BrNO	419.0879	O=C(C1=CC=CC2=C1C=CC=C2)C3=CN(CCCCCBr)C4=CC=CC=C43
JWH-018 N-(5-Chloropentyl) Analogue	C24H22ClNO	375.1385	O=C(C1=CN(CCCCCCl)C2=C1C=CC=C2)C3=C4C(C=CC=C4)=CC=C3
JWH-018 N-Pentanoic Acid	C24H21NO3	371.1516	C1(=CC=CC2=CC=CC=C12)C(=O)C1=CN(C2=CC=CC=C12)CCCCC(=O)O
JWH-018-1-methyl-hexyl	C26H27NO	369.2087	O=C(C1=CN(C(CCCCC)C)C2=C1C=CC=C2)C3=C4C=CC=CC4=CC=C3
JWH-019	C25H25NO	355.1931	O=C(C1=CN(CCCCCC)C2=C1C=CC=C2)C3=C4C=CC=CC4=CC=C3
JWH-019 (5-OH-Indole)	C25H25NO2	371.1880	C(CCCCC)N1C=C(C2=CC(=CC=C12)O)C(=O)C1=CC=CC2=CC=CC=C12
JWH-019 (6-OH-Hexyl)	C25H25NO2	371.1880	O=C(C1=CN(CCCCCCO)C2=C1C=CC=C2)C3=C4C=CC=CC4=CC=C3
JWH-020	C26H27NO	369.2087	O=C(C1=CN(CCCCCCC)C2=C1C=CC=C2)C3=C4C=CC=CC4=CC=C3
JWH-022	C24H21NO	339.1618	O=C(C1=C2C=CC=CC2=CC=C1)C3=CN(CCCC=C)C4=C3C=CC=C4
JWH-030	C20H21NO	291.1618	O=C(C1=C2C=CC=CC2=CC=C1)C3=CN(CCCCC)C=C3
JWH-031	C21H23NO	305.1774	CCCCCCN1C=CC(=C1)C(=O)C1=CC=CC2=CC=CC=C12
JWH-047	C25H25NO	355.1931	O=C(C1=C(C)N(CCCC)C2=C1C=CC=C2)C3=C4C=C(C)C=CC4=CC=C3
JWH-048	C26H27NO	369.2087	O=C(C1=C(C)N(CCCCC)C2=C1C=CC=C2)C3=C4C=C(C)C=CC4=CC=C3
JWH-049	C27H29NO	383.2244	O=C(C1=C(C)N(CCCCCC)C2=C1C=CC=C2)C3=C4C=C(C)C=CC4=CC=C3
JWH-050	C28H31NO	397.2400	O=C(C1=C(C)N(CCCCCCC)C2=C1C=CC=C2)C3=C4C=C(C)C=CC4=CC=C3
JWH-071	C21H17NO	299.1305	CCn1cc(-c2ccc3ccccc3c2C=O)c2ccccc21
JWH-072	C22H19NO	313.1461	O=C(C1=C2C=CC=CC2=CC=C1)C3=CN(CCC)C4=C3C=CC=C4
JWH-072 (Acid)	C22H17NO3	343.1203	O=C(O)CCN1C=C(C(C2=C3C=CC=CC3=CC=C2)=O)C4=C1C=CC=C4
JWH-073	C23H21NO	327.1618	CCCCN1C=C(C2=CC=CC=C21)C(=O)C3=CC=CC4=CC=CC=C43
JWH-073 (2-Methylbutyl) Homolog	C24H23NO	341.1774	CC(CN1C=C(C2=CC=CC=C12)C(=O)C1=CC=CC2=CC=CC=C12)CC
JWH-073 (6-OH-Indole)	C23H21NO2	343.1567	O=C(C1=CN(CCCC)C2=C1C=CC(O)=C2)C3=C4C=CC=CC4=CC=C3
JWH-073 2-Methylnaphthyl Analogue	C24H23NO	341.1774	CCCCN1C=C(C2=CC=CC=C21)C(=O)C3=C(C=CC4=CC=CC=C43)C
JWH-073 4-Methylnaphthyl Analogue	C24H23NO	341.1774	O=C(C1=CN(CCCC)C2=C1C=CC=C2)C3=CC=C(C)C4=CC=CC=C43
JWH-073 6-Methoxyindole Analogue	C24H23NO2	357.1723	O=C(C1=CN(CCCC)C2=C1C=CC(OC)=C2)C3=CC=CC4=C3C=CC=C4
JWH-073-3-Methyl	C24H23NO	341.1774	O=C(C1=CN(CCC(C)C)C2=C1C=CC=C2)C3=C4C=CC=CC4=CC=C3
JWH-073-M-2-OH-Ind	C23H21NO2	343.1567	O=C(C1=C(O)N(CCCC)C2=C1C=CC=C2)C3=C4C=CC=CC4=CC=C3
JWH-073-M-7-OH-Ind	C23H21NO2	343.1567	O=C(C1=CN(CCCC)C2=C1C=CC=C2O)C3=C4C=CC=CC4=CC=C3
JWH-079	C23H21NO2	343.1567	O=C(C1=C2C=CC=CC2=C(OC)C=C1)C3=CN(CCC)C4=C3C=CC=C4
JWH-080	C24H23NO2	357.1723	O=C(C1=CN(CCCC)C2=C1C=CC=C2)C3=C4C=CC=CC4=C(OC)C=C3
JWH-081	C25H25NO2	371.1880	COC1=CC=C(C2=CC=CC=C12)C(=O)C1=CN(C2=CC=CC=C12)CCCCC
JWH-081 (4-OH-Naphthyl)	C24H23NO2	357.1723	OC1=CC=C(C2=CC=CC=C12)C(=O)C1=CN(C2=CC=CC=C12)CCCCC
JWH-081 (4-OH-Pentyl)	C25H25NO3	387.1829	OC(CCCN1C=C(C2=CC=CC=C12)C(=O)C1=CC=C(C2=CC=CC=C12)OC)C
JWH-081 (5-OH-Pentyl)	C25H25NO3	387.1829	OCCCCCN1C=C(C2=CC=CC=C12)C(=O)C1=CC=C(C2=CC=CC=C12)OC
JWH-081 (Pentanoic Acid)	C25H23NO4	401.1622	O=C(O)CCCCN1C=C(C(C2=C3C=CC=CC3=C(OC)C=C2)=O)C4=C1C=CC=C4
JWH-081 N-(Cyclohexylmethyl) Analogue	C27H27NO2	397.2036	O=C(C1=CN(CC2CCCCC2)C3=C1C=CC=C3)C4=C5C=CC=CC5=C(OC)C=C4
JWH-082	C26H27NO2	385.2036	O=C(C1=CN(CCCCCC)C2=C1C=CC=C2)C3=C4C=CC=CC4=C(OC)C=C3
JWH-098	C26H27NO2	385.2036	O=C(C1=C2C=CC=CC2=C(OC)C=C1)C3=C(C)N(CCCCC)C4=C3C=CC=C4
JWH-116	C26H27NO	369.2087	CCCCCn1c(CC)c(C(=O)c2cccc3ccccc23)c2ccccc21
JWH-122	C25H25NO	355.1931	O=C(C1=C2C=CC=CC2=C(C)C=C1)C3=CN(CCCCC)C4=C3C=CC=C4
JWH-122 (2-OH-Pentyl)	C25H25NO2	371.1880	CC1=CC=C(C2=CC=CC=C12)C(=O)C1=CN(C2=CC=CC=C12)CC(CCC)O
JWH-122 (3-OH-Pentyl)	C25H25NO2	371.1880	CC1=CC=C(C2=CC=CC=C12)C(=O)C1=CN(C2=CC=CC=C12)CCC(CC)O

**Table S9.** Compound database imported into the *Search Mass Lists* node (continued)			
Compound Name	Molecular Formula	Exact Mass	SMILES
JWH-122 (4-OH-Pentyl)	C25H25NO2	371.1880	OC(CCCN1C=C(C2=CC=CC=C12)C(=O)C1=CC=C(C2=CC=CC=C12)C)C
JWH-122 (5-OH-Indole)	C25H25NO2	371.1880	CC1=CC=C(C2=CC=CC=C12)C(=O)C1=CN(C2=CC=C(C=C12)O)CCCCC
JWH-122 (6-OH-Indole)	C25H25NO2	371.1880	CC1=CC=C(C2=CC=CC=C12)C(=O)C1=CN(C2=CC(=CC=C12)O)CCCCC
JWH-122 N-(4-Pentenyl) Analogue	C25H23NO	353.1774	CC1=CC=C(C2=CC=CC=C12)C(=O)C3=CN(C4=CC=CC=C43)CCCC=C
JWH-122 N-(5-Chloropentyl) Derivative	C25H24ClNO	389.1541	O=C(C1=CN(CCCCCCl)C2=C1C=CC=C2)C3=C4C=CC=CC4=C(C)C=C3
JWH-122 Pentenyl 2-Methylindole Derivative	C26H25NO	367.1931	O=C(C1=C(C)N(CCCC=C)C2=C1C=CC=C2)C3=C4C=CC=CC4=C(C)C=C3
JWH-133	C22H32O	312.2448	[H][C@@]12CC(C)=CC[C@@]1([H])C(C)(C)OC1=C2C=CC(=C1)C(C)(C)CCC
JWH-145	C26H25NO	367.1931	O=C(C1=C2C=CC=CC2=CC=C1)C3=CN(CCCCC)C(C4=CC=CC=C4)=C3
JWH-146	C28H29NO	395.2244	O=C(C1=CN(CCCCCCC)C(C2=CC=CC=C2)=C1)C3=C4C=CC=CC4=CC=C3
JWH-147	C27H27NO	381.2087	O=C(C1=CN(CCCCCC)C(C2=CC=CC=C2)=C1)C3=C4C=CC=CC4=CC=C3
JWH-149	C26H27NO	369.2087	O=C(C1=C2C=CC=CC2=C(C)C=C1)C3=C(C)N(CCCCC)C4=C3C=CC=C4
JWH-150	C25H23NO	353.1774	O=C(C1=CN(CCCC)C(C2=CC=CC=C2)=C1)C3=C4C=CC=CC4=CC=C3
JWH-166	C25H25NO2	371.1880	O=C(C1=C2C=CC(OC)=CC2=CC=C1)C3=CN(CCCCC)C4=C3C=CC=C4
JWH-167	C21H23NO	305.1774	CCCCCN1C=C(C(CC2=CC=CC=C2)=O)C3=C1C=CC=C3
JWH-175	C24H25N	327.1982	CCCCCN1C=C(CC2=CC=CC3=CC=CC=C23)C2=CC=CC=C12
JWH-176	C25H24	324.1873	[H]\C(=C1/C=C(CCCCC)C2=CC=CC=C12)C1=CC=CC2=CC=CC=C12
JWH-180	C25H25NO	355.1931	O=C(C1=CN(CCC)C2=C1C=CC=C2)C3=C4C=CC=CC4=C(CCC)C=C3
JWH-181	C28H31NO	397.2400	O=C(C1=C(C)N(CCCCC)C2=C1C=CC=C2)C3=C4C=CC=CC4=C(CCC)C=C3
JWH-182	C27H29NO	383.2244	O=C(C1=CN(CCCCC)C2=C1C=CC=C2)C3=C4C=CC=CC4=C(CCC)C=C3
JWH-189	C26H27NO	369.2087	O=C(C1=C(C)N(CCC)C2=C1C=CC=C2)C3=C4C=CC=CC4=C(CCC)C=C3
JWH-193	C26H26N2O2	398.1989	O=C(C1=C2C=CC=CC2=C(C)C=C1)C3=CN(CCN4CCOCC4)C5=C3C=CC=C5
JWH-198	C26H26N2O3	414.1938	O=C(C1=CN(CCN2CCOCC2)C3=C1C=CC=C3)C4=C5C=CC=CC5=C(OC)C=C4
JWH-200	C25H24N2O2	384.1832	O=C(C1=CC=CC2=C1C=CC=C2)C3=CN(C4=C3C=CC=C4)CCN5CCOCC5
JWH-200 (5-OH-Indole)	C25H24N2O3	400.1781	OC=1C=C2C(=CN(C2=CC1)CCN1CCOCC1)C(=O)C1=CC=CC2=CC=CC=C12
JWH-200 (6-OH-Indole)	C25H24N2O3	400.1781	OC1=CC=C2C(=CN(C2=C1)CCN1CCOCC1)C(=O)C1=CC=CC2=CC=CC=C12
JWH-200 Analogue	C22H30N2O2	354.2302	O=C(/C=C(C(C(C)(C)C)\C)C1=CN(CCN2CCOCC2)C3=CC=CC=C31
JWH-200-M-4-OH-Ind	C25H24N2O3	400.1781	O=C(C1=CN(CCN2CCOCC2)C3=C1C(O)=CC=C3)C4=C5C=CC=CC5=CC=C4
JWH-201	C22H25NO2	335.1880	CCCCCN1C=C(C(CC2=CC=C(OC)C=C2)=O)C3=C1C=CC=C3
JWH-203	C21H22ClNO	339.1385	CCCCCN1C=C(C(CC2=CC=CC=C2Cl)=O)C3=C1C=CC=C3
JWH-203 (4-OH-Pentyl)	C21H22ClNO2	355.1334	ClC1=C(C=CC=C1)CC(=O)C1=CN(C2=CC=CC=C12)CCCC(C)O
JWH-203 (5-OH-Pentyl)	C21H22ClNO2	355.1334	O=C(C1=CN(CCCCCO)C2=C1C=CC=C2)CC3=CC=CC=C3Cl
JWH-203 N-Pentanoic Acid Metabolite	C21H20ClNO3	369.1126	ClC1=C(C=CC=C1)CC(=O)C1=CN(C2=CC=CC=C12)CCCCC(=O)O
JWH-204	C22H24ClNO	353.1541	CCCCCN1C(C)=C(C(CC2=CC=CC=C2Cl)=O)C3=C1C=CC=C3
JWH-210	C26H27NO	369.2087	C(C)C1=CC=C(C2=CC=CC=C12)C(=O)C1=CN(C2=CC=CC=C12)CCCCC
JWH-210 (5-OH-Indole)	C26H27NO2	385.2036	C(C)C1=CC=C(C2=CC=CC=C12)C(=O)C1=CN(C2=CC=C(C=C12)O)CCCCC
JWH-210 (5-OH-Pentyl)	C26H27NO2	385.2036	O=C(C1=C2C=CC=CC2=C(CC)C=C1)C3=CN(CCCCCO)C4=C3C=CC=C4
JWH-210 N-Pentanoic Acid	C26H25NO3	399.1829	C(C)C1=CC=C(C2=CC=CC=C12)C(=O)C1=CN(C2=CC=CC=C12)CCCCC(=O)O
JWH-211	C25H25NO	355.1931	O=C(C1=C2C=CC=CC2=C(CC)C=C1)C3=C(C)N(CCC)C4=C3C=CC=C4
JWH-212	C24H23NO	341.1774	O=C(C1=C2C=CC=CC2=C(CC)C=C1)C3=CN(CCC)C4=C3C=CC=C4
JWH-213	C27H29NO	383.2244	O=C(C1=C2C=CC=CC2=C(CC)C=C1)C3=C(C)N(CCCCC)C4=C3C=CC=C4
JWH-234	C26H27NO	369.2087	C(C)C1=CC=C2C=CC=C(C2=C1)C(=O)C1=CN(C2=CC=CC=C12)CCCCC
JWH-240	C28H31NO	397.2400	O=C(C1=C2C=CC=CC2=C(CCCC)C=C1)C3=CN(CCCCC)C4=C3C=CC=C4

Table S9. Compound database imported into the *Search Mass Lists* node (continued)

Compound Name	Molecular Formula	Exact Mass	SMILES
JWH-242	C29H33NO	411.2557	O=C(C1=C2C=CC=CC2=C(CCCC)C=C1)C3=C(C)N(CCCCC)C4=C3C=CC=C4
JWH-246	C26H24ClNO	401.1541	O=C(C1=CN(CCCCC)C(C2=CC=CC(Cl)=C2)=C1)C3=C4C=CC=CC4=CC=C3
JWH-249	C21H22BrNO	383.0879	CCCCCN1C=C(C(CC2=CC=CC=C2Br)=O)C3=C1C=CC=C3
JWH-250	C22H25NO2	335.1880	C(CCCC)N1C=C(C2=CC=CC=C12)C(CC1=C(C=CC=C1)OC)=O
JWH-250 (5-OH-Indole)	C22H25NO3	351.1829	OC=1C=C2C(=CN(C2=CC1)CCCCC)C(CC1=C(C=CC=C1)OC)=O
JWH-250 (5-OH-Pentyl)	C22H25NO3	351.1829	O=C(C1=CN(CCCCCO)C2=C1C=CC=C2)CC3=CC=CC=C3OC
JWH-250 N-Pentanoic Acid	C22H23NO4	365.1622	COC1=C(C=CC=C1)CC(=O)C1=CN(C2=CC=CC=C12)CCCCC(=O)O
JWH-251	C22H25NO	319.1931	CCCCCN1C=C(C(CC2=CC=CC=C2C)=O)C3=C1C=CC=C3
JWH-252	C23H27NO	333.2087	CC1=CC=CC=C1CC(C2=C(C)N(CCCCC)C3=C2C=CC=C3)=O
JWH-253	C23H27NO2	349.2036	CCCCCN1C(C)=C(C(CC2=CC=CC(OC)=C2)=O)C3=C1C=CC=C3
JWH-267	C25H25NO2	371.1880	COC1=C(C2=CC=CC=C2C=C1)C(=O)C1=CN(C2=CC=CC=C12)CCCCC
JWH-292	C27H27NO2	397.2036	O=C(C1=CN(CCCCC)C(C2=CC=CC=C2OC)=C1)C3=C4C=CC=CC4=CC=C3
JWH-302	C22H25NO2	335.1880	CCCCCN1C=C(C(CC2=CC=CC(OC)=C2)=O)C3=C1C=CC=C3
JWH-305	C22H24BrNO	397.1036	CCCCCN1C(C)=C(C(CC2=CC=CC=C2Br)=O)C3=C1C=CC=C3
JWH-307	C26H24FNO	385.1837	O=C(C1=CN(CCCCC)C(C2=CC=CC=C2F)=C1)C3=C4C=CC=CC4=CC=C3
JWH-307 Brominated Analogue	C26H24BrNO	445.1036	O=C(C1=CN(CCCCC)C(C2=CC=CC=C2Br)=C1)C3=C4C=CC=CC4=CC=C3
JWH-308	C26H24FNO	385.1837	CCCCCN1C(C2=CC=C(F)C=C2)=CC(C(C3=C4C=CC=CC4=CC=C3)=O)=C1
JWH-309	C30H27NO	417.2087	CCCCCN1C(C2=C3C=CC=CC3=CC=C2)=CC(C(C4=C5C=CC=CC5=CC=C4)=O)=C1
JWH-311	C21H22FNO	323.1680	CCCCCN1C=C(C(CC2=CC=CC=C2F)=O)C3=C1C=CC=C3
JWH-314	C22H24FNO	337.1837	CCCCCN1C(C)=C(C(CC2=CC=CC=C2F)=O)C3=C1C=CC=C3
JWH-364	C28H29NO	395.2244	O=C(C1=CN(CCCCC)C(C2=CC=C(CC)C=C2)=C1)C3=C4C=CC=CC4=CC=C3
JWH-368	C26H24FNO	385.1837	O=C(C1=CN(CCCCC)C(C2=CC=CC(F)=C2)=C1)C3=C4C=CC=CC4=CC=C3
JWH-369	C26H24ClNO	401.1541	CCCCCN1C(C2=CC=CC=C2Cl)=CC(C(C3=C4C=CC=CC4=CC=C3)=O)=C1
JWH-370	C27H27NO	381.2087	O=C(C1=CN(CCCCC)C(C2=CC=CC=C2C)=C1)C3=C4C=CC=CC4=CC=C3
JWH-371	C30H33NO	423.2557	O=C(C1=CN(CCCCC)C(C2=CC=C(CCCC)C=C2)=C1)C3=C4C=CC=CC4=CC=C3
JWH-386	C22H18BrNO	391.0566	O=C(C1=C2C=CC=CC2=C(Br)C=C1)C3=CN(CCC)C4=C3C=CC=C4
JWH-387	C24H22BrNO	419.0879	O=C(C1=C2C=CC=CC2=C(Br)C=C1)C3=CN(CCCCC)C4=C3C=CC=C4
JWH-394	C25H24BrNO	433.1036	O=C(C1=C(C)N(CCCCC)C2=C1C=CC=C2)C3=C4C=CC=CC4=C(Br)C=C3
JWH-397	C25H24ClNO	389.1541	O=C(C1=C2C=CC=CC2=C(Cl)C=C1)C3=C(C)N(CCCCC)C4=C3C=CC=C4
JWH-398	C24H22ClNO	375.1385	CCCCCN1C2=C(C=CC=C2)C(C(C3=CC=C(Cl)C4=C3C=CC=C4)=O)=C1
JWH-398 (5-OH-Pentyl)	C24H22ClNO2	391.1334	ClC1=CC=C(C2=CC=CC=C12)C(=O)C1=CN(C2=CC=CC=C12)CCCCCO
JWH-398 (N-Pentanoic Acid)	C24H20ClNO3	405.1126	ClC1=CC=C(C2=CC=CC=C12)C(=O)C1=CN(C2=CC=CC=C12)CCCCC(=O)O
JWH-398 N-(4-Hydroxypentyl) Metabolite	C24H22ClNO2	391.1334	CC(O)CCCn1cc(C(=O)c2ccc(Cl)c3ccccc23)c2ccccc21
JWH-400	C22H18ClNO	347.1072	O=C(C1=C2C=CC=CC2=C(Cl)C=C1)C3=CN(CCC)C4=C3C=CC=C4
JWH-412	C24H22FNO	359.1680	O=C(C1=C2C=CC=CC2=C(F)C=C1)C3=CN(CCCCC)C4=C3C=CC=C4
JWH-413	C25H24FNO	373.1837	O=C(C1=C2C=CC=CC2=C(F)C=C1)C3=C(C)N(CCCCC)C4=C3C=CC=C4
JWH-424	C24H22BrNO	419.0879	O=C(C1=C2C(Br)=CC=CC2=CC=C1)C3=CN(CCCCC)C4=C3C=CC=C4
Kavain	C14H14O3	230.0938	O=C1C=C(OC)C[C@H](/C=C/C2=CC=CC=C2)O1
Ketamine	C13H16ClNO	237.0915	ClC1=C(C=CC=C1)C1(C(CCCC1)=O)NC
Ketazolam	C20H17ClN2O3	368.0922	ClC=1C=CC2=C(C3(N(CC(N2C)=O)C(C=C(O3)C)=O)C3=CC=CC=C3)C1
Lacosamide	C13H18N2O3	250.1312	[H][C@](COC)(N=C(C)O)C(O)=NCC1=CC=CC=C1
LAMPA	C20H25N3O	323.1992	CN(C(=O)[C@H]1CN([C@@H]2CC3=CNC4=CC=CC(C2=C1)=C34)C)CCC

Table S9. Compound database imported into the *Search Mass Lists* node (continued)

Compound Name	Molecular Formula	Exact Mass	SMILES
Levorphanol	C17H23NO	257.1774	[H][C@@]12CC3=C(C=C(O)C=C3)[C@@]3(CCCC[C@@]13[H])CCN2C
LH-21	C20H20Cl3N3	407.0717	CCCCCCC1=NN(C2=CC=C(Cl)C=C2Cl)C(C3=CC=C(Cl)C=C3)=N1
Lidocaine	C14H22N2O	234.1727	C(C)N(CC(=O)NC1=C(C=CC=C1C)C)CC
Lisdexamphetamine	C15H25N3O	263.1992	N[C@H](C(=O)N[C@H](CC1=CC=CC=C1)C)CCCCN
Lofendazam	C15H13ClN2O	272.0711	ClC=1C=CC2=C(N(C(CCN2)=O)C2=CC=CC=C2)C1
Lofentanil	C25H32N2O3	408.2407	O=C([C@]1(N(C2=CC=CC=C2)C(CC)=O)[C@@H](C)CN(CCC3=CC=CC=C3)CC1)OC
Lopirazepam	C14H9Cl2N3O2	321.0066	ClC=1C=CC=2NC(C(N=C(C2N1)C1=C(C=CC=C1)Cl)O)=O
Loprazolam	C23H21ClN6O3	464.1358	ClC1=C(C=CC=C1)C1=NCC=2N(C3=C1C=C(C=C3)[N+](=O)[O-])C(/C(/N2)=C/N2CCN(CC2)C)=O
Lorazepam	C15H10Cl2N2O2	320.0114	ClC=1C=CC2=C(C(=NC(C(N2)=O)O)C2=C(C=CC=C2)Cl)C1
Lormetazepam	C16H12Cl2N2O2	334.0270	ClC=1C=CC2=C(C(=NC(C(N2C)=O)O)C2=C(C=CC=C2)Cl)C1
LSA	C16H17N3O	267.1366	O=C([C@H](C=C12)CN(C)[C@]2([H])CC3=CNC4=C3C1=CC=C4)N
LSD	C20H25N3O	323.1992	C(C)N(C(=O)[C@@H]1C=C2C=3C=CC=C4NC=C(C[C@H]2N(C1)C)C34)CC
LSM-775	C20H23N3O2	337.1785	CN1C[C@@H](C=C2C=3C=CC=C4NC=C(C[C@@H]12)C34)C(=O)N3CCOCC3
LSZ	C21H25N3O	335.1992	O=C([C@H](C=C12)CN(C)[C@]2([H])CC3=CNC4=C3C1=CC=C4)N5[C@@H](C)C[C@@H]5C
LY2183240	C17H17N5O	307.1428	O=C(N1N=NN=C1CC2=CC=C(C3=CC=CC=C3)C=C2)N(C)C
Lysergic Acid Methylester	C17H18N2O2	282.1363	O=C(C(C=C12)CN(C)C2CC3=CNC4=C3C1=CC=C4)OC
M-144	C22H30FNO	343.2306	O=C(C1=C(C)N(CCCCCF)C2=C1C=CC=C2)C3C(C)(C)C3(C)C
MAB-CHMINACA	C21H30N4O2	370.2363	O=C(C1=NN(CC2CCCCC2)C3=C1C=CC=C3)NC(C(C)(C)C)C(N)=O
MAB-CHMINACA Metabolite M7	C21H27N3O5	401.1945	O=C(O)C(C)(C)C(NC(C1=NN(CC2CCCCC2)C3=C1C=CC=C3)=O)C(O)=O
MA-CHMINACA	C21H29N3O3	371.2203	CC(C)[C@H](NC(C1=NN(CC2CCCCC2)C3=C1C=CC=C3)=O)C(OC)=O
M-ALPHA	C11H15NO2	193.1097	CCC(C1=CC=C(OCO2)C2=C1)NC
M-ALPHA-HCMA	C13H18N2O4	266.1261	O1COC2=C1C=CC(=C2)C(C(C(=O)NC)(C)O)NC
MAM-2201	C25H24FNO	373.1837	O=C(C1=CN(CCCCCF)C2=C1C=CC=C2)C3=C4C=CC=CC4=C(C)C=C3
MAM2201 N-(4-Hydroxypentyl) Metabolite	C25H24FNO2	389.1786	O=C(C1=CN(CCCC(O)CF)C2=C1C=CC=C2)C3=C4C=CC=CC4=C(C)C=C3
MAM2201 N-Pentanoic Acid Metabolite	C25H23NO3	385.1673	O=C(O)CCCCN1C=C(C(C2=C3C=CC=CC3=C(C)C=C2)=O)C4=C1C=CC=C4
MAPA	C11H12O3	192.0781	O=C(C(C(=O)OC)C1=CC=CC=C1)C
Mazindol	C16H13ClN2O	284.0711	ClC1=CC=C(C=C1)C1(N2C(C3=CC=CC=C13)=NCC2)O
MBDB	C12H17NO2	207.1254	CCC(NC)CC1=CC=C(OCO2)C2=C1
MBZP (N-)	C12H18N2	190.1465	CN1CCN(CC2=CC=CC=C2)CC1
MCHB-1	C28H37N3O2	447.2880	O=C(C1=CC=C2C(N=C(CC3=CC=C(OCC)C=C3)N2CC4CCCCC4)=C1)N(CC)CC
M-CHMIC	C17H21NO2	271.1567	O=C(C1=CN(CC2CCCCC2)C3=C1C=CC=C3)OC
mCPP	C10H13ClN2	196.0762	ClC1=CC(N2CCNCC2)=CC=C1
McPT	C14H18N2	214.1465	CN(CCC1=CNC2=C1C=CC=C2)C3CC3
MDA 2-Aldoxime Analog	C11H13NO3	207.0890	O/N=C/C(C)CC1=CC=C(OCO2)C2=C1
MDA 77	C21H23N3O3	365.1734	CCCCCN1C(=O)C(=NN=C(O)C2=CC=CC=C2)C2=C1C=C(OC)C=C2
MDA-19 4-Hydroxybenzoyl Metabolite	C21H23N3O3	365.1734	C(CCCCC)N1C(\C(\C2=CC=CC=C12)=N/NC(C1=CC=C(C=C1)O)=O)=O
MDA-19 N-(5-Hydroxyhexyl) Metabolite	C21H23N3O3	365.1734	O=C(N/N=C1C(N(CCCCC(O)C)C2=C\1C=CC=C2)=O)C3=CC=CC=C3
MDAI	C10H11NO2	177.0784	C1OC=2C=C3CC(CC3=CC2O1)N
MDBZP	C12H16N2O2	220.1206	N1(CC2=CC=C(OCO3)C3=C2)CCNCC1
MDEA	C12H17NO2	207.1254	CC(CC1=CC(OCO2)=C2C=C1)NCC
MDHOET	C12H17NO3	223.1203	OCCNC(C)CC1=CC=C(OCO2)C2=C1
MDMAI	C11H13NO2	191.0941	CNC(C1)CC2=C1C=C3OCOC3=C2

**Table S9.** Compound database imported into the *Search Mass Lists* node (continued)			
Compound Name	Molecular Formula	Exact Mass	SMILES
MDMB-3en-BUTINACA	C19H25N3O3	343.1890	C=CCCn1nc(C(=O)NC(C(=O)OC)C(C)(C)C)c2ccccc21
MDMB-3en-BUTINACA Butanoic Acid Metabolite	C18H23N3O3	329.1734	C(CC=C)N1N=C(C2=CC=CC=C12)C(=O)N[C@H](C(=O)O)C(C)(C)C
MDMB-4en-PICA	C21H28N2O3	356.2094	O=C(N[C@@H](C(C)(C)C)C(OC)=O)C1=CN(CCCC=C)C2=C1C=CC=C2
MDMB-4en-PICA Butanoic Acid	C20H26N2O3	342.1938	CC([C@@H](C(=O)O)NC(=O)C1=CN(C2=CC=CC=C12)CCCC=C)(C)C
MDMB-4en-PINACA	C20H27N3O3	357.2047	CC(C(C(=O)OC)NC(=O)C1=NN(C2=CC=CC=C12)CCCC=C)(C)C
MDMB-4en-PINACA Butanoic Acid Metabolite	C19H25N3O3	343.1890	O=C(N[C@@H](C(C)(C)C)C(O)=O)C1=NN(CCCC=C)C2=C1C=CC=C2
MDMB-5Br-INACA	C15H18BrN3O3	367.0526	O=C(N[C@H](C(OC)=O)C(C)(C)C)C1=NNC2=C1C=C(Br)C=C2
MDMB-7Br-INACA	C15H18BrN3O3	367.0526	BrC=1C=CC=C2C(=NNC12)C(=O)NC(C(=O)OC)C(C)(C)C
MDMB-BUTINACA	C19H27N3O3	345.2047	O=C(N[C@H](C(OC)=O)C(C)(C)C)C1=NN(CCCC)C2=C1C=CC=C2
MDMB-BUTINACA Butanoic Acid Metabolite	C18H25N3O3	331.1890	C(CCC)N1N=C(C2=CC=CC=C12)C(=O)N[C@H](C(=O)O)C(C)(C)C
MDMB-CHM7AICA	C22H31N3O3	385.2360	C1(CCCCC1)CN1C=C(C=2C1=NC=CC2)C(=O)N[C@H](C(=O)OC)C(C)(C)C
MDMB-CHMCZCA	C27H34N2O3	434.2564	CC(C)(C)[C@H](NC(C1=CC2=C(C=C1)N(CC3CCCCC3)C4=C2C=CC=C4)=O)C(OC)=O
MDMB-CHMCZCA (Hydrolysat)	C26H32N2O3	420.2407	C1(CCCCC1)CN1C2=CC=CC=C2C=2C=C(C=CC12)C(=O)N[C@H](C(=O)O)C(C)(C)C
MDMB-CHMCZCA Metabolite M3	C20H21NO2	307.1567	O=C(O)c1ccc2c(c1)c1ccccc1n2CC1CCCCC1
MDMB-CHMICA	C23H32N2O3	384.2407	CC(C)(C)[C@@H](C(OC)=O)NC(C1=CN(CC2CCCCC2)C3=C1C=CC=C3)=O
MDMB-CHMICA Metabolite M2	C22H30N2O3	370.2251	CC(C)(C)[C@H](NC(C1=CN(CC2CCCCC2)C3=C1C=CC=C3)=O)C(O)=O
MDMB-CHMINACA	C22H31N3O3	385.2360	CC(C)(C)[C@@H](C(OC)=O)NC(C1=NN(CC2CCCCC2)C3=C1C=CC=C3)=O
MDMB-FUB7AICA	C22H24FN3O3	397.1796	FC1=CC=C(CN2C=C(C=3C2=NC=CC3)C(=O)N[C@H](C(=O)OC)C(C)(C)C)C=C1
MDMB-FUBICA	C23H25FN2O3	396.1844	CC(C)(C)C(NC(C1=CN(CC2=CC=C(F)C=C2)C3=C1C=CC=C3)=O)C(OC)=O
MDMB-FUBINACA	C22H24FN3O3	397.1796	CC(C)(C)[C@H](NC(C1=NN(CC2=CC=C(F)C=C2)C3=C1C=CC=C3)=O)C(OC)=O
MDMB-FUBINACA 3,3-Dimethylbutanoic Acid	C21H22FN3O3	383.1640	FC1=CC=C(CN2N=C(C3=CC=CC=C23)C(=O)N[C@H](C(=O)O)C(C)(C)C)C=C1
MDMB-INACA	C15H19N3O3	289.1421	CC(C)(C)C(NC(C1=NNC2=C1C=CC=C2)=O)C(OC)=O
MDMB-PCZCA	C25H32N2O3	408.2407	CC(C)(C)C(NC(C1=CC2=C(C=C1)N(CCCCC)C3=C2C=CC=C3)=O)C(OC)=O
MDMB-PICA	C21H30N2O3	358.2251	CC([C@@H](C(=O)OC)NC(=O)C1=CN(C2=CC=CC=C12)CCCCC)(C)C
MDMB-PICA (Hydrolysat)	C20H28N2O3	344.2094	CC([C@@H](C(=O)O)NC(=O)C1=CN(C2=CC=CC=C12)CCCCC)(C)C
MDMB-PINACA	C20H29N3O3	359.2203	CC([C@@H](C(=O)OC)NC(=O)C1=NN(C2=CC=CC=C12)CCCCC)(C)C
MDMB-PINACA (Hydrolysat)	C19H27N3O3	345.2047	CC([C@@H](C(=O)O)NC(=O)C1=NN(C2=CC=CC=C12)CCCCC)(C)C
MDP2P Glycidate	C12H12O5	236.0679	O1COC2=C1C=CC(=C2)C2C(O2)(C(=O)OC)C
MDPBP	C15H19NO3	261.1360	CCC(N1CCCC1)C(C2=CC=C(OCO3)C3=C2)=O
MDPHiP	C17H23NO3	289.1673	O1COC2=C1C=CC(=C2)C(C(CC(C)C)N2CCCC2)=O
MDPHP	C17H23NO3	289.1673	CCCCC(N1CCCC1)C(C2=CC=C(OCO3)C3=C2)=O
MDPPP	C14H17NO3	247.1203	CC(N1CCCC1)C(C2=CC(OCO3)=C3C=C2)=O
MDPV	C16H21NO3	275.1516	O1COC2=C1C=CC(=C2)C(C(CCC)N2CCCC2)=O
MDPV-M, Demethylenylmethyl	C16H23NO3	277.1673	CCCC(N1CCCC1)C(C2=CC=C(O)C(OC)=C2)=O
MEAI	C10H13NO	163.0992	COC=1C=C2CC(CC2=CC1)N
Mebroqualone	C15H11BrN2O	314.0049	O=C1N(C2=CC=CC=C2Br)C(C)=NC3=C1C=CC=C3
Medazepam	C16H15ClN2	270.0918	ClC1=CC(C(C2=CC=CC=C2)=NCCN3C)=C3C=C1
Medetomidine	C13H16N2	200.1308	CC1=C(C)C(C(C)C2=CN=CN2)=CC=C1
MEM	C13H21NO3	239.1516	C(C)OC1=CC(=C(C=C1OC)CC(C)N)OC
Memantine	C12H21N	179.1669	NC12CC3(C)CC(C2)(C)CC(C3)C1
Menitazene	C21H26N4O2	366.2050	C(C)N(CCN1C(=NC2=C1C=CC(=C2)[N+](=O)[O-])CC2=CC=C(C=C2)C)CC
Menitrazepam	C16H17N3O3	299.1264	C1(=CCCCC1)C1=NCC(N(C2=C1C=C(C=C2)[N+](=O)[O-])C)=O

**Table S9.** Compound database imported into the *Search Mass Lists* node (continued)			
Compound Name	Molecular Formula	Exact Mass	SMILES
MeO-MDA	C11H15NO3	209.1047	COC1=CC(=CC2=C1OCO2)CC(N)C
MeOP	C13H18N2O2	234.1363	O=C(C1=CC=C(OC)C=C1)N2CCN(C)CC2
MEP-CHMICA	C22H30N2O3	370.2251	CCC[C@H](NC(C1=CN(CC2CCCCC2)C3=C1C=CC=C3)=O)C(OC)=O
MEP-FUBICA	C22H23FN2O3	382.1687	CCC[C@H](NC(C1=CN(CC2=CC=C(F)C=C2)C3=C1C=CC=C3)=O)C(OC)=O
Mephedrene	C9H15NS	169.0920	CNC(CC=1SC(=CC1)C)C
Mephedrone	C11H15NO	177.1148	CNC(C(=O)C1=CC=C(C(C=C1)C)C
Mephedrone-M (Dihydro-)	C11H17NO	179.1305	CN[C@@H]([C@H](O)C1=CC=C(C(C=C1)C)C
MEPIRAPIM	C19H27N3O	313.2149	O=C(N1CCN(C)CC1)C2=CN(CCCCC)C3=C2C=CC=C3
MePPP	C14H19NO	217.1461	CC(N1CCCC1)C(C2=CC=C(C)C=C2)=O
Meprobamate	C9H18N2O4	218.1261	O=C(OCC(COC(=O)N)(C)CCC)N
Mescaline	C11H17NO3	211.1203	COC1=C(OC)C(OC)=CC(CCN)=C1
Mescaline-NBOMe	C19H25NO4	331.1778	COC1=C(OC)C(OC)=CC(CCNCC2=CC=CC=C2OC)=C1
Mesembrine	C17H23NO3	289.1673	O=C1CC[C@@]2(C3=CC=C(OC)C(OC)=C3)CCN(C)[C@@]2([H])C1
Mesoridazine	C21H26N2OS2	386.1481	CN1CCCCC1CCN1C2=CC=CC=C2SC2=C1C=C(C=C2)S(C)=O
MET	C13H18N2	202.1465	C(C)N(C)CCC1=CNC2=CC=CC=C12
meta-Chlorofentanyl	C22H27ClN2O	370.1807	ClC=1C=C(C=CC1)N(C(CC)=O)C1CCN(CC1)CCC1=CC=CC=C1
Metaclazepam	C18H18BrClN2O	392.0286	BrC=1C=CC2=C(C(=NCC(N2C)COC)C2=C(C=CC=C2)Cl)C1
meta-Hydroxycocaine	C17H21NO5	319.1414	OC=1C=C(C(=O)O[C@@H]2[C@@H]([C@H]3CC[C@@H](C2)N3C)C(=O)OC)C=CC1
Metamfepramone	C11H15NO	177.1148	CC(N(C)C)C(C1=CC=CC=C1)=O
Metaxalone	C12H15NO3	221.1047	CC1=CC(OCC2CN=C(O)O2)=CC(C)=C1
Methallylescaline	C14H21NO3	251.1516	NCCC1=CC(OC)=C(OCC(C)=C)C(OC)=C1
Methamnetamine	C14H17N	199.1356	CC(NC)CC1=CC=C2C=CC=CC2=C1
Methamphetamine Methyl Carbamate	C12H17NO2	207.1254	CN(C(OC)=O)C(CC1=CC=CC=C1)C
Methanandamide	C23H39NO2	361.2975	CCCCC/C=C\C/C=C\C/C=C\C/C=C\CCCC(N[C@H](C)CO)=O
Methaqualone	C16H14N2O	250.1101	O=C1N(C2=CC=CC=C2C)C(C)=NC3=C1C=CC=C3
Methcathinone	C10H13NO	163.0992	CC(NC)C(C1=CC=CC=C1)=O
Methedrone	C11H15NO2	193.1097	CC(NC)C(C1=CC=C(OC)C=C1)=O
Methiopropamine	C8H13NS	155.0763	S1C(=CC=C1)CC(C)NC
Methoxamine	C11H17NO3	211.1203	CC(N)C(C1=CC(OC)=CC=C1OC)O
Methoxetamine	C15H21NO2	247.1567	COC=1C=C(C=CC1)C1(C(CCCC1)=O)NCC
Methoxetamine Brominated Derivative	C15H20BrNO2	325.0672	O=C1C(NCC)(C2=CC(OC)=CC=C2Br)CCCC1
Methoxmetamine	C14H19NO2	233.1410	COC=1C=C(C=CC1)C1(C(CCCC1)=O)NC
Methoxphenidine	C20H25NO	295.1931	COC1=C(C=CC=C1)C(CC1=CC=CC=C1)N1CCCCC1
Methoxpropamine	C16H23NO2	261.1723	COC=1C=C(C=CC1)C1(C(CCCC1)=O)NCCC
Methoxy U-47700	C16H22Cl2N2O2	344.1053	ClC=1C=C(C(=O)N(OC)[C@H]2[C@@H](CCCC2)N(C)C)C=CC1Cl
Methoxyacetyl Norfentanyl	C14H20N2O2	248.1519	O=C(N(C1=CC=CC=C1)C2CCNCC2)COC
Methoxyphenamine	C11H17NO	179.1305	CC(NC)CC1=CC=CC=C1OC
Methyl (S)-2-(1H-Indole-3-carboxamido)-3,3-dimethylbutanoate	C16H20N2O3	288.1468	N1C=C(C2=CC=CC=C12)C(=O)N[C@H](C(=O)OC)C(C)(C)C
Methyl 1-(4-Fluorobenzyl)-1H-indazole-3-carboxylate	C16H13FN2O2	284.0956	O=C(OC)C1=NN(CC2=CC=C(F)C=C2)C3=C1C=CC=C3
Methyl 2-(1H-Indazole-3-carboxamido)-3-methylbutanoate	C14H17N3O3	275.1264	N=1NC(=C2C=CC=CC12)C(=O)N[C@@H](C(C)C)C(=O)OC
Methyl 2-Phenyl-2-(pyrrolidin-1-yl)acetate	C13H17NO2	219.1254	C1(=CC=CC=C1)C(C(=O)OC)N1CCCC1
Methyl-1-(5-fluoropentyl)-1H-indole-3-carboxylate	C15H18FNO2	263.1316	O=C(C1=CN(CCCCCF)C2=C1C=CC=C2)OC

**Table S9.** Compound database imported into the *Search Mass Lists* node (continued)			
Compound Name	Molecular Formula	Exact Mass	SMILES
Methyl-1-pentyl-1H-indole-3-carboxylate	C15H19NO2	245.1410	O=C(C1=CN(CCCCC)C2=C1C=CC=C2)OC
Methylbutyltryptamine	C15H22N2	230.1778	CCCCN(CCC1=CNC2=C1C=CC=C2)C
Methylclonazepam	C16H12ClN3O3	329.0562	O=C1N(C)C2=CC=C([N+]([O-])=O)C=C2C(C3=CC=CC=C3Cl)=NC1
Methylephedrine	C11H17NO	179.1305	CN([C@H]([C@H](O)C1=CC=CC=C1)C)C
Methylergonovine	C20H25N3O2	339.1941	O=C([C@H](C=C12)CN(C)[C@]2([H])CC3=CNC4=C3C1=CC=C4)N[C@@H](CC)CO
Methylhexanamine	C7H17N	115.1356	CC(N)CC(C)CC
Methylketobemidone	C14H19NO2	233.1410	OC=1C=C(C=CC1)C1(CCN(CC1)C)C(C)=O
Methylmethaqualone	C17H16N2O	264.1257	O=C1N(C2=CC=C(C)C=C2C)C(C)=NC3=C1C=CC=C3
Methylmorphenate	C13H17NO3	235.1203	O=C(OC)C(C1NCCOC1)C2=CC=CC=C2
Methylone	C11H13NO3	207.0890	O1COC2=C1C=CC(=C2)C(C(C)NC)=O
Methylphenidate	C14H19NO2	233.1410	O=C(OC)C(C1CCCCN1)C2=CC=CC=C2
Methysticin	C15H14O5	274.0836	O=C1C=C(OC)C[C@H](/C=C/C2=CC=C(OCO3)C3=C2)O1
Metodesnitazene	C21H27N3O	337.2149	C(C)N(CCN1C(=NC2=C1C=CC=C2)CC2=CC=C(C=C2)OC)CC
Mexazolam	C18H16Cl2N2O2	362.0583	ClC=1C=CC2=C(C3(N(CC(N2)=O)C(CO3)C)C3=C(C=CC=C3)Cl)C1
Mexedrone	C12H17NO2	207.1254	O=C(C1=CC=C(C)C=C1)C(NC)COC
Mexiletine	C11H17NO	179.1305	CC(N)COC1=C(C)C=CC=C1C
m-Fluorocyclopropylbenzylfentanyl	C22H25FN2O	352.1946	O=C(C1CC1)N(C2CCN(CC3=CC=CC=C3)CC2)C4=CC=CC(F)=C4
Midazolam	C18H13ClFN3	325.0777	ClC1=CC=C2C(C(C3=CC=CC=C3F)=NCC4=CN=C(C)N42)=C1
MIPLA	C20H25N3O	323.1992	O=C([C@H](C=C12)CN(C)[C@]2([H])CC3=CNC4=C3C1=CC=C4)N(C)C(C)C
Mirfentanil	C22H24N4O2	376.1894	O=C(N(C1CCN(CCC2=CC=CC=C2)CC1)C3=NC=CN=C3)C4=CC=CO4
Mitragynine	C23H30N2O4	398.2200	COC1=CC=CC2=C1C3=C([C@@](C[C@H](/C(C(OC)=O)=C\OC)[C@H](CC)C4)([H])N4CC3)N2
Mitraphylline	C21H24N2O4	368.1731	O=C(C1=CO[C@@H](C)[C@@]2([H])CN3CC[C@]4(C(NC5=C4C=CC=C5)=O)[C@]3([H])C[C@@]21[H])OC
MK-801	C16H15N	221.1199	C[C@@]12C3=C([C@@H](CC4=C1C=CC=C4)N2)C=CC=C3
MMAI	C11H15NO	177.1148	NC1CC2=C(C=C(OC)C(C)=C2)C1
MMB018	C20H28N2O3	344.2094	CC(C)[C@@H](C(OC)=O)NC(C1=CN(CCCCC)C2=C1C=CC=C2)=O
MMB-2201	C20H27FN2O3	362.2000	CC(C)[C@H](NC(C1=CN(CCCCCF)C2=C1C=CC=C2)=O)C(OC)=O
MMB-2201 (Valin)	C19H25FN2O3	348.1844	FCCCCCN1C=C(C2=CC=CC=C12)C(=O)N[C@@H](C(C)C)C(=O)O
MMB-4en-PICA	C20H26N2O3	342.1938	CC(C(C(=O)OC)NC(=O)C1=CN(C2=CC=CC=C12)CCCC=C)C
MMB-4en-PICA Butanoic Acid Metabolite	C19H24N2O3	328.1781	C(CCC=C)N1C=C(C2=CC=CC=C12)C(=O)N[C@@H](C(C)C)C(=O)O
MMB-5Br-INACA	C14H16BrN3O3	353.0370	O=C(N[C@H](C(OC)=O)C(C)C)C1=NNC2=C1C=C(Br)C=C2
MMB-CHM7AICA	C21H29N3O3	371.2203	C1(CCCCC1)CN1C=C(C=2C1=NC=CC2)C(=O)N[C@@H](C(C)C)C(=O)OC
MMB-CHMICA	C22H30N2O3	370.2251	C1(CCCCC1)CN1C=C(C2=CC=CC=C12)C(=O)N[C@H](C(=O)OC)C(C)C
MMB-FUBGACLONE	C24H23FN2O3	406.1687	FC1=CC=C(CN2C3=C(C=4C=CC=CC24)C(N(C=C3)C(C(=O)OC)C(C)C)=O)C=C1
MMB-FUBICA Metabolite 3	C21H21FN2O3	368.1531	FC1=CC=C(C=C1)CN1C=C(C2=CC=CC=C12)C(=O)N[C@H](C(=O)O)C(C)C
MMB-FUBINACA	C21H22FN3O3	383.1640	FC1=CC=C(C=C1)CN1N=C(C2=CC=CC=C12)C(=O)N[C@H](C(=O)OC)C(C)C
MMDA-2	C11H15NO3	209.1047	NC(C)CC1=C(OC)C=C(OCO2)C2=C1
mMDCK	C14H19NO	217.1461	CNC1(C(CCCC1)=O)C1=CC(=CC=C1)C
MMDPPA	C11H13NO3	207.0890	CC(C(=O)N)CC1=CC2=C(OCO2)C=C1
MMMP	C15H21NO2S	279.1288	CC(N1CCOCC1)(C)C(C2=CC=C(SC)C=C2)=O
MN-18	C23H23N3O	357.1836	O=C(C1=NN(CCCCC)C2=C1C=CC=C2)NC3=C4C=CC=CC4=CC=C3
MN-25	C26H37N3O3	439.2829	O=C(C1=CN(CCN2CCOCC2)C3=C1C=CC=C3OC)N[C@@H]4C(C)(C)[C@]5([H])CC[C@@]4(C)C5
MN-25 2-Methyl Derivative	C27H39N3O3	453.2986	O=C(C1=C(C)N(CCN2CCOCC2)C3=C1C=CC=C3OC)N[C@H]4[C@@](C5)(C)CC[C@@]5([H])C4(C)C

Table S9. Compound database imported into the *Search Mass Lists* node (continued)

Compound Name	Molecular Formula	Exact Mass	SMILES
MO-CHMINACA	C22H30N2O4	386.2200	O=C(C1=NN(CC2CCCCC2)C3=C1C=CC=C3)OC(C(C)(C)C)C(OC)=O
Modafiendz	C16H15F2NO2S	323.0786	O=C(NC)CS(C(C1=CC=C(F)C=C1)C2=CC=C(F)C=C2)=O
Modafinil	C15H15NO2S	273.0818	O=C(N)CS(C(C1=CC=CC=C1)C2=CC=CC=C2)=O
Modafinil Sulphone	C15H15NO3S	289.0767	O=C(N)CS(=O)(C(C1=CC=CC=C1)C2=CC=CC=C2)=O
Monoethylglycinexylidide	C12H18N2O	206.1414	CCNCC(=O)NC1=C(C=CC=C1C)C
MOPPP	C14H19NO2	233.1410	CC(N1CCCC1)C(C2=CC=C(OC)C=C2)=O
Motrazepam	C17H15N3O4	325.1057	COCN1C(CN=C(C2=C1C=CC(=C2)[N+](=O)[O-])C2=CC=CC=C2)=O
MPBP	C15H21NO	231.1618	CCC(N1CCCC1)C(C2=CC=C(C)C=C2)=O
MPBP-M, 4	C15H19NO3	261.1360	O=C(O)C1=CC=C(C(C(N2CCCC2)CC)=O)C=C1
MPHP	C17H25NO	259.1931	CCCCC(N1CCCC1)C(C2=CC=C(C)C=C2)=O
MPhP-2201	C24H27FN2O3	410.2000	O=C(OC)C(NC(C1=CN(CCCCCF)C2=C1C=CC=C2)=O)CC3=CC=CC=C3
MPHP-M, 4	C17H23NO3	289.1673	O=C(O)C1=CC=C(C(C(N2CCCC2)CCCC)=O)C=C1
MPP-PICA	C24H28N2O3	392.2094	O=C(N[C@H](C(OC)=O)CC1=CC=CC=C1)C2=CN(CCCCC)C3=C2C=CC=C3
MTTA	C12H15NO	189.1148	O=C1C(CNC)CCC2=C1C=CC=C2
MXiPr	C16H23NO2	261.1723	COc1cccc(c1)C1(CCCCC1=O)NC(C)C
N-(1-Benzyl-4-piperidyl)-N-benzylpropanamide	C22H28N2O	336.2196	C(C1=CC=CC=C1)N(C(CC)=O)C1CCN(CC1)CC1=CC=CC=C1
N-(2,6-Dimethylphenyl)-1-piperidineacetamide	C15H22N2O	246.1727	O=C(NC1=C(C)C=CC=C1C)CN2CCCCC2
N-(2-APB) Fentanyl	C25H30N2O2	390.2302	O1C(=CC2=C1C=CC=C2)CC(C)N2CCC(CC2)N(C(CC)=O)C2=CC=CC=C2
N-(6-APB) Fentanyl	C25H30N2O2	390.2302	O1C=CC2=C1C=C(C=C2)CC(C)N2CCC(CC2)N(C(CC)=O)C2=CC=CC=C2
N-(6-Dihydrobenzofuranylethyl) alpha-Methyl Fentanyl	C25H32N2O2	392.2458	CCC(N(C1=CC=CC=C1)C2CCN(C(C)CC3=CC(OCC4)=C4C=C3)CC2)=O
N,N-Bisdesethylisotonitazene	C19H22N4O3	354.1686	C(C)(C)OC1=CC=C(CC2=NC3=C(N2CCN)C=CC(=C3)[N+](=O)[O-])C=C1
N,N-Dibutyltryptamine	C18H28N2	272.2247	CCCCN(CCCC)CCC1=CNC2=C1C=CC=C2
N,N-Diethyl Hexedrone	C16H25NO	247.1931	CCCCC(N(CC)CC)C(C1=CC=CC=C1)=O
N,N-Diethyl Hexylone	C17H25NO3	291.1829	CCCCC(N(CC)CC)C(C1=CC=C(OCO2)C2=C1)=O
N,N-Diformylmescaline	C13H17NO5	267.1101	C(=O)N(C=O)CCC1=CC(=C(C(=C1)OC)OC)OC
N,N-Dimethyl Heptylone	C16H23NO3	277.1673	O1COC2=C1C=CC(=C2)C(C(CCCCC)N(C)C)=O
N,N-Dimethyl-3,4-DMA	C13H21NO2	223.1567	COC=1C=C(C=CC1OC)CC(N(C)C)C
N,N-Dimethyl-3,4-methylenedioxyamphetamine	C12H17NO2	207.1254	CC(N(C)C)CC1=CC=C(OCO2)C2=C1
N,N-Dimethylamphetamine	C11H17N	163.1356	CC(N(C)C)CC1=CC=CC=C1
N,N-Dimethylpentylone	C14H19NO3	249.1360	CCCC(N(C)C)C(C1=CC=C(OCO2)C2=C1)=O
N,N-Dimethylphenethylamine	C10H15N	149.1199	CN(C)CCC1=CC=CC=C1
N,N-DMT N-oxide	C12H16N2O	204.1257	C[N+](CCC1=CNC2=CC=CC=C12)(C)[O-]
N-Acetyl 25I-NBOMe	C20H24INO4	469.0745	CC(N(CCC1=CC(OC)=C(I)C=C1OC)CC2=CC=CC=C2OC)=O
N-Acetyl-3,4-methylenedioxymethcathinone	C13H15NO4	249.0996	O1COC2=C1C=CC(=C2)C(C(C)N(C(C)=O)C)=O
N-Acetyl-DOB	C13H18BrNO3	315.0465	CC(NC(C)CC1=CC(OC)=C(Br)C=C1OC)=O
N-Acetylmethamphetamine	C12H17NO	191.1305	CC(N(C)C(C)CC1=CC=CC=C1)=O
N-Adamantyl-fentanyl	C26H38N2O	394.2979	C12(CC3CC(CC(C1)C3)C2)N(C(CC)=O)C2CCN(CC2)CCC2=CC=CC=C2
Nalbuphine	C21H27NO4	357.1935	C1(CCC1)CN1[C@H]2[C@@]3(CC[C@@H]([C@H]4[C@@]3(C=3C(=C(C=CC3C2)O)O4)CC1)O)O
Nalmefene	C21H25NO3	339.1829	C1(CC1)CN1[C@H]2[C@@]3(CCC([C@H]4[C@@]3(C=3C(=C(C=CC3C2)O)O4)CC1)=C)O
Nalorphine	C19H21NO3	311.1516	C=CCN1CCC23C4C1CC5=C2C(=C(C=C5)O)OC3C(C=C4)O
Naloxegol	C33H51NO11	637.3456	OC1=CC=C2C[C@@H](N(CC=C)CC3)[C@]4(O)CCC(OCCOCCOCCOCCOCCOCCOCCO)[C@@]5([H])[C@]43C2=C1O5
Naltrexone	C20H23NO4	341.1622	[H][C@@]12OC3=C(O)C=CC4=C3[C@]11CCN(CC3CC3)[C@]([H])(C4)[C@@]1(O)CCC2=O

**Table S9.** Compound database imported into the *Search Mass Lists* node (continued)			
Compound Name	Molecular Formula	Exact Mass	SMILES
NAMIE	C21H17NO	299.1305	Cn1cc(C(=O)Cc2cccc3ccccc23)c2ccccc21
Naphyrone	C19H23NO	281.1774	CCCC(N1CCCC1)C(C2=CC=C3C=CC=CC3=C2)=O
NAPIE	C25H25NO	355.1931	CCCCCn1cc(C(=O)Cc2cccc3ccccc23)c2ccccc21
N-Benzoxazolyl-fentanyl	C23H27N3O2	377.2098	O1C=NC2=C1C=C(C=C2)N(C(CC)=O)C2CCN(CC2)CCC2=CC=CC=C2
N-Benzyl-1-phenethylamine	C15H17N	211.1356	CC(C1=CC=CC=C1)NCC2=CC=CC=C2
N-Benzyl-3,4-DMA	C18H23NO2	285.1723	CC(NCC1=CC=CC=C1)CC2=CC=C(OC)C(OC)=C2
N-Benzyl-3-F-norfentanyl	C21H25FN2O	340.1946	C(C1=CC=CC=C1)N1CCC(CC1)N(C(CC)=O)C1=CC(=CC=C1)F
N-Benzyl-3-methylfentanyl	C22H28N2O	336.2196	C(C1=CC=CC=C1)N1CC(C(CC1)N(C(CC)=O)C1=CC=CC=C1)C
N-Benzyl-butyrylfentanyl	C22H28N2O	336.2196	C(C1=CC=CC=C1)N1CCC(CC1)N(C(CCC)=O)C1=CC=CC=C1
N-Benzyl-p-fluoro-isobutyrylfentanyl	C23H28FNO	353.2150	C(C1=CC=CC=C1)N1CCC(CC1)C(C(C(C)C)=O)C1=CC=C(C=C1)F
N-Butyl Pentylone	C16H23NO3	277.1673	CCCC(NCCCC)C(C1=CC=C(OCO2)C2=C1)=O
N-Butylbutylone	C15H21NO3	263.1516	O1COC2=C1C=CC(=C2)C(C(CC)NCCCC)=O
N-Butylhexedrone	C16H25NO	247.1931	C(CCC)NC(C(=O)C1=CC=CC=C1)CCCC
N-Cyclohexyl Butylone	C17H23NO3	289.1673	O1COC2=C1C=CC(=C2)C(C(CC)NC2CCCCC2)=O
N-Cyclohexyl Methylone	C16H21NO3	275.1516	O1COC2=C1C=CC(=C2)C(C(C)NC2CCCCC2)=O
N-Cyclohexyl-N-methyl Methylone	C17H23NO3	289.1673	O1COC2=C1C=CC(=C2)C(C(C)N(C)C2CCCCC2)=O
N-Desethyl Etonitazene	C20H24N4O3	368.1843	CCNCCN1C2=CC=C([N+]([O-])=O)C=C2N=C1CC3=CC=C(OCC)C=C3
N-Desethyl Isotonitazene	C21H26N4O3	382.1999	C(C)NCCN1C(=NC2=C1C=CC(=C2)[N+](=O)[O-])CC2=CC=C(C=C2)OC(C)C
N-Desethyl Protonitazene	C21H26N4O3	382.1999	C(C)NCCN1C(=NC2=C1C=CC(=C2)[N+](=O)[O-])CC2=CC=C(C=C2)OCCC
N-Desethyl-O-desisopropyl-isotonitazene (M3)	C18H20N4O3	340.1530	C(C)NCCN1C(=NC2=C1C=CC(=C2)[N+](=O)[O-])CC2=CC=C(C=C2)O
NDTDI	C19H27N3O	313.2149	O=C(N(CC)CC)CCN(C)C(C1)CC2=CC=CC3=C2C1=CN3
NEB-Indene-analog	C15H21NO	231.1618	CCC(NCC)C(C1=CC2=C(CCC2)C=C1)=O
NE-CHMIMO	C26H25NO	367.1931	O=C(C1=CN(CC2CCCCC2)C3=C1C=CC=C3)C4=C5C=CC=CC5=CC=C4
Nefiracetam	C14H18N2O2	246.1363	CC1=C(C(=CC=C1)C)NC(CN1C(CCC1)=O)=O
NEiH	C14H21NO	219.1618	CC(C)CC(NCC)C(C1=CC=CC=C1)=O
N-Ethyl Hexylone	C15H21NO3	263.1516	CCCCC(NCC)C(C1=CC=C(OCO2)C2=C1)=O
N-Ethyl Zolpidem	C19H21N3O	307.1679	C(C)NC(CC1=C(N=C2N1C=C(C=C2)C)C2=CC=C(C=C2)C)=O
N-Ethyl-2C-B	C12H18BrNO2	287.0516	CCNCCC1=CC(OC)=C(Br)C=C1OC
N-Ethyl-3,4-DMA	C13H21NO2	223.1567	C(C)NC(CC1=CC(=C(C=C1)OC)OC)C
N-Ethylamphetamine	C11H17N	163.1356	CC(NCC)CC1=CC=CC=C1
N-Ethylbuphedrone	C12H17NO	191.1305	C(C)NC(C(=O)C1=CC=CC=C1)CC
N-Ethylheptedrone	C15H23NO	233.1774	C(C)NC(C(=O)C1=CC=CC=C1)CCCCC
N-Ethylheptylone	C16H23NO3	277.1673	O1COC2=C1C=CC(=C2)C(C(CCCCC)NCC)=O
N-Ethylnorketamine	C14H18ClNO	251.1072	O=C1C(NCC)(C2=CC=CC=C2Cl)CCCC1
N-Ethyl-N-propyl-1H-indole-3-ethanamine	C15H22N2	230.1778	CCCN(CC)CCC1=CNC2=C1C=CC=C2
N-Ethylpentylone	C14H19NO3	249.1360	O1COC2=C1C=CC(=C2)C(C(CCC)NCC)=O
N-Ethylphenmetrazol	C13H19NO2	221.1410	OC1(C2=CC=CC=C2)C(C)N(CC)CCO1
N-Ethyltryptamine (NET)	C12H16N2	188.1308	CCNCCC1=CNC2=C1C=CC=C2
N-Ethyl-U-47700	C17H24Cl2N2O	342.1260	ClC=1C=C(C(=O)N(CC)C2C(CCCC2)N(C)C)C=CC1Cl
NFEPP	C22H27FN2O	354.2102	CCC(N(C1C(F)CN(CCC2=CC=CC=C2)CC1)C3=CC=CC=C3)=O
N-Hydroxy MDA	C10H13NO3	195.0890	ONC(C)CC1=CC=C(OCO2)C2=C1
Nifoxipam	C15H10FN3O4	315.0650	O=C1C(O)N=C(C2=CC=CC=C2F)C3=CC([N+]([O-])=O)=CC=C3N1

**Table S9.** Compound database imported into the *Search Mass Lists* node (continued)			
Compound Name	Molecular Formula	Exact Mass	SMILES
Nimetazepam	C16H13N3O3	295.0951	O=C1N(C)C2=CC=C([N+]([O-])=O)C=C2C(C3=CC=CC=C3)=NC1
NiPH	C15H23NO	233.1774	C(C)(C)NC(C(=O)C1=CC=CC=C1)CCCC
NiPP	C14H21NO	219.1618	CCCC(NC(C)C)C(C1=CC=CC=C1)=O
N-Isopropyl Hexylone	C16H23NO3	277.1673	O1COC2=C1C=CC(=C2)C(C(CCCC)NC(C)C)=O
N-Isopropylbenzylamine	C10H15N	149.1199	CC(C)NCC1=CC=CC=C1
N-Isopropyl-N-propyltryptamine	C16H24N2	244.1934	N1C=C(C2=CC=CC=C12)CCN(CCC)C(C)C
Nitazene	C20H24N4O2	352.1894	C(C1=CC=CC=C1)C1=NC2=C(N1CCN(CC)CC)C=CC(=C2)[N+](=O)[O-]
Nitemazepam	C16H13N3O4	311.0901	OC1C(N(C2=C(C(=N1)C1=CC=CC=C1)C=C(C=C2)[N+](=O)[O-])C)=O
Nitizolam	C15H10ClN5O2S	359.0238	ClC1=C(C=CC=C1)C1=NCC=2N(C3=C1C=C(S3)[N+](=O)[O-])C(=NN2)C
Nitracaine	C16H24N2O4	308.1731	O=C(OCC(C)(C)CN(CC)CC)C1=CC=C([N+]([O-])=O)C=C1
Nitrazepam	C15H11N3O3	281.0795	[O-][N+](C1=CC2=C(C=C1)NC(CN=C2C3=CC=CC=C3)=O)=O
Nitrazolam	C17H13N5O2	319.1064	O=[N+](C1=CC=C(N2C(CN=C3C4=CC=CC=C4)=NN=C2C)C3=C1)[O-]
Nitromethaqualone	C16H13N3O4	311.0901	COC1=C(C=CC(=C1)[N+](=O)[O-])N1C(=NC2=CC=CC=C2C1=O)C
NM-2201	C24H22FNO2	375.1629	O=C(C1=CN(CCCCCF)C2=C1C=CC=C2)OC3=C4C=CC=CC4=CC=C3
NMDMSB	C20H19NO4S	369.1029	CN(S(=O)(=O)C=1C=C(C(=O)OC2=CC=CC3=CC=CC=C23)C=CC1C)C
N-Me-2C-H	C11H17NO2	195.1254	CNCCC1=CC(OC)=CC=C1OC
N-Me-bk-MMDA-2	C12H15NO4	237.0996	CC(NC)C(C1=C(OC)C=C(OCO2)C2=C1)=O
N-Methyl Aminorex Derivative	C10H12N2O	176.0944	N=C1OC(C2=CC=CC=C2)CN1C
N-Methyl Benzedrone	C18H21NO	267.1618	CC(N(CC1=CC=CC=C1)C)C(C2=CC=C(C)C=C2)=O
N-Methyl Mescaline	C12H19NO3	225.1360	COC=1C=C(C=C(C1OC)OC)CCNC
N-Methyl meta-Methyl Phenyl Fentanyl	C20H24N2O	308.1883	O=C(N(C1=CC(C)=CC=C1)C2CCN(C)CC2)C3=CC=CC=C3
N-Methyl ortho-Methyl Phenyl Fentanyl	C20H24N2O	308.1883	CN1CCC(CC1)N(C(C1=CC=CC=C1)=O)C1=C(C=CC=C1)C
N-Methyl p-Methylphenylnorfentanyl	C20H24N2O	308.1883	O=C(N(C1CCN(C)CC1)C2=CC=C(C)C=C2)C3=CC=CC=C3
N-Methyl Tryptamine	C11H14N2	174.1152	CNCCC1=CNC2=C1C=CC=C2
N-Methyl-1-phenylethylamine	C9H13N	135.1043	CC(NC)C1=CC=CC=C1
N-Methyl-1-phenylpropylamine	C10H15N	149.1199	CCC(NC)C1=CC=CC=C1
N-Methyl-2AI	C10H13N	147.1043	CNC1CC2=C(C=CC=C2)C1
N-Methyl-2C-B	C11H16BrNO2	273.0359	CNCCC1=CC(OC)=C(Br)C=C1OC
N-Methyl-acetyl-norfentanyl	C14H20N2O	232.1570	CN1CCC(CC1)N(C(C)=O)C1=CC=CC=C1
N-Methyl-butyrylfentanyl	C16H24N2O	260.1883	CN1CCC(CC1)N(C(CCC)=O)C1=CC=CC=C1
N-Methylcyclazodone	C13H14N2O2	230.1050	C1(CC1)N(C=1OC(C(N1)=O)C1=CC=CC=C1)C
N-Methyl-hexylone	C14H19NO3	249.1360	CCCCC(C(=O)c1ccc2c(c1)OCO2)NC
N-Methyl-N-allyl Tryptamine	C14H18N2	214.1465	N1C=C(C2=CC=CC=C12)CCN(CC=C)C
N-Methyl-N-ethyl Hexylone	C16H23NO3	277.1673	O1COC2=C1C=CC(=C2)C(C(CCCC)N(C)CC)=O
N-Methyl-N-isopropyl Tryptamine	C14H20N2	216.1621	CC(N(CCC1=CNC2=C1C=CC=C2)C)C
N-Methyl-N-propyl Methylone	C14H19NO3	249.1360	O1COC2=C1C=CC(=C2)C(C(C)N(CCC)C)=O
N-Methyl-N-propyl Pentylone	C16H23NO3	277.1673	O1COC2=C1C=CC(=C2)C(C(CCC)N(CCC)C)=O
N-Moc-MDMA	C13H17NO4	251.1152	O1COC2=C1C=CC(=C2)CC(C)N(C(OC)=O)C
NMP	C5H9NO	99.0679	O=C1N(C)CCC1
NMPEA	C9H13N	135.1043	CNCCC1=CC=CC=C1
NNEI	C24H24N2O	356.1883	O=C(C1=CN(CCCCC)C2=C1C=CC=C2)NC3=C4C=CC=CC4=CC=C3
NNEI 2-Indazole Isomer	C23H23N3O	357.1836	O=C(C1=C2C=CC=CC2=NN1CCCCC)NC3=C4C=CC=CC4=CC=C3

**Table S9.** Compound database imported into the *Search Mass Lists* node (continued)			
Compound Name	Molecular Formula	Exact Mass	SMILES
Noopept	C17H22N2O4	318.1574	C1(=CC=CC=C1)CC(=O)N1[C@H](CCC1)C(=O)NCC(=O)OCC
Nor-3-methylfentanyl	C15H22N2O	246.1727	CCC(N(C1C(C)CNCC1)C2=CC=CC=C2)=O
Norbaeocystin	C10H13N2O4P	256.0607	P(=O)(O)(O)OC=1C=2C(=CNC2C=CC1)CCN
Norclobazam	C15H11ClN2O2	286.0504	C1C(=O)NC2=C(C=C(C=C2)Cl)N(C1=O)C3=CC=CC=C3
Norephedrine	C9H13NO	151.0992	N[C@H]([C@H](O)C1=CC=CC=C1)C
Norketamine	C12H14ClNO	223.0759	O=C1C(C2=CC=CC=C2Cl)(N)CCCC1
Nor-mephedrone	C10H13NO	163.0992	CC(N)C(C1=CC=C(C)C=C1)=O
Normethoxetamine	C13H17NO2	219.1254	NC1(C(CCCC1)=O)C1=CC(=CC=C1)OC
Norpropoxyphene	C21H27NO2	325.2036	[H][C@@](C)(CNC)[C@@](CC1=CC=CC=C1)(OC(=O)CC)C1=CC=CC=C1
Nortetrazepam	C15H15ClN2O	274.0868	ClC=1C=CC2=C(C(=NCC(N2)=O)C2=CCCCC2)C1
Nortilidine	C16H21NO2	259.1567	CCOC(=O)C1(CCC=CC1NC)C2=CC=CC=C2
NPB-22	C22H21N3O2	359.1628	O=C(C1=NN(CCCCC)C2=C1C=CC=C2)OC3=C4N=CC=CC4=CC=C3
NPDPA	C17H21N	239.1669	CC(NC(C1=CC=CC=C1)CC2=CC=CC=C2)C
N-Pentyl Methylone	C15H21NO3	263.1516	O1COC2=C1C=CC(=C2)C(C(C)NCCCCC)=O
N-Phenethyl-N-phenylpropionamide	C17H19NO	253.1461	C1(=CC=CC=C1)N(C(CC)=O)CCC1=CC=CC=C1
N-Piperidinyl 4’-Hydroxy Nitazene	C21H24N4O3	380.1843	[N+](=O)([O-])C1=CC2=C(N(C(=N2)CC2=CC=C(C=C2)O)CCN2CCCCC2)C=C1
N-Piperidinyl Isotonitazene	C24H30N4O3	422.2312	C(C)(C)OC1=CC=C(CC2=NC3=C(N2CCN2CCCCC2)C=CC(=C3)[N+](=O)[O-])C=C1
N-Piperidinyl Metonitazene	C22H26N4O3	394.1999	COC1=CC=C(CC2=NC3=C(N2CCN2CCCCC2)C=CC(=C3)[N+](=O)[O-])C=C1
N-Piperidinyl Protonitazene	C24H30N4O3	422.2312	[N+](=O)([O-])C1=CC2=C(N(C(=N2)CC2=CC=C(C=C2)OCCC)CCN2CCCCC2)C=C1
NPNK	C15H20ClNO	265.1228	ClC1=C(C=CC=C1)C1(C(CCCC1)=O)NCCC
N-Propionyl Norfentanyl	C17H24N2O2	288.1832	O=C(CC)N1CCC(CC1)N(C(CC)=O)C1=CC=CC=C1
N-Propyl Butylone	C14H19NO3	249.1360	O1COC2=C1C=CC(=C2)C(C(CC)NCCC)=O
N-Propyl Hexylone	C16H23NO3	277.1673	O1COC2=C1C=CC(=C2)C(C(CCCC)NCCC)=O
N-Propyl Pentedrone	C14H21NO	219.1618	CCCC(NCCC)C(C1=CC=CC=C1)=O
N-Propylamphetamine	C12H19N	177.1512	CCCC1=CC=CC=C1CC(C)N
N-Pyrrolidino 4’-Hydroxy Nitazene	C20H22N4O3	366.1686	[N+](=O)([O-])C1=CC2=C(N(C(=N2)CC2=CC=C(C=C2)O)CCN2CCCC2)C=C1
N-Pyrrolidino Isotonitazene	C23H28N4O3	408.2156	C(C)(C)OC1=CC=C(CC2=NC3=C(N2CCN2CCCC2)C=CC(=C3)[N+](=O)[O-])C=C1
N-Pyrrolidino Metonitazene	C21H24N4O3	380.1843	COC1=CC=C(CC2=NC3=C(N2CCN2CCCC2)C=CC(=C3)[N+](=O)[O-])C=C1
N-Pyrrolidino Protonitazene	C23H28N4O3	408.2156	[N+](=O)([O-])C1=CC2=C(N(C(=N2)CC2=CC=C(C=C2)OCCC)CCN2CCCC2)C=C1
N-Pyrrolidinyl-3,4-DMA	C15H23NO2	249.1723	COC=1C=C(C=CC1OC)CC(C)N1CCCC1
N-Quinolinyl-fentanyl	C25H29N3O	387.2305	C1(=CC=CC=C1)CCN1CCC(CC1)N(C(CC)=O)C=1C=CC=C2C=CC=NC12
NRG-3	C16H19NO	241.1461	CNC(C(=O)C1=CC2=CC=CC=C2C=C1)CCC
NSI-189	C22H30N4O	366.2414	C(C1=CC=CC=C1)N1CCN(CC1)C(=O)C=1C(=NC=CC1)NCCC(C)C
O-AMKD	C16H21NO3	275.1516	C(C)(=O)OC1=CC(=CC=C1)C1(CCN(CC1)C)C(C)=O
Octodrine	C8H19N	129.1512	CC(N)CCCC(C)C
Octyl-CP	C19H37N	279.2921	C(CCCCCCC)C1(CCCCC1)N1CCCCC1
O-Desmethyl Venlafaxine	C16H25NO2	263.1880	CN(C)CC(C1=CC=C(O)C=C1)C1(O)CCCCC1
Ohmefentanyl	C23H30N2O2	366.2302	CCC(N(C1C(C)CN(CC(O)C2=CC=CC=C2)CC1)C3=CC=CC=C3)=O
o-MDCK	C14H19NO	217.1461	CNC1(C(CCCC1)=O)C1=C(C=CC=C1)C
Org 27569	C24H28ClN3O	409.1915	O=C(C(N1)=C(CC)C2=C1C=CC(Cl)=C2)NCCC3=CC=C(N4CCCCC4)C=C3
Org 27759	C21H24FN3O	353.1898	O=C(C(N1)=C(CC)C2=C1C=CC(F)=C2)NCCC3=CC=C(N(C)C)C=C3
Org 28611	C23H33N3O2	383.2567	O=C(C1=CN(CC2CCCCC2)C3=C1C=CC=C3OC)N4CC(C)N(C)CC4

Table S9. Compound database imported into the *Search Mass Lists* node (continued)			
Compound Name	Molecular Formula	Exact Mass	SMILES
Org 29647	C22H24ClN3O	381.1602	O=C(C(N1)=C(CC)C2=C1C=CC(Cl)=C2)N[C@H]3CN(CC4=CC=CC=C4)CC3
Orphenadrine	C18H23NO	269.1774	CC1=CC=CC=C1C(C2=CC=CC=C2)OCCN(C)C
Orphenadrine-Nor (Tofenacin. Elamol)	C17H21NO	255.1618	CC1=CC=CC=C1C(C2=CC=CC=C2)OCCNC
Ostarine	C19H14F3N3O3	389.0982	O=C(NC1=CC=C(C#N)C(C(F)(F)F)=C1)C(C)(O)COC2=CC=C(C#N)C=C2
Oxazepam	C15H11ClN2O2	286.0504	ClC=1C=CC2=C(C(=NC(C(N2)=O)O)C2=CC=CC=C2)C1
Oxazolam	C18H17ClN2O2	328.0973	ClC=1C=CC2=C(C3(N(CC(N2)=O)CC(O3)C)C3=CC=CC=C3)C1
Oxiracetam	C6H10N2O3	158.0686	OC1CC(N(C1)CC(=O)N)=O
P2NP	C9H9NO2	163.0628	C/C([N+]([O-])=O)=C\C1=CC=CC=C1
P4TCP	C16H23NS	261.1546	C1(=CC=CC=C1)C1(CCSCC1)N1CCCCC1
Pagoclone	C23H22ClN3O2	407.1395	ClC1=CC=C2C=CC(=NC2=N1)N1C(C2=CC=CC=C2C1CC(CCC(C)C)=O)=O
para-Bromo 4-ANPP	C19H23BrN2	358.1039	BrC1=CC=C(NC2CCN(CCC3=CC=CC=C3)CC2)C=C1
para-Fluoro 4-Anilinopiperidine	C11H15FN2	194.1214	FC1=CC=C(C=C1)NC1CCNCC1
para-Fluoro Furanyl Norfentanyl	C16H17FN2O2	288.1269	FC1=CC=C(C=C1)N(C(=O)C1=COC=C1)C1CCNCC1
para-Fluoro Phenethyl 4-ANPP	C27H31FN2	402.2466	FC1=CC=C(C=C1)N(C1CCN(CC1)CCC1=CC=CC=C1)CCC1=CC=CC=C1
para-Fluorophenylpiperazine	C10H13FN2	180.1057	FC1=CC=C(N2CCNCC2)C=C1
para-Hydroxycocaine	C17H21NO5	319.1414	OC1=CC=C(C(=O)O[C@@H]2[C@@H]([C@H]3CC[C@@H](C2)N3C)C(=O)OC)C=C1
para-Methoxy 4-ANPP	C20H26N2O	310.2040	COC1=CC=C(NC2CCN(CCC3=CC=CC=C3)CC2)C=C1
para-Methyl AP-237	C18H26N2O	286.2040	C1(=CC=C(C=C1)/C=C/CN1CCN(CC1)C(CCC)=O)C
Paynantheine (Mitragyna Alkaloid)	C23H28N2O4	396.2044	O=C(OC)/C([C@@H]1[C@@H](C=C)CN2CCC3=C(NC4=C3C(OC)=CC=C4)[C@]2([H])C1)=C/OC
PB-22	C23H22N2O2	358.1676	O=C(C1=CN(CCCCC)C2=C1C=CC=C2)OC3=C4N=CC=CC4=CC=C3
PB-22 (N-4-OH-Pentyl-3-carboxyindole)	C14H17NO3	247.1203	OC(CCCN1C=C(C2=CC=CC=C12)C(=O)O)C
PB-22 (N-5-OH-Pentyl)	C23H22N2O3	374.1625	OCCCCCN1C=C(C2=CC=CC=C12)C(=O)OC=1C=CC=C2C=CC=NC12
PB-22 (N-5-OH-Pentyl-3-carboxyindole)	C14H17NO3	247.1203	OCCCCCN1C=C(C2=CC=CC=C12)C(=O)O
PB-22 (N-Pentanoic acid-3-carboxyindole)	C14H15NO4	261.0996	C(=O)(O)CCCCN1C=C(C2=CC=CC=C12)C(=O)O
PB-22 3-Carboxyindole Metabolite	C14H17NO2	231.1254	O=C(C1=CN(CCCCC)C2=C1C=CC=C2)O
PB-22 N-(4-Hydroxypentyl) Metabolite	C23H22N2O3	374.1625	CC(O)CCCn1cc(C(=O)Oc2cccc3cccnc23)c2ccccc21
PB-22 N-Pentanoic Acid Metabolite	C23H20N2O4	388.1418	O=C(O)CCCCN1C=C(C(OC2=C3N=CC=CC3=CC=C2)=O)C4=C1C=CC=C4
PBCHA	C13H17N	187.1356	C1(=CC=CC=C1)C1(C2CCC(C1)C2)N
PBCHE	C15H21N	215.1669	C(C)NC1(C2CCC(C1)C2)C2=CC=CC=C2
PBCHP	C18H25N	255.1982	C1(=CC=CC=C1)C1(C2CCC(C1)C2)N2CCCCC2
PBCHPr	C16H23N	229.1825	C1(=CC=CC=C1)C1(C2CCC(C1)C2)NCCC
PBCHPy	C17H23N	241.1825	C1(=CC=CC=C1)C1(C2CCC(C1)C2)N2CCCC2
pBPP	C10H13BrN2	240.0257	BrC1=CC=C(N2CCNCC2)C=C1
PC3MP	C18H27N	257.2138	CC1CN(CCC1)C1(CCCCC1)C1=CC=CC=C1
PC44DMP	C19H29N	271.2295	CC1(CCN(CC1)C1(CCCCC1)C1=CC=CC=C1)C
PC4HOP	C17H25NO	259.1931	C1(=CC=CC=C1)C1(CCCCC1)N1CCC(CC1)O
PC4MP	C18H27N	257.2138	CC1CCN(CC1)C1(CCCCC1)C1=CC=CC=C1
PCAl	C15H21N	215.1669	C1(=CC=CC=C1)C1(CCCCC1)NCC=C
PCBP	C15H21N	215.1669	C1(=CC=CC=C1)C1(CCC1)N1CCCCC1
PCBu	C16H25N	231.1982	C(CCC)NC1(CCCCC1)C1=CC=CC=C1
PCBzl	C19H23N	265.1825	C(C1=CC=CC=C1)NC1(CCCCC1)C1=CC=CC=C1
PCDMe	C14H21N	203.1669	CN(C1(CCCCC1)C1=CC=CC=C1)C

**Table S9.** Compound database imported into the *Search Mass Lists* node (continued)			
Compound Name	Molecular Formula	Exact Mass	SMILES
PCDMPy	C18H27N	257.2138	CC1(CN(CC1)C1(CCCCC1)C1=CC=CC=C1)C
PCEEA	C16H25NO	247.1931	CCOCCNC1(C2=CC=CC=C2)CCCCC1
PCEM	C15H23N	217.1825	C(C)N(C1(CCCCC1)C1=CC=CC=C1)C
PCHEPy	C17H25N	243.1982	C1(=CC=CC=C1)C1(CCCCCC1)N1CCCC1
PCHOEA	C14H21NO	219.1618	C1(=CC=CC=C1)C1(CCCCC1)NCCO
PCiP	C15H23N	217.1825	C1(=CC=CC=C1)C1(CCCCC1)NC(C)C
PCMe	C13H19N	189.1512	CNC1(CCCCC1)C1=CC=CC=C1
PCMEA	C15H23NO	233.1774	COCCNC1(CCCCC1)C1=CC=CC=C1
PCMo	C16H23NO	245.1774	C1(=CC=CC=C1)C1(CCCCC1)N1CCOCC1
PCMPA	C16H25NO	247.1931	COCCCNC1(CCCCC1)C1=CC=CC=C1
PCP	C17H25N	243.1982	N1(C2(C3=CC=CC=C3)CCCCC2)CCCCC1
PCP-4”-one	C17H23NO	257.1774	C1(=CC=CC=C1)C1(CCCCC1)N1CCC(CC1)=O
PCPe	C17H27N	245.2138	C(CCCC)NC1(CCCCC1)C1=CC=CC=C1
PCPEP	C16H23N	229.1825	C1(=CC=CC=C1)C1(CCCC1)N1CCCCC1
PCPEPr	C14H21N	203.1669	C1(=CC=CC=C1)C1(CCCC1)NCCC
pCPP	C10H13ClN2	196.0762	ClC1=CC=C(N2CCNCC2)C=C1
PCPr	C15H23N	217.1825	C1(=CC=CC=C1)C1(CCCCC1)NCCC
PCPyrrole	C16H19N	225.1512	C1(=CC=CC=C1)C1(CCCCC1)N1C=CC=C1
PDM-35	C12H17NO	191.1305	CC1COC(C2=CC=CC=C2)C(C)N1
PDPC	C22H29NO	323.2244	CN(C1(CCC(CC1)(O)CCC1=CC=CC=C1)C1=CC=CC=C1)C
PEAP	C13H21N	191.1669	C(C)NC(CC1=CC=CC=C1)CCC
Pentazocine	C19H27NO	285.2087	CC12CCN(C(CC3=C1C=C(C=C3)O)C2C)CC=C(C)C
Pentedrone	C12H17NO	191.1305	O=C(C(CCC)NC)C1=CC=CC=C1
Pentedrone Metabolite (Ephedrine Stereochemistry)	C12H19NO	193.1461	CN[C@H](CCC)C(O)C1=CC=CC=C1
PF-03550096	C19H28N4O4	376.2105	O=C(N1C2=CC=CC=C2N(CCC(C)(O)C)C1=O)N[C@@H](C(C)(C)C)C(N)=O
pF-4-Methylaminorex	C10H11FN2O	194.0850	FC1=CC=C(C=C1)C1C(N=C(O1)N)C
pF-BZP	C11H15FN2	194.1214	FC1=CC=C(C=C1)CN1CCNCC1
p-Fluoro-beta-hydroxy-thiobutyrylfentanyl	C21H27FN2O2S	390.1772	FC1=CC=C(C=C1)N(C(CCC)=O)C1CCN(CC1)CC(C=1SC=CC1)O
p-Fluorococaine	C17H20FNO4	321.1371	O=C([C@@H]1[C@@](N2C)([H])CC[C@@]2([H])C[C@@H]1OC(C3=CC=C(F)C=C3)=O)OC
p-Fluoro-furanylethylfentanyl	C21H26FNO2	343.1942	FC1=CC=C(C=C1)C(C(CC)=O)C1CCN(CC1)CCC=1OC=CC1
p-Fluoro-furanylremifentanil	C22H25FN2O6	432.1691	FC1=CC=C(C=C1)N(C1(CCN(CC1)CCC(=O)OC)C(=O)OC)C(=O)C=1OC=CC1
p-Fluoroisobutyrylbenzylfentanyl	C22H27FN2O	354.2102	CCCC(N(C1CCN(CC2=CC=CC=C2)CC1)C3=CC=C(F)C=C3)=O
p-Fluoro-remifentanil Ethyl Ester	C21H29FN2O5	408.2055	C(C)OC(CCN1CCC(CC1)(C(=O)OC)N(C(CC)=O)C1=CC=C(C=C1)F)=O
p-Fluoro-thiofentanyl	C20H25FN2OS	360.1666	FC1=CC=C(C=C1)N(C(CC)=O)C1CCN(CC1)CCC=1SC=CC1
Pharaohfentanyl	C23H30N2O2	366.2302	OC(CN1CCC(CC1)(C)N(C(CC)=O)C1=CC=CC=C1)C1=CC=CC=C1
Phenacetin	C10H13NO2	179.0941	C(C)OC1=CC=C(C=C1)NC(C)=O
Phenaridine	C24H32N2O	364.2509	CCC(N(C1CC(C)N(CCC2=CC=CC=C2)CC1C)C3=CC=CC=C3)=O
Phenazepam 4-Oxide	C15H10BrClN2O2	363.9609	O=C1N=C2C(C=C(Br)C=C2)=C(C3=CC=CC=C3Cl)N(O)C1
Phenethyl 4-ANPP	C27H32N2	384.2560	C(CC1=CC=CC=C1)N(C1CCN(CC1)CCC1=CC=CC=C1)C1=CC=CC=C1
Phenethylamine (2-)	C8H11N	121.0886	NCCC1=CC=CC=C1
Phenetrazine	C12H17NO	191.1305	CCC1NCCOC1C2=CC=CC=C2
Phenibut	C10H13NO2	179.0941	NCC(CC(=O)O)C1=CC=CC=C1

**Table S9.** Compound database imported into the *Search Mass Lists* node (continued)			
Compound Name	Molecular Formula	Exact Mass	SMILES
Phenmetetrazine	C13H19NO	205.1461	CC1N(CC)CCOC1C2=CC=CC=C2
Phenmetrazine	C11H15NO	177.1148	CC1NCCOC1C2=CC=CC=C2
Phenoxyethyl-norfentanyl	C22H28N2O2	352.2145	O(C1=CC=CC=C1)CCN1CCC(CC1)N(C(CC)=O)C1=CC=CC=C1
Phentermine	C10H15N	149.1199	CC(N)(C)CC1=CC=CC=C1
Phenylephrine	C9H13NO2	167.0941	O[C@@H](CNC)C=1C=C(C=CC1)O
Phenylpiracetam	C12H14N2O2	218.1050	O=C1N(CC(C1)C1=CC=CC=C1)CC(=O)N
Phenylpropylmethylamine	C10H15N	149.1199	CC(CNC)C1=CC=CC=C1
Phenylpropyl-norfentanyl	C23H30N2O	350.2353	C1(=CC=CC=C1)N(C(CC)=O)C1CCN(CC1)CCCC1=CC=CC=C1
Pinazepam	C18H13ClN2O	308.0711	ClC=1C=CC2=C(C(=NCC(N2CC#C)=O)C2=CC=CC=C2)C1
p-Iodofentanyl	C22H27IN2O	462.1163	IC1=CC=C(C=C1)N(C(CC)=O)C1CCN(CC1)CCC1=CC=CC=C1
Piperazine-1((4-chlorophenyl) phenyl methyl)	C17H19ClN2	286.1231	ClC1=CC=C(C(N2CCNCC2)C3=CC=CC=C3)C=C1
Piperazine-o-chlorophenyl	C10H13ClN2	196.0762	ClC1=CC=CC=C1N2CCNCC2
Pivoxazepam	C20H19ClN2O3	370.1079	CC(C)(C)C(OC1C(NC2=CC=C(Cl)C=C2C(C3=CC=CC=C3)=N1)=O)=O
PMA	C10H15NO	165.1148	CC(N)CC1=CC=C(OC)C=C1
PMEA	C12H19NO	193.1461	CC(NCC)CC1=CC=C(OC)C=C1
PMMA	C11H17NO	179.1305	CC(NC)CC1=CC=C(OC)C=C1
Poppers	C5H11NO2	117.0784	O=NOCCCCC
PPAP	C14H23N	205.1825	C1(=CC=CC=C1)CC(CCC)NCCC
PPEPP	C24H32N2	348.2560	C1(=CC=CC=C1)C1(CCN(CC1)CCC1=CC=CC=C1)N1CCCCC1
PPH	C16H23NO2	261.1723	O=C(OCCC)C(C1=CC=CC=C1)C2NCCCC2
PPyP	C16H23NO	245.1774	C1(=CC=CC=C1)C1(CCOCC1)N1CCCCC1
Prazepam	C19H17ClN2O	324.1024	O=C1N(CC2CC2)C3=CC=C(Cl)C=C3C(C4=CC=CC=C4)=NC1
PRE-084	C19H27NO3	317.1986	O=C(C1(C2=CC=CC=C2)CCCCC1)OCCN3CCOCC3
Pregabalin	C8H17NO2	159.1254	CC(C)CC(CC(=O)O)CN
Pregabalin Methyl Ester	C9H19NO2	173.1410	CC(C)CC(CN)CC(OC)=O
Premazepam	C15H15N3O	253.1210	CC=1N(C=C2NC(CN=C(C21)C2=CC=CC=C2)=O)C
Primidone	C12H14N2O2	218.1050	CCC1(C(O)=NCN=C1O)C1=CC=CC=C1
PRL-8-53	C18H21NO2	283.1567	O=C(OC)C1=CC=CC(CCN(C)CC2=CC=CC=C2)=C1
Procaine	C13H20N2O2	236.1519	NC1=CC=C(C(=O)OCCN(CC)CC)C=C1
Proflazepam	C18H16ClFN2O3	362.0828	ClC=1C=CC2=C(C(=NCC(N2CC(CO)O)=O)C2=C(C=CC=C2)F)C1
PRO-LAD	C22H29N3O	351.2305	C(C)N(C(=O)[C@H]1CN([C@@H]2CC=3C4=C(C2=C1)C=CC=C4NC3)CCC)CC
Prolintane	C15H23N	217.1825	CCCC(N1CCCC1)CC2=CC=CC=C2
Promethazine	C17H20N2S	284.1342	CN(C(CN1C2=CC=CC=C2SC=2C=CC=CC12)C)C
Propoxyphene	C22H29NO2	339.2193	[H][C@@](C)(CN(C)C)[C@@](CC1=CC=CC=C1)(OC(=O)CC)C1=CC=CC=C1
Propoxyphenyl Homohydroxysildenafil	C24H34N6O5S	518.2306	OCCN1CCN(CC1)S(=O)(=O)C=1C=CC(=C(C1)C=1NC(C2=C(N1)C(=NN2C)CCC)=O)OCCC
Propyl U-47700	C18H26Cl2N2O	356.1417	ClC=1C=C(C(=O)N(CCC)C2C(CCCC2)N(C)C)C=CC1Cl
Propylcathinone	C12H17NO	191.1305	CC(NCCC)C(C1=CC=CC=C1)=O
Propyl-CP	C14H27N	209.2138	C(CC)C1(CCCCC1)N1CCCCC1
Propyl-norfentanyl	C17H26N2O	274.2040	C1(=CC=CC=C1)N(C(CC)=O)C1CCN(CC1)CCC
Propylone	C13H17NO3	235.1203	CC(NCCC)C(C1=CC=C(OCO2)C2=C1)=O
Proscaline	C13H21NO3	239.1516	NCCC1=CC(OC)=C(OCCC)C(OC)=C1
Protodesnitazene	C23H31N3O	365.2462	C(C)N(CCN1C(=NC2=C1C=CC=C2)CC2=CC=C(C=C2)OCCC)CC

**Table S9.** Compound database imported into the *Search Mass Lists* node (continued)			
Compound Name	Molecular Formula	Exact Mass	SMILES
Psicofentanil	C32H40N4O3	528.3095	N1C=C(C2=CC=CC=C12)CCN1CCC(CC1)(C(=O)OC1CN2CCC1CC2)N(C(CC)=O)C2=CC=CC=C2
Psilocin	C12H16N2O	204.1257	OC=1C=CC=C2NC=C(CCN(C)C)C12
Psilocybin	C12H17N2O4P	284.0920	CN(C)CCC1=CNC2=C1C(=CC=C2)OP(=O)(O)O
p-TFM-fentanyl	C23H27F3N2O	404.2070	C1(=CC=CC=C1)CCN1CCC(CC1)N(C(CC)=O)C1=CC=C(C=C1)C(F)(F)F
PTI-1	C21H29N3S	355.2077	CCN(CC)CC1=CSC(C2=CN(CCCCC)C3=C2C=CC=C3)=N1
PTI-2	C23H33N3OS	399.2339	CCCCCn1cc(-c2nc(CN(CCOC)C(C)C)cs2)c2ccccc21
PTI-3	C21H28FN3OS	389.1932	CN(CC1=CSC(C2=CN(CCCCCF)C3=CC=CC=C32)=N1)CCOC
PV8	C17H25NO	259.1931	C1(=CC=CC=C1)C(C(CCCCC)N1CCCC1)=O
PV9	C18H27NO	273.2087	CCCCCCC(N1CCCC1)C(C2=CC=CC=C2)=O
PX-1	C23H26FN3O2	395.2004	O=C(N)[C@H](CC1=CC=CC=C1)NC(C2=CN(CCCCCF)C3=C2C=CC=C3)=O
PX-2	C22H25FN4O2	396.1956	O=C(C1=NN(CCCCCF)C2=C1C=CC=C2)N[C@H](CC3=CC=CC=C3)C(N)=O
Pyclazolam	C16H12ClN5	309.0776	ClC=1C=CC2=C(C(=NCC=3N2C(=NN3)C)C3=NC=CC=C3)C1
Pyeazolam	C18H13N5	299.1166	C(#C)C=1C=CC2=C(C(=NCC=3N2C(=NN3)C)C3=NC=CC=C3)C1
Pynazolam	C16H12N6O2	320.1016	CC1=NN=C2N1C1=C(C(=NC2)C2=NC=CC=C2)C=C(C=C1)[N+](=O)[O-]
Pyrilamine	C17H23N3O	285.1836	COC1=CC=C(CN(CCN(C)C)C2=CC=CC=N2)C=C1
Pyrovalerone	C16H23NO	245.1774	CCCC(N1CCCC1)C(C2=CC=C(C)C=C2)=O
Pyr-T	C14H18N2	214.1465	C1(CCN2CCCC2)=CNC3=C1C=CC=C3
Quazepam	C17H11ClF4N2S	386.0262	ClC=1C=CC2=C(C(=NCC(N2CC(F)(F)F)=S)C2=C(C=CC=C2)F)C1
Quetiapine	C21H25N3O2S	383.1662	OCCOCCN1CCN(CC1)C1=NC2=C(SC3=C1C=CC=C3)C=CC=C2
R-30490	C24H32N2O2	380.2458	CCC(N(C1(COC)CCN(CCC2=CC=CC=C2)CC1)C3=CC=CC=C3)=O
Razobazam	C14H14N4O2	270.1111	CC=1NN=C2N(C(CC(N(C21)C2=CC=CC=C2)=O)=O)C
RCS-4	C21H23NO2	321.1723	COC1=CC=C(C=C1)C(=O)C1=CN(C2=CC=CC=C12)CCCCC
RCS-4 (5-OH-Pentyl)	C21H23NO3	337.1673	O=C(C1=CN(CCCCCO)C2=C1C=CC=C2)C3=CC=C(OC)C=C3
RCS-4 2-Methoxy Isomer	C21H23NO2	321.1723	O=C(C1=CC=CC=C1OC)C2=CN(CCCCC)C3=C2C=CC=C3
RCS-4 3-Methoxy Isomer	C21H23NO2	321.1723	COC=1C=C(C=CC1)C(=O)C1=CN(C2=CC=CC=C12)CCCCC
RCS-4 N-(4-Oxo-pentyl)phenol Metabolite (M11)	C20H19NO3	321.1360	CC(CCCN1C=C(C(C2=CC=C(O)C=C2)=O)C3=C1C=CC=C3)=O
RCS-4 N-(5-Hydroxypentyl)phenol	C20H21NO3	323.1516	OC(CCCN1C=C(C2=CC=CC=C12)C(=O)C1=CC=C(C=C1)O)C
RCS-4 N-Pentanoic Acid	C21H21NO4	351.1465	O=C(O)CCCCN1C=C(C(C2=CC=C(OC)C=C2)=O)C3=C1C=CC=C3
RCS-4 ortho Pentanoic Acid Metabolite	C21H21NO4	351.1465	O=C(O)CCCCN1C=C(C(C2=CC=CC=C2OC)=O)C3=C1C=CC=C3
RCS-4-C4 Homolog	C20H21NO2	307.1567	O=C(C1=CC=C(OC)C=C1)C2=CN(CCCC)C3=C2C=CC=C3
RCS-4N-(4-Hydroxypentyl) Metabolite	C21H23NO3	337.1673	COc1ccc(C(=O)c2cn(CCCC(C)O)c3ccccc23)cc1
RCS-8	C25H29NO2	375.2193	COC1=CC=CC=C1CC(C2=CN(CCC3CCCCC3)C4=C2C=CC=C4)=O
Reclazepam	C18H13Cl2N3O2	373.0379	ClC=1C=CC2=C(C(=NCCN2C=2OCC(N2)=O)C2=C(C=CC=C2)Cl)C1
Remifentanil bis Ethyl Ester	C22H32N2O5	404.2306	C(C)OC(CCN1CCC(CC1)(C(=O)OCC)N(C(CC)=O)C1=CC=CC=C1)=O
Remimazolam	C21H19BrN4O2	438.0686	BrC=1C=CC2=C(C(=N[C@H](C=3N2C(=CN3)C)CCC(=O)OC)C3=NC=CC=C3)C1
RH-34	C18H19N3O3	325.1421	O=C(N1CCNCC2=CC=CC=C2OC)NC3=C(C=CC=C3)C1=O
Ricinine	C8H8N2O2	164.0580	N#CC1=C(OC)C=CN(C)C1=O
Rilmazafone	C21H20Cl2N6O3	474.0968	NCC(=O)NCC1=NC(=NN1C1=C(C=C(C=C1)Cl)C(C1=C(C=CC=C1)Cl)=O)C(=O)N(C)C
Rilmazolam	C19H15Cl2N5O	399.0648	ClC=1C=CC2=C(C(=NCC=3N2N=C(N3)C(=O)N(C)C)C3=C(C=CC=C3)Cl)C1
Rimonabant	C22H21Cl3N4O	462.0775	O=C(C1=NN(C2=CC=C(Cl)C=C2Cl)C(C3=CC=C(Cl)C=C3)=C1C)NN4CCCCC4
Ripazepam	C15H16N4O	268.1319	C(C)N1N=C(C=2NC(CN=C(C21)C2=CC=CC=C2)=O)C
Ritalinic Acid	C13H17NO2	219.1254	C1(=CC=CC=C1)C(C(=O)O)C1NCCCC1

Table S9. Compound database imported into the *Search Mass Lists* node (continued)

Compound Name	Molecular Formula	Exact Mass	SMILES
Ro 07-4065	C16H11ClF2N2O	320.0523	O=C1N(C)C2=CC=C(Cl)C=C2C(C3=C(F)C=CC=C3F)=NC1
Ro-07-3953	C15H9ClF2N2O	306.0366	ClC=1C=CC2=C(C(=NCC(N2)=O)C2=C(C=CC=C2F)F)C1
Ro5-4864	C16H12Cl2N2O	318.0321	O=C1N(C)C2=CC=C(Cl)C=C2C(C3=CC=C(Cl)C=C3)=NC1
Rolicyclidine	C16H23N	229.1825	C1(=CC=CC=C1)C1(CCCCC1)N1CCCC1
RTI-111	C16H19Cl2NO2	327.0787	O=C(C1C(N2C)CCC2CC1C3=CC=C(Cl)C(Cl)=C3)OC
RTI-240	C17H24ClN	277.1592	ClC1=CC=C(C=C1)[C@@H]1[C@@H](C2CCC(C1)N2C)C(C)C
RTI-31	C16H20ClNO2	293.1177	ClC1=CC=C(C=C1)[C@@H]1[C@@H]([C@H]2CC[C@@H](C1)N2C)C(=O)OC
Ru-28306	C13H16N2	200.1308	CN(C)C(C1)CC2=CC=CC3=C2C1=CN3
Salvinorin A	C23H28O8	432.1779	O=C([C@@H]1C[C@H](OC(C)=O)C([C@]([C@@]1(C)CC[C@]23[H])([H])[C@@]2(C)C[C@@H](C4=COC=C4)OC3=O)=O)OC
Salvinorin B	C21H26O7	390.1673	O=C([C@@H]1C[C@H](O)C([C@]([C@@]1(C)CC[C@]23[H])([H])[C@@]2(C)C[C@@H](C4=COC=C4)OC3=O)=O)OC
Scopolamine	C17H21NO4	303.1465	[H][C@](CO)(C(=O)O[C@]1([H])C[C@@]2([H])N(C)[C@@]([H])(C1)[C@]1([H])O[C@]21[H])C1=CC=CC=C1
SDB-005	C23H22N2O2	358.1676	O=C(C1=NN(CCCCC)C2=C1C=CC=C2)OC3=C4C=CC=CC4=CC=C3
SDB-006	C21H24N2O	320.1883	O=C(C1=CN(CCCCC)C2=C1C=CC=C2)NCC3=CC=CC=C3
SDB-006-N-Phenyl	C20H22N2O	306.1727	O=C(C1=CN(CCCCC)C2=C1C=CC=C2)NC3=CC=CC=C3
Sec-Butonitazene	C24H32N4O3	424.2469	C(C)(CC)OC1=CC=C(CC2=NC3=C(N2CCN(CC)CC)C=CC(=C3)[N+](=O)[O-])C=C1
SER-601	C28H38N2O2	434.2928	O=C(C1=CN(CCCCC)C2=C(C=C(C(C)C)C=C2)C1=O)NC34CC5CC(C4)CC(C5)C3
Sertraline	C17H17Cl2N	305.0733	ClC1=CC=C([C@H]2C3=C([C@H](CC2)NC)C=CC=C3)C=C1Cl
SGT-13	C22H20F2N2O4S	446.1106	O=C(OC1=C2N=CC=CC2=CC=C1)C3=CC=C(C)C(S(=O)(N4CCC(F)(F)CC4)=O)=C3
SGT-235	C24H28F2N4O	426.2226	O=C(C1=NN(CCN2CCC(F)(F)CC2)C3=C1C=CC=C3)NC(C)(C4=CC=CC=C4)C
Sibutramine	C17H26ClN	279.1748	CC(C)CC(C1(C2=CC=C(Cl)C=C2)CCC1)N(C)C
sibutramine-Nor	C16H24ClN	265.1592	CC(C)CC(C1(C2=CC=C(Cl)C=C2)CCC1)NC
SL-164	C16H12Cl2N2O	318.0321	ClC1=C2C(N(C(=NC2=CC=C1)C)C1=C(C=C(C=C1)Cl)C)=O
SL-651,498	C23H20FN3O2	389.1534	FC=1C=C2C3=C(N(C2=CC1)C)C(N(C=C3C(=O)N3CCCC3)C3=CC=CC=C3)=O
Strychnine	C21H22N2O2	334.1676	[H][C@]12C[C@@]3([H])C4=CCO[C@@]5([H])CC(=O)N6C7=CC=CC=C7[C@]1(CCN2C4)[C@]6([H])[C@@]35[H]
STS-135	C24H31FN2O	382.2415	O=C(C1=CN(CCCCCF)C2=C1C=CC=C2)NC34CC5CC(C4)CC(C5)C3
STS-135 (4-OH-Pentyl)	C24H31FN2O2	398.2364	C12(CC3CC(CC(C1)C3)C2)NC(=O)C2=CN(C3=CC=CC=C23)CCCC(CF)O
Sulazepam	C16H13ClN2S	300.0483	ClC=1C=CC2=C(C(=NCC(N2C)=S)C2=CC=CC=C2)C1
Sultiame	C10H14N2O4S2	290.0389	O=S(C1=CC=C(N(CCCC2)S2(=O)=O)C=C1)(N)=O
Sunifiram	C14H18N2O2	246.1363	CCC(N1CCN(C(C2=CC=CC=C2)=O)CC1)=O
T3CP	C15H23NS	249.1546	S1C=C(C=C1)C1(CCCCC1)N1CCCCC1
Tadalafil	C22H19N3O4	389.1370	O=C1N(CC(N2[C@@H]1CC3=C([C@H]2C4=CC5=C(OCO5)C=C4)NC6=C3C=CC=C6)=O)C
Tapentadol	C14H23NO	221.1774	CN(C[C@@H]([C@@H](CC)C=1C=C(C=CC1)O)C)C
TBCHA	C11H15NS	193.0920	S1C(=CC=C1)C1(C2CCC(C1)C2)N
TBCHP	C16H23NS	261.1546	S1C(=CC=C1)C1(C2CCC(C1)C2)N2CCCCC2
TBCHPy	C15H21NS	247.1389	S1C(=CC=C1)C1(C2CCC(C1)C2)N2CCCC2
tBuONE	C14H19NO3	249.1360	O1COC2=C1C=CC(=C2)C(C(C)NC(C)(C)C)=O
TCE	C12H19NS	209.1233	C(C)NC1(CCCCC1)C=1SC=CC1
TCM	C11H17NS	195.1076	CNC1(CCCCC1)C=1SC=CC1
TCPr	C13H21NS	223.1389	C(CC)NC1(CCCCC1)C=1SC=CC1
TCPy	C14H21NS	235.1389	S1C(=CC=C1)C1(CCCCC1)N1CCCC1
TDIQ	C10H11NO2	177.0784	C12=CC3=C(OCO3)C=C1CNCC2
Temazepam	C16H13ClN2O2	300.0660	CN1C2=C(C(C3=CC=CC=C3)=NC(O)C1=O)C=C(Cl)C=C2

**Table S9.** Compound database imported into the *Search Mass Lists* node (continued)			
Compound Name	Molecular Formula	Exact Mass	SMILES
Tenocyclidine	C15H23NS	249.1546	S1C(=CC=C1)C1(CCCCC1)N1CCCCC1
Tetrahydrofuran Fentanyl	C24H30N2O2	378.2302	O=C(C1OCCC1)N(C2=CC=CC=C2)C3CCN(CCC4=CC=CC=C4)CC3
Tetrahydrothiophene Fentanyl	C24H30N2OS	394.2073	O=C(C1CCCS1)N(C2CCN(CCC3=CC=CC=C3)CC2)C4=CC=CC=C4
Tetrazepam	C16H17ClN2O	288.1024	ClC=1C=CC2=C(C(=NCC(N2C)=O)C2=CCCCC2)C1
TFMPP	C11H13F3N2	230.1025	FC(C=1C=C(C(=CC1)N1CCNCC1)(F)F
Thebaine	C19H21NO3	311.1516	COC1=CC=C2[C@@H](C3)N(C)CC[C@@]24C5=C3C=CC(OC)=C5O[C@@H]14
Thiafentanil	C22H28N2O4S	416.1764	COCC(=O)N(C1(CCN(CC1)CCC=1SC=CC1)C(=O)OC)C1=CC=CC=C1
Thienoamphetamine	C7H11NS	141.0607	CC(N)CC1=CC=CS1
Thionordazepam	C15H11ClN2S	286.0326	S=C1NC2=CC=C(Cl)C=C2C(C3=CC=CC=C3)=NC1
Thiothinon	C8H11NOS	169.0556	CC(NC)C(C1=CC=CS1)=O
THJ	C22H22N4O	358.1788	O=C(C1=NN(CCCCC)C2=C1C=CC=C2)NC3=C4N=CC=CC4=CC=C3
THJ-018	C23H22N2O	342.1727	O=C(C1=C2C=CC=CC2=CC=C1)C3=NN(CCCCC)C4=C3C=CC=C4
THJ-2201	C23H21FN2O	360.1633	O=C(C1=NN(CCCCCF)C2=C1C=CC=C2)C3=C4C=CC=CC4=CC=C3
THJ-2201 (N-Pentanoic Acid)	C23H20N2O3	372.1468	O=C(O)CCCCN1N=C(C(C2=C3C=CC=CC3=CC=C2)=O)C4=C1C=CC=C4
THJ2201 N-(5-Hydroxypentyl) Metabolite	C23H22N2O2	358.1676	O=C(C1=NN(CCCCCO)C2=C1C=CC=C2)C3=C4C=CC=CC4=CC=C3
TH-PBP	C18H25NO	271.1931	CCC(N1CCCC1)C(C2=CC=C3CCCCC3=C2)=O
TH-PHP	C20H29NO	299.2244	CCCCC(N1CCCC1)C(C2=CC=C3CCCCC3=C2)=O
TH-PVP	C19H27NO	285.2087	CCCC(N1CCCC1)C(C2=CC=C3CCCCC3=C2)=O
THQ-PINACA	C22H25N3O	347.1992	N1(CCCC2=CC=CC=C12)C(=O)C1=NN(C2=CC=CC=C12)CCCCC
Tianeptine-M MC5	C19H21ClN2O4S	408.0905	CN1C2=CC=CC=C2C(C3=C(S1([O-])[O-])C=C(C=C3)Cl)NCCCCC(=O)O
Tiletamine	C12H17NOS	223.1026	O=C1C(C2=CC=CS2)(NCC)CCCC1
Tilidine	C17H23NO2	273.1723	O=C([C@@]1(C2=CC=CC=C2)[C@H](N(C)C)C=CCC1)OCC
TMA-2	C12H19NO3	225.1360	CC(N)CC1=CC(OC)=C(OC)C=C1OC
TMA-6	C12H19NO3	225.1360	CC(N)CC1=C(OC)C=C(OC)C=C1OC
Tofisopam	C22H26N2O4	382.1887	CCC1C(=NN=C(C2=CC(=C(C=C12)OC)OC)C3=CC(=C(C=C3)OC)OC)C
Tolufazepam	C24H20Cl2N2O3S	486.0566	ClC=1C=CC2=C(C(=NCC(N2CCS(=O)(=O)C2=CC=C(C=C2)C)=O)C2=C(C=CC=C2)Cl)C1
trans-3-Methyl 4’,para-Difluoro Fentanyl	C23H28F2N2O	386.2164	FC1=CC=C(CCN2C[C@H]([C@@H](CC2)N(C(CC)=O)C2=CC=C(C=C2)F)C)C=C1
trans-3-Methyl Thiofentanyl	C21H28N2OS	356.1917	CCC(N([C@@H]1[C@H](C)CN(CCC2=CC=CS2)CC1)C3=CC=CC=C3)=O
trans-Cinnamoylcocaine	C19H23NO4	329.1622	CN1[C@H]2[C@H]([C@H](C[C@@H]1CC2)OC(\C=C\C2=CC=CC=C2)=O)C(=O)OC
Tranylcypromine	C9H11N	133.0886	[H][C@@]1(N)C[C@@]1([H])C1=CC=CC=C1
Triazolam	C17H12Cl2N4	342.0434	ClC1=CC=CC=C1C2=NCC3=NN=C(C)N3C4=CC=C(Cl)C=C42
Triflubazam	C17H13F3N2O2	334.0924	CN1C(CC(N(C2=C1C=CC(=C2)C(F)(F)F)C2=CC=CC=C2)=O)=O
Triflunordazepam	C16H11F3N2O	304.0818	C1(=CC=CC=C1)C1=NCC(NC2=C1C=C(C=C2)C(F)(F)F)=O
Trifluoromethyldeschloroketamine	C14H16F3NO	271.1179	CNC1(C(CCCC1)=O)C1=C(C=CC=C1)C(F)(F)F
Troparil	C16H21NO2	259.1567	O=C(C1C(C2=CC=CC=C2)CC3CCC1N3C)OC
Tropicamide	C17H20N2O2	284.1519	O=C(N(CC)CC1=CC=NC=C1)C(C2=CC=CC=C2)CO
Tuclazepam	C17H16Cl2N2O	334.0634	ClC=1C=CC2=C(C(=NCC(N2C)CO)C2=C(C=CC=C2)Cl)C1
U-47109	C15H20Cl2N2O	314.0947	ClC=1C=C(C(=O)N[C@H]2[C@@H](CCCC2)N(C)C)C=CC1Cl
U-47931E	C15H21BrN2O	324.0832	O=C(NC1C(N(C)C)CCCC1)C2=CC=C(Br)C=C2
U-50488	C19H26Cl2N2O	368.1417	ClC=1C=C(C=CC1Cl)CC(=O)N(C1C(CCCC1)N1CCCC1)C
U-51754	C17H24Cl2N2O	342.1260	ClC=1C=C(C=CC1Cl)CC(=O)N(C)[C@H]1[C@@H](CCCC1)N(C)C
U-62066	C22H30Cl2N2O2	424.1679	ClC=1C=C(C=CC1Cl)CC(=O)N([C@H]1[C@@H](C[C@@]2(CCCO2)CC1)N1CCCC1)C

**Table S9.** Compound database imported into the *Search Mass Lists* node (continued)			
Compound Name	Molecular Formula	Exact Mass	SMILES
U-69593	C22H32N2O2	356.2458	CN(C1CCC2(CCCO2)CC1N3CCCC3)C(=O)CC4=CC=CC=C4
UF-17	C17H26N2O	274.2040	CN(C1C(CCCC1)N(C(CC)=O)C1=CC=CC=C1)C
Uldazepam	C18H15Cl2N3O	359.0587	ClC=1C=CC2=C(C(=NCC(=N2)NOCC=C)C2=C(C=CC=C2)Cl)C1
UR-144	C21H29NO	311.2244	O=C(C1=CN(CCCCC)C2=C1C=CC=C2)C3C(C)(C)C3(C)C
UR-144 (5-OH-Pentyl)	C21H29NO2	327.2193	OCCCCCN1C=C(C2=CC=CC=C12)C(=O)C1C(C1(C)C)(C)C
UR-144 Degradant	C21H29NO	311.2244	C=C(C)C(C)(C)CC(C1=CN(CCCCC)C2=C1C=CC=C2)=O
UR-144 N-(2-Chloropentyl) Analog	C21H28ClNO	345.1854	O=C(C1=CN(CC(Cl)CCC)C2=C1C=CC=C2)C3C(C)(C)C3(C)C
UR-144 N-(3-Chloropentyl) Analog	C21H28ClNO	345.1854	O=C(C1=CN(CCC(Cl)CC)C2=C1C=CC=C2)C3C(C)(C)C3(C)C
UR-144 N-(4-Chloropentyl) Analog	C21H28ClNO	345.1854	O=C(C1=CN(CCCC(Cl)C)C2=C1C=CC=C2)C3C(C)(C)C3(C)C
UR-144 N-(4-Hydroxypentyl) Metabolite	C21H29NO2	327.2193	O=C(C1=CN(CCCC(O)C)C2=C1C=CC=C2)C3C(C)(C)C3(C)C
UR-144 N-(5-Chloropentyl) Analog	C21H28ClNO	345.1854	O=C(C1=CN(CCCCCCl)C2=C1C=CC=C2)C3C(C)(C)C3(C)C
UR-144 N-(5-Methylhexyl) Analog	C23H33NO	339.2557	O=C(C1=CN(CCCCC(C)C)C2=C1C=CC=C2)C3C(C)(C)C3(C)C
UR-144 N-Heptyl Analogue	C23H33NO	339.2557	O=C(C1=CN(CCCCCCC)C2=C1C=CC=C2)C3C(C)(C)C3(C)C
UR-144 N-Pentanoic Acid	C21H27NO3	341.1986	O=C(O)CCCCN1C=C(C(C2C(C)(C)C2(C)C)=O)C3=C1C=CC=C3
UR-144-N5-brompentyl	C21H28BrNO	389.1349	O=C(C1=CN(CCCCCBr)C2=C1C=CC=C2)C3C(C)(C)C3(C)C
URB-447	C25H21ClN2O	400.1337	O=C(C1=C(C)N(CC2=CC=C(Cl)C=C2)C(C3=CC=CC=C3)=C1N)C4=CC=CC=C4
URB-597	C20H22N2O3	338.1625	O=C(OC1=CC(C2=CC=CC(C(N)=O)=C2)=CC=C1)NC3CCCCC3
URB-754	C16H14N2O2	266.1050	O=C1C2=CC(C)=CC=C2N=C(NC3=CC=C(C)C=C3)O1
UWA-101	C13H17NO2	219.1254	O1COC2=C1C=CC(=C2)CC(NC)C2CC2
Valone	C14H14O3	230.0938	O=C1C(C(CC(C)C)=O)C(C2=C1C=CC=C2)=O
Vanoxerine	C28H32F2N2O	450.2477	FC1=CC=C(C(C2=CC=C(F)C=C2)OCCN3CCN(CCCC4=CC=CC=C4)CC3)C=C1
Venlafaxine	C17H27NO2	277.2036	OC1(C(C2=CC=C(OC)C=C2)CN(C)C)CCCCC1
Viloxazine	C13H19NO3	237.1360	CCOC1=CC=CC=C1OCC2CNCCO2
Vinpocetine	C22H26N2O2	350.1989	C(C)C12CCCN3CCC=4C5=CC=CC=C5N(C(=C1)C(=O)OCC)C4C23
Vortioxetine	C18H22N2S	298.1498	CC1=CC(=C(C=C1)SC2=CC=CC=C2N3CCNCC3)C
VX-809	C24H18F2N2O5	452.1178	O=C(O)C1=CC=CC(C2=NC(NC(C3(C4=CC=C(OC(F)(F)O5)C5=C4)CC3)=O)=CC=C2C)=C1
W-15	C19H21ClN2O2S	376.1007	O=S(C1=CC=C(Cl)C=C1)(/N=C2N(CCC3=CC=CC=C3)CCCC\2)=O
W-18	C19H20ClN3O4S	421.0857	C1CCN(/C(=N\S(=O)(=O)C2=CC=C(C=C2)Cl)/C1)CCC3=CC=C(C=C3)[N+](=O)[O-]
W-19	C19H22ClN3O2S	391.1116	NC1=CC=C(C=C1)CCN1\C(\CCCC1)=N/S(=O)(=O)C1=CC=C(C=C1)Cl
WIN 35,428	C16H20FNO2	277.1473	O=C(C1C(C2=CC=C(F)C=C2)CC3CCC1N3C)OC
WIN 48,098	C23H26N2O3	378.1938	O=C(C1=CC=C(OC)C=C1)C2=C(C)N(CCN3CCOCC3)C4=C2C=CC=C4
WIN 55,212-2	C27H26N2O3	426.1938	O=C(C1=C(C)N2C3=C1C=CC=C3OC[C@H]2CN4CCOCC4)C5=C6C=CC=CC6=CC=C5
WIN-54,461	C23H25BrN2O3	456.1043	O=C(C1=C(C)N(CCN2CCOCC2)C3=C1C=CC(Br)=C3)C4=CC=C(OC)C=C4
XLR-11	C21H28FNO	329.2150	O=C(C1=CN(CCCCCF)C2=C1C=CC=C2)C3C(C)(C)C3(C)C
XLR-11 (6-OH-Indole)	C21H28FNO2	345.2099	FCCCCCN1C=C(C2=CC=C(C=C12)O)C(=O)C1C(C1(C)C)(C)C
XLR11 Degradant	C21H28FNO	329.2150	C=C(C)C(C)(C)CC(C1=CN(CCCCCF)C2=C1C=CC=C2)=O
XLR11 N-(4-Hydroxypentyl) Metabolite	C21H28FNO2	345.2099	FCC(CCCN1C=C(C2=CC=CC=C12)C(=O)C1C(C1(C)C)(C)C)O
XLR-11-N4-pentenyl	C21H27NO	309.2087	O=C(C1=CN(CCCC=C)C2=C1C=CC=C2)C3C(C)(C)C3(C)C
XLR-12	C20H24F3NO	351.1805	O=C(C1C(C)(C)C1(C)C)C2=CN(CCCC(F)(F)F)C3=C2C=CC=C3
Yangonin	C15H14O4	258.0887	O=C1C=C(OC)C=C(/C=C/C2=CC=C(OC)C=C2)O1
Yohimbine	C21H26N2O3	354.1938	COC(=O)C1C(CCC2C1CC3C4=C(CCN3C2)C5=CC=CC=C5N4)O
Zaleplon	C17H15N5O	305.1271	CC(N(C1=CC=CC(C2=CC=NC3=C(C#N)C=NN23)=C1)CC)=O

Table S9. Compound database imported into the *Search Mass Lists* node (continued)

Compound Name	Molecular Formula	Exact Mass	SMILES
Zapizolam	C15H9Cl2N5	329.0230	ClC=1C=CC2=C(C(=NCC=3N2C=NN3)C3=C(C=CC=C3)Cl)N1
ZDCM-04	C20H28ClN5O4	437.1824	ClC1=CC(=C(C=C1OC)CC(C)NCCN1C=NC2N(C(N(C(C12)=O)C)=O)C)OC
Zolazepam	C15H15FN4O	286.1224	FC1=C(C=CC=C1)C=1C2=C(N(C(CN1)=O)C)N(N=C2C)C
Zolpidem	C19H21N3O	307.1679	CN(C(CC1=C(N=C2N1C=C(C=C2)C)C2=CC=C(C=C2)C)=O)C
Zomebazam	C15H16N4O2	284.1268	CN1N=C(C2=C1N(C(CC(N2C2=CC=CC=C2)=O)=O)C)C
Zonisamide	C8H8N2O3S	212.0250	NS(=O)(=O)CC1=NOC2=CC=CC=C12
Zopiclone	C17H17ClN6O3	388.1045	O=C(N1CCN(C)CC1)OC(C2=NC=CN=C23)N(C4=NC=C(Cl)C=C4)C3=O

**Table S10.** Fragment ions for psychoactive substances imported into the *Compound Class Scoring* node		
Formula	*m/z*	Structure
**Fentanyl Analogs**		
$C_{13}H_{18}N^+$	188.1434	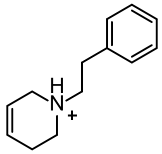 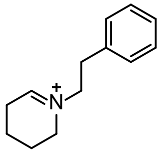 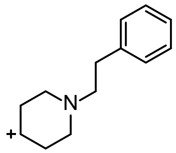
$C_9H_{12}N^+$	134.0965	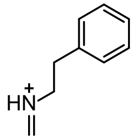
$C_{10}H_{12}N^+$	146.0965	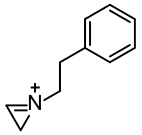
$C_9H_{10}N^+$	132.0808	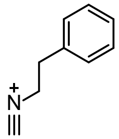
$C_5H_{10}N^+$	84.0808	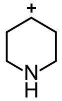
$C_{12}H_{16}N^+$	174.1278	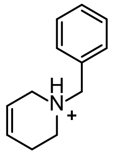 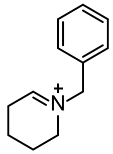 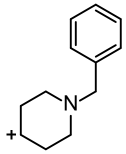

**Table S10.** Fragment ions for psychoactive substances imported into the *Compound Class Scoring* node (continued)			
Formula	*m/z*	Structure	
$C_{11}H_{16}NS^+$	194.0998	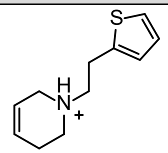<br>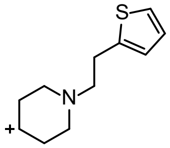	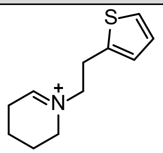
**Synthetic Cannabinoids**			
$C_{10}H_9O^+$	145.0648	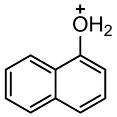	
$C_{11}H_7O^+$	155.0492	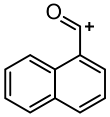	
$C_9H_6NO^+$	144.0444	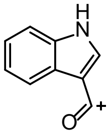	
$C_8H_6N^+$	116.0495	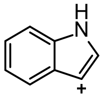	
$C_8H_5N_2O^+$	145.0397	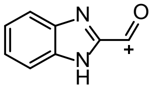	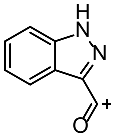
$C_7H_5N_2^+$	117.0448	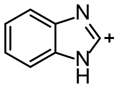	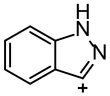
$C_8H_7N_2O_2^+$	163.0503	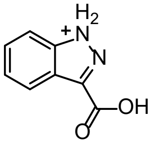	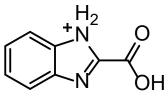

**Table S10.** Fragment ions for psychoactive substances imported into the *Compound Class Scoring* node (continued)			
Formula	*m/z*	Structure	
**Synthetic Cathinones**			
$C_9H_9N^{•+}$	131.0730	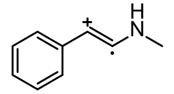	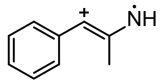
		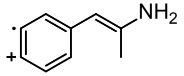	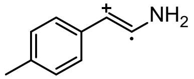
$C_9H_8N^{+}$	130.0652	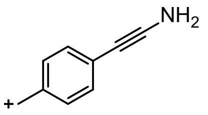	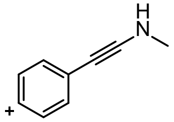
$C_{10}H_{11}N^{•+}$	145.0886	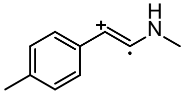	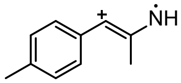
		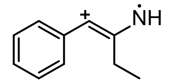	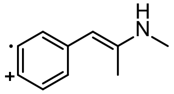
		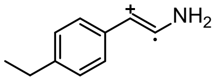	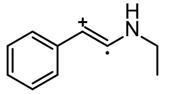
		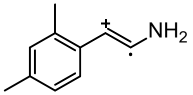	
$C_{10}H_{10}N^{+}$	144.0808	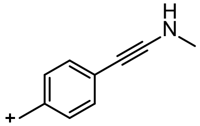	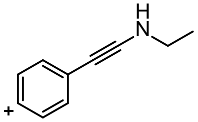
		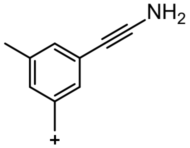	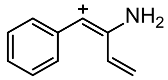
		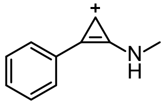	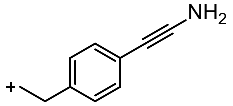

Table S10. Fragment ions for psychoactive substances imported into the *Compound Class Scoring* node (continued)

Formula	*m/z*	Structure	
$C_{11}H_{13}N^{\bullet+}$	159.1043	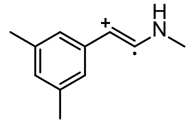	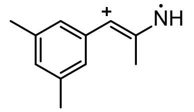
		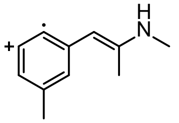	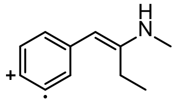
		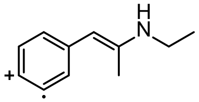	
$C_{11}H_{12}N^{+}$	158.0965	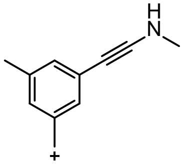	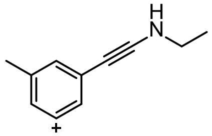
		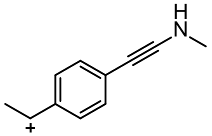	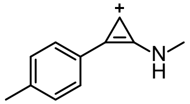
		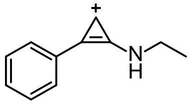	
$C_9H_9O_2^{+}$	149.0598	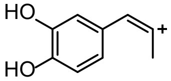	
$C_8H_5O_3^{+}$	149.0234	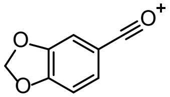	
$C_9H_7O_2^{+}$	147.0441	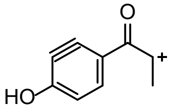	
$C_8H_7O_2^{+}$	135.0441	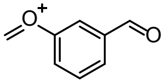	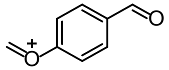

Table S10. Fragment ions for psychoactive substances imported into the *Compound Class Scoring* node (continued)

Formula	*m/z*	Structure
$C_{10}H_{11}NO^{\bullet+}$	161.0836	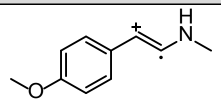
$C_{11}H_{13}NO_2^{\bullet+}$	191.0941	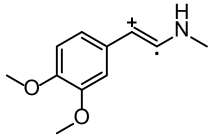
$C_9H_9O^+$	133.0648	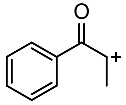
$C_7H_5O^+$	105.0335	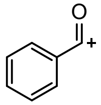
$C_9H_8FN^{\bullet+}$	149.0636	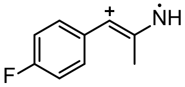
$C_8H_8F^+$	123.0605	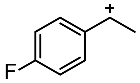
**Phenyl-Substituted Phenethylamines**		
$C_{10}H_{12}O_2^{\bullet+}$	164.0832	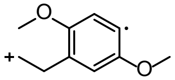
$C_9H_9O_2^+$	149.0598	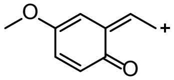 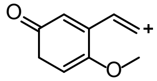 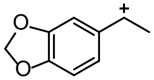
$C_9H_{10}O^{\bullet+}$	134.0727	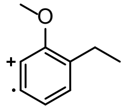
$C_8H_7O^+$	119.0492	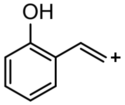
$C_{11}H_{14}O_2^{\bullet+}$	178.0989	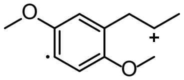

**Table S10.** Fragment ions for psychoactive substances imported into the *Compound Class Scoring* node (continued)		
Formula	*m/z*	Structure
$C_{10}H_{11}O_2^+$	163.0754	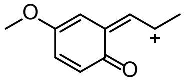 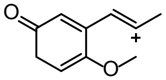 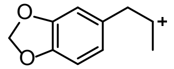
$C_{10}H_{11}O^+$	147.0805	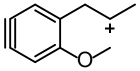
$C_9H_{11}O^+$	135.0805	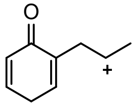
$C_8H_7O_2^+$	135.0441	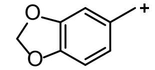
$C_9H_9O^+$	133.0648	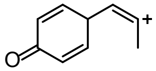
$C_{11}H_{11}O^+$	159.0805	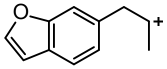
$C_{10}H_7O_2^+$	159.0441	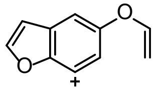
$C_9H_7O^+$	131.0492	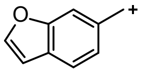 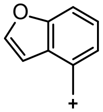
**Arylcyclohexylamines**		
$C_{12}H_{11}O^+$	171.0805	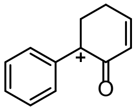
$C_{12}H_{13}O^+$	173.0961	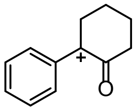
$C_{14}H_{18}NO^+$	216.1383	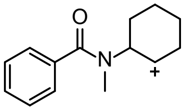

Table S10. Fragment ions for psychoactive substances imported into the *Compound Class Scoring* node (continued)		
Formula	*m/z*	Structure
**Indolealkylamines (Lysergamides and Tryptamines)**		
$C_{15}H_{15}N_2^+$	223.1230	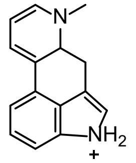
$C_{14}H_{10}NO^+$	208.0757	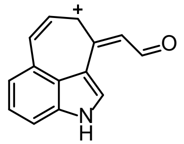
$C_{13}H_{10}N^+$	180.0808	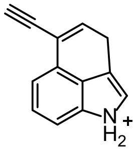
$C_{10}H_{10}N^+$	144.0808	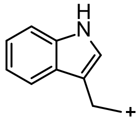
$C_9H_8N^+$	130.0652	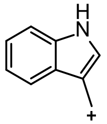

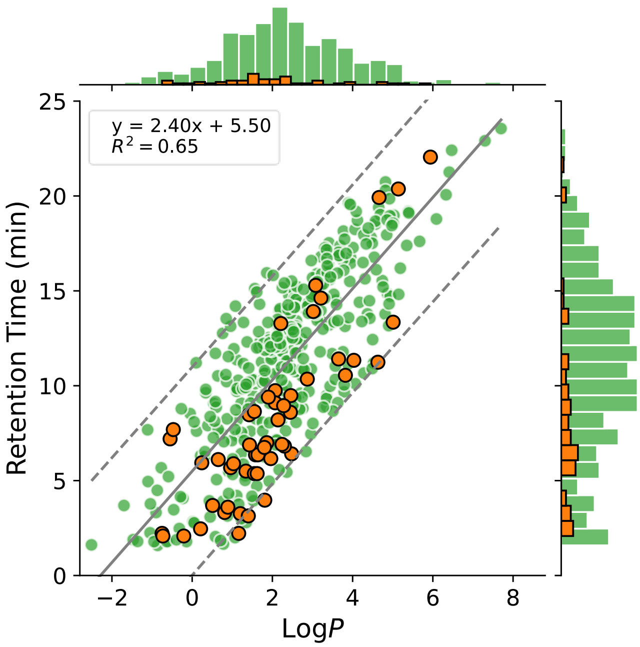

Figure S1. Linear regression between the chromatographic retention times and predicted Log*P* of 432 compounds analyzed under the same conditions as target substances (n = 51; the orange circles; Table S4). The grey solid line represents the linear regression line. The grey dashed lines bracket the 95% confidence interval of the linear regression line.

S4. Stability factors, sorption data, and excretion rates of target substances

Table S11. Summary of literature data on the in-sample and in-sewer stability of target substances

Compound Name	Min (%)	Max (%)	Median (%)	Count	References (In-Sample)	References (In-Sewer)
Fentanyl	-61.5	0.6	-12.1	12	[5]	[6]
Norfentanyl	-19.9	1.9	-6.8	8	[5]	NA
Meperidine	-32.0	3.2	-18.9	3	NA	[7]
Normeperidine	NA	NA	NA	NA	NA	NA
Codeine	-98.6	95.0	5.7	38	[5, 8-10]	[11-16]
Norcodeine	-4.5	4.5	-2.7	8	[5]	NA
Hydrocodone	-67.6	2.2	-24.3	3	NA	[7]
Norhydrocodone	NA	NA	NA	NA	NA	NA
Morphine	-61.1	74.6	-0.9	32	[5, 8-10, 17]	[6, 11, 14, 15, 18]
Hydromorphone	-28.6	5.6	-19.9	3	NA	[7]
Normorphine	-0.7	14.4	7.9	8	[5]	NA
Morphine-3-glucuronide (M3G)	-98.3	-5.7	-84.7	9	[5, 9]	NA
Heroin	-95.5	-21.4	-63.4	9	[5, 17]	NA
6-Monoacetylmorphine (6-MAM)	-98.9	21.0	-25.7	32	[5, 8-10, 19, 20]	[11, 14, 21, 22]
Oxycodone	-4.0	63.0	8.3	13	[5, 10]	[12]
Noroxycodone	1.0	243.6	121.3	2	NA	[16]
Oxymorphone	-59.6	31.2	18.2	11	[5]	[7]
Noroxymorphone	NA	NA	NA	NA	NA	NA
Dihydrocodeine	-6.6	9.4	-0.1	8	[5]	NA
Dihydromorphine	NA	NA	NA	NA	NA	NA
Desomorphine	NA	NA	NA	NA	NA	NA
Buprenorphine	-32.0	71.0	2.8	15	[5, 10]	[12, 15]
Norbuprenorphine	0.1	7.5	3.7	8	[5]	NA
Methadone	-99.9	76.0	-9.6	41	[5, 8-10, 17, 19]	[11, 12, 14, 15, 18]
2-Ethylidene-1,5-dimethyl-3,3-diphenylpyrrolidine (EDDP)	-95.6	30.4	-13.0	24	[5, 9, 10, 19]	[11, 14, 22]
Tramadol	-19.5	-6.0	-13.4	8	[5]	NA
O-Desmethyltramadol	-31.9	-8.4	-20.5	3	NA	[7]
N-Desmethyltramadol	-61.1	-8.4	-34.4	11	[5]	[7]
Naloxone	-67.2	13.7	-24.0	3	NA	[7]
Diphenhydramine	NA	NA	NA	NA	NA	NA
Alprazolam	NA	NA	NA	NA	NA	NA
α-Hydroxyalprazolam	NA	NA	NA	NA	NA	NA
Diazepam	-36.7	-3.0	-23.4	11	[5]	[23]
Nordiazepam	1.9	21.5	14.5	8	[5]	NA
Cocaine	-96.7	-0.2	-30.6	55	[5, 8-10, 17, 19, 20, 24, 25]	[6, 11, 14, 18, 21, 22, 26]
Benzoylecgonine	-77.7	59.5	2.7	47	[5, 8-10, 17, 19, 20, 24, 25]	[11, 14, 18, 21, 22]
Ecgonine Methyl Ester	-72.4	16.6	-54.3	3	[20, 24]	NA
Norcocaine	-20.6	-3.7	-9.9	9	[5, 24]	NA
3,4-Methylenedioxymethamphetamine (MDMA)	-82.7	7.3	-3.9	42	[5, 8-10, 19, 25]	[11, 14, 18, 21, 22]
3,4-Methylenedioxyamphetamine (MDA)	-15.0	3.7	-1.6	15	[5, 8, 10, 25]	NA
Methamphetamine	-71.9	8.1	-3.3	33	[5, 8-10, 19]	[11, 14, 18, 21, 22]
Amphetamine	-95.7	73.8	0.4	41	[5, 8-10, 17, 19]	[6, 11, 14, 22]
Nicotine	-81.4	14.0	-1.2	19	[5, 27]	[15, 16, 28, 29]
Cotinine	-17.1	70.0	12.0	22	[5, 27]	[15, 16, 26, 28, 29]
trans-3'-Hydroxycotinine	-10.8	38.2	2.5	13	NA	[15, 16, 26, 28, 29]
Δ^9-Tetrahydrocannabinol (THC)	NA	NA	NA	NA	NA	NA
11-nor-9-Carboxy-Δ^9-tetrahydrocannabinol (THC-COOH)	-10.7	7.3	-1.7	2	[9, 17]	NA
11-Hydroxy-Δ^9-tetrahydrocannabinol (11-OH-THC)	-24.9	-24.9	-24.9	1	[9]	NA
Caffeine	-3.4	22.9	10.2	8	[5]	NA
Paraxanthine	-26.0	1.1	-16.8	8	[5]	NA
Sucralose	0.0	0.0	0.0	2	[30]	NA

Table S12. Summary of literature data on sorption of target substances to suspended particulate matter					
Compound Name	Min (%)	Max (%)	Median (%)	Count	References
Fentanyl	17.2	17.2	17.2	1	[31]
Norfentanyl	NA	NA	NA	NA	NA
Meperidine	NA	NA	NA	NA	NA
Normeperidine	NA	NA	NA	NA	NA
Codeine	1.7	5.2	2.7	15	[31-33]
Norcodeine	3.5	4.4	4.0	3	[31]
Hydrocodone	NA	NA	NA	NA	NA
Norhydrocodone	NA	NA	NA	NA	NA
Morphine	1.5	6.0	3.4	9	[31, 33, 34]
Hydromorphone	NA	NA	NA	NA	NA
Normorphine	2.9	3.4	3.2	4	[31]
Morphine-3-glucuronide (M3G)	NA	NA	NA	NA	NA
Heroin	NA	NA	NA	NA	NA
6-Monoacetylmorphine (6-MAM)	NA	NA	NA	NA	NA
Oxycodone	NA	NA	NA	NA	NA
Noroxycodone	NA	NA	NA	NA	NA
Oxymorphone	NA	NA	NA	NA	NA
Noroxymorphone	NA	NA	NA	NA	NA
Dihydrocodeine	1.4	3.6	1.8	13	[31, 32]
Dihydromorphine	NA	NA	NA	NA	NA
Desomorphine	NA	NA	NA	NA	NA
Buprenorphine	NA	NA	NA	NA	NA
Norbuprenorphine	NA	NA	NA	NA	NA
Methadone	5.7	41.1	12.2	16	[31-34]
2-Ethylidene-1,5-dimethyl-3,3-diphenylpyrrolidine (EDDP)	11.9	49.8	25.7	16	[31-34]
Tramadol	1.5	4.7	2.7	13	[31, 32]
O-Desmethyltramadol	1.1	12.6	6.9	8	[31, 32]
N-Desmethyltramadol	NA	NA	NA	NA	NA
Naloxone	NA	NA	NA	NA	NA
Diphenhydramine	NA	NA	NA	NA	NA
Alprazolam	NA	NA	NA	NA	NA
α-Hydroxyalprazolam	NA	NA	NA	NA	NA
Diazepam	NA	NA	NA	NA	NA
Nordiazepam	NA	NA	NA	NA	NA
Cocaine	0.9	12.8	1.8	18	[31-35]
Benzoylecgonine	0.0	3.0	0.3	18	[31-35]
Ecgonine Methyl Ester	NA	NA	NA	NA	NA
Norcocaine	NA	NA	NA	NA	NA
3,4-Methylenedioxymethamphetamine (MDMA)	1.0	3.3	1.4	13	[31-35]
3,4-Methylenedioxyamphetamine (MDA)	18.0	34.3	26.2	2	[34, 35]
Methamphetamine	1.6	6.3	2.0	9	[32, 35]
Amphetamine	1.6	40.1	3.7	7	[31, 32, 34]
Nicotine	NA	NA	NA	NA	NA
Cotinine	NA	NA	NA	NA	NA
trans-3'-Hydroxycotinine	NA	NA	NA	NA	NA
Δ^9-Tetrahydrocannabinol (THC)	7.5	20.4	13.9	2	[36]
11-nor-9-Carboxy-Δ^9-tetrahydrocannabinol (THC-COOH)	5.1	44.2	18.2	4	[33, 36]
11-Hydroxy-Δ^9-tetrahydrocannabinol (11-OH-THC)	12.2	36.3	21.6	4	[33, 36]
Caffeine	NA	NA	NA	NA	NA
Paraxanthine	NA	NA	NA	NA	NA
Sucralose	NA	NA	NA	NA	NA

**Table S13.** Summary of literature data on the excretion rates of target substances					
Compound Name	Min	Max	Median	Count	References
Fentanyl	0.0300	0.0800	0.0320	3	[37-39]
Norfentanyl	0.4000	0.4050	0.4025	2	[37, 39]
Meperidine	0.0500	0.1300	0.0600	3	[38-40]
Normeperidine	0.1400	0.1400	0.1400	1	[40]
Codeine	0.2900	0.3000	0.2950	2	[41, 42]
Norcodeine	0.0510	0.0510	0.0510	1	[37]
Hydrocodone	0.0900	0.1200	0.1020	3	[38, 39, 43]
Norhydrocodone	0.1920	0.1920	0.1920	1	[43]
Morphine	0.4800	0.7460	0.5420	4	[37, 44]
Hydromorphone	0.3203	0.3600	0.3402	2	[39, 40]
Normorphine	0.0500	0.0500	0.0500	1	[37]
Morphine-3-glucuronide (M3G)	NA	NA	NA	NA	NA
Heroin	0.0003	0.0010	0.0006	2	[37, 45]
6-Monoacetylmorphine (6-MAM)	0.0046	0.0130	0.0055	4	[37, 44, 45]
Oxycodone	0.0810	0.2200	0.1900	5	[37-39, 43, 46]
Noroxycodone	0.1750	0.2310	0.2030	2	[37, 43]
Oxymorphone	0.0850	0.4540	0.1070	3	[37, 40, 43]
Noroxymorphone	0.0042	0.1420	0.0510	3	[37, 40, 43]
Dihydrocodeine	0.5400	0.5400	0.5400	1	[37]
Dihydromorphine	0.0630	0.0630	0.0630	1	[37]
Desomorphine	NA	NA	NA	NA	NA
Buprenorphine	0.0100	0.0500	0.0102	3	[37-39]
Norbuprenorphine	0.0965	0.0965	0.0965	1	[37]
Methadone	0.1970	0.2780	0.2400	5	[37, 38, 42, 44, 45]
2-Ethylidene-1,5-dimethyl-3,3-diphenylpyrrolidine (EDDP)	0.5000	0.5500	0.5250	2	[41, 42]
Tramadol	0.2900	0.3190	0.3000	3	[37, 39, 47]
O-Desmethyltramadol	0.1870	0.2000	0.1935	2	[37, 39]
N-Desmethyltramadol	0.1700	0.1700	0.1700	1	[40]
Naloxone	NA	NA	NA	NA	NA
Diphenhydramine	0.0200	0.0200	0.0200	1	[38]
Alprazolam	0.2000	0.2000	0.2000	1	[48]
α-Hydroxyalprazolam	NA	NA	NA	NA	NA
Diazepam	0.0050	0.0050	0.0050	1	[38]
Nordiazepam	0.0700	0.0700	0.0700	1	[37]
Cocaine	0.0100	0.0500	0.0153	3	[37, 38, 45]
Benzoylecgonine	0.2920	0.3070	0.3058	3	[37, 49, 50]
Ecgonine Methyl Ester	0.1300	0.4050	0.1500	3	[45, 51, 52]
Norcocaine	0.0004	0.0004	0.0004	1	[37]
3,4-Methylenedioxymethamphetamine (MDMA)	0.1578	0.2600	0.2140	4	[37, 45, 50, 53]
3,4-Methylenedioxyamphetamine (MDA)	0.0180	0.0180	0.0180	1	[53]
Methamphetamine	0.2270	0.4090	0.2856	3	[50, 53]
Amphetamine	0.2912	0.3630	0.3271	2	[50, 53]
Nicotine	0.1300	0.1670	0.1650	3	[38, 54, 55]
Cotinine	0.2700	0.3230	0.3000	3	[54-56]
trans-3'-Hydroxycotinine	0.4340	0.4450	0.4400	3	[54-56]
Δ^9-Tetrahydrocannabinol (THC)	0.0001	0.0001	0.0001	1	[53]
11-nor-9-Carboxy-Δ^9-tetrahydrocannabinol (THC-COOH)	0.0050	0.0674	0.0355	4	[45, 48, 57, 58]
11-Hydroxy-Δ^9-tetrahydrocannabinol (11-OH-THC)	0.0200	0.0200	0.0200	1	[45]
Caffeine	0.2090	0.2090	0.2090	1	[59]
Paraxanthine	0.2210	0.2210	0.2210	1	[59]
Sucralose	1.0000	1.0000	1.0000	1	[60]

**Table S14.** Substance-specific parameter estimates and standard errors for Monte Carlo simulations							
Compound Name	Molecular Weight	Stability (%)	Std Error	Sorption (%)	Std Error	Excretion Rate	Std Error
Fentanyl	336.48	-19.1	6.4	17.2	3.4[b]	0.0473	0.0163
Norfentanyl	232.33	-6.4	2.8	3.3[a]	0.7[b]	0.4025	0.0025
Meperidine	247.34	-15.9	10.3	NA	NA	0.0800	0.0252
Normeperidine	233.31	NA	NA	NA	NA	0.1400	0.0140
Codeine	299.37	-2.7	6.3	2.9	0.3	0.2950	0.0050
Norcodeine	285.34	-2.0	1.1	4.0	0.3	0.0510	0.0210
Hydrocodone	299.37	-29.9	20.3	NA	NA	0.1040	0.0087
Norhydrocodone	285.34	NA	NA	NA	NA	0.1920	0.0220
Morphine	285.34	5.3	6.1	3.6	0.5	0.5775	0.0581
Hydromorphone	285.34	-14.3	10.3	NA	NA	0.3402	0.0199
Normorphine	271.32	7.5	1.8	3.2	0.1	0.0500	0.0100[b]
Morphine-3-glucuronide (M3G)	461.47	-74.3	9.7	NA	NA	NA	NA
Heroin	369.42	-58.2	8.4	NA	NA	0.0006	0.0004
6-Monoacetylmorphine (6-MAM)	327.38	-36.1	6.2	NA	NA	0.0072	0.0020
Oxycodone	315.37	11.5	5.4	NA	NA	0.1540	0.0287
Noroxycodone	301.34	11.7	10.7	NA	NA	0.2030	0.0280
Oxymorphone	301.34	6.5	8.8	NA	NA	0.2153	0.1195
Noroxymorphone	287.32	NA	NA	NA	NA	0.0657	0.0405
Dihydrocodeine	301.39	-0.5	2.0	2.1	0.2	0.5400	0.0680
Dihydromorphine	287.36	NA	NA	NA	NA	0.0630	0.0370
Desomorphine	271.36	NA	NA	NA	NA	NA	NA
Buprenorphine	467.65	10.2	7.2	NA	NA	0.0234	0.0133
Norbuprenorphine	413.56	3.7	0.9	NA	NA	0.0965	0.0193[b]
Methadone	309.45	-21.1	6.0	14.1	2.0	0.2420	0.0156
2-Ethylidene-1,5-dimethyl-3,3-diphenylpyrrolidine (EDDP)	278.42	-14.4	5.6	25.2	2.7	0.5250	0.0250
Tramadol	263.38	-13.1	1.7	2.9	0.3	0.3030	0.0085
O-Desmethyltramadol	249.35	-20.3	6.8	6.8	1.7	0.1935	0.0065
N-Desmethyltramadol	249.35	-35.6	5.3	5.3[a]	1.1[b]	0.1700	0.0340[b]
Naloxone	327.38	-25.8	23.4	NA	NA	NA	NA
Diphenhydramine	255.36	NA	NA	NA	NA	0.0200	0.0040[b]
Alprazolam	308.77	NA	NA	NA	NA	0.2000	0.0400[b]
α-Hydroxyalprazolam	324.77	NA	NA	NA	NA	NA	NA
Diazepam	284.74	-20.2	2.9	NA	NA	0.0050	0.0010[b]
Nordiazepam	270.72	12.9	2.5	NA	NA	0.0700	0.0140[b]
Cocaine	303.36	-38.0	4.0	2.5	0.6	0.0251	0.0125
Benzoylecgonine	289.33	1.3	3.8	0.6	0.2	0.3016	0.0048
Ecgonine Methyl Ester	199.25	-36.7	27.2	NA	NA	0.2283	0.0885
Norcocaine	289.33	-11.0	2.0	NA	NA	0.0004	0.0001
3,4-Methylenedioxymethamphetamine (MDMA)	193.25	-11.9	3.2	1.7	0.2	0.2115	0.0214
3,4-Methylenedioxyamphetamine (MDA)	179.22	-1.6	1.3	26.2	8.2	0.0180	0.0036
Methamphetamine	149.24	-6.0	2.4	2.8	0.6	0.3072	0.0536
Amphetamine	135.21	-16.8	7.0	9.2	5.2	0.3271	0.0359
Nicotine	162.24	-11.0	6.4	NA	NA	0.1540	0.0120
Cotinine	176.22	18.0	5.2	NA	NA	0.2977	0.0153
trans-3'-Hydroxycotinine	192.22	5.1	4.1	NA	NA	0.4397	0.0032
Δ^9-Tetrahydrocannabinol (THC)	314.47	NA	NA	13.9	6.5	0.0001	0.0000
11-nor-9-Carboxy-Δ^9-tetrahydrocannabinol (THC-COOH)	344.45	-1.7	9.0	21.4	8.6	0.0359	0.0134
11-Hydroxy-Δ^9-tetrahydrocannabinol (11-OH-THC)	330.47	-24.9	5.0[b]	22.9	5.0	0.0200	0.0040[b]
Caffeine	194.19	11.1	3.4	NA	NA	0.2090	0.0600
Paraxanthine	180.17	-14.9	3.8	NA	NA	0.2210	0.0400
Sucralose	397.63	0.0	0.0	NA	NA	1.0000	0.2000[b]

[a] Estimated based on Sorption (%) = [0.031(±0.006) × Log$P$ - 0.011(±0.019)] × 100% (R^2 = 0.63). [b] Assumed 20% of the parameter estimate.[49]

S5. Target screening of substances in wastewater samples

Table S15. Predicted Log*P* and *p*K_a of target substances

Compound Name	SMILES	Log*P*	*p*K_a (Strongest Acidic)	*p*K_a (Strongest Basic)
Fentanyl	CCC(=O)N(C1CCN(CC1)CCC2=CC=CC=C2)C3=CC=CC=C3	3.82	-	8.46
Norfentanyl	CCC(=O)N(C1CCNCC1)C2=CC=CC=C2	1.42	-	10.02
Meperidine	CCOC(=O)C1(CCN(CC1)C)C2=CC=CC=C2	2.46	-	8.14
Normeperidine	CCOC(=O)C1(CCNCC1)C2=CC=CC=C2	2.07	-	9.31
Codeine	CN1CC[C@]23[C@@H]4[C@H]1CC5=C2C(=C(C=C5)OC)O[C@H]3[C@H](C=C4)O	1.34	-	8.89
Norcodeine	COC1=C2C3=C(C[C@@H]4[C@H]5[C@]3(CCN4)[C@@H](O2)[C@H](C=C5)O)C=C1	0.96	-	9.76
Hydrocodone	CN1CC[C@]23[C@@H]4[C@H]1CC5=C2C(=C(C=C5)OC)O[C@H]3C(=O)CC4	1.96	-	8.86
Norhydrocodone	COC1=C2C3=C(C[C@@H]4[C@H]5[C@]3(CCN4)[C@@H](O2)C(=O)CC5)C=C1	1.58	-	9.82
Morphine	CN1CC[C@]23[C@@H]4[C@H]1CC5=C2C(=C(C=C5)O)O[C@H]3[C@H](C=C4)O	1.20	-	8.64
Hydromorphone	CN1CC[C@]23[C@@H]4[C@H]1CC5=C2C(=C(C=C5)O)O[C@H]3C(=O)CC4	1.81	-	9.42
Normorphine	C1CN[C@@H]2CC3=C4[C@@]15[C@H]2C=C[C@@H]([C@@H]5OC4=C(C=C3)O)O	0.81	-	9.96
M3G	CN1CC[C@]23[C@@H]4[C@H]1CC5=C2C(=C(C=C5)O[C@H]6[C@@H]([C@H]([C@@H]([C@H](O6)C(=O)O)O)O)O)O[C@H]3[C@H](C=C4)O	-0.75	2.67	8.87
Heroin	CC(=O)O[C@H]1C=C[C@H]2[C@H]3CC4=C5[C@]2([C@H]1OC5=C(C=C4)OC(=O)C)CCN3C	1.55	-	8.80
6-MAM	CC(=O)O[C@H]1C=C[C@H]2[C@H]3CC4=C5[C@]2([C@H]1OC5=C(C=C4)O)CCN3C	1.64	-	9.43
Oxycodone	CN1CC[C@]23[C@@H]4C(=O)CC[C@]2([C@H]1CC5=C3C(=C(C=C5)OC)O4)O	1.03	-	8.07
Noroxycodone	COC1=C2C3=C(C[C@@H]4[C@]5([C@]3(CCN4)[C@@H](O2)C(=O)CC5)O)C=C1	0.65	-	9.26
Oxymorphone	CN1CC[C@]23[C@@H]4C(=O)CC[C@]2([C@H]1CC5=C3C(=C(C=C5)O)O4)O	0.89	-	8.01
Noroxymorphone	C1C[C@]2([C@H]3CC4=C5[C@@]2(CCN3)[C@H](C1=O)OC5=C(C=C4)O)O	0.51	-	9.53
Dihydrocodeine	CN1CC[C@]23[C@@H]4[C@H]1CC5=C2C(=C(C=C5)OC)O[C@H]3[C@H](CC4)O	1.55	-	9.03
Dihydromorphine	CN1CC[C@]23[C@@H]4[C@H]1CC5=C2C(=C(C=C5)O)O[C@H]3[C@H](CC4)O	1.40	-	9.60
Desomorphine	CN1CC[C@]23[C@@H]4[C@H]1CC5=C2C(=C(C=C5)O)O[C@H]3CCC4	2.48	-	9.72
Buprenorphine	C[C@]([C@H]1C[C@@]23CC[C@@]1([C@H]4[C@@]25CCN([C@@H]3CC6=C5C(=C(C=C6)O)O4)CC7CC7)OC)(C(C)(C)C)O	4.03	-	10.11
Norbuprenorphine	C[C@]([C@H]1C[C@@]23CC[C@@]1([C@H]4[C@@]25CCN[C@@H]3CC6=C5C(=C(C=C6)O)O4)OC)(C(C)(C)C)O	2.87	-	10.24
(±)-Methadone	CCC(=O)C(CC(C)N(C)C)(C1=CC=CC=C1)C2=CC=CC=C2	5.01	-	9.11
EDDP	C/C=C\1/C(CC(N1C)C)(C2=CC=CC=C2)C3=CC=CC=C3	4.63	-	10.99
(+)-Tramadol	CN(C)C[C@H]1CCCC[C@@]1(C2=CC(=CC=C2)OC)O	2.45	-	9.38
(+)-*O*-Desmethyltramadol	CN(C)C[C@H]1CCCC[C@@]1(C2=CC(=CC=C2)O)O	2.30	-	9.69
(+)-*N*-Desmethyltramadol	CNC[C@H]1CCCC[C@@]1(C2=CC(=CC=C2)OC)O	2.07	-	9.89
Naloxone	C=CCN1CC[C@]23[C@@H]4C(=O)CC[C@]2([C@H]1CC5=C3C(=C(C=C5)O)O4)O	1.62	-	7.67
Diphenhydramine	CN(C)CCOC(C1=CC=CC=C1)C2=CC=CC=C2	3.65	-	8.86
Alprazolam	CC1=NN=C2N1C3=C(C=C(C=C3)Cl)C(=NC2)C4=CC=CC=C4	3.02	-	1.45
α-Hydroxyalprazolam	C1C2=NN=C(N2C3=C(C=C(C=C3)Cl)C(=N1)C4=CC=CC=C4)CO	2.21	-	-0.12
Diazepam	CN1C(=O)CN=C(C2=C1C=CC(=C2)Cl)C3=CC=CC=C3	3.08	-	2.92
Nordiazepam	C1C(=O)NC2=C(C=C(C=C2)Cl)C(=N1)C3=CC=CC=C3	3.21	-	2.85
Cocaine	CN1[C@H]2CC[C@@H]1[C@H]([C@H](C2)OC(=O)C3=CC=CC=C3)C(=O)OC	2.28	-	8.94
Benzoylecgonine	CN1[C@H]2CC[C@@H]1[C@H]([C@H](C2)OC(=O)C3=CC=CC=C3)C(=O)O	2.14	3.15	9.14
Ecgonine Methyl Ester	CN1[C@H]2CC[C@@H]1[C@H]([C@H](C2)O)C(=O)OC	-0.21	-	9.14
(-)-Norcocaine	COC(=O)[C@@H]1[C@H]2CC[C@H](N2)C[C@@H]1OC(=O)C3=CC=CC=C3	1.90	-	8.96

Table S15. Predicted Log*P* and pK_a of target substances (continued)

Compound Name	SMILES	Log*P*	pK_a (Strongest Acidic)	pK_a (Strongest Basic)
(±)-MDMA	CC(CC1=CC2=C(C=C1)OCO2)NC	1.86	-	10.13
(±)-MDA	CC(CC1=CC2=C(C=C1)OCO2)N	1.43	-	9.93
(±)-Methamphetamine	CC(CC1=CC=CC=C1)NC	2.24	-	10.21
(±)-Amphetamine	CC(CC1=CC=CC=C1)N	1.80	-	10.03
(-)-Nicotine	CN1CCC[C@H]1C2=CN=CC=C2	1.16	-	2.70
(-)-Cotinine	CN1[C@@H](CCC1=O)C2=CN=CC=C2	0.21	-	4.79
trans-3'-Hydroxycotinine	CN1[C@@H](C[C@H](C1=O)O)C2=CN=CC=C2	-0.73	-	4.79
THC	CCCCCC1=CC(=C2[C@@H]3C=C(CC[C@H]3C(OC2=C1)(C)C)C)O	5.94	9.34	-
THC-COOH	CCCCCC1=CC(=C2[C@@H]3C=C(CC[C@H]3C(OC2=C1)(C)C)C(=O)O)O	5.14	4.21	-
11-OH-THC	CCCCCC1=CC(=C2[C@@H]3C=C(CC[C@H]3C(OC2=C1)(C)C)CO)O	4.66	9.34	-
Caffeine	CN1C=NC2=C1C(=O)N(C(=O)N2C)C	-0.55	-	-1.16
Paraxanthine	CN1C=NC2=C1C(=O)N(C(=O)N2)C	0.24	-	-1.10
Sucralose	C([C@@H]1[C@@H]([C@@H]([C@H]([C@H](O1)O[C@]2([C@H]([C@@H]([C@H](O2)CCl)O)O)CCl)O)O)Cl)O	-0.47	-	-

Log*P* (predicted by Consensus model using ChemAxon and Klopman's models and the PhysProp database with 0 electrolyte concentration) and pK_a (predicted in macro mode with static acid/base prefix and a minimal basic pK_a of -2 and a maximum acidic pK_a of 12 at 298.15 K) predicted by *MarvinSketch 23.10.0* (ChemAxon Ltd.).

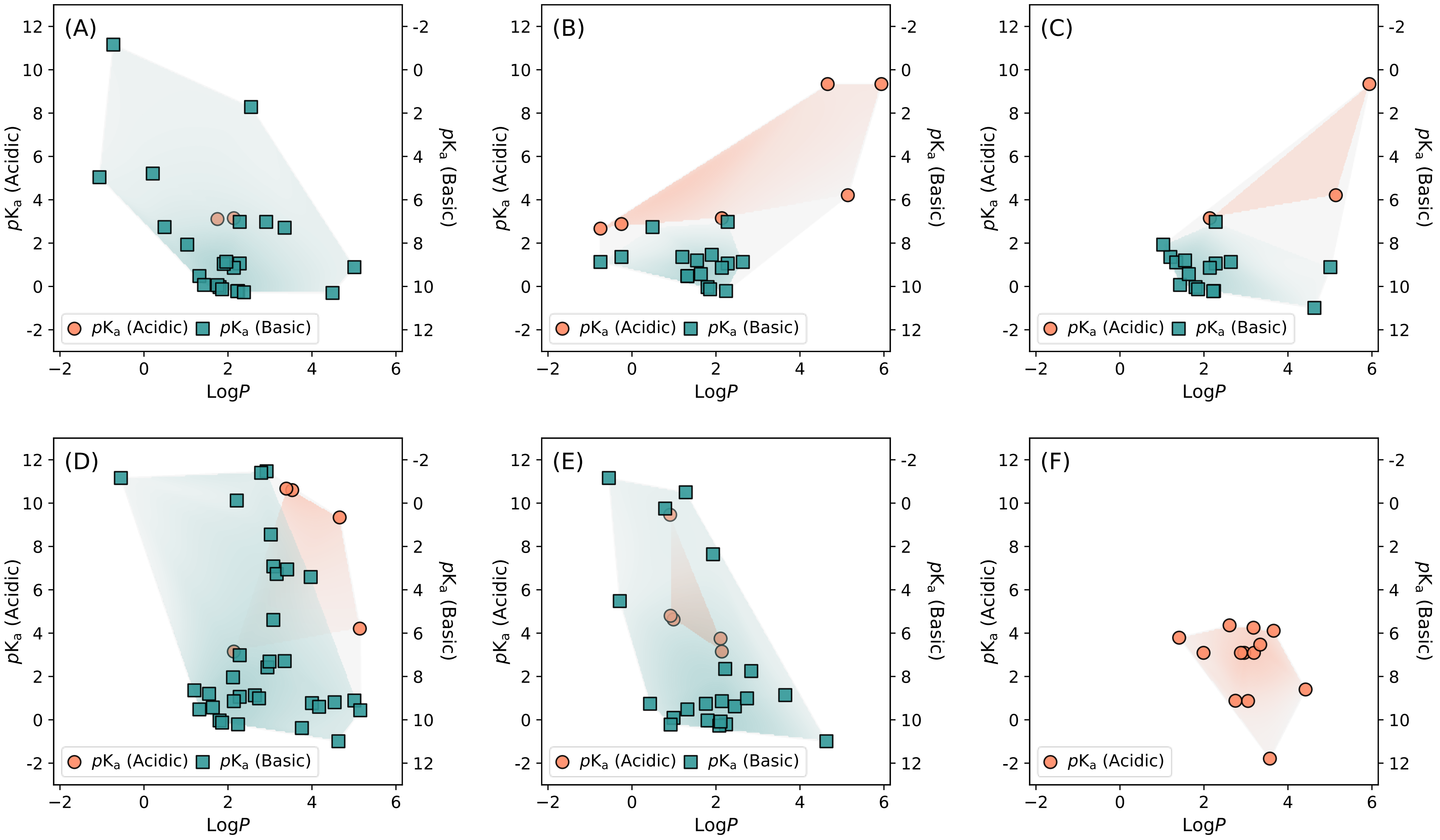

Figure S2. Comparison of the analytical space covered by online SPE-LC-HRMS methods developed in previous WBE studies: **(A)** Target substances (n = 23) validated by Chiaia *et al.*[61] **(B)** Target substances (n = 19) validated by Postigo *et al.*[62] **(C)** Target substances (n = 18) validated by Heuett *et al.*[63] **(D)** Target substances (n = 37) validated by López-García *et al.*[64] **(E)** Target substances (n = 27) validated by Wang *et al.*[65] **(F)** Target substances (n = 29) validated by Senta *et al.*[66] Shaded areas highlight the analytical space defined by target substances. Strongest acidic pK_a values are plotted on the left y-axis, whereas strongest basic pK_a values are plotted on the right y-axis (reversed). Note that substances lacking ionizable atoms (within the range of a minimal basic pK_a of -2 and a maximum acidic pK_a of 12) are not plotted.

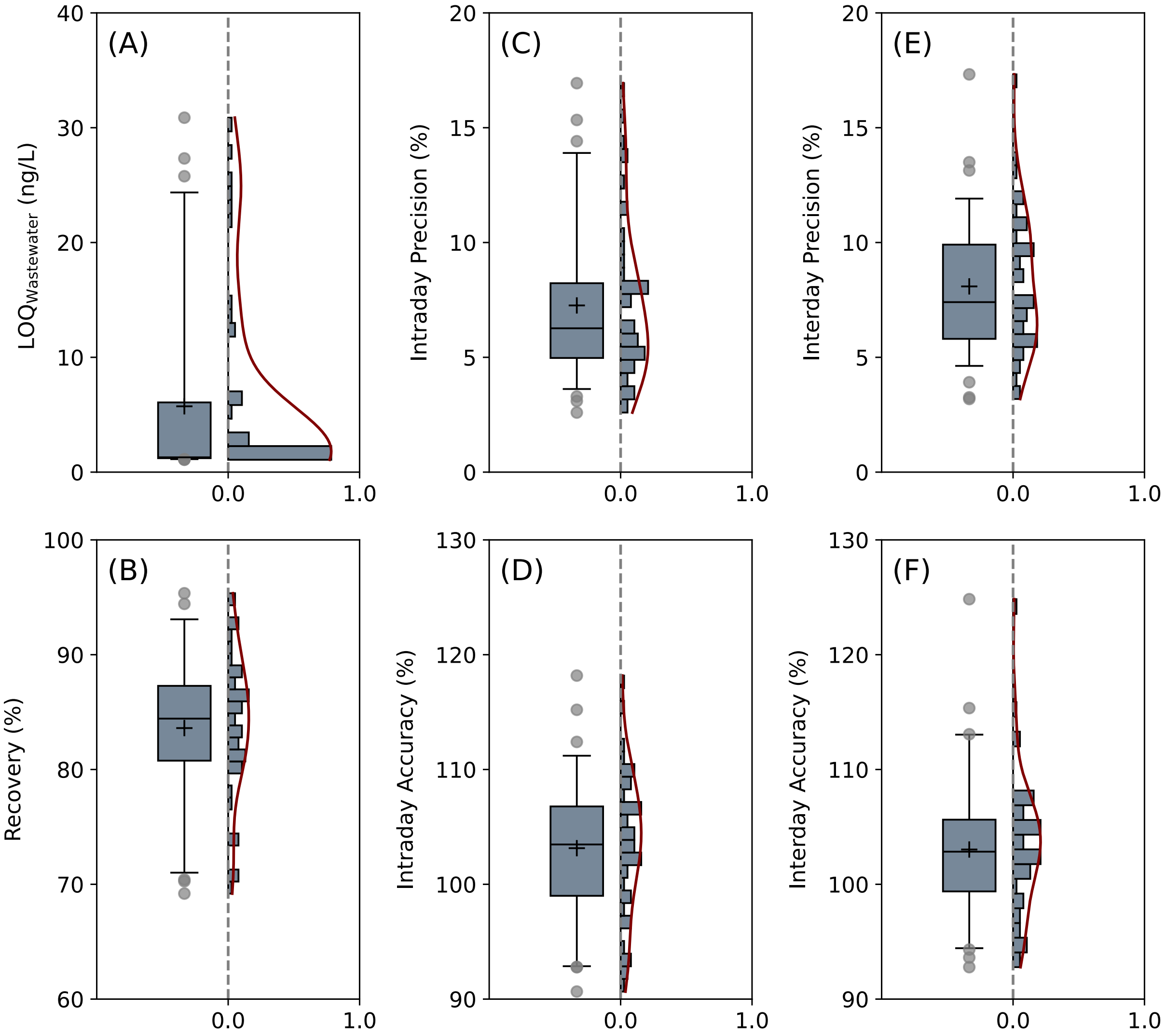

Figure S3. Online SPE-LC-HRMS method performance for target substances (n = 51): **(A)** Limit of quantification in wastewater; **(B)** Recovery; **(C)** Intraday precision; **(D)** Intraday accuracy; **(E)** Interday precision; and **(F)** Interday accuracy. Substance-specific method performance data are summarized in Table S6. On each plot, the box spans from the 25th to the 75th percentiles. The whiskers extend from the box to the 5th and 95th percentiles. The centerline and "+" sign mark the median and mean, respectively. The filled grey circles represent outliers. The maroon solid curve represents the kernel density estimate plot.

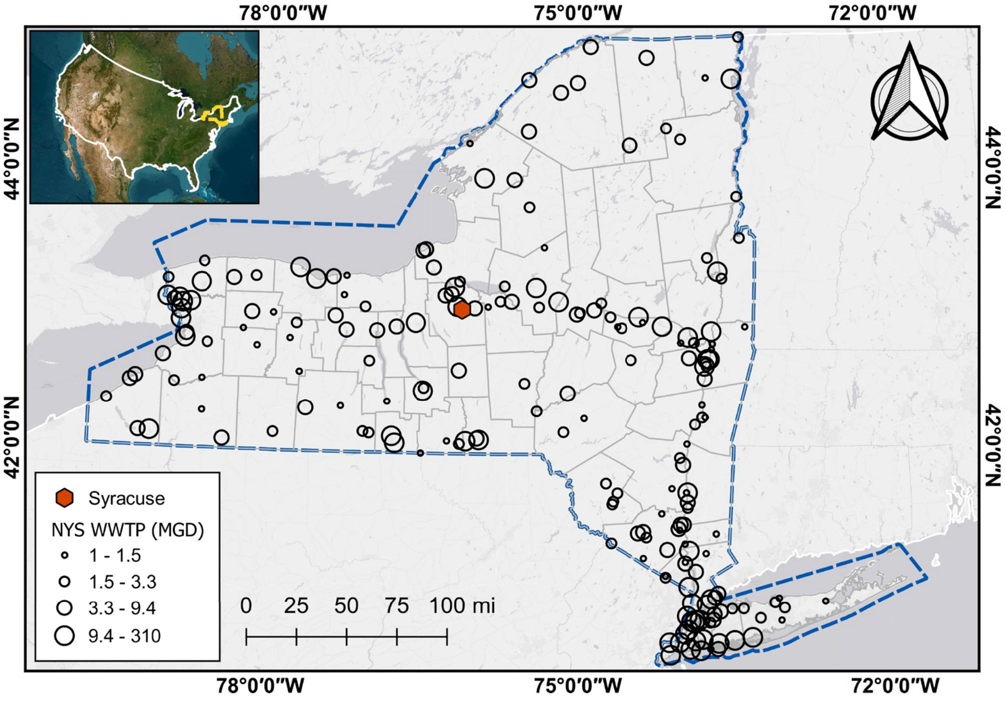

Figure S4. Municipal WWTPs with an average design hydraulic flow of ≥1 million gallons per day (n = 201) in New York State.[67] Satellite Image Sources: Esri, DigitalGlobe, GeoEye, i-cubed, USDA FSA, USGS, AEX, Getmapping, Aerogrid, IGN, IGP, swisstopo, and the GIS User Community.

Table S16. Concentration ranges and detection frequencies of target substances in wastewater samples					
Compound Name	Min (ng/L)	Mean (ng/L)	Median (ng/L)	Max (ng/L)	Detection Frequency
Fentanyl	3	4	4	8	94.3%
Norfentanyl	14	28	27	73	96.6%
Meperidine	-	-	-	-	1.2%[a]
Normeperidine	-	-	-	-	2.9%[a]
Codeine	10	48	33	405	82.1%
Norcodeine	-	-	-	-	2.9%[a]
Hydrocodone	-	-	-	-	7.0%[a]
Norhydrocodone	-	-	-	-	0.0%[b]
Morphine	-	-	-	-	0.4%[a]
Hydromorphone	-	-	-	-	0.0%[b]
Normorphine	-	-	-	-	0.0%[b]
Morphine-3-glucuronide (M3G)	-	-	-	-	0.0%[b]
Heroin	-	-	-	-	0.0%[b]
6-Monoacetylmorphine (6-MAM)	-	-	-	-	0.0%[b]
Oxycodone	-	-	-	-	7.6%[a]
Noroxycodone	-	-	-	-	5.3%[a]
Oxymorphone	-	-	-	-	0.0%[b]
Noroxymorphone	-	-	-	-	0.0%[b]
Dihydrocodeine	-	-	-	-	0.0%[b]
Dihydromorphine	-	-	-	-	0.4%[a]
Desomorphine	-	-	-	-	0.0%[b]
Buprenorphine	-	-	-	-	6.6%[a]
Norbuprenorphine	-	-	-	-	6.4%[a]
Methadone	21	61	57	216	100.0%
2-Ethylidene-1,5-dimethyl-3,3-diphenylpyrrolidine (EDDP)	44	113	91	445	100.0%
Tramadol	144	612	531	3178	100.0%
O-Desmethyltramadol	35	271	219	1963	100.0%
N-Desmethyltramadol	21	103	89	319	100.0%
Naloxone	-	-	-	-	0.4%[a]
Diphenhydramine	60	708	674	1562	100.0%
Alprazolam	-	-	-	-	1.8%[a]
α-Hydroxyalprazolam	-	-	-	-	1.6%[a]
Diazepam	-	-	-	-	1.0%[a]
Nordiazepam	-	-	-	-	2.9%[a]
Cocaine	61	271	239	2332	100.0%
Benzoylecgonine	202	912	849	4625	100.0%
Ecgonine Methyl Ester	-	-	-	-	0.0%[b]
Norcocaine	-	-	-	-	2.9%[a]
3,4-Methylenedioxymethamphetamine (MDMA)	-	-	-	-	10.9%[a]
3,4-Methylenedioxyamphetamine (MDA)	-	-	-	-	0.4%[a]
Methamphetamine	51	262	166	1844	100.0%
Amphetamine	65	269	259	727	100.0%
Nicotine	259	3410	3354	8695[c]	100.0%
Cotinine	135	1571	1406	4942	100.0%
trans-3’-Hydroxycotinine	864	2402	2371	4924	75.6%
Δ^9-Tetrahydrocannabinol (THC)	-	-	-	-	0.0%[b]
11-nor-9-Carboxy-Δ^9-tetrahydrocannabinol (THC-COOH)	29	392	301	2882	100.0%
11-Hydroxy-Δ^9-tetrahydrocannabinol (11-OH-THC)	54	213	150	2228	55.0%
Caffeine	12166	68813[c]	64884[c]	198402[c]	100.0%
Paraxanthine	7027	35984[c]	34569[c]	116682[c]	100.0%
Sucralose	8806	39280[c]	33766[c]	115464[c]	100.0%

[a] Target substance detected but not quantified due to the low detection frequency. [b] Target substance not detected at quantifiable levels. [c] Concentration extrapolated beyond the calibration range of 1-5000 ng/L or 500-25000 ng/L.

S6. Nontarget screening of substances in wastewater samples

Table S17. Mass spectral features confirmed at confidence level 1 by reference standards

Compound Name	Molecular Formula	ΔMass [ppm]	Exact Mass	*m/z*	RT [min]	Max Area	Adduct	*mzCloud* Best Match	*mzVault* Best Match
				Fragment Ion #1	Fragment Ion #2	Fragment Ion #3	Fragment Ion #4		
Dimethyl phthalate	$C_{10}H_{10}O_4$	-1.23	194.0577	195.0647	11.58	4.33E+08	[M+H]+	91.7	95.2
				163.0387	135.0439	133.0282	105.0445		
	Reference standard			195.0652	11.57				
				163.0390	135.0441	133.0284	105.0445		
Ephedrine	$C_{10}H_{15}NO$	-0.55	165.1153	166.1225	4.32	1.92E+08	[M+H]+	75.6	64.2
				91.0541	148.1112	56.0493	-		
	Reference standard			166.1226	4.36				
				91.0537	148.1121	135.0804	117.0697		
Pseudoephedrine	$C_{10}H_{15}NO$	-0.43	165.1153	166.1226	6.54	2.56E+08	[M+H]+	94.9	95.0
				148.1119	117.0698	133.0885	115.0541		
	Reference standard			166.1227	6.50				
				148.1121	117.0699	133.0886	115.0542		
N-Butylbenzenesulfonamide	$C_{10}H_{15}NO_2S$	-0.56	213.0822	214.0895	13.78	9.48E+07	[M+H]+	30.5	70.4
				158.0265	140.9998	77.0386	105.0440		
	Reference standard			214.0897	14.17				
				158.0268	141.0003	77.0385	105.0446		
Cimetidine	$C_{10}H_{16}N_6S$	-0.31	252.1156	253.1230	5.10	7.12E+07	[M+H]+	85.8	89.2
				95.0603	159.0697	117.0481	99.0665		
	Reference standard			253.1230	5.14				
				95.0604	159.0699	117.0481	99.0665		
Camphor	$C_{10}H_{16}O$	-0.40	152.1201	153.1274	15.32	6.44E+08	[M+H]+	95.4	76.1
				69.0699	135.1169	93.0699	107.0855		
	Reference standard			153.1274	15.30				
				69.0699	135.1168	93.0699	107.0855		
Sulfapyridine	$C_{11}H_{11}N_3O_2S$	-0.45	249.0571	250.0644	6.25	2.00E+07	[M+H]+	84.9	81.8
				92.0493	156.0113	108.0442	184.0866		
	Reference standard			250.0645	6.29				
				92.0487	156.0114	108.0438	184.0864		
N-Ethylamphetamine (**Figure S7**)	$C_{11}H_{17}N$	-0.50	163.1360	164.1433	8.76	3.47E+08	[M+H]+	87.9	37.8
				119.0854	91.0542	117.0696	104.0621		
	Reference standard			164.1434	8.80				
				119.0855	164.1434	91.0542	117.0698		
Diphenylphosphinic acid	$C_{12}H_{11}O_2P$	-0.18	218.0496	219.0567	11.43	5.98E+08	[M+H]+	89.8	-
				159.0204	201.0463	141.0098	105.0446		
	Reference standard			219.0569	11.46				
				159.0206	201.0464	141.0100	105.0447		

Table S17. Mass spectral features confirmed at confidence level 1 by reference standards (continued)

Compound Name	Molecular Formula	ΔMass [ppm]	Exact Mass	*m/z*	RT [min]	Max Area	Adduct	*mzCloud* Best Match	*mzVault* Best Match
				Fragment Ion #1	Fragment Ion #2	Fragment Ion #3	Fragment Ion #4		
N4-Acetylsulfamethoxazole	$C_{12}H_{13}N_3O_4S$	-0.15	295.0626	296.0699	9.65	1.96E+08	[M+H]+	94.2	96.2
				134.0600	65.0386	108.0443	198.0219		
	Reference standard			296.0700	9.68				
				134.0600	65.0386	108.0444	198.0219		
Metaxalone	$C_{12}H_{15}NO_3$	-0.11	221.1052	222.1124	13.46	6.34E+07	[M+H]+	92.8	93.4
				161.0961	135.0805	105.0698	123.0804		
	Reference standard			222.1126	13.47				
				161.0962	135.0805	105.0700	123.0805		
N,N-Diethyl-3-methylbenzamide (DEET)	$C_{12}H_{17}NO$	-1.02	191.1308	192.1382	14.09	2.60E+10	[M+H]+	94.5	95.1
				119.0491	91.0541	72.0444	100.0756		
	Reference standard			192.1383	14.06				
				119.0491	91.0542	72.0444	100.0757		
Icaridin	$C_{12}H_{23}NO_3$	-0.47	229.1677	230.1751	15.02	6.68E+08	[M+H]+	95.6	97.8
				130.1226	112.1120	95.0855	174.1126		
	Reference standard			230.1751	15.05				
				130.1226	112.1121	95.0855	174.1125		
Benzophenone	$C_{13}H_{10}O$	-0.71	182.0730	183.0804	15.76	2.23E+08	[M+H]+	91.6	91.9
				105.0334	77.0385	95.0491	-		
	Reference standard			183.0804	15.80				
				105.0335	77.0383	95.0491	-		
Fluconazole	$C_{13}H_{12}F_2N_6O$	0.10	306.1041	307.1117	9.48	7.06E+07	[M+H]+	94.0	96.3
				220.0680	238.0785	169.0458	70.0399		
	Reference standard			307.1113	9.52				
				220.0681	238.0786	169.0460	70.0400		
N,N′-Diphenylguanidine (1,3-Diphenylguanidine)	$C_{13}H_{13}N_3$	-0.62	211.1108	212.1181	8.23	1.78E+09	[M+H]+	97.3	96.7
				119.0603	94.0651	195.0917	92.0494		
	Reference standard			212.1182	8.24				
				119.0604	94.0651	195.0917	92.0493		
Bupropion	$C_{13}H_{18}ClNO$	0.03	239.1077	240.1149	10.34	9.56E+07	[M+H]+	96.1	97.6
				184.0523	131.0728	166.0417	57.0698		
	Reference standard			240.1143	10.33				
				184.0524	131.0730	166.0418	57.0699		
Hydroxybupropion	$C_{13}H_{18}ClNO_2$	0.00	255.1026	256.1098	9.85	8.73E+07	[M+H]+	92.8	-
				238.0992	139.0307	131.0729	167.0494		
	Reference standard			256.1092	9.84				
				238.0993	139.0309	131.0730	167.0496		
N-Ethyl-*p*-menthane-3-carboxamide	$C_{13}H_{25}NO$	-0.28	211.1936	212.2008	16.76	3.78E+08	[M+H]+	-	97.3
				170.1540	83.0854	72.0443	69.0698		
	Reference standard			212.2008	16.70				
				170.1539	83.0855	72.0443	69.0698		

Table S17. Mass spectral features confirmed at confidence level 1 by reference standards (continued)

Compound Name	Molecular Formula	ΔMass [ppm]	Exact Mass	*m/z*	RT [min]	Max Area	Adduct	*mzCloud* Best Match	*mzVault* Best Match
				Fragment Ion #1	Fragment Ion #2	Fragment Ion #3	Fragment Ion #4		
Diclofenac	$C_{14}H_{11}Cl_2NO_2$	-0.45	295.0166	296.0244	18.01	4.12E+07	[M+H]+	92.3	95.2
				250.0185	278.0130	214.0418	215.0496		
	Reference standard			296.0240	18.03				
				250.0185	278.0134	214.0418	215.0496		
Oxybenzone	$C_{14}H_{12}O_3$	-0.43	228.0786	229.0859	17.39	7.65E+07	[M+H]+	93.6	93.1
				151.0389	105.0335	95.0490	77.0385		
	Reference standard			229.0859	17.43				
				151.0389	105.0335	95.0491	77.0386		
Naproxen	$C_{14}H_{14}O_3$	-0.48	230.0942	231.1014	15.48	5.56E+07	[M+H]+	90.4	97.1
				185.0960	170.0725	153.0696	155.0853		
	Reference standard			231.1016	15.50				
				185.0961	170.0726	153.0699	155.0855		
Trimethoprim	$C_{14}H_{18}N_4O_3$	-0.18	290.1378	291.1452	7.25	2.47E+08	[M+H]+	94.7	96.0
				230.1164	123.0665	261.0982	275.1139		
	Reference standard			291.1452	7.20				
				230.1162	123.0665	261.0982	275.1139		
Abacavir	$C_{14}H_{18}N_6O$	-0.22	286.1542	287.1613	7.37	1.34E+08	[M+H]+	93.2	-
				191.1038	79.0542	150.0647	174.0773		
	Reference standard			287.1615	7.39				
				191.1040	79.0542	150.0649	174.0774		
Atenolol acid / Metoprolol acid	$C_{14}H_{21}$NO4	-0.57	267.1469	268.1543	7.20	9.77E+08	[M+H]+	95.4	94.2
				145.0646	56.0495	191.0701	72.0807		
	Reference standard			268.1543	7.19				
				145.0648	56.0495	191.0703	72.0808		
Lidocaine	$C_{14}H_{22}N_2O$	-0.04	234.1732	235.1806	7.89	7.39E+08	[M+H]+	99.0	98.0
				86.0964	58.0652	105.8729	79.1044		
	Reference standard			235.1805	7.88				
				86.0964	58.0651	-	-		
Atenolol	$C_{14}H_{22}N_2O_3$	-0.58	266.1629	267.1699	5.02	2.47E+08	[M+H]+	93.8	93.8
				145.0646	74.0599	190.0860	56.0494		
	Reference standard			267.1703	5.00				
				145.0648	74.0600	190.0863	56.0495		
Tapentadol (**Figure S5**)	$C_{14}H_{23}NO$	0.15	221.1780	222.1850	9.51	1.03E+08	[M+H]+	94.1	-
				107.0490	121.0647	135.0803	77.0386		
	Reference standard			222.1852	9.52				
				107.0491	121.0648	135.0804	77.0383		
Daidzein	$C_{15}H_{10}O_4$	0.04	254.0579	255.0652	11.73	8.19E+07	[M+H]+	95.4	94.3
				199.0754	137.0233	181.0648	227.0706		
	Reference standard			255.0649	11.76				
				199.0755	137.0233	181.0644	227.0710		

Table S17. Mass spectral features confirmed at confidence level 1 by reference standards (continued)

Compound Name	Molecular Formula	ΔMass [ppm]	Exact Mass	*m/z* / Fragment Ion #1	RT [min] / Fragment Ion #2	Max Area / Fragment Ion #3	Adduct / Fragment Ion #4	*mzCloud* Best Match	*mzVault* Best Match
Carbamazepine	$C_{15}H_{12}N_2O$	-0.17	236.0949	237.1021	12.97	2.13E+08	[M+H]+	96.3	97.2
				194.0963	192.0808	179.0730	220.0759		
	Reference standard			237.1022	12.97				
				194.0964	192.0808	179.0726	220.0757		
1,3-Di-*o*-tolylguanidine	$C_{15}H_{17}N_3$	-0.39	239.1422	240.1495	9.47	8.92E+08	[M+H]+	97.8	92.8
				133.0760	108.0807	106.0650	116.0495		
	Reference standard			240.1495	9.50				
				133.0760	108.0808	106.0651	116.0495		
Metoprolol	$C_{15}H_{25}NO_3$	-0.08	267.1834	268.1906	9.12	6.80E+08	[M+H]+	95.6	94.4
				74.0599	116.1069	56.0495	98.0963		
	Reference standard			268.1907	9.15				
				74.0600	116.1070	56.0495	98.0964		
Hexa(methoxymethyl)melamine	$C_{15}H_{30}N_6O_6$	-0.10	390.2226	391.2299	13.07	1.71E+08	[M+H]+	87.3	92.2
				177.0881	207.0987	283.1511	359.2035		
	Reference standard			391.2305	13.07				
				177.0884	207.0990	283.1516	359.2041		
Sitagliptin	$C_{16}H_{15}F_6N_5O$	-0.29	407.1180	408.1251	9.59	1.05E+08	[M+H]+	91.2	94.2
				174.0524	193.0694	235.0801	154.0461		
	Reference standard			408.1254	9.57				
				174.0525	193.0696	235.0801	154.0463		
Piperine	$C_{17}H_{19}NO_3$	-0.12	285.1365	286.1437	16.66	8.45E+08	[M+H]+	95.5	98.2
				201.0546	135.0441	115.0542	143.0491		
	Reference standard			286.1438	16.68				
				201.0546	135.0441	115.0542	143.0491		
Flecainide	$C_{17}H_{20}F_6N_2O_3$	-0.05	414.1378	415.1451	11.34	1.54E+08	[M+H]+	91.4	93.1
				398.1186	301.0295	98.0964	81.0699		
	Reference standard			415.1452	11.33				
				398.1185	301.0294	98.0964	81.0699		
Mycophenolic acid	$C_{17}H_{20}O_6$	-0.34	320.1259	321.1328	15.13	1.21E+08	[M+H]+	88.1	93.5
				207.0650	303.1226	159.0439	177.0545		
	Reference standard			321.1333	15.14				
				207.0652	303.1227	159.0440	177.0546		
Levorphanol (**Figure S6**)	$C_{17}H_{23}NO$	-0.05	257.1780	258.1851	8.76	1.71E+08	[M+H]+	97.2	95.8
				199.1117	201.1274	133.0647	157.0649		
	Reference standard			258.1852	8.77				
				199.1118	201.1274	133.0649	157.0651		
Venlafaxine	$C_{17}H_{27}NO_2$	-0.39	277.2041	278.2114	11.03	2.90E+08	[M+H]+	96.9	96.3
				58.0651	260.2008	121.0647	147.0805		
	Reference standard			278.2115	11.03				
				58.0651	260.2009	121.0648	147.0804		

**Table S17.** Mass spectral features confirmed at confidence level 1 by reference standards (continued)									
Compound Name	Molecular Formula	ΔMass [ppm]	Exact Mass	*m/z* / Fragment Ion #1	RT [min] / Fragment Ion #2	Max Area / Fragment Ion #3	Adduct / Fragment Ion #4	*mzCloud* Best Match	*mzVault* Best Match
Triphenylphosphine oxide	$C_{18}H_{15}OP$	-0.39	278.0859	279.0930	15.29	5.48E+07	[M+H]+	84.1	91.5
				219.0567	201.0461	173.0512	105.0446		
	Reference standard			279.0933	15.25				
				219.0569	201.0464	173.0515	105.0447		
Galaxolidone	$C_{18}H_{24}O_2$	-0.31	272.1776	273.1846	19.80	2.74E+08	[M+H]+	95.8	96.1
				255.1741	240.1507	225.1271	212.1557		
	Reference standard			273.1850	19.82				
				255.1742	240.1509	225.1273	212.1558		
Dextromethorphan	$C_{18}H_{25}NO$	0.13	271.1937	272.2007	11.53	5.37E+07	[M+H]+	94.7	90.3
				213.1274	215.1428	147.0803	171.0803		
	Reference standard			272.2009	11.56				
				213.1274	215.1430	147.0804	171.0805		
Tributyl citrate	$C_{18}H_{32}O_7$	-0.49	360.2146	361.2221	19.79	4.59E+08	[M+H]+	92.8	87.7
				129.0182	185.0808	259.1542	68.9971		
	Reference standard			361.2224	19.80				
				129.0184	185.0811	259.1542	68.9973		
Labetalol	$C_{19}H_{24}N_2O_3$	0.14	328.1787	329.1887	10.68	8.21E+07	[M+H]+	89.0	94.6
				91.0541	311.1752	162.0548	294.1487		
	Reference standard			329.1862	10.70				
				91.0543	311.1757	162.0550	294.1491		
Androstenedione	$C_{19}H_{26}O_2$	-0.43	286.1932	287.2005	15.63	3.60E+08	[M+H]+	92.9	96.9
				97.0647	109.0647	123.0803	79.0541		
	Reference standard			287.2006	15.62				
				97.0648	109.0648	123.0804	79.0542		
Testosterone	$C_{19}H_{28}O_2$	-0.04	288.2089	289.2161	16.75	4.92E+07	[M+H]+	90.9	93.7
				97.0647	109.0646	271.2054	123.0804		
	Reference standard			289.2162	16.79				
				97.0648	109.0648	271.2056	123.0804		
Lauramidopropyl betaine	$C_{19}H_{38}N_2O_3$	-0.80	342.2880	343.2957	19.08	5.61E+09	[M+H]+	-	94.1
				240.2322	57.0699	71.0854	109.1012		
	Reference standard			343.2955	19.08				
				240.2324	57.0698	71.0855	109.1012		
Citalopram	$C_{20}H_{21}FN_2O$	-0.24	324.1637	325.1709	11.36	2.33E+08	[M+H]+	94.7	96.8
				109.0447	262.1027	234.0711	116.0493		
	Reference standard			325.1711	11.37				
				109.0448	262.1027	234.0714	116.0495		
Quinine	$C_{20}H_{24}N_2O_2$	-0.27	324.1837	325.1910	8.39	2.70E+09	[M+H]+	92.1	91.2
				81.0698	307.1805	79.0542	172.0757		
	Reference standard			325.1911	8.40				
				81.0699	307.1805	79.0542	172.0757		

Table S17. Mass spectral features confirmed at confidence level 1 by reference standards (continued)

Compound Name	Molecular Formula	ΔMass [ppm]	Exact Mass	*m/z* / Fragment Ion #1	RT [min] / Fragment Ion #2	Max Area / Fragment Ion #3	Adduct / Fragment Ion #4	*mzCloud* Best Match	*mzVault* Best Match
Nobiletin	$C_{21}H_{22}O_8$	-0.27	402.1314	403.1386	16.02	5.17E+07	[M+H]+	81.0	88.3
				373.0916	183.0287	211.0235	327.0871		
	Reference standard			403.1374	16.05				
				373.0918	183.0288	211.0237	327.0863		
Cetirizine	$C_{21}H_{25}ClN_2O_3$	0.06	388.1554	389.1627	14.40	2.56E+08	[M+H]+	93.1	98.4
				201.0465	166.0776	187.1086	183.0804		
	Reference standard			389.1619	14.39				
				201.0466	166.0777	187.1077	183.0805		
Propafenone	$C_{21}H_{27}NO_3$	-0.29	341.1990	342.2063	13.39	6.67E+07	[M+H]+	95.3	62.1
				116.1069	72.0807	98.0963	324.1961		
	Reference standard			342.2064	13.42				
				116.1070	72.0813	98.0964	324.1958		
Norgestrel	$C_{21}H_{28}O_2$	-0.28	312.2088	313.2141	17.29	5.49E+07	[M+H]+	70.8	46.1
				295.2051	95.0854	133.1008	161.0955		
	Reference standard			313.2162	17.34				
				295.2056	95.0855	133.1012	161.0961		
Losartan	$C_{22}H_{23}ClN_6O$	-0.16	422.1621	423.1689	14.43	2.89E+08	[M+H]+	86.3	96.4
				207.0916	405.1587	180.0807	377.1523		
	Reference standard			423.1694	14.46				
				207.0916	405.1589	180.0807	377.1528		
Diltiazem	$C_{22}H_{26}N_2O_4S$	-0.30	414.1612	415.1686	12.29	1.61E+08	[M+H]+	92.2	95.9
				178.0320	150.0371	109.0105	72.0807		
	Reference standard			415.1682	12.34				
				178.0320	150.0372	109.0106	72.0808		
Valsartan	$C_{24}H_{29}N_5O_3$	-0.39	435.2269	436.2337	16.23	5.96E+08	[M+H]+	88.2	95.3
				207.0915	235.0980	291.1490	180.0806		
	Reference standard			436.2352	16.36				
				207.0917	235.0977	291.1492	180.0808		
Drospirenone	$C_{24}H_{30}O_3$	-0.80	366.2192	367.2260	15.54	3.20E+07	[M+H]+	79.3	55.4
				93.0698	349.2160	173.0961	107.0853		
	Reference standard			367.2268	15.56				
				93.0699	349.2162	173.0961	107.0855		
Irbesartan	$C_{25}H_{28}N_6O$	-0.27	428.2324	429.2398	14.81	4.03E+08	[M+H]+	91.5	97.6
				207.0915	180.0807	195.1491	84.0807		
	Reference standard			429.2402	14.86				
				207.0918	180.0809	195.1494	84.0808		
Darunavir	$C_{27}H_{37}N_3O_7S$	0.01	547.2352	548.2426	13.97	8.22E+07	[M+H]+	89.5	89.4
				69.0334	392.2001	113.0596	156.0113		
	Reference standard			548.2425	13.99				
				69.0335	392.2004	113.0597	156.0114		

Table S17. Mass spectral features confirmed at confidence level 1 by reference standards (continued)

Compound Name	Molecular Formula	ΔMass [ppm]	Exact Mass	*m/z* / Fragment Ion #1	RT [min] / Fragment Ion #2	Max Area / Fragment Ion #3	Adduct / Fragment Ion #4	*mzCloud* Best Match	*mzVault* Best Match
Fexofenadine	$C_{32}H_{39}NO_4$	0.49	501.2882	502.2950	13.53	1.19E+09	[M+H]+	93.0	94.6
				171.1167	466.2739	484.2843	131.0853		
	Reference standard			502.2940	13.54				
				171.1168	466.2741	484.2846	131.0855		
Telmisartan	$C_{33}H_{30}N_4O_2$	-0.41	514.2367	515.2440	14.71	5.33E+07	[M+H]+	91.8	96.8
				276.1369	497.2337	289.1450	211.0751		
	Reference standard			515.2442	14.72				
				276.1378	497.2336	289.1448	211.0754		
Metformin	$C_4H_{11}N_5$	-0.69	129.1014	130.1086	2.11	1.34E+08	[M+H]+	97.7	96.3
				60.0556	71.0603	85.0508	88.0868		
	Reference standard			130.1087	2.10				
				60.0556	71.0604	85.0509	88.0869		
Caprolactam	$C_6H_{11}NO$	-1.06	113.0839	114.0911	6.85	2.58E+09	[M+H]+	61.1	-
				69.0698	79.0542	96.0809	86.0962		
	Reference standard			114.0913	6.79				
				69.0699	79.0542	96.0808	86.0964		
Benzotriazole	$C_6H_5N_3$	-0.61	119.0483	120.0554	8.34	5.64E+08	[M+H]+	97.8	98.6
				65.0385	92.0494	66.0337	-		
	Reference standard			120.0556	8.35				
				65.0386	92.0495	66.0338	-		
1-Methylxanthine	$C_6H_6N_4O_2$	-0.40	166.0490	167.0563	3.65	2.29E+08	[M+H]+	94.4	91.1
				124.0505	96.0556	149.0455	150.0298		
	Reference standard			167.0560	3.65				
				124.0505	96.0556	149.0455	150.0292		
Simazine	$C_7H_{12}ClN_5$	-0.34	201.0781	202.0853	12.07	4.64E+07	[M+H]+	94.6	94.5
				132.0321	124.0869	104.0009	96.0555		
	Reference standard			202.0854	12.09				
				132.0323	124.0869	104.0010	96.0556		
Levocarnitine	$C_7H_{15}NO_3$	-0.78	161.1051	162.1123	1.80	1.17E+08	[M+H]+	98.3	96.4
				103.0389	60.0807	85.0283	57.0335		
	Reference standard			162.1125	1.94				
				103.0390	60.0808	85.0284	57.0333		
2-Hydroxybenzothiazole	C_7H_5NOS	-0.14	151.0092	152.0163	11.31	1.99E+08	[M+H]+	95.0	95.7
				124.0214	92.0494	109.0106	119.0363		
	Reference standard			152.0165	11.30				
				124.0216	92.0495	109.0107	119.0366		
Benzothiazole	C_7H_5NS	-0.51	135.0142	136.0213	11.57	5.29E+08	[M+H]+	95.9	96.7
				109.0106	77.0383	65.0389	105.0450		
	Reference standard			136.0217	11.59				
				109.0107	77.0387	65.0386	105.0449		

Table S17. Mass spectral features confirmed at confidence level 1 by reference standards (continued)

Compound Name	Molecular Formula	ΔMass [ppm]	Exact Mass	*m/z*	RT [min]	Max Area	Adduct	*mzCloud* Best Match	*mzVault* Best Match
				Fragment Ion #1	Fragment Ion #2	Fragment Ion #3	Fragment Ion #4		
5-Methyl-1H-benzotriazole	$C_7H_7N_3$	-0.65	133.0639	134.0711	10.42	1.74E+09	[M+H]+	98.0	97.8
				79.0542	106.0650	77.0385	95.0490		
	Reference standard			134.0713	10.44				
				79.0542	106.0651	77.0386	95.0490		
1,7-Dimethyluric acid	$C_7H_8N_4O_3$	-0.59	196.0595	197.0669	6.12	3.61E+08	[M+H]+	91.2	93.6
				140.0454	69.0447	182.0439	112.0507		
	Reference standard			197.0669	6.11				
				140.0455	69.0447	182.0434	112.0505		
Pyridoxine	$C_8H_{11}NO_3$	-0.55	169.0738	170.0811	2.26	6.14E+07	[M+H]+	88.2	95.9
				152.0705	134.0599	124.0757	-		
	Reference standard			170.0812	2.25				
				152.0706	134.0600	124.0757	-		
Atrazine	$C_8H_{14}ClN_5$	-0.92	215.0936	216.1009	13.80	1.11E+09	[M+H]+	97.3	98.2
				174.0539	96.0555	104.0009	132.0323		
	Reference standard			216.1011	13.82				
				174.0541	96.0556	104.0010	132.0323		
Pregabalin	$C_8H_{17}NO_2$	-0.38	159.1259	160.1332	6.78	1.73E+08	[M+H]+	96.3	96.8
				142.1226	55.0542	83.0855	97.1011		
	Reference standard			160.1332	6.80				
				142.1226	55.0542	83.0855	97.1012		
Acetaminophen	$C_8H_9NO_2$	-0.48	151.0633	152.0705	5.28	1.42E+09	[M+H]+	97.6	95.4
				110.0599	93.0334	65.0386	134.0598		
	Reference standard			152.0706	5.31				
				110.0600	93.0335	65.0386	134.0600		
Azelaic acid	$C_9H_{16}O_4$	-0.39	188.1048	189.1121	11.59	5.14E+07	[M+H]+	90.9	89.3
				187.0976	125.0973	97.0658	123.0817		
	Reference standard			189.1131	11.56				
				187.0976	125.0972	97.0659	123.0815		
Gabapentin	$C_9H_{17}NO_2$	-0.81	171.1258	172.1332	6.96	5.83E+09	[M+H]+	98.2	97.9
				154.1226	137.0961	55.0179	95.0855		
	Reference standard			172.1332	6.98				
				154.1226	137.0961	55.0178	95.0855		
Dexpanthenol	$C_9H_{19}NO_4$	-0.51	205.1313	206.1385	5.37	4.80E+08	[M+H]+	96.1	94.2
				76.0756	58.0651	188.1279	102.0548		
	Reference standard			206.1387	5.44				
				76.0757	58.0651	188.1281	102.0550		
Triisopropanolamine	$C_9H_{21}NO_3$	-0.77	191.1520	192.1593	2.10	4.53E+08	[M+H]+	96.9	93.5
				174.1488	98.0963	59.0491	156.1382		
	Reference standard			192.1594	2.11				
				174.1489	98.0964	59.0491	156.1383		

Table S17. Mass spectral features confirmed at confidence level 1 by reference standards (continued)

Compound Name	Molecular Formula	ΔMass [ppm]	Exact Mass	*m/z*	RT [min]	Max Area	Adduct	*mzCloud* Best Match	*mzVault* Best Match
				Fragment Ion #1	Fragment Ion #2	Fragment Ion #3	Fragment Ion #4		
Lamotrigine	$C_9H_7Cl_2N_5$	-0.03	255.0078	256.0151	9.72	1.23E+08	[M+H]+	97.1	94.3
				210.9824	186.9827	171.9715	166.0292		
	Reference standard			256.0151	9.78				
				210.9824	186.9824	171.9715	166.0292		
Phentermine	$C_{10}H_{15}N$	-0.28	149.1204	150.1277	8.47	1.61E+07	[M+H]+	93.1	95.5
				91.0541	133.1011	105.0698	55.0543		
	Reference standard			150.1279	8.48				
				91.0542	133.1012	105.0699	55.0546		
N-Desmethylvenlafaxine	$C_{16}H_{25}NO_2$	-0.20	263.1885	264.1956	11.23	1.07E+08	[M+H]+	94.7	95.3
				246.1852	121.0647	215.1428	147.0803		
	Reference standard			264.1958	11.25				
				246.1852	121.0648	215.1430	147.0804		
O-Desmethylvenlafaxine	$C_{16}H_{25}NO_2$	-0.45	263.1884	264.1956	8.76	4.11E+08	[M+H]+	94.1	97.2
				58.0651	246.1851	107.0491	133.0647		
	Reference standard			264.1958	8.78				
				58.0651	246.1852	107.0491	133.0648		
Amitriptyline	$C_{20}H_{23}N$	-0.09	277.1830	278.1902	13.82	2.25E+07	[M+H]+	96.9	98.3
				91.0542	105.0698	233.1325	117.0698		
	Reference standard			278.1903	13.84				
				91.0542	105.0699	233.1325	117.0699		
17α-Hydroxyprogesterone	$C_{21}H_{30}O_3$	-0.19	330.2194	331.2269	14.67	1.15E+08	[M+H]+	88.8	74.4
				271.2056	253.1950	105.0697	313.2168		
	Reference standard			331.2272	14.67				
				271.2060	253.1955	105.0701	313.2167		
Theophylline	$C_7H_8N_4O_2$	-0.57	180.0646	181.0719	6.36	1.78E+09	[M+H]+	92.2	89.3
				124.0503	96.0555	137.0821	69.0447		
	Reference standard			181.0720	6.34				
				124.0505	96.0554	137.0823	69.0447		

**Table S18.** Mass spectral features identified at confidence level 2 as probable structures									
Compound Name	Molecular Formula	ΔMass [ppm]	Exact Mass	*m/z*	RT [min]	Max Area	Adduct	*mzCloud* Best Match	*mzVault* Best Match
				Fragment Ion #1	Fragment Ion #2	Fragment Ion #3	Fragment Ion #4		
Inosine	$C_{10}H_{12}N_4O_5$	-0.70	268.0806	269.0879	2.81	5.73E+08	[M+H]+	91.6	95.6
				137.0457	110.0351	94.0397	57.0335		
Xanthosine	$C_{10}H_{12}N_4O_6$	-0.74	284.0755	285.0828	3.65	4.12E+08	[M+H]+	-	96.5
				153.0406	136.0139	133.0494	57.0335		
N-Methyl-2,3-dihydro-1H-inden-2-amine (*N*-Methyl-2-AI; **Figure S9**)	$C_{10}H_{13}N$	-0.57	147.1047	148.1119	6.46	2.85E+08	[M+H]+	91.0	-
				117.0697	115.0541	91.0541	133.0885		
2'-Deoxyadenosine	$C_{10}H_{13}N_5O_3$	-0.65	251.1017	252.1090	2.90	1.41E+09	[M+H]+	97.4	92.5
				136.0616	117.0547	99.0438	73.0283		
Adenosine	$C_{10}H_{13}N_5O_4$	-0.64	267.0966	268.1039	2.78	1.27E+09	[M+H]+	93.5	95.4
				136.0616	119.0351	57.0334	85.0281		
Guanosine	$C_{10}H_{13}N_5O_5$	-1.03	283.0914	284.0987	3.03	3.42E+09	[M+H]+	-	95.0
				152.0565	135.0300	110.0349	69.0335		
Thymidine	$C_{10}H_{14}N_2O_5$	-0.40	242.0902	243.0974	4.36	3.53E+08	[M+H]+	96.2	95.8
				127.0501	117.0547	99.0439	110.0236		
Cyclo(prolylvalyl)	$C_{10}H_{16}N_2O_2$	-0.37	196.1211	197.1284	7.31	2.88E+08	[M+H]+	89.1	-
				70.0651	72.0807	169.1334	124.1120		
Ethylenediaminetetraacetic acid (EDTA)	$C_{10}H_{16}N_2O_8$	-0.88	292.0904	293.0977	2.07	7.38E+09	[M+H]+	90.7	97.2
				160.0604	132.0654	114.0548	74.0599		
Citral	$C_{10}H_{16}O$	-0.08	152.1201	153.1274	13.40	1.05E+08	[M+H]+	92.6	65.7
				107.0854	135.1168	93.0698	81.0698		
Pulegone	$C_{10}H_{16}O$	-0.05	152.1201	153.1274	13.19	1.72E+08	[M+H]+	95.3	68.3
				107.0855	135.1168	93.0698	95.0854		
N-Cyclohexyl-2-pyrrolidone	$C_{10}H_{17}NO$	-0.59	167.1309	168.1382	12.60	1.44E+08	[M+H]+	-	96.7
				86.0600	83.0855	55.0542	69.0334		
Glutathione	$C_{10}H_{17}N_3O_6S$	-0.04	307.0838	308.0911	2.17	1.30E+07	[M+H]+	88.3	91.8
				76.0215	179.0483	84.0443	162.0219		
Valylproline	$C_{10}H_{18}N_2O_3$	-0.76	214.1316	215.1389	4.14	3.71E+09	[M+H]+	97.1	-
				116.0705	72.0807	70.0651	55.0542		
Eucalyptol	$C_{10}H_{18}O$	-0.38	154.1357	155.1430	16.96	4.87E+07	[M+H]+	92.7	-
				95.0854	81.0698	137.1323	67.0542		
Linalool	$C_{10}H_{18}O$	-0.42	154.1357	155.1430	18.22	3.18E+08	[M+H]+	-	88.3
				95.0854	81.0698	137.1323	85.0648		
ε-Decalactone	$C_{10}H_{18}O_2$	-0.53	170.1306	171.1379	15.10	3.91E+08	[M+H]+	-	94.7
				69.0699	153.1273	135.1168	55.0542		
Tetraglyme	$C_{10}H_{22}O_5$	-0.73	222.1466	223.1539	8.40	1.39E+09	[M+H]+	96.2	98.3
				59.0492	103.0753	147.1017	89.0595		
Pentaethylene glycol	$C_{10}H_{22}O_6$	-0.86	238.1414	239.1488	6.13	1.13E+09	[M+H]+	97.0	82.7
				89.0596	133.0859	177.1121	151.0969		
Kynurenic acid	$C_{10}H_7NO_3$	-0.23	189.0426	190.0498	7.26	5.87E+08	[M+H]+	90.5	98.0
				162.0549	144.0444	116.0494	172.0396		

Table S18. Mass spectral features identified at confidence level 2 as probable structures (continued)									
Compound Name	Molecular Formula	ΔMass [ppm]	Exact Mass	*m/z*	RT [min]	Max Area	Adduct	*mzCloud* Best Match	*mzVault* Best Match
				Fragment Ion #1	Fragment Ion #2	Fragment Ion #3	Fragment Ion #4		
2-Naphthylamine	$C_{10}H_9N$	-0.41	143.0734	144.0807	6.54	1.29E+09	[M+H]+	90.2	88.3
				117.0696	115.0540	127.0540	91.0540		
Indole-3-acetic acid	$C_{10}H_9NO_2$	-0.69	175.0632	176.0705	10.33	5.39E+08	[M+H]+	94.6	94.4
				130.0650	158.0603	103.0540	-		
2-(4-Morpholinyl)benzothiazole	$C_{11}H_{12}N_2OS$	-0.39	220.0670	221.0742	13.33	7.24E+07	[M+H]+	68.3	92.9
				177.0480	150.0373	136.0214	109.0108		
Tryptophan	$C_{11}H_{12}N_2O_2$	-0.80	204.0897	205.0970	6.50	5.55E+09	[M+H]+	97.6	99.3
				188.0706	146.0600	118.0651	132.0806		
N,N-Bis(2-hydroxyethyl)-*p*-toluidine	$C_{11}H_{17}NO_2$	-0.47	195.1258	196.1331	6.76	1.54E+07	[M+H]+	-	81.5
				134.0963	178.1227	120.0804	160.1121		
Leucylproline	$C_{11}H_{20}N_2O_3$	-0.31	228.1473	229.1546	5.86	1.97E+09	[M+H]+	92.2	61.4
				116.0705	86.0964	70.0651	-		
O-Butanoylcarnitine	$C_{11}H_{21}NO_4$	-0.57	231.1469	232.1542	4.76	2.05E+08	[M+H]+	57.9	90.5
				85.0283	173.0806	60.0807	71.0490		
Indole-3-acrylic acid	$C_{11}H_9NO_2$	-0.76	187.0632	188.0705	6.32	6.24E+09	[M+H]+	91.3	86.8
				146.0600	118.0650	170.0602	115.0542		
4,4’-Sulfonyldiphenol	$C_{12}H_{10}O_4S$	-0.34	250.0299	251.0372	9.91	3.93E+08	[M+H]+	93.6	97.0
				156.9953	121.0395	65.0385	109.0283		
1,2,3,4-Tetrahydro-beta-carboline-3-carboxylic acid	$C_{12}H_{12}N_2O_2$	-0.63	216.0897	217.0970	7.68	1.51E+09	[M+H]+	94.9	40.3
				144.0807	74.0236	171.0916	130.0653		
Diethyl phthalate	$C_{12}H_{14}O_4$	-0.36	222.0891	223.0964	14.36	9.65E+07	[M+H]+	91.3	92.8
				149.0231	177.0544	121.0282	65.0385		
Monoisobutyl phthalate	$C_{12}H_{14}O_4$	-0.06	222.0892	223.0965	19.67	6.30E+07	[M+H]+	81.8	85.1
				149.0231	121.0282	57.0698	65.0385		
2,4,6-Tris(allyloxy)-1,3,5-triazine	$C_{12}H_{15}N_3O_3$	-0.31	249.1113	250.1185	16.37	7.54E+07	[M+H]+	-	87.1
				81.0698	79.0542	170.0560	87.0189		
Triethyl citrate	$C_{12}H_{20}O_7$	-0.38	276.1208	277.1281	12.17	2.57E+08	[M+H]+	94.6	95.5
				157.0494	203.0915	115.0389	133.0496		
Sucrose	$C_{12}H_{22}O_{11}$	-0.70	342.1160	343.1233	2.05	1.20E+07	[M+H]+	95.2	95.1
				59.0139	89.0244	71.0139	179.0563		
Dimethyl sebacate	$C_{12}H_{22}O_4$	-0.32	230.1517	231.1590	13.21	1.99E+08	[M+H]+	91.4	-
				217.1433	139.1116	69.0698	121.1010		
Dicyclohexylamine	$C_{12}H_{23}N$	-1.24	181.1828	182.1901	9.48	1.62E+10	[M+H]+	-	98.9
				83.0855	55.0542	100.1120	98.0959		
1-Octylpyrrolidin-2-one	$C_{12}H_{23}NO$	-0.53	197.1779	198.1851	18.13	1.70E+08	[M+H]+	95.0	96.8
				86.0600	57.0699	71.0855	69.0335		
Carisoprodol	$C_{12}H_{24}N_2O_4$	-0.38	260.1735	261.1808	14.19	1.51E+08	[M+H]+	89.8	94.8
				62.0236	55.0542	97.1011	176.1280		
Diethylene glycol dibutyl ether	$C_{12}H_{26}O_3$	-0.17	250.1780	251.1853	10.57	5.63E+08	[M+H]+	92.6	66.6
				59.0491	117.0909	57.0335	175.1329		

Table S18. Mass spectral features identified at confidence level 2 as probable structures (continued)

Compound Name	Molecular Formula	ΔMass [ppm]	Exact Mass	*m/z*	RT [min]	Max Area	Adduct	*mzCloud* Best Match	*mzVault* Best Match
				Fragment Ion #1	Fragment Ion #2	Fragment Ion #3	Fragment Ion #4		
Tetrapropylene glycol	$C_{12}H_{26}O_5$	-0.66	282.1677	283.1750	6.00	5.57E+08	[M+H]+	96.4	78.7
				89.0596	133.0858	177.1117	195.1220		
Dodecylamine	$C_{12}H_{27}N$	-0.23	185.2143	186.2216	14.91	2.23E+08	[M+H]+	94.2	90.9
				57.0698	71.0855	85.1011	-		
N,N-Dimethyldecylamine *N*-oxide	$C_{12}H_{27}NO$	-0.28	201.2092	202.2165	15.41	3.65E+08	[M+H]+	98.0	98.9
				58.0651	62.0600	184.2056	71.0855		
Triisobutyl phosphate	$C_{12}H_{27}O_4P$	-0.40	266.1646	267.1719	19.55	2.66E+08	[M+H]+	92.4	96.7
				98.9840	155.0465	211.1093	57.0697		
N,N'-Dicyclohexylurea (1,3-Dicyclohexylurea)	$C_{13}H_{24}N_2O$	-0.43	224.1888	225.1960	15.57	2.17E+08	[M+H]+	97.4	81.2
				100.1120	83.0855	143.1178	-		
N-Methyldodecylamine	$C_{13}H_{29}N$	-0.67	199.2299	200.2371	17.69	8.46E+08	[M+H]+	-	90.5
				57.0698	71.0855	85.1012	55.0544		
Iminostilbene	$C_{14}H_{11}N$	-0.34	193.0891	194.0964	10.89	2.36E+07	[M+H]+	91.0	59.2
				179.0728	177.0704	193.0886	-		
Pindone	$C_{14}H_{14}O_3$	-0.28	230.0942	231.1015	15.48	4.64E+07	[M+H]+	89.0	97.7
				185.0960	170.0725	154.0773	158.0723		
Dibenzylamine	$C_{14}H_{15}N$	-0.20	197.1204	198.1277	9.19	1.96E+08	[M+H]+	97.6	99.5
				91.0541	181.1013	106.0652	65.0388		
Aspartame	$C_{14}H_{18}N_2O_5$	-0.65	294.1214	295.1287	5.06	4.80E+08	[M+H]+	70.4	71.1
				120.0806	103.0541	93.0696	91.0540		
N,N-Dimethylpentylone (Dipentylone; **Figure S8**)	$C_{14}H_{19}NO_3$	-0.85	249.1363	250.1438	9.37	8.57E+08	[M+H]+	81.6	41.1
				100.1120	135.0441	149.0235	175.0749		
N-Valylphenylalanine	$C_{14}H_{20}N_2O_3$	-0.28	264.1473	265.1546	7.88	6.25E+08	[M+H]+	89.5	47.3
				72.0807	120.0807	166.0861	55.0542		
Cashmeran	$C_{14}H_{22}O$	-0.52	206.1670	207.1742	17.62	1.94E+08	[M+H]+	-	81.4
				189.1636	95.0854	81.0698	67.0542		
(*R*)-3-Hydroxytetradecanoic acid	$C_{14}H_{28}O_3$	-0.26	244.2038	245.2110	19.60	4.25E+06	[M+H]+	95.4	33.0
				69.0698	191.1793	55.0542	57.0698		
N-Lauroylethanolamine	$C_{14}H_{29}NO_2$	-0.40	243.2197	244.2270	19.92	1.62E+08	[M+H]+	-	94.3
				62.0600	57.0698	227.2004	71.0855		
Heptaethylene glycol	$C_{14}H_{30}O_8$	-0.68	326.1939	327.2011	6.65	2.63E+09	[M+H]+	95.3	-
				89.0595	133.0858	177.1120	87.0437		
Tetradecylamine	$C_{14}H_{31}N$	-0.57	213.2455	214.2528	17.51	1.77E+09	[M+H]+	94.6	76.6
				57.0699	71.0855	85.1011	-		
Lauramine oxide	$C_{14}H_{31}NO$	-0.93	229.2404	230.2476	17.80	2.33E+10	[M+H]+	-	96.3
				58.0651	62.0600	212.2374	71.0855		
N,N,N',N'-Tetrakis(2-hydroxypropyl)ethylenediamine	$C_{14}H_{32}N_2O_4$	-0.41	292.2361	293.2434	2.24	1.01E+08	[M+H]+	95.9	-
				160.1330	84.0807	142.1225	102.0912		
Clopidogrel carboxylic acid	$C_{15}H_{14}ClNO_2S$	0.41	307.0435	308.0508	10.05	6.09E+07	[M+H]+	94.8	95.9
				198.0316	152.0261	141.0101	125.0152		

Table S18. Mass spectral features identified at confidence level 2 as probable structures (continued)

Compound Name	Molecular Formula	ΔMass [ppm]	Exact Mass	*m/z*	RT [min]	Max Area	Adduct	*mzCloud* Best Match	*mzVault* Best Match
				Fragment Ion #1	Fragment Ion #2	Fragment Ion #3	Fragment Ion #4		
3,5-Di-*tert*-butyl-4-hydroxybenzaldehyde	$C_{15}H_{22}O_2$	-0.41	234.1619	235.1692	18.72	4.48E+08	[M+H]+	91.5	93.5
				57.0698	179.1066	163.0758	-		
N,O-Didesmethylvenlafaxine	$C_{15}H_{23}NO_2$	0.07	249.1729	250.1802	8.80	1.22E+08	[M+H]+	82.5	95.4
				232.1697	107.0490	133.0648	201.1273		
Lupanine	$C_{15}H_{24}N_2O$	-0.15	248.1888	249.1961	4.02	2.86E+08	[M+H]+	-	95.8
				136.1119	114.0912	247.1815	150.1274		
Pentapropylene glycol	$C_{15}H_{32}O_6$	-0.30	308.2198	309.2271	12.19	6.94E+08	[M+H]+	95.0	-
				59.0491	117.0909	175.1326	57.0335		
Dodecyltrimethylammonium	$C_{15}H_{34}N^+$	-0.38	228.2686	228.2685	17.27	1.36E+08	[M]+	98.6	-
				60.0808	57.0699	71.0855	85.1013		
Ketoprofen	$C_{16}H_{14}O_3$	-0.04	254.0943	255.1016	15.05	4.77E+07	[M+H]+	75.9	75.1
				105.0334	209.0960	77.0385	177.0537		
O-Desmethylpyrilamine	$C_{16}H_{21}N_3O$	-0.49	271.1683	272.1756	6.36	1.14E+06	[M+H]+	56.0	62.5
				121.0761	227.1184	107.0494	72.0806		
Isocyclemone E	$C_{16}H_{26}O$	-0.29	234.1983	235.2056	20.60	7.36E+08	[M+H]+	-	82.5
				217.1950	95.0854	81.0698	133.1009		
5-Cyclohexadecen-1-one	$C_{16}H_{28}O$	-0.33	236.2139	237.2212	20.58	3.96E+08	[M+H]+	-	90.8
				69.0698	55.0542	219.2107	81.0698		
Palmitoleic acid	$C_{16}H_{30}O_2$	-0.19	254.2245	255.2318	20.49	4.04E+08	[M+H]+	94.7	-
				69.0698	55.0542	83.0855	219.2107		
Hexadecanamide	$C_{16}H_{33}NO$	-0.10	255.2562	256.2635	23.21	1.01E+08	[M+H]+	94.8	56.2
				88.0756	57.0698	102.0912	74.0600		
Lauryl diethanolamide	$C_{16}H_{33}NO_3$	-0.25	287.2460	288.2533	19.72	5.68E+08	[M+H]+	-	98.4
				106.0861	88.0756	70.0651	227.2005		
Bis(2-ethylhexyl)amine	$C_{16}H_{35}N$	-0.36	241.2769	242.2841	15.60	8.72E+07	[M+H]+	88.8	56.9
				130.1590	57.0699	71.0856	-		
Fenofibric acid	$C_{17}H_{15}ClO_4$	-0.41	318.0658	319.0730	17.71	7.14E+07	[M+H]+	94.0	93.3
				233.0362	138.9943	121.0284	-		
Riboflavin	$C_{17}H_{20}N_4O_6$	-0.02	376.1383	377.1456	8.31	2.53E+08	[M+H]+	80.1	95.2
				243.0877	172.0869	198.0662	69.0335		
Gingerol	$C_{17}H_{26}O_4$	-0.06	294.1831	295.1901	15.49	7.18E+06	[M+H]+	93.9	71.1
				177.0909	145.0647	137.0596	117.0698		
N-[3-(Dimethylamino)propyl]dodecanamide	$C_{17}H_{36}N_2O$	-0.49	284.2826	285.2899	17.71	8.66E+08	[M+H]+	-	98.4
				240.2320	57.0698	71.0854	95.0855		
Sulfasalazine	$C_{18}H_{14}N_4O_5S$	-0.12	398.0684	399.0757	13.89	1.71E+08	[M+H]+	84.6	84.9
				381.0649	119.0127	94.0524	223.0499		
Oxaprozin	$C_{18}H_{15}NO_3$	-0.33	293.1051	294.1124	17.38	1.68E+08	[M+H]+	93.1	92.1
				103.0541	276.1019	234.0913	206.0963		
8-[3-Oxo-2-[(*E*)-pent-2-enyl]cyclopenten-1-yl]octanoic acid	$C_{18}H_{28}O_3$	-0.59	292.2037	293.2110	18.67	2.45E+09	[M+H]+	93.9	76.1
				275.2005	81.0698	93.0698	95.0856		

Table S18. Mass spectral features identified at confidence level 2 as probable structures (continued)

Compound Name	Molecular Formula	ΔMass [ppm]	Exact Mass	*m/z*	RT [min]	Max Area	Adduct	*mzCloud* Best Match	*mzVault* Best Match
				Fragment Ion #1	Fragment Ion #2	Fragment Ion #3	Fragment Ion #4		
9*S*,13*R*-12-Oxophytodienoic acid	$C_{18}H_{28}O_3$	-0.25	292.2038	293.2111	18.95	2.28E+09	[M+H]+	93.2	72.9
				275.2006	81.0698	69.0699	105.0697		
α-Eleostearic acid	$C_{18}H_{30}O_2$	-0.79	278.2244	279.2316	20.90	1.07E+09	[M+H]+	93.8	82.2
				67.0542	81.0698	95.0854	109.1010		
Linolenic Acid	$C_{18}H_{30}O_2$	-0.57	278.2244	279.2317	20.73	1.01E+09	[M+H]+	95.8	82.8
				67.0542	81.0698	95.0854	109.1011		
13(*S*)-HOTrE	$C_{18}H_{30}O_3$	-0.54	294.2193	295.2266	18.46	1.06E+09	[M+H]+	89.7	64.9
				277.2161	67.0542	81.0698	71.0855		
9-Oxo-10,12-octadecadienoic acid	$C_{18}H_{30}O_3$	-0.62	294.2193	295.2266	20.73	5.71E+08	[M+H]+	93.4	69.7
				277.2161	67.0542	69.0698	151.1117		
Oleamide	$C_{18}H_{35}NO$	-0.24	281.2718	282.2791	23.47	5.64E+08	[M+H]+	94.9	43.2
				69.0698	83.0854	57.0698	55.0542		
Stearamide	$C_{18}H_{37}NO$	-0.30	283.2874	284.2947	24.29	1.42E+08	[M+H]+	93.1	-
				88.0756	57.0699	102.0912	74.0600		
Palmitoylethanolamide	$C_{18}H_{37}NO_2$	-0.32	299.2823	300.2896	23.07	2.89E+08	[M+H]+	94.3	56.1
				62.0600	57.0698	283.2632	71.0854		
Hexapropylene glycol	$C_{18}H_{38}O_7$	-0.22	366.2617	389.2509	13.86	1.39E+09	[M+Na]+	90.5	-
				59.0491	117.0908	175.1327	57.0335		
Trazodone	$C_{19}H_{22}ClN_5O$	0.03	371.1513	372.1586	10.30	7.76E+07	[M+H]+	91.2	89.7
				176.0817	148.0505	78.0339	96.0443		
Androsta-1,4-diene-3,17-dione	$C_{19}H_{24}O_2$	-0.20	284.1776	285.1849	14.46	8.79E+08	[M+H]+	96.3	84.2
				121.0648	151.1118	147.1168	267.1744		
Adrenosterone	$C_{19}H_{24}O_3$	-0.27	300.1725	301.1797	12.88	6.91E+07	[M+H]+	68.7	88.2
				121.0647	257.1534	109.0647	283.1688		
Boldenone	$C_{19}H_{26}O_2$	-0.32	286.1932	287.2005	15.56	5.62E+07	[M+H]+	95.2	93.3
				121.0647	135.1168	173.0960	93.0698		
11-Ketotestosterone	$C_{19}H_{26}O_3$	-0.07	302.1882	303.1955	13.52	1.71E+08	[M+H]+	91.9	88.5
				121.0647	267.1745	259.1694	105.0699		
Methyl alpha-eleostearate	$C_{19}H_{32}O_2$	-0.09	292.2402	293.2475	24.05	2.23E+08	[M+H]+	94.5	68.1
				81.0698	67.0542	95.0854	109.1010		
cis-12-Octadecenoic acid methyl ester	$C_{19}H_{36}O_2$	-0.56	296.2714	297.2786	22.96	1.76E+08	[M+H]+	91.1	-
				69.0698	247.2419	265.2524	83.0854		
Deacetyldiltiazem	$C_{20}H_{24}N_2O_3S$	0.01	372.1508	373.1580	11.07	1.17E+08	[M+H]+	91.7	79.6
				178.0319	150.0370	72.0807	109.0105		
1,4*a*-Dimethyl-9-oxo-7-propan-2-yl-3,4,10,10*a*-tetrahydro-2H-phenanthrene-1-carboxylic acid	$C_{20}H_{26}O_3$	-0.93	314.1879	315.1952	19.85	1.46E+08	[M+H]+	94.3	88.1
				187.1116	171.0803	213.1272	199.1117		
Abietic acid	$C_{20}H_{30}O_2$	-0.24	302.2245	303.2318	19.56	2.52E+08	[M+H]+	95.0	94.0
				123.1167	257.2264	149.1324	121.1011		
(+/-)12-HpETE	$C_{20}H_{32}O_4$	-0.33	336.2300	319.2266	17.70	2.82E+08	[M+H-H2O]+	79.6	65.0
				273.2211	255.2106	301.2161	81.0698		

**Table S18.** Mass spectral features identified at confidence level 2 as probable structures (continued)									
Compound Name	Molecular Formula	ΔMass [ppm]	Exact Mass	*m/z* / Fragment Ion #1	RT [min] / Fragment Ion #2	Max Area / Fragment Ion #3	Adduct / Fragment Ion #4	*mzCloud* Best Match	*mzVault* Best Match
Dihomo-γ-linolenic acid	$C_{20}H_{34}O_2$	-0.11	306.2559	307.2631	22.36	2.27E+08	[M+H]+	94.8	89.7
				67.0542	81.0698	95.0854	109.1011		
Linoleoyl ethanolamide	$C_{20}H_{37}NO_2$	-0.39	323.2823	324.2896	22.61	7.93E+08	[M+H]+	91.1	-
				62.0600	67.0541	81.0698	95.0854		
Oleylethanolamide	$C_{20}H_{39}NO_2$	-0.47	325.2979	326.3052	23.36	7.12E+08	[M+H]+	94.4	82.5
				62.0600	69.0698	55.0542	83.0855		
Tetraethylene glycol monododecyl ether	$C_{20}H_{42}O_5$	0.21	362.3033	363.3106	23.95	1.41E+09	[M+H]+	-	90.4
				89.0596	57.0698	133.0857	195.1225		
Quetiapine	$C_{21}H_{25}N_3O_2S$	-0.02	383.1667	384.1740	11.81	2.05E+08	[M+H]+	90.5	87.6
				253.0793	221.1072	279.0950	210.0370		
Heptapropylene glycol	$C_{21}H_{44}O_8$	-0.31	424.3035	425.3108	15.19	1.58E+09	[M+H]+	94.8	-
				59.0491	117.0909	175.1327	57.0335		
Azoxystrobin	$C_{22}H_{17}N_3O_5$	-0.27	403.1167	404.1240	15.31	1.17E+08	[M+H]+	92.3	94.1
				372.0978	344.1030	329.0795	172.0391		
Rosuvastatin	$C_{22}H_{28}FN_3O_6S$	-0.24	481.1682	482.1754	14.43	1.16E+08	[M+H]+	91.3	88.0
				258.1398	300.1505	270.1397	272.1553		
Erucamide	$C_{22}H_{43}NO$	-0.15	337.3344	338.3417	25.66	3.62E+08	[M+H]+	91.3	92.3
				69.0698	57.0698	83.0855	321.3152		
Didecyldimethylammonium	$C_{22}H_{48}N^+$	-0.65	326.3781	326.3779	20.49	3.52E+08	[M]+	96.8	-
				186.2215	57.0698	71.0855	184.2057		
Diisooctyl phthalate	$C_{24}H_{38}O_4$	-0.30	390.2769	391.2842	24.34	1.02E+08	[M+H]+	81.1	84.3
				149.0230	71.0854	57.0698	167.0336		
Chenodiol	$C_{24}H_{40}O_4$	-0.45	392.2925	415.2817	20.84	1.52E+08	[M+Na]+	93.8	-
				391.2859	409.2969	-	-		
Octapropylene glycol	$C_{24}H_{50}O_9$	-0.05	482.3455	483.3527	16.41	1.52E+09	[M+H]+	91.5	-
				59.0491	117.0908	175.1327	57.0334		
Bis(3,5,5-trimethylhexyl) phthalate	$C_{26}H_{42}O_4$	-0.24	418.3082	419.3155	25.35	2.79E+08	[M+H]+	87.5	93.2
				149.0232	71.0854	57.0698	85.1011		
Glycochenodeoxycholic acid	$C_{26}H_{43}NO_5$	-0.15	449.3141	450.3214	19.64	1.59E+08	[M+H]+	-	88.5
				414.3003	76.0392	339.2684	107.0854		
Glycocholic acid	$C_{26}H_{43}NO_6$	-0.50	465.3088	466.3161	17.79	4.48E+08	[M+H]+	78.5	91.8
				412.2848	337.2528	430.2954	76.0393		
Cholesta-4,6-dien-3-one	$C_{27}H_{42}O$	-0.37	382.3234	383.3307	28.84	9.93E+07	[M+H]+	-	83.9
				95.0853	81.0699	247.2414	93.0698		
Cholest-4-en-3-one	$C_{27}H_{44}O$	-0.10	384.3392	385.3464	29.07	9.89E+08	[M+H]+	89.7	88.2
				109.0647	97.0647	81.0698	123.0803		
N-Nitrosodiethylamine	$C_4H_{10}N_2O$	-0.93	102.0792	103.0865	7.20	2.36E+08	[M+H]+	92.1	82.1
				75.0552	-	-	-		
Diethanolamine	$C_4H_{11}NO_2$	-1.05	105.0789	106.0861	13.76	1.71E+08	[M+H]+	92.3	91.4
				88.0757	70.0651	62.0600	-		

Table S18. Mass spectral features identified at confidence level 2 as probable structures (continued)

Compound Name	Molecular Formula	ΔMass [ppm]	Exact Mass	*m/z*	RT [min]	Max Area	Adduct	*mzCloud* Best Match	*mzVault* Best Match
				Fragment Ion #1	Fragment Ion #2	Fragment Ion #3	Fragment Ion #4		
Diethyl phosphate	$C_4H_{11}O_4P$	-0.13	154.0395	155.0468	13.38	8.56E+07	[M+H]+	84.1	-
				98.9841	80.9735	-	-		
Uracil	$C_4H_4N_2O_2$	-0.99	112.0272	113.0344	2.18	1.67E+08	[M+H]+	94.4	95.4
				70.0287	96.0079	68.0131	95.0239		
Cytosine	$C_4H_5N_3O$	-1.03	111.0432	112.0504	2.08	7.23E+07	[M+H]+	96.9	96.6
				95.0239	69.0447	67.0288	94.0399		
Creatinine	$C_4H_7N_3O$	-1.08	113.0588	114.0661	1.98	6.92E+08	[M+H]+	98.7	92.4
				86.0712	72.0443	-	-		
4-Aminobutanoic acid	$C_4H_9NO_2$	-1.20	103.0632	104.0705	1.96	5.10E+07	[M+H]+	53.0	87.6
				87.0439	60.0807	69.0334	-		
Creatine	$C_4H_9N_3O_2$	-0.82	131.0694	132.0767	2.05	1.06E+08	[M+H]+	88.0	88.1
				90.0549	87.0550	114.0659	115.0501		
Glutamine	$C_5H_{10}N_2O_3$	-0.42	146.0691	147.0764	1.75	1.06E+07	[M+H]+	41.9	76.2
				56.0495	84.0443	130.0497			
Valine	$C_5H_{11}NO_2$	-1.07	117.0789	118.0861	2.14	6.70E+08	[M+H]+	77.4	91.1
				72.0807	55.0542	100.1120	58.0651		
Methionine	$C_5H_{11}NO_2S$	-0.62	149.0510	150.0582	2.31	6.11E+08	[M+H]+	97.5	96.2
				104.0528	56.0495	133.0317	61.0106		
4-Guanidinobutyric acid	$C_5H_{11}N_3O_2$	-0.45	145.0851	146.0923	2.10	7.71E+07	[M+H]+	88.3	90.2
				87.0440	60.0556	104.0705	128.0819		
Choline	$C_5H_{14}NO^+$	-1.54	104.1070	104.1068	1.85	9.15E+07	[M]+	93.0	91.9
				60.0807	58.0650	-	-		
Agmatine	$C_5H_{14}N_4$	-0.35	130.1218	131.1291	1.72	1.84E+06	[M+H]+	56.4	89.0
				84.9596	72.0807	60.0556	114.1023		
Hypoxanthine	$C_5H_4N_4O$	-0.88	136.0384	137.0457	2.30	1.55E+09	[M+H]+	97.3	96.1
				110.0349	119.0352	94.0400	82.0400		
Oxypurinol	$C_5H_4N_4O_2$	-0.11	152.0334	153.0407	3.70	1.07E+09	[M+H]+	89.0	95.3
				110.0348	136.0141	135.0302	-		
Uric acid	$C_5H_4N_4O_3$	-0.55	168.0283	169.0355	2.17	8.16E+07	[M+H]+	93.4	96.7
				141.0406	152.0090	126.0297	70.0399		
Adenine	$C_5H_5N_5$	-0.73	135.0544	136.0617	2.09	1.55E+08	[M+H]+	97.2	93.7
				119.0352	94.0400	-	-		
Guanine	$C_5H_5N_5O$	-0.71	151.0493	152.0566	3.13	3.14E+09	[M+H]+	97.8	97.7
				135.0301	110.0348	128.0454	107.0356		
Thymine	$C_5H_6N_2O_2$	-0.92	126.0428	127.0501	4.37	1.52E+09	[M+H]+	95.5	95.6
				110.0235	84.0444	56.0495	82.0287		
Pyroglutamic acid	$C_5H_7NO_3$	-0.55	129.0425	130.0498	2.42	2.54E+08	[M+H]+	92.4	94.4
				84.0442	56.0494	-	-		
Proline	$C_5H_9NO_2$	-0.98	115.0632	116.0705	2.05	4.04E+08	[M+H]+	99.4	96.2
				70.0651	68.0495	98.0598	-		

**Table S18.** Mass spectral features identified at confidence level 2 as probable structures (continued)									
Compound Name	Molecular Formula	ΔMass [ppm]	Exact Mass	*m/z*	RT [min]	Max Area	Adduct	*mzCloud* Best Match	*mzVault* Best Match
				Fragment Ion #1	Fragment Ion #2	Fragment Ion #3	Fragment Ion #4		
Glutamic acid	$C_5H_9NO_4$	-0.49	147.0531	148.0604	1.88	3.26E+08	[M+H]+	95.2	97.2
				84.0443	102.0548	130.0497	56.0494		
Adipic acid	$C_6H_{10}O_4$	-0.31	146.0579	147.0651	19.35	1.27E+08	[M+H]+	87.2	60.0
				101.0596	55.0542	111.0439	129.0546		
Pipecolic acid	$C_6H_{11}NO_2$	-0.40	129.0789	130.0862	2.12	1.41E+08	[M+H]+	91.5	91.5
				84.0807	56.0494	-	-		
Norleucine	$C_6H_{13}NO_2$	-0.89	131.0945	132.1018	3.17	5.90E+09	[M+H]+	97.7	95.7
				86.0963	69.0698	-	-		
Isoleucine	$C_6H_{13}NO_2$	-0.90	131.0945	132.1018	2.97	1.55E+09	[M+H]+	95.8	96.1
				86.0963	69.0698	67.0543	-		
Leucine	$C_6H_{13}NO_2$	-0.91	131.0945	132.1018	3.16	3.57E+09	[M+H]+	97.5	92.9
				86.0963	69.0698	87.0996	-		
Citrulline	$C_6H_{13}N_3O_3$	-0.90	175.0955	176.1028	1.91	8.72E+07	[M+H]+	92.8	95.0
				159.0764	70.0651	113.0709	115.0865		
Lysine	$C_6H_{14}N_2O_2$	-0.10	146.1055	147.1128	1.65	3.59E+07	[M+H]+	83.8	85.5
				84.0807	130.0861	129.1022	-		
Arginine	$C_6H_{14}N_4O_2$	-0.67	174.1116	175.1188	1.77	1.35E+08	[M+H]+	94.5	94.4
				70.0651	60.0556	116.0705	130.0973		
N,N-Diethylethanolamine	$C_6H_{15}NO$	-1.28	117.1152	118.1225	2.10	8.83E+07	[M+H]+	91.9	94.7
				100.1120	72.0807	74.0964	58.0649		
Triethanolamine	$C_6H_{15}NO_3$	-0.81	149.1051	150.1124	1.78	1.01E+08	[M+H]+	98.1	89.2
				132.1018	70.0651	88.0756	114.0912		
Triethyl phosphate	$C_6H_{15}O_4P$	-0.45	182.0707	183.0780	11.00	2.02E+08	[M+H]+	91.4	96.8
				98.9840	127.0153	155.0466	80.9737		
Nicotinic acid	$C_6H_5NO_2$	-0.59	123.0320	124.0392	2.27	7.45E+07	[M+H]+	90.7	93.7
				80.0494	78.0338	96.0443	122.0235		
Urocanic acid	$C_6H_6N_2O_2$	-0.30	138.0429	139.0502	2.12	1.63E+08	[M+H]+	97.3	97.4
				121.0395	93.0447	95.0603	68.0494		
3-Methylxanthine	$C_6H_6N_4O_2$	-0.89	166.0489	167.0562	4.47	1.14E+09	[M+H]+	89.3	92.0
				124.0504	96.0555	149.0453	69.0447		
1-Methyluric acid	$C_6H_6N_4O_3$	-0.44	182.0439	183.0512	3.89	1.34E+08	[M+H]+	94.5	93.7
				155.0564	126.0298	152.0089	70.0398		
Pyrogallol	$C_6H_6O_3$	-0.42	126.0316	127.0389	7.56	3.36E+10	[M+H]+	91.4	92.3
				109.0282	81.0334	53.0385	-		
5-Hydroxymethylfurfural	$C_6H_6O_3$	-1.34	126.0315	127.0388	5.10	7.17E+10	[M+H]+	89.5	90.0
				109.0282	81.0334	53.0385	69.0334		
7-Methylguanine	$C_6H_7N_5O$	-0.54	165.0650	166.0723	2.23	7.00E+07	[M+H]+	90.7	83.9
				149.0458	124.0505	107.0238	-		
4-Methyl-5-thiazoleethanol	C_6H_9NOS	-0.14	143.0405	144.0477	4.10	4.63E+08	[M+H]+	97.2	93.3
				113.0292	126.0371	99.0262	65.0385		

Table S18. Mass spectral features identified at confidence level 2 as probable structures (continued)									
Compound Name	Molecular Formula	ΔMass [ppm]	Exact Mass	*m/z* / Fragment Ion #1	RT [min] / Fragment Ion #2	Max Area / Fragment Ion #3	Adduct / Fragment Ion #4	*mzCloud* Best Match	*mzVault* Best Match
Histidine	$C_6H_9N_3O_2$	-0.95	155.0693	156.0766	1.75	7.22E+06	[M+H]+	91.3	96.1
				110.0711	83.0602	93.0446	95.0603		
Glycylproline	$C_7H_{12}N_2O_3$	-0.70	172.0847	173.0920	2.11	8.76E+07	[M+H]+	90.0	83.1
				116.0705	70.0651	127.0865	-		
Stachydrine	$C_7H_{13}NO_2$	-0.38	143.0946	144.1019	2.06	1.06E+08	[M+H]+	93.4	78.8
				98.0963	58.0651	84.0807	102.0549		
5-Chloro-6-methyl-2H-benzotriazole	$C_7H_6ClN_3$	-0.35	167.0250	168.0322	13.23	1.19E+08	[M+H]+	95.2	-
				113.0152	140.0261	77.0386	95.0490		
Anthranilic acid	$C_7H_7NO_2$	-0.98	137.0475	138.0549	8.58	9.50E+08	[M+H]+	95.6	95.5
				120.0442	92.0494	65.0385	110.0599		
Trigonelline	$C_7H_7NO_2$	-0.62	137.0476	138.0549	2.05	7.73E+07	[M+H]+	92.7	95.3
				94.0651	92.0494	110.0599	78.0340		
N1-Methyl-4-pyridone-5-carboxamide	$C_7H_8N_2O_2$	-0.90	152.0584	153.0657	3.04	1.94E+08	[M+H]+	62.2	82.7
				136.0391	108.0442	65.0384	91.0541		
2-Phenethylamine (**Figure S10**)	$C_8H_{11}N$	-0.64	121.0891	122.0963	5.34	8.47E+08	[M+H]+	97.5	96.0
				105.0697	79.0542	51.0231	77.0384		
Glycylleucine	$C_8H_{16}N_2O_3$	-0.47	188.1160	189.1233	5.83	1.30E+09	[M+H]+	95.9	88.6
				86.0964	132.1019	143.1179	171.1125		
4-Aminovaleric acid betaine	$C_8H_{17}NO_2$	-0.54	159.1258	160.1331	2.09	4.46E+07	[M+H]+	88.3	91.2
				101.0596	60.0808	55.0542	83.0490		
Heptaminol	$C_8H_{19}NO$	-0.25	145.1466	146.1539	7.79	1.29E+09	[M+H]+	92.2	-
				128.1433	69.0698	55.0542	72.0808		
Octylphosphonic acid	$C_8H_{19}O_3P$	-0.41	194.1071	195.1144	15.90	8.49E+08	[M+H]+	94.9	-
				125.0361	69.0698	139.0517	57.0698		
Dibutyl phosphate	$C_8H_{19}PO_4$	-0.10	210.1021	211.1094	13.39	1.05E+08	[M+H]+	77.0	90.9
				98.9841	155.0468	57.0699	80.9735		
Oxindole	C_8H_7NO	0.11	133.0528	134.0601	9.20	9.40E+08	[M+H]+	92.8	99.0
				106.0651	79.0542	116.0497	-		
2-(Methylthio)benzothiazole	$C_8H_7NS_2$	-0.86	181.0018	182.0091	15.65	3.10E+08	[M+H]+	94.9	96.4
				166.9857	135.0138	109.0106	-		
Indoline	C_8H_9N	-0.92	119.0734	120.0807	4.74	4.74E+09	[M+H]+	-	86.7
				103.0542	93.0700	91.0541	118.0649		
N-Benzylformamide	C_8H_9NO	-0.64	135.0683	136.0756	7.84	1.08E+09	[M+H]+	80.7	94.3
				91.0541	65.0391	-	-		
4-Pyridoxic acid	$C_8H_9NO_4$	-0.39	183.0531	184.0604	3.48	2.86E+08	[M+H]+	88.3	92.0
				166.0498	148.0393	138.0553	-		
5,6-Dimethyl-1H-benzotriazole	$C_8H_9N_3$	-0.05	147.0796	148.0869	11.78	7.16E+07	[M+H]+	-	89.4
				93.0697	91.0541	120.0806	-		
Phenylalanine	$C_9H_{11}NO_2$	-1.27	165.0788	166.0860	5.03	7.64E+09	[M+H]+	97.6	98.1
				120.0807	103.0541	131.0490	107.0492		

**Table S18.** Mass spectral features identified at confidence level 2 as probable structures (continued)									
Compound Name	Molecular Formula	ΔMass [ppm]	Exact Mass	*m/z*	RT [min]	Max Area	Adduct	*mzCloud* Best Match	*mzVault* Best Match
				Fragment Ion #1	Fragment Ion #2	Fragment Ion #3	Fragment Ion #4		
Tyrosine	$C_9H_{11}NO_3$	-0.86	181.0737	182.0810	2.61	5.27E+08	[M+H]+	97.7	97.2
				136.0756	165.0546	123.0440	119.0491		
N-Ethyl-*p*-toluenesulfonamide	$C_9H_{13}NO_2S$	-0.26	199.0667	200.0739	11.78	1.02E+08	[M+H]+	87.8	94.1
				91.0541	119.0602	155.0160	109.0647		
Isophorone	$C_9H_{14}O$	-0.33	138.1044	139.1117	12.27	8.83E+07	[M+H]+	91.8	89.5
				69.0334	97.0646	121.1011	83.0490		
Acetylcarnitine	$C_9H_{17}NO_4$	-0.67	203.1156	204.1229	2.10	2.29E+07	[M+H]+	94.4	96.9
				85.0283	60.0807	145.0496	57.0335		
Pantothenic acid	$C_9H_{17}NO_5$	-0.49	219.1106	220.1178	5.27	4.50E+08	[M+H]+	82.3	95.8
				90.0548	202.1073	184.0968	98.0236		
2,2,6,6-Tetramethyl-4-piperidinol	$C_9H_{19}NO$	-0.72	157.1466	158.1538	2.28	1.36E+08	[M+H]+	93.6	42.5
				58.0651	85.0647	123.1167	102.0913		
N,N'-Dibutylurea	$C_9H_{20}N_2O$	-0.55	172.1575	173.1648	12.91	8.13E+07	[M+H]+	-	80.1
				74.0964	57.0699	41.0386	-		
2-(2-(2-Propoxyethoxy)ethoxy)ethanol	$C_9H_{20}O_4$	-0.64	192.1360	193.1433	9.51	1.54E+08	[M+H]+	-	85.3
				89.0596	45.0335	133.0860	69.0698		
N-Methyloctylamine	$C_9H_{21}N$	-0.19	143.1674	144.1747	11.80	7.82E+07	[M+H]+	82.7	93.5
				57.0698	71.0855	43.0542	41.0386		
Indole-4-carboxaldehyde	C_9H_7NO	-0.11	145.0528	146.0600	9.96	6.44E+08	[M+H]+	97.6	99.3
				118.0650	91.0541	117.0572	-		
1,5-Isoquinolinediol	$C_9H_7NO_2$	0.06	161.0477	162.0550	9.93	7.02E+07	[M+H]+	85.1	97.3
				116.0494	144.0443	91.0542	134.0601		
N-Acetyl mesalazine	$C_9H_9NO_4$	-0.05	195.0532	196.0604	7.35	1.75E+08	[M+H]+	89.5	94.4
				136.0391	178.0498	108.0442	80.0495		
Ethyl 4-dimethylaminobenzoate	$C_{11}H_{15}NO_2$	-0.37	193.1102	194.1175	16.08	4.38E+07	[M+H]+	71.6	90.3
				151.0625	166.0860	179.0938	134.0598		
Butyl 4-aminobenzoate	$C_{11}H_{15}NO_2$	-0.30	193.1102	194.1175	14.68	4.43E+07	[M+H]+	89.8	-
				138.0548	94.0651	57.0699	120.0443		
Progesterone	$C_{21}H_{30}O_2$	-0.40	314.2245	315.2317	18.58	1.78E+07	[M+H]+	77.3	74.3
				97.0647	109.0647	297.2199	79.0542		
Theobromine	$C_7H_8N_4O_2$	-0.74	180.0646	181.0719	5.14	1.63E+09	[M+H]+	97.9	94.6
				138.0661	110.0712	108.0555	163.0614		

Table S19. Mass spectral features assigned at confidence level 3 as tentative candidates

Compound Name	Molecular Formula	ΔMass [ppm]	Exact Mass	*m/z*	RT [min]	Max Area	Adduct	*mzCloud* Best Match	*mzVault* Best Match
				Fragment Ion #1	Fragment Ion #2	Fragment Ion #3	Fragment Ion #4		
Indole-3-acetamide	$C_{10}H_{10}N_2O$	-0.66	174.0792	175.0865	6.04	5.18E+08	[M+H]+	74.4	69.0
				130.0650	157.0759	158.0599	-		
2-Methoxycinnamaldehyde	$C_{10}H_{10}O_2$	-0.59	162.0680	163.0753	19.01	1.54E+08	[M+H]+	-	93.4
				107.0854	93.0698	121.1011	81.0698		
Ferulic acid	$C_{10}H_{10}O_4$	-0.43	194.0578	195.0651	9.57	1.15E+08	[M+H]+	72.5	83.7
				177.0545	145.0283	117.0334	149.0596		
4-Isopropylbenzaldehyde	$C_{10}H_{12}O$	-0.56	148.0887	149.0960	12.26	3.33E+08	[M+H]+	84.1	61.2
				79.0542	121.1011	131.0855	91.0542		
4-Phenylbutanoic acid	$C_{10}H_{12}O_2$	-0.72	164.0836	165.0909	7.34	3.17E+08	[M+H]+	88.0	83.8
				137.0959	67.0542	109.1011	119.0853		
3-(3-Methylbut-2-enyl)-7H-purin-6-imine	$C_{10}H_{13}N_5$	-0.23	203.1171	204.1243	7.38	7.47E+07	[M+H]+	84.9	82.2
				136.0617	69.0698	119.0351	-		
2’-Deoxyguanosine	$C_{10}H_{13}N_5O_4$	-0.70	267.0966	268.1038	3.30	5.55E+08	[M+H]+	95.9	93.2
				152.0566	135.0301	110.0348	117.0545		
Carvone	$C_{10}H_{14}O$	-0.38	150.1044	151.1117	11.91	2.48E+08	[M+H]+	88.8	90.3
				133.1012	105.0698	81.0698	91.0542		
9-Methoxy-9-oxononanoic acid	$C_{10}H_{18}O_4$	-0.19	202.1205	203.1278	11.57	2.63E+08	[M+H]+	89.3	45.7
				55.0542	125.0961	97.1011	83.0855		
Decanamide	$C_{10}H_{21}NO$	-0.45	171.1622	172.1695	17.54	2.12E+07	[M+H]+	81.9	30.3
				88.0756	102.0913	74.0600	71.0854		
Triethylene glycol monobutyl ether	$C_{10}H_{22}O_4$	-0.98	206.1516	207.1589	11.49	8.10E+09	[M+H]+	92.7	-
				89.0596	151.0963	133.0859	101.0959		
4-Methylumbelliferone	$C_{10}H_8O_3$	-0.42	176.0473	177.0546	14.22	2.30E+08	[M+H]+	72.3	37.4
				149.0232	65.0386	111.0440	105.0694		
3-Oxoindane-1-carboxylic acid	$C_{10}H_8O_3$	-0.52	176.0473	177.0545	9.55	8.23E+07	[M+H]+	74.7	39.9
				91.0545	107.0490	103.0541	99.0440		
Scopoletin	$C_{10}H_8O_4$	-0.52	192.0422	193.0494	9.06	2.14E+07	[M+H]+	76.1	67.6
				133.0284	178.0256	137.0593	91.0541		
6-Methylquinoline	$C_{10}H_9N$	-0.45	143.0734	144.0807	6.45	3.01E+08	[M+H]+	91.7	86.8
				115.0540	91.0540	142.0657	117.0578		
5-Hydroxyindole-3-acetic acid	$C_{10}H_9NO_3$	-0.11	191.0582	192.0655	12.85	9.45E+07	[M+H]+	61.3	67.2
				146.0600	91.0541	117.0574	119.0490		
7-Deoxyechinosporin	$C_{10}H_9NO_4$	-0.39	207.0531	208.0604	7.40	1.13E+08	[M+H]+	75.6	-
				162.0548	190.0497	147.0440	119.0487		
1-(*p*-Tolyl)pyrrolidine-2,5-dione	$C_{11}H_{11}NO_2$	0.07	189.0790	190.0863	9.99	7.29E+07	[M+H]+	68.9	-
				162.0912	91.0541	95.0490	117.0696		
4,6-Dimethyl-2-oxo-5-prop-2-enyl-1H-pyridine-3-carbonitrile	$C_{11}H_{12}N_2O$	-0.02	188.0950	189.1022	9.93	1.49E+08	[M+H]+	78.9	81.9
				171.0915	161.0710	130.0651	91.0546		
5-Phenylvaleric acid	$C_{11}H_{14}O_2$	-0.41	178.0993	179.1066	12.91	8.85E+07	[M+H]+	51.7	70.4
				137.0952	133.0647	151.0759	105.0698		

Compound Name	Molecular Formula	ΔMass [ppm]	Exact Mass	*m/z*	RT [min]	Max Area	Adduct	*mzCloud* Best Match	*mzVault* Best Match
				Fragment Ion #1	Fragment Ion #2	Fragment Ion #3	Fragment Ion #4		
N-6-Methyl-2-deoxyadenosine	$C_{11}H_{15}N_5O_3$	-0.44	265.1174	266.1247	4.47	2.34E+08	[M+H]+	92.3	-
				150.0772	117.0543	73.0283	-		
1-Methylguanosine	$C_{11}H_{15}N_5O_5$	-0.59	297.1071	298.1144	4.33	1.56E+08	[M+H]+	77.5	82.8
				166.0723	149.0457	-	-		
2-(*N*-Ethyl-*m*-toluidino)ethanol	$C_{11}H_{17}NO$	-0.53	179.1309	180.1382	15.15	8.97E+07	[M+H]+	78.4	73.8
				107.0728	120.0806	147.1044	134.0962		
Cyclo(leucyloprolyl)	$C_{11}H_{18}N_2O_2$	-0.29	210.1368	211.1440	9.20	2.75E+08	[M+H]+	94.0	87.6
				70.0650	86.0963	183.1490	114.0911		
5-(2-Methylpropyl)-3,6-dioxo-2-piperazinepropanoic acid	$C_{11}H_{18}N_2O_4$	-0.25	242.1266	243.1339	5.90	5.85E+08	[M+H]+	87.2	41.1
				84.0443	86.0963	132.1017	197.1283		
Prolylleucine	$C_{19}H_{26}N_2O_5$	-0.35	228.1473	229.1546	5.54	3.61E+08	[M+H]+	91.4	-
				70.0650	183.1496	-	-		
δ-Undecalactone	$C_{11}H_{20}O_2$	-0.34	184.1463	185.1535	16.92	1.85E+08	[M+H]+	-	78.9
				149.1324	167.1430	83.0855	107.0854		
Lumichrome	$C_{12}H_{10}N_4O_2$	-0.19	242.0803	243.0876	11.22	4.80E+08	[M+H]+	80.9	86.4
				172.0868	216.0765	198.0662	170.0711		
N-(1,2,3,9-Tetrahydrocarbazol-4-ylidene)hydroxylamine	$C_{12}H_{12}N_2O$	-0.21	200.0949	201.1022	8.06	7.68E+07	[M+H]+	73.6	-
				184.0994	145.0759	143.0727	132.0808		
Dimethylone (bk-MDDMA; **Figure S12**)	$C_{12}H_{15}NO_3$	-0.41	221.1051	222.1124	5.56	9.99E+08	[M+H]+	78.0	-
				100.0757	72.0804	149.0230	121.0284		
Isoelemicin	$C_{12}H_{16}O_3$	-0.18	208.1099	209.1172	16.77	2.65E+07	[M+H]+	74.2	-
				117.0696	135.1167	177.1271	149.1323		
4-Methoxy-*N,N*-dimethylcathinone (4-Methoxy-*N,N*-DMC; **Figure S11**)	$C_{12}H_{17}NO_2$	-0.32	207.1259	208.1332	13.33	1.46E+08	[M+H]+	73.6	83.0
				135.0439	79.0542	107.0493	100.0759		
9-Methoxycarbonyldec-9-enoic acid	$C_{12}H_{20}O_4$	-0.33	228.1361	229.1433	14.16	1.18E+08	[M+H]+	70.5	-
				211.1329	81.0698	151.1117	133.1010		
4-Oxododecanedioic acid	$C_{12}H_{20}O_5$	0.12	244.1311	245.1384	10.93	2.92E+08	[M+H]+	78.0	-
				97.1011	125.0960	83.0854	171.1013		
γ-Dodecalactone	$C_{12}H_{22}O_2$	-0.72	198.1618	199.1691	19.16	3.48E+09	[M+H]+	49.5	76.8
				163.1480	83.0854	181.1587	97.1011		
Laurolactam	$C_{12}H_{23}NO$	-0.57	197.1779	198.1851	17.83	1.41E+08	[M+H]+	89.6	68.2
				72.0443	55.0542	69.0698	-		
12-Aminododecanoic acid	$C_{12}H_{25}NO_2$	-0.49	215.1884	216.1957	14.21	3.92E+07	[M+H]+	89.5	-
				163.1480	55.0542	83.0855	69.0699		
9-Aminoacridine	$C_{13}H_{10}N_2$	-0.69	194.0843	195.0915	11.68	1.78E+08	[M+H]+	73.6	-
				138.0661	163.0391	89.0597	135.1169		
Allenolic acid	$C_{13}H_{12}O_3$	-0.15	216.0786	217.0859	13.19	2.89E+07	[M+H]+	65.2	-
				171.0801	143.0854	115.0545	128.0620		
(4*E*)-4-[1-(dimethylamino)ethylidene]-2-phenyl-1,3-oxazol-5-one	$C_{13}H_{14}N_2O_2$	-0.31	230.1055	231.1127	7.99	7.50E+08	[M+H]+	67.5	-
				188.0706	158.0963	146.0600	130.0650		

Table title: **Table S19.** Mass spectral features assigned at confidence level 3 as tentative candidates (continued)

Table S19. Mass spectral features assigned at confidence level 3 as tentative candidates (continued)

Compound Name	Molecular Formula	ΔMass [ppm]	Exact Mass	*m/z* / Fragment Ion #1	RT [min] / Fragment Ion #2	Max Area / Fragment Ion #3	Adduct / Fragment Ion #4	*mzCloud* Best Match	*mzVault* Best Match
Phenylacetylglutamine	$C_{13}H_{16}N_2O_4$	0.14	264.1110	265.1183	7.45	3.73E+08	[M+H]+	94.7	97.4
				130.0498	84.0443	91.0542	136.0757		
2-Phenyl-2,3-dihydroquinazolin-4(1H)-one	$C_{14}H_{12}N_2O$	-0.23	224.0949	225.1022	13.05	2.24E+08	[M+H]+	65.6	-
				180.0806	120.0443	95.0487	208.0764		
Cyclo(phenylalanylprolyl)	$C_{14}H_{16}N_2O_2$	-0.25	244.1211	245.1284	9.97	1.49E+08	[M+H]+	89.5	-
				120.0807	70.0651	217.1337	154.0736		
Dihydrokawain	$C_{14}H_{16}O_3$	0.58	232.1101	233.1174	13.68	3.72E+07	[M+H]+	73.0	70.2
				159.0804	215.1068	141.0695	91.0543		
N-[4-[(2*R*,3*R*)-3-(hydroxymethyl)-4-methyl-5-oxomorpholin-2-yl]phenyl]acetamide	$C_{14}H_{18}N_2O_4$	-0.57	278.1265	279.1338	5.36	2.50E+09	[M+H]+	74.9	-
				136.0756	116.0705	114.0545	-		
2,6-Di-*tert*-butyl-*p*-benzoquinone	$C_{14}H_{20}O_2$	-0.37	220.1463	221.1535	13.31	3.32E+08	[M+H]+	75.4	53.4
				193.1586	123.0803	93.0698	133.1014		
Dibenzoylmethane	$C_{15}H_{12}O_2$	-0.05	224.0837	225.0910	16.74	1.47E+08	[M+H]+	80.7	-
				197.0959	105.0334	77.0385	95.0491		
10-Hydroxycarbazepine	$C_{15}H_{14}N_2O_2$	-0.14	254.1055	255.1128	12.26	1.12E+08	[M+H]+	67.8	70.3
				194.0962	192.0799	179.0727	-		
N6-Threonylcarbamoyladenosine	$C_{15}H_{20}N_6O_8$	-0.16	412.1342	413.1415	7.13	2.00E+08	[M+H]+	-	86.0
				136.0616	281.0991	162.0409	120.0654		
Tolycaine	$C_{15}H_{22}N_2O_3$	-0.69	278.1629	279.1701	9.60	4.18E+08	[M+H]+	77.9	44.3
				86.0963	120.0806	166.0860	-		
2,11,11-Trimethyl-3-oxotricyclo[4.3.2.$0^{1,5}$]undecane-9-carboxylic acid	$C_{15}H_{22}O_3$	-0.43	250.1568	251.1641	15.37	6.62E+07	[M+H]+	88.1	79.3
				187.1481	233.1536	205.1583	93.0697		
(4*S*,4*aR*)-4-(Hydroxymethyl)-3,4*a*,8,8-tetramethyl-5,6,7,8*a*-tetrahydro-4H-naphthalen-1-one	$C_{15}H_{24}O_2$	-0.01	236.1776	237.1850	17.53	8.13E+07	[M+H]+	84.9	70.6
				95.0854	109.1010	121.1010	107.0854		
6-Hydroxy-4-(hydroxymethyl)-3,4*a*,8,8-tetramethyl-5,6,7,8*a*-tetrahydro-4H-naphthalen-1-one	$C_{15}H_{24}O_3$	-0.38	252.1725	253.1798	15.76	9.48E+07	[M+H]+	86.7	70.2
				235.1690	123.0803	95.0854	109.1009		
3-(7-Methyloctyl)-3,6-dihydro-1H-uro[3,4-c]uran-4-one	$C_{15}H_{24}O_3$	0.07	252.1726	253.1799	16.93	1.24E+08	[M+H]+	77.4	52.1
				197.1171	57.0699	141.0545	179.1065		
2-(9-Decenyl)glutaconic acid	$C_{15}H_{24}O_4$	0.46	268.1676	269.1749	16.84	7.38E+07	[M+H]+	82.3	61.3
				209.1532	121.1009	163.1480	139.0751		
[5,8*a*-Bis(hydroxymethyl)-2,5-dimethyl-1,4,4*a*,6,7,8-hexahydronaphthalen-1-yl]methanol	$C_{15}H_{26}O_3$	-0.80	254.1880	255.1953	17.67	5.52E+07	[M+H]+	76.1	-
				237.1849	149.1323	219.1743	109.1011		
Methyl *cis*-9-tetradecenoate	$C_{15}H_{28}O_2$	-0.47	240.2088	241.2161	18.34	2.77E+08	[M+H]+	65.5	-
				185.1538	83.0855	135.1167	223.2056		
N-Lauroylsarcosine	$C_{15}H_{29}NO_3$	-0.67	271.2146	272.2218	20.42	2.72E+09	[M+H]+	88.5	92.0
				90.0548	57.0698	71.0855	95.0856		
3,3’,5,5’-Tetramethyldiphenoquinone	$C_{16}H_{16}O_2$	-0.54	240.1149	241.1222	18.61	9.97E+08	[M+H]+	70.6	-
				213.1273	91.0541	143.0851	129.0696		
1-(2,6-Dihydroxy-4-methoxyphenyl)-3-phenylpropan-1-one	$C_{16}H_{16}O_4$	-0.02	272.1049	255.1016	13.59	9.64E+07	[M+H-H2O]+	80.2	48.8
				213.0909	195.0804	237.0913	227.1060		

Table S19. Mass spectral features assigned at confidence level 3 as tentative candidates (continued)									
Compound Name	Molecular Formula	ΔMass [ppm]	Exact Mass	*m/z*	RT [min]	Max Area	Adduct	*mzCloud* Best Match	*mzVault* Best Match
				Fragment Ion #1	Fragment Ion #2	Fragment Ion #3	Fragment Ion #4		
Dibutyl phthalate	$C_{16}H_{22}O_4$	-0.64	278.1516	279.1589	16.13	3.74E+08	[M+H]+	71.2	52.8
				205.0855	149.0235	223.0962	167.0337		
2-Hydroxy-7-methyl-6-oxabicyclo[11.3.0]hexadeca-3,11-dien-5-one	$C_{16}H_{24}O_3$	0.01	264.1726	265.1798	16.83	6.05E+08	[M+H]+	87.5	-
				247.1690	219.1743	145.1011	95.0854		
2-[4-(2,4,4-Trimethylpentan-2-yl)phenoxy]ethanol	$C_{16}H_{26}O_2$	-0.08	250.1933	251.2005	19.16	1.51E+08	[M+H]+	80.4	92.6
				95.0854	109.1011	233.1902	81.0699		
Shogaol	$C_{17}H_{24}O_3$	-0.37	276.1724	277.1797	16.79	1.99E+08	[M+H]+	82.4	60.7
				137.0596	81.0698	235.1693	79.0541		
Valylleucyllysine	$C_{17}H_{34}N_4O_4$	-0.39	358.2579	359.2651	6.15	1.26E+08	[M+H]+	84.8	-
				84.0806	129.1020	86.0963	72.0807		
Phenylalanylphenylalanine	$C_{18}H_{20}N_2O_3$	0.30	312.1475	313.1548	9.81	1.03E+08	[M+H]+	73.3	80.0
				120.0806	166.0861	103.0541	267.1497		
Oleic acid	$C_{18}H_{34}O_2$	-0.32	282.2558	283.2631	21.86	3.75E+08	[M+H]+	81.6	82.0
				265.2524	247.2422	95.0854	121.1012		
2-Amino-4-octadecyne-1,3-diol	$C_{18}H_{35}NO_2$	-0.64	297.2666	298.2739	17.02	2.25E+08	[M+H]+	71.0	44.3
				280.2634	69.0698	67.0541	109.1011		
Leucylleucyllysine	$C_{18}H_{36}N_4O_4$	-0.10	372.2736	373.2809	7.18	6.42E+07	[M+H]+	82.1	-
				84.0807	86.0964	197.1647	242.1862		
Sphinganine	$C_{18}H_{39}NO_2$	-0.63	301.2979	302.3052	21.00	2.89E+07	[M+H]+	68.8	83.2
				60.0443	284.2945	69.0698	95.0853		
Bis(2-butoxyethyl) 2-(3-hydroxybutoxy)ethyl phosphate	$C_{18}H_{39}O_8P$	-0.15	414.2382	415.2455	16.80	1.61E+08	[M+H]+	-	75.8
				57.0698	73.0647	98.9841	124.9996		
Nalorphine	$C_{19}H_{21}NO_3$	-0.23	311.1521	312.1594	18.21	5.14E+07	[M+H]+	67.1	-
				227.0702	199.0751	84.0808	169.0648		
Iohexol	$C_{19}H_{26}I_3N_3O_9$	-0.92	820.8796	821.8868	3.81	1.86E+08	[M+H]+	-	73.8
				803.8757	602.9136	652.9745	-		
Formestane	$C_{19}H_{26}O_3$	-0.04	302.1882	303.1955	13.35	1.42E+08	[M+H]+	75.6	68.5
				257.1899	285.1851	239.1793	199.1481		
Androstanolone	$C_{19}H_{30}O_2$	0.08	290.2246	291.2319	21.33	2.75E+07	[M+H]+	80.9	77.0
				273.2216	95.0853	81.0698	109.1011		
Kahweol	$C_{20}H_{26}O_3$	-0.20	314.1881	315.1954	16.65	2.58E+08	[M+H]+	83.9	75.4
				147.0804	269.1901	91.0542	187.1117		
5-Fluoro AMB-PICA (MMB-2201; **Figure S14**)	$C_{20}H_{27}FN_2O_3$	0.01	362.2006	363.2078	17.17	1.41E+08	[M+H]+	84.0	-
				232.1130	144.0440	57.0699	69.0704		
Grandiflorenic acid	$C_{20}H_{28}O_2$	-0.34	300.2088	301.2161	21.14	1.35E+08	[M+H]+	90.3	79.8
				255.2106	133.1010	109.1012	121.1010		
7-Ethenyl-1,1,7-trimethyl-9-oxo-2,3,4,4*b*,5,6,10,10*a*-octahydrophenanthrene-4*a*-carboxylic acid	$C_{20}H_{28}O_3$	-0.58	316.2037	317.2109	18.31	4.71E+08	[M+H]+	88.5	86.3
				271.2055	253.1950	299.2008	197.1323		
Marrubin	$C_{20}H_{28}O_4$	-0.40	332.1986	333.2059	16.31	1.61E+08	[M+H]+	86.4	81.0
				287.2005	315.1955	269.1900	145.1010		

Table S19. Mass spectral features assigned at confidence level 3 as tentative candidates (continued)									
Compound Name	Molecular Formula	ΔMass [ppm]	Exact Mass	*m/z*	RT [min]	Max Area	Adduct	*mzCloud* Best Match	*mzVault* Best Match
				Fragment Ion #1	Fragment Ion #2	Fragment Ion #3	Fragment Ion #4		
Prostaglandin A3	$C_{20}H_{28}O_4$	-0.20	332.1987	333.2060	19.04	7.59E+07	[M+H]+	84.3	67.8
				315.1948	269.1898	95.0854	297.1843		
5-Ketoeicosatetraenoic acid	$C_{20}H_{30}O_3$	-0.40	318.2194	319.2266	20.18	8.98E+07	[M+H]+	85.8	76.8
				273.2212	109.1010	255.2105	301.2157		
4’-Ethenyl-2’-hydroxy-1,4’,4*a*-trimethyl-5-oxospiro[2,3,4,7,8,8*a*-hexahydronaphthalene-6,1’-cyclopentane]-1-carboxylic acid	$C_{20}H_{30}O_4$	-0.22	334.2143	335.2217	14.39	1.12E+08	[M+H]+	86.5	78.1
				271.2062	289.2172	299.2005	145.1010		
7-Hydroxy-1,4*a*-dimethyl-9-oxo-7-propan-2-yl-2,3,4,4b,5,6,10,10*a*-octahydrophenanthrene-1-carboxylic acid	$C_{20}H_{30}O_4$	-1.20	334.2140	335.2212	17.46	3.34E+08	[M+H]+	83.5	73.9
				317.2104	271.2059	201.1638	247.1692		
6-Oxocativic acid	$C_{20}H_{32}O_3$	-0.45	320.2350	321.2423	17.94	1.27E+08	[M+H]+	86.2	82.3
				275.2369	257.2263	109.1011	303.2321		
(+/-)11,12-EpETrE	$C_{20}H_{32}O_3$	-0.28	320.2351	321.2423	19.59	8.71E+08	[M+H]+	86.2	80.1
				303.2318	201.1637	81.0698	121.1010		
Prostaglandin B1	$C_{20}H_{32}O_4$	-0.90	336.2298	337.2364	17.29	1.82E+07	[M+H]+	84.2	53.2
				301.2146	319.2277	-	-		
20β-Dihydrocortisol	$C_{21}H_{32}O_5$	-0.35	364.2249	365.2321	15.33	5.27E+07	[M+H]+	81.2	67.4
				347.2216	329.2105	311.1990	149.1327		
1-Hexadecanoyl-sn-glycero-3-phosphoethanolamine	$C_{21}H_{44}NO_7P$	0.19	453.2856	454.2929	22.85	8.14E+07	[M+H]+	60.9	74.6
				313.2739	282.2781	71.0853	98.9841		
Bis(methylbenzylidene)sorbitol	$C_{22}H_{26}O_6$	-0.44	386.1728	387.1801	15.77	6.19E+08	[M+H]+	90.2	94.0
				105.0698	79.0541	103.0541	119.0494		
Docosahexaenoic acid	$C_{22}H_{32}O_2$	-0.18	328.2402	329.2475	19.69	2.14E+08	[M+H]+	88.4	38.6
				147.1166	161.1323	311.2370	95.0854		
Testosterone propionate	$C_{22}H_{32}O_3$	-0.29	344.2350	345.2423	19.55	1.97E+08	[M+H]+	89.0	86.8
				97.0646	109.0646	271.2056	81.0698		
Medrysone	$C_{22}H_{32}O_3$	-0.22	344.2351	345.2423	15.00	3.22E+07	[M+H]+	81.6	79.5
				327.2321	309.2218	105.0697	93.0697		
Docosapentaenoic acid	$C_{22}H_{34}O_2$	-0.11	330.2558	331.2631	20.32	1.52E+07	[M+H]+	80.3	-
				81.0698	93.0698	79.0542	67.0541		
Eicosapentaenoic acid ethyl ester	$C_{22}H_{34}O_2$	0.11	330.2559	331.2632	20.31	1.08E+07	[M+H]+	74.1	-
				81.0698	93.0697	105.0698	133.1009		
(+/-)5(6)-EET Ethanolamide	$C_{22}H_{37}NO_3$	0.05	363.2774	364.2846	15.54	4.58E+08	[M+H]+	68.6	-
				346.2740	145.1009	107.0854	81.0698		
Leucomalachite green	$C_{23}H_{26}N_2$	-0.52	330.2094	331.2167	12.60	4.47E+06	[M+H]+	78.2	57.7
				316.1950	301.1692	315.1868	-		
Bis(4-ethylbenzylidene)sorbitol	$C_{24}H_{30}O_6$	-0.36	414.2041	415.2114	17.32	1.97E+08	[M+H]+	91.5	-
				119.0854	91.0541	117.0700	133.0647		
Megestrol acetate	$C_{24}H_{32}O_4$	-0.44	384.2299	385.2372	18.14	2.11E+08	[M+H]+	93.9	96.6
				267.1741	325.2160	224.1558	209.1322		
Docosahexaenoic acid ethyl ester	$C_{24}H_{36}O_2$	-0.16	356.2715	357.2788	16.62	5.93E+07	[M+H]+	74.2	54.0
				135.1167	121.1011	119.0854	109.1011		

Table S19. Mass spectral features assigned at confidence level 3 as tentative candidates (continued)

Compound Name	Molecular Formula	ΔMass [ppm]	Exact Mass	*m/z*	RT [min]	Max Area	Adduct	*mzCloud* Best Match	*mzVault* Best Match
				Fragment Ion #1	Fragment Ion #2	Fragment Ion #3	Fragment Ion #4		
Bis(2-Ethylhexyl) phthalate	$C_{24}H_{38}O_4$	-0.03	390.2770	391.2843	24.79	1.24E+08	[M+H]+	83.4	87.4
				149.0231	71.0854	57.0698	167.0339		
Diisononyl phthalate	$C_{26}H_{42}O_4$	-0.37	418.3082	419.3155	25.51	2.86E+08	[M+H]+	88.0	94.1
				149.0231	71.0855	57.0698	85.1010		
Δ^4-Dafachronic acid	$C_{27}H_{42}O_3$	0.49	414.3136	415.3209	23.53	1.47E+08	[M+H]+	64.5	89.1
				109.0647	97.0647	81.0698	123.0804		
Demissidine	$C_{27}H_{45}NO$	-0.34	399.3500	400.3573	14.97	4.23E+07	[M+H]+	-	68.9
				98.0964	126.1284	-	-		
Lupeol	$C_{30}H_{50}O$	-0.12	426.3861	427.3933	28.10	2.17E+07	[M+H]+	69.8	55.5
				95.0854	109.1010	149.1326	137.1330		
Stercobilin	$C_{33}H_{46}N_4O_6$	0.02	594.3417	595.3490	12.13	4.52E+08	[M+H]+	77.7	-
				470.2648	344.1713	-	-		
1,2-Dipalmitoylglycerol	$C_{35}H_{68}O_5$	-0.42	568.5064	591.4956	28.48	3.33E+08	[M+Na]+	-	63.7
				313.2734	335.2575	71.0858	-		
Methyl (2*R*)-2-aminopropanoate	$C_4H_9NO_2$	-0.85	103.0632	104.0705	1.77	5.31E+07	[M+H]+	38.7	87.1
				60.0807	87.0440	58.0651	-		
Cadaverine	$C_5H_{14}N_2$	-0.76	102.1156	103.1229	1.56	4.55E+06	[M+H]+	-	87.1
				56.9647	84.9596	86.0963	103.1228		
Tiglinic acid	$C_5H_8O_2$	-0.96	100.0523	101.0596	5.38	7.65E+08	[M+H]+	83.3	78.1
				83.0491	73.0649	57.0334	59.0491		
Levoglucosan	$C_6H_{10}O_5$	1.47	162.0531	163.0603	11.32	5.47E+08	[M+H]+	-	77.5
				149.0447	133.0498	-	-		
Maltol	$C_6H_6O_3$	-1.36	126.0315	127.0388	1.91	2.11E+10	[M+H]+	91.6	94.0
				109.0283	81.0335	53.0386	55.0178		
3-Hydroxy-2-methylpyridine	C_6H_7NO	-1.22	109.0526	110.0599	2.23	5.60E+07	[M+H]+	94.2	72.2
				82.0650	92.0495	67.0418	80.0494		
2-Aminophenol	C_6H_7NO	-1.19	109.0526	110.0599	2.12	1.08E+07	[M+H]+	93.3	70.1
				92.0495	82.0650	65.0387	-		
Methylimidazoleacetic acid	$C_6H_8N_2O_2$	-0.38	140.0585	141.0658	2.06	2.15E+08	[M+H]+	89.3	82.1
				95.0602	81.0447	68.0494	54.0339		
2-(Hydroxymethyl)-3-methoxy-2H-furan-5-one	$C_6H_8O_4$	-1.86	144.0420	145.0493	1.86	3.11E+09	[M+H]+	87.2	-
				99.0439	127.0388	71.0491	85.0283		
3-Hydroxy-4-methoxy-2,3-dihydropyran-6-one	$C_6H_8O_4$	-0.93	144.0421	145.0494	1.91	1.27E+10	[M+H]+	81.1	-
				85.0283	69.0334	99.0439	127.0389		
1-Methylhistidine	$C_7H_{11}N_3O_2$	-0.52	169.0850	170.0923	1.73	1.81E+07	[M+H]+	87.1	87.1
				124.0868	96.0681	109.0757	83.0602		
trans-2-Aminomethyl-1-cyclohexanol	$C_7H_{15}NO$	-0.45	129.1153	130.1226	15.02	1.91E+08	[M+H]+	90.8	-
				95.0854	112.1120	67.0542	55.0541		
Acetylcholine	$C_7H_{16}NO_2^+$	-0.65	146.1176	146.1175	2.08	1.28E+08	[M]+	90.8	91.4
				87.0440	60.0807	-	-		

Table S19. Mass spectral features assigned at confidence level 3 as tentative candidates (continued)

Compound Name	Molecular Formula	ΔMass [ppm]	Exact Mass	*m/z*	RT [min]	Max Area	Adduct	*mzCloud* Best Match	*mzVault* Best Match
				Fragment Ion #1	Fragment Ion #2	Fragment Ion #3	Fragment Ion #4		
6-Dimethylaminopurine	$C_7H_9N_5$	-0.05	163.0858	164.0931	3.57	3.12E+08	[M+H]+	75.9	-
				123.0665	162.0776	94.0400	96.0556		
2,4-Dimethylaniline	$C_8H_{11}N$	-0.59	121.0891	122.0964	3.51	2.03E+08	[M+H]+	87.3	92.4
				79.0542	107.0727	105.0699	81.0699		
2,4,6-Trimethylpyridine	$C_8H_{11}N$	-0.57	121.0891	122.0964	3.66	4.09E+08	[M+H]+	87.7	90.4
				79.0542	107.0729	81.0699	106.0651		
2-Methoxy-5-methylaniline	$C_8H_{11}NO$	-0.44	137.0840	138.0913	11.43	2.38E+08	[M+H]+	80.1	79.3
				123.0678	79.0542	120.0808	108.0813		
2,3,5,6-Tetramethylpyrazine	$C_8H_{12}N_2$	-0.85	136.0999	137.1072	3.38	7.34E+08	[M+H]+	73.5	-
				122.0843	55.0544	81.0701	109.0760		
3-Hydroxyquinuclidine-3-carbonitrile	$C_8H_{12}N_2O$	-0.41	152.0949	153.1022	4.78	9.67E+07	[M+H]+	78.7	42.1
				55.0542	68.0495	125.0710	135.0917		
5-Ethylcyclohexane-1,3-dione	$C_8H_{12}O_2$	-0.59	140.0837	141.0909	8.90	1.06E+08	[M+H]+	72.6	-
				95.0853	123.0805	67.0544	81.0337		
3-*tert*-Butyl-1-methyl-2-pyrazolin-5-one	$C_8H_{14}N_2O$	-0.34	154.1106	155.1178	4.77	6.44E+07	[M+H]+	66.3	-
				95.0853	81.0699	55.0543	109.1011		
Alanylproline	$C_8H_{14}N_2O_3$	-0.42	186.1004	187.1076	2.23	1.88E+08	[M+H]+	90.0	-
				116.0704	70.0651	-	-		
Tranexamic acid	$C_8H_{15}NO_2$	-0.27	157.1102	158.1175	8.45	5.64E+08	[M+H]+	62.2	31.7
				67.0543	112.1118	95.0855	85.0646		
Sulcatol	$C_8H_{16}O$	-0.59	128.1200	129.1274	11.61	9.60E+07	[M+H]+	89.2	-
				69.0698	111.1167	57.0698	55.0542		
Coniine	$C_8H_{17}N$	-0.16	127.1361	128.1434	7.78	5.25E+08	[M+H]+	86.3	58.3
				69.0698	55.0542	111.1165	72.0807		
Phthalic acid	$C_8H_6O_4$	-0.31	166.0266	149.0233	14.36	3.14E+08	[M+H-H2O]+	-	75.5
				121.0282	65.0385	93.0333	-		
Acetophenone	C_8H_8O	-0.78	120.0574	121.0647	12.22	4.06E+08	[M+H]+	82.9	59.4
				93.0698	91.0542	79.0542	105.0447		
4-Methoxybenzaldehyde	$C_8H_8O_2$	-0.54	136.0524	137.0596	6.35	4.31E+08	[M+H]+	91.1	58.6
				94.0412	109.0647	79.0542	122.0361		
3’-Aminoacetophenone	C_8H_9NO	-0.71	135.0683	136.0756	2.58	4.87E+08	[M+H]+	74.3	87.5
				91.0542	119.0491	94.0654	-		
2,4-Dimethylbenzaldehyde	$C_9H_{10}O$	-0.55	134.0731	135.0804	15.65	2.38E+08	[M+H]+	92.8	-
				91.0542	105.0699	107.0855	119.0490		
2-Hydroxyphenylalanine	$C_9H_{11}NO_3$	-0.10	181.0739	182.0811	7.33	5.78E+07	[M+H]+	79.5	72.9
				136.0756	119.0492	164.0704	-		
Cytidine	$C_9H_{13}N_3O_5$	-0.45	243.0854	244.0927	2.08	2.72E+07	[M+H]+	89.3	89.8
				112.0504	95.0240	-	-		
Cyclo(prolylthreonyl)	$C_9H_{14}N_2O_3$	-0.42	198.1004	199.1076	4.35	1.36E+08	[M+H]+	73.0	-
				163.0864	181.0970	153.1020	125.0706		

Table S19. Mass spectral features assigned at confidence level 3 as tentative candidates (continued)									
Compound Name	Molecular Formula	ΔMass [ppm]	Exact Mass	*m/z* / Fragment Ion #1	RT [min] / Fragment Ion #2	Max Area / Fragment Ion #3	Adduct / Fragment Ion #4	*mzCloud* Best Match	*mzVault* Best Match
Ecgonine	$C_9H_{15}NO_3$	-0.64	185.1051	186.1124	9.57	3.00E+08	[M+H]+	71.7	42.9
				168.1011	122.0958	108.0808	83.0854		
Triisopropanolamine cyclic borate	$C_9H_{18}BNO_3$	-0.37	199.1379	200.1452	3.79	4.16E+07	[M+H]+	69.6	71.5
				142.1034	182.1360	98.0967	56.0494		
Alanylleucine	$C_9H_{18}N_2O_3$	-0.63	202.1316	203.1389	3.14	4.75E+08	[M+H]+	-	96.3
				86.0963	69.0698	-	-		
Isoquinoline	C_9H_7N	-0.12	129.0578	130.0651	10.21	5.41E+08	[M+H]+	82.8	75.5
				103.0542	57.0699	128.0498	77.0388		
8-Hydroxyquinoline	C_9H_7NO	-0.40	145.0527	146.0600	9.65	1.41E+08	[M+H]+	91.6	91.7
				128.0494	118.0647	91.0539	-		
2-Hydroxyquinoline	C_9H_7NO	-0.49	145.0527	146.0600	9.66	8.36E+08	[M+H]+	89.2	92.0
				128.0493	132.8313	-	-		
1-Phenyl-1H-pyrazole	$C_9H_8N_2$	-0.09	144.0687	145.0760	9.48	1.00E+09	[M+H]+	82.7	-
				128.0494	104.0496	118.0653	77.0387		
2-Hydroxycinnamic acid	$C_9H_8O_3$	-0.58	164.0473	165.0545	2.69	7.90E+08	[M+H]+	92.6	79.5
				123.0440	95.0490	119.0490	147.0439		
4-Oxo-4,5,6,7-tetrahydro-1-benzofuran-3-carboxylic acid	$C_9H_8O_4$	-0.88	180.0421	181.0494	7.24	2.61E+08	[M+H]+	73.4	58.8
				135.0441	163.0394	107.0492	79.0543		
4-Acetamidobenzaldehyde	$C_9H_9NO_2$	-0.06	163.0633	164.0706	7.41	6.52E+07	[M+H]+	75.9	-
				136.0756	77.0385	122.0596	53.0384		
Hippuric acid	$C_9H_9NO_3$	-0.24	179.0582	180.0655	7.27	2.71E+08	[M+H]+	86.8	92.9
				105.0334	77.0385	95.0491	162.0548		
α-Hydroxyhippuric acid	$C_9H_9NO_4$	-0.68	195.0530	196.0603	8.55	1.04E+08	[M+H]+	85.9	85.1
				121.0283	65.0385	93.0335	111.0445		
N-Methyl homarylamine (**Figure S13**)	$C_{11}H_{15}NO_2$	-0.57	193.1102	194.1174	5.48	1.79E+08	[M+H]+	83.3	-
				149.0595	91.0541	58.0651	121.0646		
Methylphenidate	$C_{14}H_{19}NO_2$	-0.43	233.1415	234.1488	9.27	1.91E+07	[M+H]+	84.0	-
				84.0807	56.0497	-	-		
Etodolac	$C_{17}H_{21}NO_3$	-0.35	287.1520	288.1593	17.73	1.23E+07	[M+H]+	89.7	90.8
				172.1119	270.1489	210.1276	224.1433		
Piperanine	$C_{17}H_{21}NO_3$	-0.38	287.1520	288.1593	16.71	5.95E+06	[M+H]+	74.7	53.1
				86.0965	131.0492	135.0438	103.0544		
Nor-6β-oxycodol	$C_{17}H_{21}NO_4$	0.57	303.1472	304.1545	5.55	9.19E+06	[M+H]+	86.4	68.4
				286.1442	213.0914	242.1179	-		
2-Isopropylaniline	$C_9H_{13}N$	-0.53	135.1047	136.1120	4.34	1.40E+07	[M+H]+	78.6	91.3
				91.0540	94.0651	118.0650	65.0385		

Table S20. Fragment ions and neutral loss of tapentadol confirmed at confidence level 1 by reference standard

Molecular Structure	Molecular Formula	ΔMass [ppm]	Exact Mass	Adduct	*m/z*	RT [min]	Area (Max.)
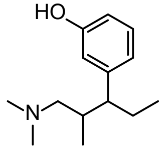	$C_{14}H_{23}NO$	0.15	221.1780	[M+H]+	222.1850	9.51	1.03E+08
Neutral Loss	Formula	ΔMass [ppm]	Exact Mass	Neutral Loss	Formula	ΔMass [ppm]	Exact Mass
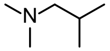	$C_6H_{15}N$	0.17	101.1205				
Fragment Ion	Formula	ΔMass [ppm]	*m/z*	Fragment Ion	Formula	ΔMass [ppm]	*m/z*
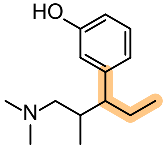	$[C_4H_6]$+H+	-0.73	55.0542	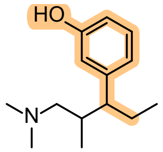	$[C_8H_8O]$+H+	-1.32	121.0647
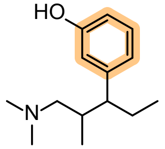	$[C_6H_4]$+H+	-0.52	77.0386	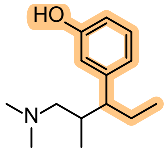	$[C_9H_{11}O]$+	-1.11	135.0803
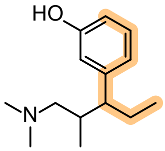	$[C_7H_9]$+	-0.32	93.0699	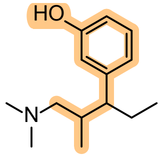	$[C_{10}H_{12}O$-H]+	1.16	147.0807
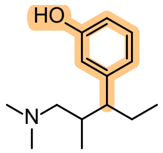	$[C_7H_6O]$+H+	-1.49	107.0490	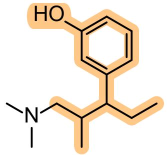	$[C_{12}H_{17}O]$+	0.34	177.1275

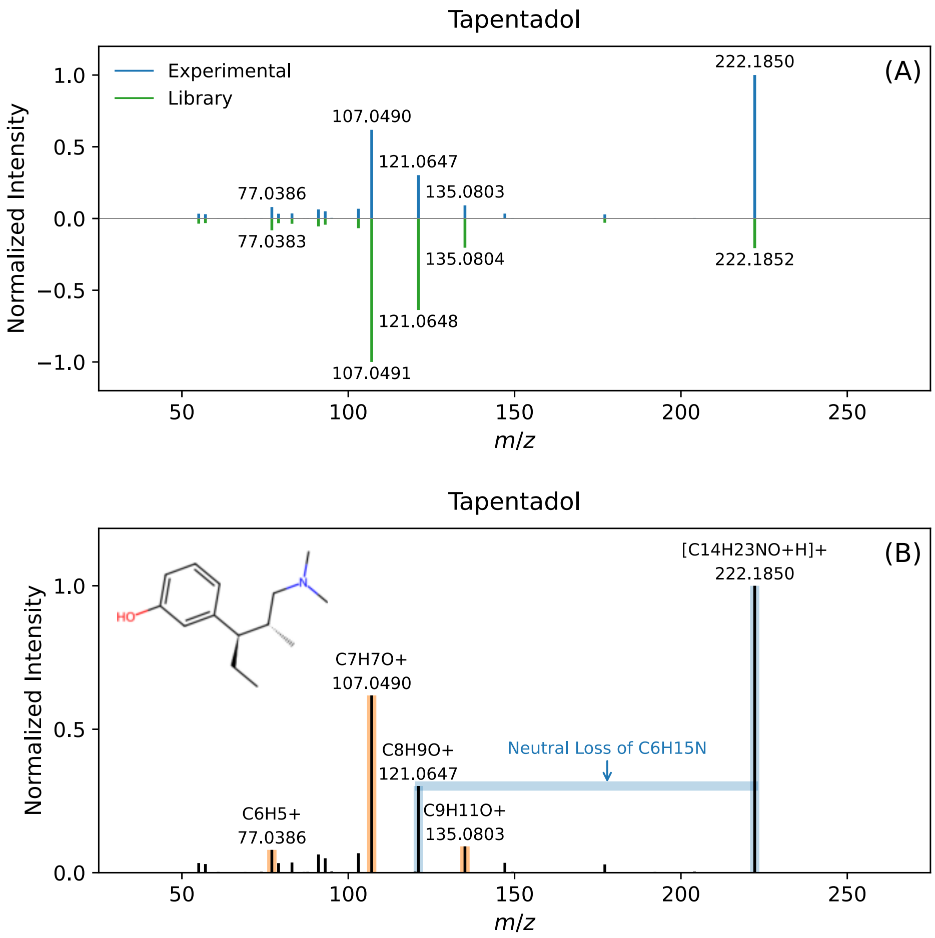

Figure S5. Tapentadol confirmed at confidence level 1 by reference standard: **(A)** Head-to-tail plots of experimental (top) and library (bottom) dd-MS2 spectra of tapentadol. **(B)** Experimental dd-MS2 spectrum of tapentadol highlighted with fragment ions (Table S20) and the neutral loss of $C_6H_{15}N$.

Table S21. Fragment ions and neutral losses of levorphanol confirmed at confidence level 1 by reference standard							
Molecular Structure	Molecular Formula	ΔMass [ppm]	Exact Mass	Adduct	*m/z*	RT [min]	Area (Max.)
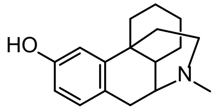	$C_{17}H_{23}NO$	-0.05	257.1780	[M+H]+	258.1851	8.76	1.71E+08
Neutral Loss	Formula	ΔMass [ppm]	Exact Mass	Neutral Loss	Formula	ΔMass [ppm]	Exact Mass
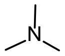	C_3H_9N	-0.16	59.0735	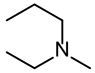	$C_6H_{15}N$	0.88	101.1205
Fragment Ion	Formula	ΔMass [ppm]	*m/z*	Fragment Ion	Formula	ΔMass [ppm]	*m/z*
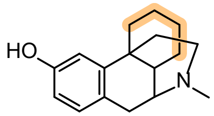	$[C_4H_8\text{-}H]^+$	-3.81	55.0541	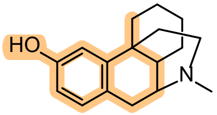	$[C_{11}H_9O]^+$	0.89	157.0649
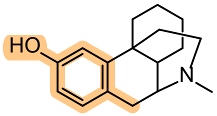	$[C_7H_6O]+H^+$	0.84	107.0493	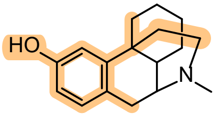	$[C_{11}H_{12}O\text{-}H]^+$	1.07	159.0807
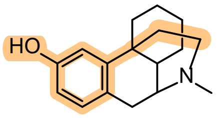	$[C_9H_8O]+H^+$	-0.75	133.0647	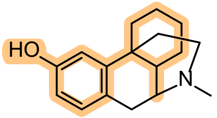	$[C_{13}H_{14}O\text{-}H]^+$	-1.35	185.0954
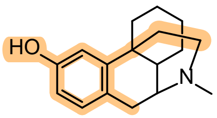	$[C_{10}H_{10}O\text{-}H]^+$	-0.90	145.0647	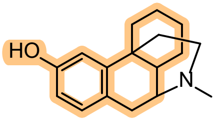	$[C_{14}H_{16}O\text{-}H]^+$	-0.50	199.1117

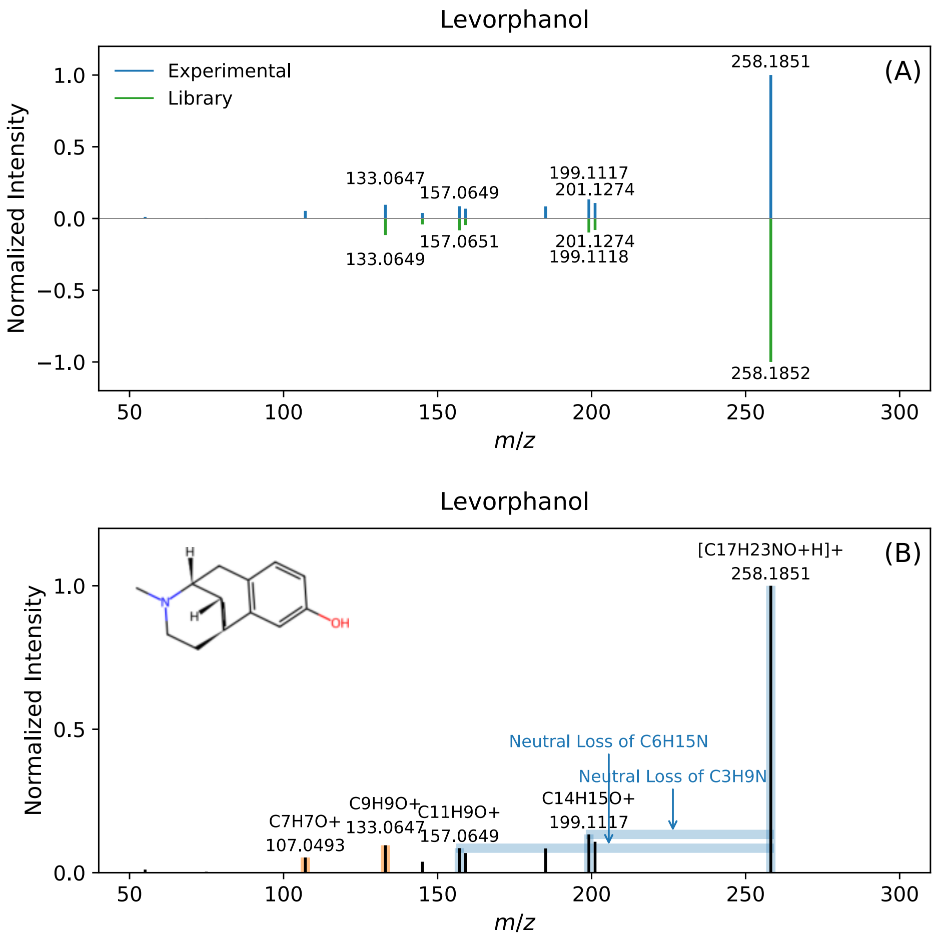

Figure S6. Levorphanol confirmed at confidence level 1 by reference standard: **(A)** Head-to-tail plots of experimental (top) and library (bottom) dd-MS2 spectra of levorphanol. **(B)** Experimental dd-MS2 spectrum of levorphanol highlighted with fragment ions (Table S21) and the neutral losses of C_3H_9N and $C_6H_{15}N$.

**Table S22.** Fragment ions and neutral losses of *N*-ethylamphetamine confirmed at confidence level 1 by reference standard							
Molecular Structure	Molecular Formula	ΔMass [ppm]	Exact Mass	Adduct	*m/z*	RT [min]	Area (Max.)
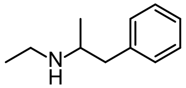	$C_{11}H_{17}N$	-0.50	163.1360	[M+H]+	164.1433	8.76	3.47E+08
Neutral Loss	Formula	ΔMass [ppm]	Exact Mass	Neutral Loss	Formula	ΔMass [ppm]	Exact Mass
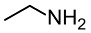	C_2H_7N	-0.05	45.0579	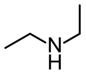	$C_4H_{11}N$	0.04	73.0892
Fragment Ion	Formula	ΔMass [ppm]	*m/z*	Fragment Ion	Formula	ΔMass [ppm]	*m/z*
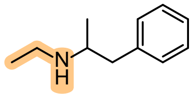	$[C_2H_6N+H]+H^+$	-0.22	46.0652	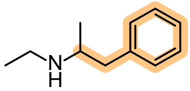	$[C_8H_8]^+$	0.19	104.0621
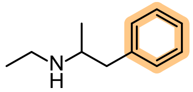	$[C_6H_5+H]+H^+$	-1.52	79.0541	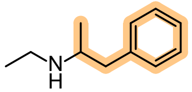	$[C_9H_{11}]^+$	-1.18	119.0854
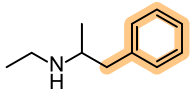	$[C_7H_7]^+$	-1.21	91.0542				

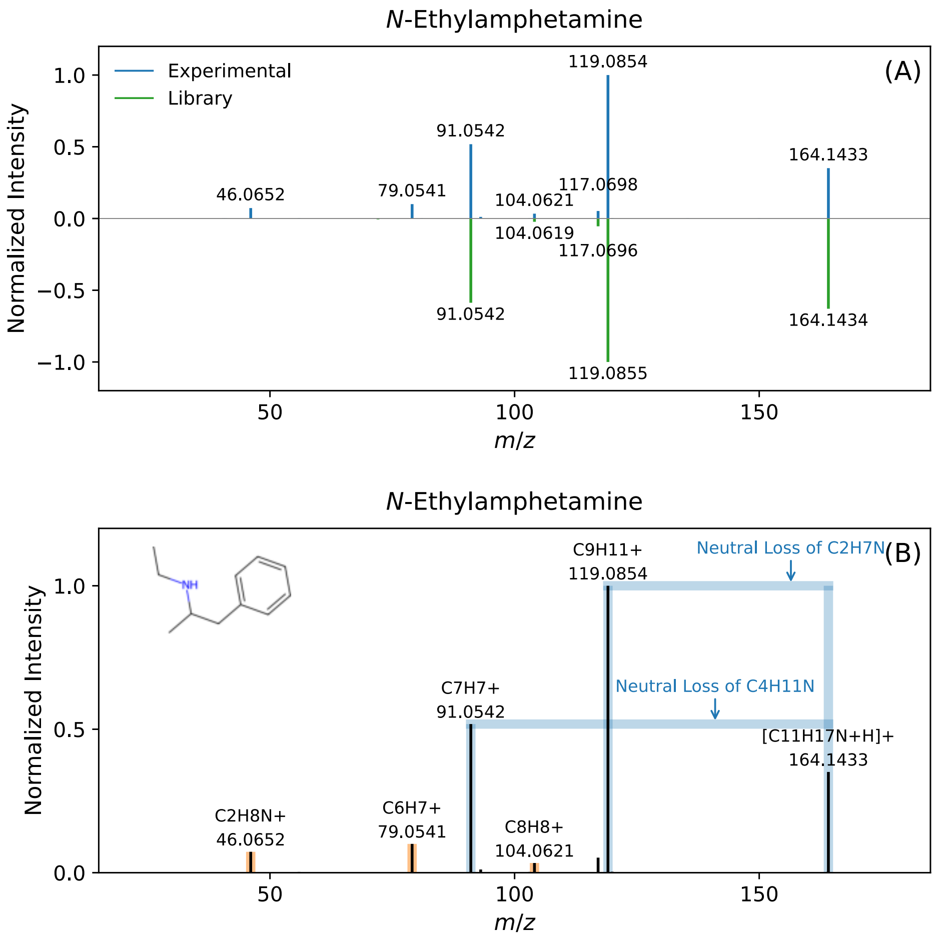

Figure S7. *N*-Ethylamphetamine confirmed at confidence level 1 by reference standard: **(A)** Head-to-tail plots of experimental (top) and library (bottom) dd-MS2 spectra of *N*-ethylamphetamine. **(B)** Experimental dd-MS2 spectrum of *N*-ethylamphetamine highlighted with fragment ions (Table S22) and the neutral losses of C_2H_7N and $C_4H_{11}N$.

Table S23. Fragment ions and neutral losses of *N,N*-dimethylpentylone (dipentylone) identified at confidence level 2 as probable structure

Molecular Structure	Molecular Formula	ΔMass [ppm]	Exact Mass	Adduct	*m/z*	RT [min]	Area (Max.)
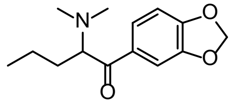	$C_{14}H_{19}NO_3$	-0.85	249.1363	[M+H]+	250.1438	9.37	8.57E+08
Neutral Loss	Formula	ΔMass [ppm]	Exact Mass	Neutral Loss	Formula	ΔMass [ppm]	Exact Mass
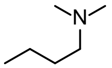	$C_6H_{15}N$	-0.71	101.1205	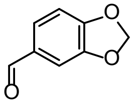	$C_8H_6O_3$	-0.02	150.0317
Fragment Ion	Formula	ΔMass [ppm]	*m/z*	Fragment Ion	Formula	ΔMass [ppm]	*m/z*
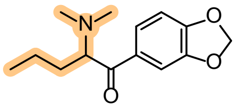	$[C_6H_{14}N]+$	-1.90	100.1120	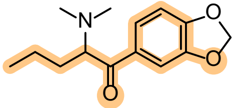	$[C_{11}H_{11}O_2]+$	-2.80	175.0749
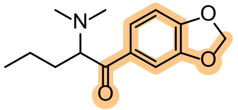	$[C_8H_5O_2+H]+H+$	0.37	135.0441	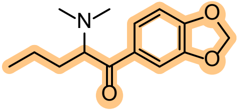	$[C_{12}H_{13}O_3]+$	0.29	205.0860
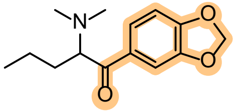	$[C_8H_5O_3]+$	1.41	149.0235				

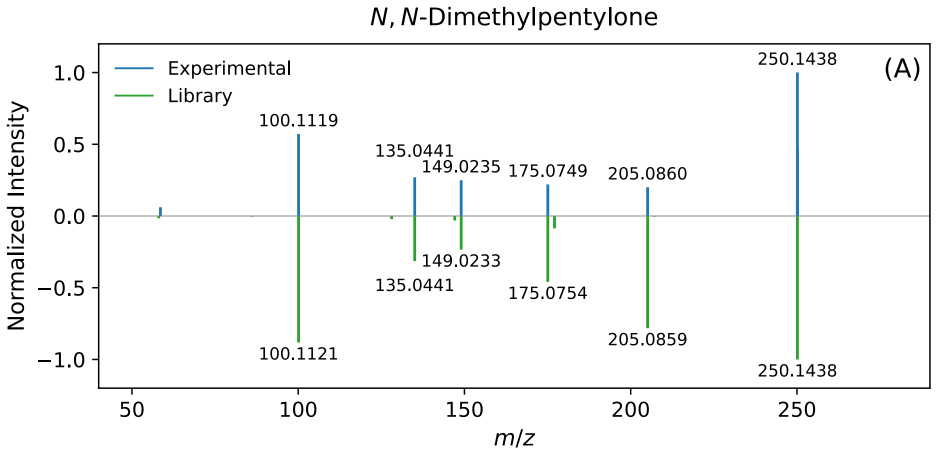

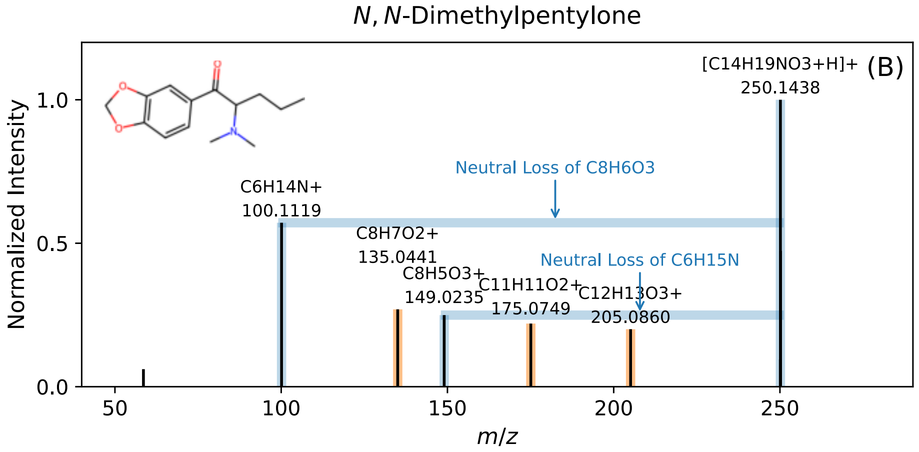

Figure S8. *N,N*-Dimethylpentylone (dipentylone) identified at confidence level 2 as probable structure: **(A)** Head-to-tail plots of experimental (top) and library (bottom) dd-MS2 spectra of *N,N*-dimethylpentylone. **(B)** Experimental dd-MS2 spectrum of *N,N*-dimethylpentylone highlighted with fragment ions (Table S23) and the neutral losses of $C_6H_{15}N$ and $C_8H_6O_3$.

Table S24. Fragment ions and neutral loss of *N*-methyl-2-aminoindane (*N*-methyl-2-AI) identified at confidence level 2 as probable structure

Molecular Structure	Molecular Formula	ΔMass [ppm]	Exact Mass	Adduct	*m/z*	RT [min]	Area (Max.)
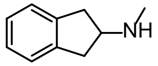	$C_{10}H_{13}N$	-0.57	147.1047	[M+H]+	148.1119	6.46	2.85E+08
Neutral Loss	Formula	ΔMass [ppm]	Exact Mass	Neutral Loss	Formula	ΔMass [ppm]	Exact Mass
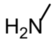	CH_5N	-0.60	31.0422				
Fragment Ion	Formula	ΔMass [ppm]	*m/z*	Fragment Ion	Formula	ΔMass [ppm]	*m/z*
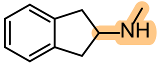	$[C_2H_5N-H]^+$	-4.52	42.0337	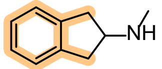	$[C_8H_8]+H^+$	-1.81	105.0697
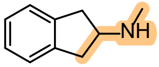	$[C_3H_7N-H]^+$	-1.43	56.0494	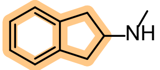	$[C_9H_8-H]^+$	-1.30	115.0541
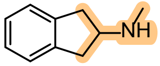	$[C_4H_9N-H]^+$	-1.71	70.0650	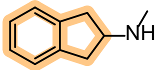	$[C_9H_9]^+$	-1.96	117.0697
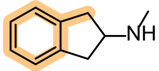	$[C_7H_6]+H^+$	-1.98	91.0541	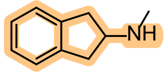	$[C_9H_{10}N]+H^+$	-1.43	133.0885

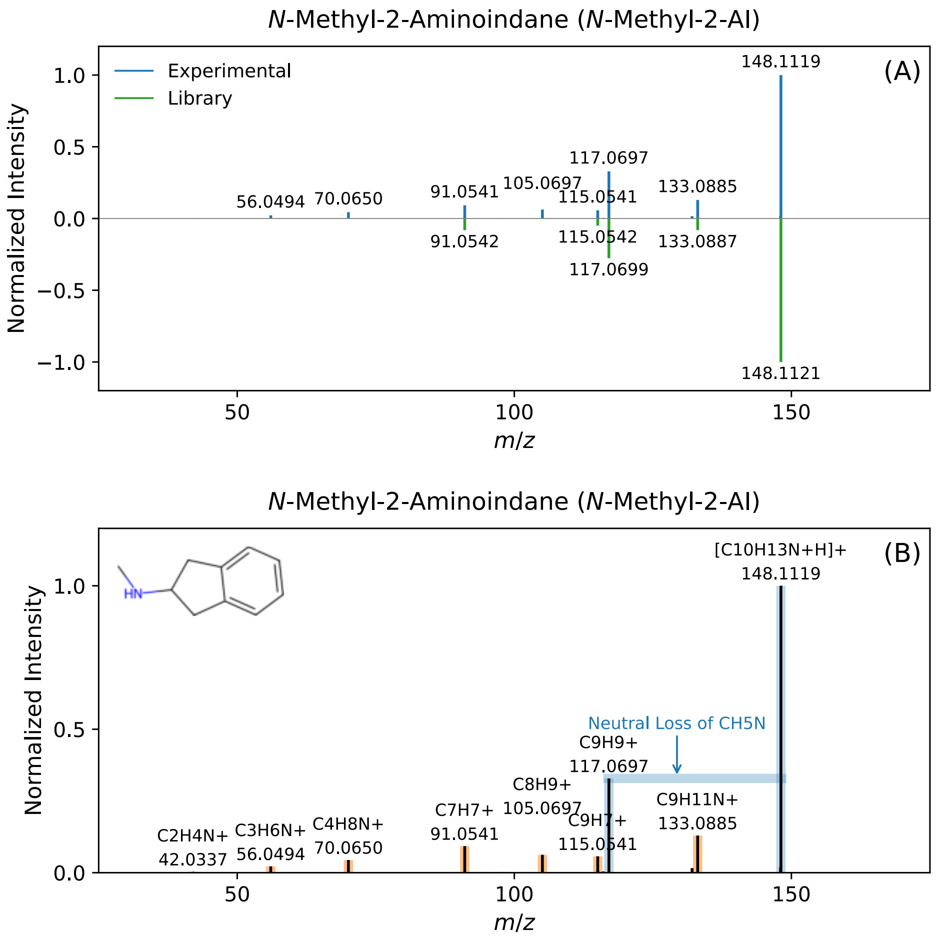

Figure S9. *N*-Methyl-2-aminoindane (*N*-methyl-2-AI) identified at confidence level 2 as probable structure: **(A)** Head-to-tail plots of experimental (top) and library (bottom) dd-MS2 spectra of *N*-methyl-2-aminoindane. **(B)** Experimental dd-MS2 spectrum of *N*-methyl-2-aminoindane highlighted with fragment ions (Table S24) and the neutral loss of CH_5N.

Table S25. Fragment ions and neutral loss of 2-phenethylamine identified at confidence level 2 as probable structure

Molecular Structure	Molecular Formula	ΔMass [ppm]	Exact Mass	Adduct	*m/z*	RT [min]	Area (Max.)
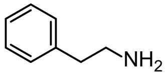	$C_8H_{11}N$	-0.64	121.0891	[M+H]+	122.0963	5.34	8.47E+08
Neutral Loss	Formula	ΔMass [ppm]	Exact Mass	Neutral Loss	Formula	ΔMass [ppm]	Exact Mass
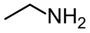	C_2H_7N	-0.90	45.0579				
Fragment Ion	Formula	ΔMass [ppm]	*m/z*	Fragment Ion	Formula	ΔMass [ppm]	*m/z*
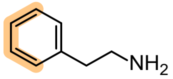	$[C_4H_4\text{-}H]^+$	3.33	51.0231	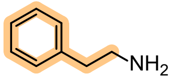	$[C_8H_8\text{-}H]^+$	-1.07	103.0541
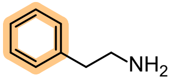	$[C_6H_5]^+$	-2.86	77.0384	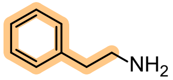	$[C_8H_9]^+$	-2.09	105.0697
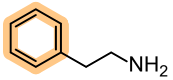	$[C_6H_6]+H^+$	-0.63	79.0542				

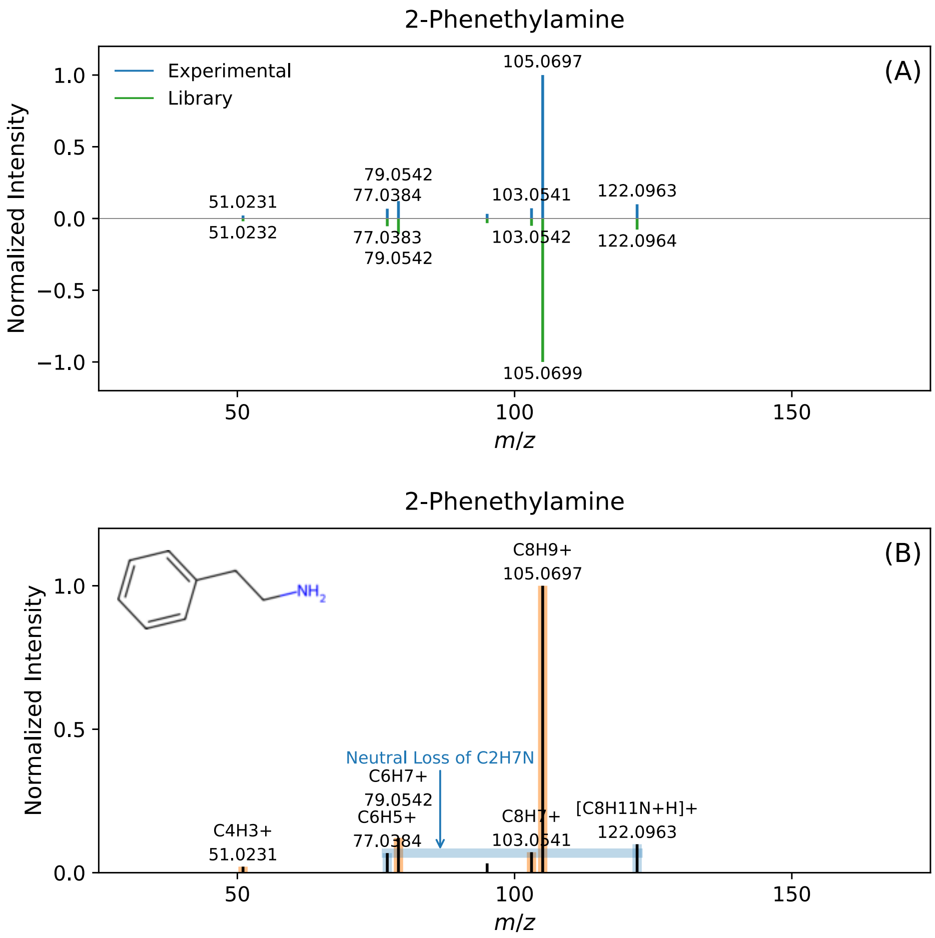

Figure S10. 2-Phenethylamine identified at confidence level 2 as probable structure: **(A)** Head-to-tail plots of experimental (top) and library (bottom) dd-MS2 spectra of 2-phenethylamine. **(B)** Experimental dd-MS2 spectrum of 2-phenethylamine highlighted with fragment ions (Table S25) and the neutral loss of C_2H_7N.

Table S26. Fragment ions and neutral losses of 4-methoxy-*N,N*-dimethylcathinone (4-methoxy-*N,N*-DMC) assigned at confidence level 3 as tentative candidate

Molecular Structure	Molecular Formula	ΔMass [ppm]	Exact Mass	Adduct	*m/z*	RT [min]	Area (Max.)
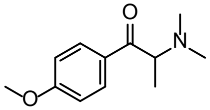	$C_{12}H_{17}NO_2$	-0.32	207.1259	[M+H]+	208.1332	13.33	1.46E+08
Neutral Loss	Formula	ΔMass [ppm]	Exact Mass	Neutral Loss	Formula	ΔMass [ppm]	Exact Mass
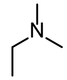	$C_4H_{11}N$	-0.85	73.0892	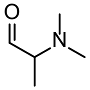	$C_5H_{11}NO$	1.77	101.0841
Fragment Ion	Formula	ΔMass [ppm]	*m/z*	Fragment Ion	Formula	ΔMass [ppm]	*m/z*
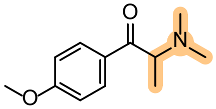	$[C_4H_{10}N+H]+H^+$	-2.29	74.0963	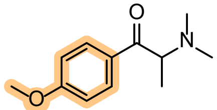	$[C_7H_7O]^+$	1.40	107.0493
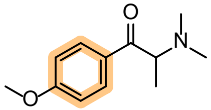	$[C_6H_4+2H]+H^+$	-0.38	79.0542	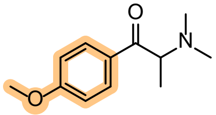	$[C_7H_7O+H]+H^+$	-0.18	109.0648
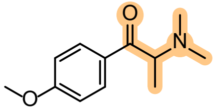	$[C_5H_{10}NO]^+$	1.30	100.0759	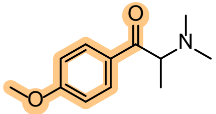	$[C_8H_7O_2]^+$	-1.11	135.0439

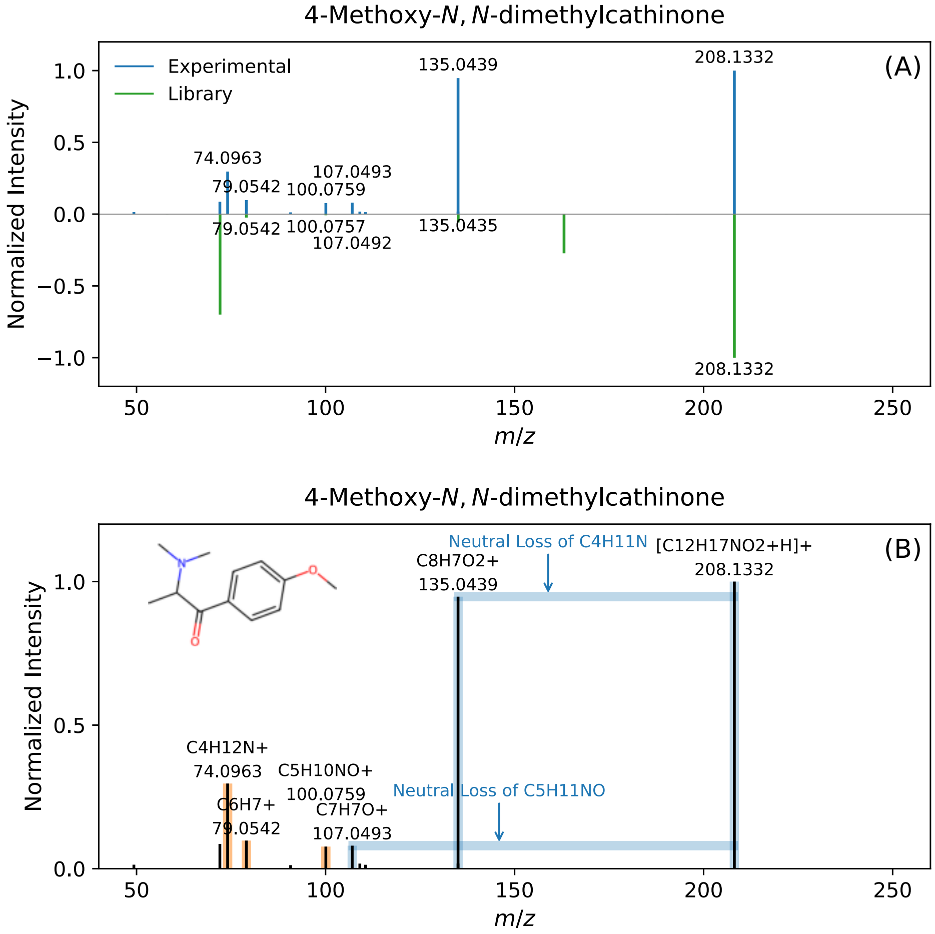

Figure S11. 4-Methoxy-*N,N*-dimethylcathinone (4-methoxy-*N,N*-DMC) assigned at confidence level 3 as tentative candidate: **(A)** Head-to-tail plots of experimental (top) and library (bottom) dd-MS2 spectra of 4-methoxy-*N,N*-dimethylcathinone. **(B)** Experimental dd-MS2 spectrum of 4-methoxy-*N,N*-dimethylcathinone highlighted with fragment ions (Table S26) and the neutral losses of $C_4H_{11}N$ and $C_5H_{11}NO$.

Table S27. Fragment ions and neutral losses of dimethylone (bk-MDDMA) assigned at confidence level 3 as tentative candidate

Molecular Structure	Molecular Formula	ΔMass [ppm]	Exact Mass	Adduct	*m/z*	RT [min]	Area (Max.)
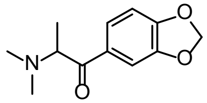	$C_{12}H_{15}NO_3$	-0.41	221.1051	[M+H]+	222.1124	5.56	9.99E+08
Neutral Loss	Formula	ΔMass [ppm]	Exact Mass	Neutral Loss	Formula	ΔMass [ppm]	Exact Mass
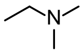	$C_4H_{11}N$	-1.87	73.0892	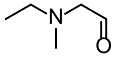	$C_5H_{11}NO$	-0.02	101.0841
Fragment Ion	Formula	ΔMass [ppm]	*m/z*	Fragment Ion	Formula	ΔMass [ppm]	*m/z*
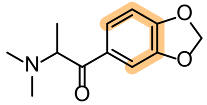	$[C_5H_3+H]+H+$	-0.77	65.0386	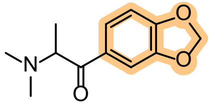	$[C_7H_5O_2]+$	0.08	121.0284
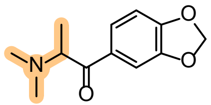	$[C_4H_{10}N]+$	-4.44	72.0805	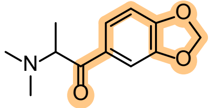	$[C_7H_5O_3+H]+H+$	-1.65	139.0388
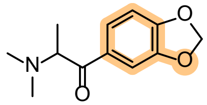	$[C_6H_3O+H]+H+$	-2.15	93.0331	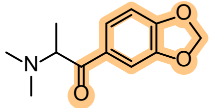	$[C_8H_5O_3]+$	-2.28	149.0230
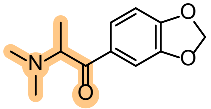	$[C_5H_{10}NO]+$	-0.10	100.0757	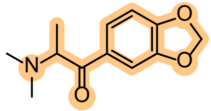	$[C_{10}H_{10}NO_3+H]+H+$	4.74	194.0821

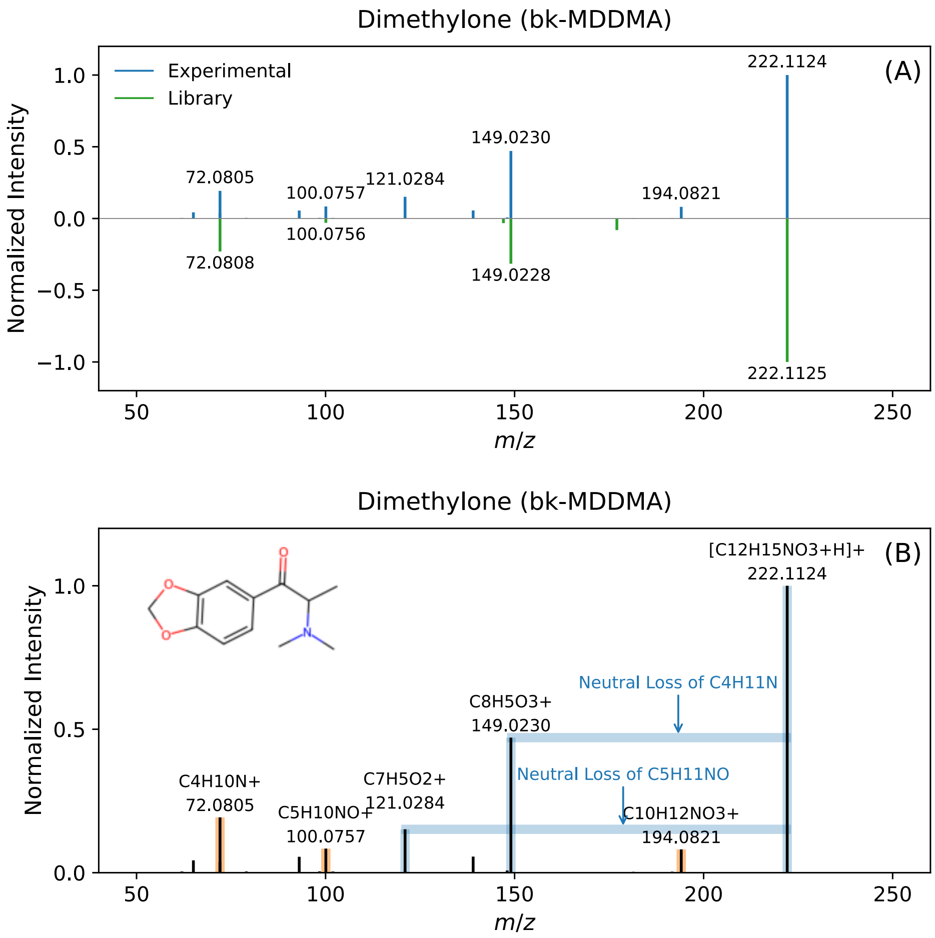

Figure S12. Dimethylone (bk-MDDMA) assigned at confidence level 3 as tentative candidate: **(A)** Head-to-tail plots of experimental (top) and library (bottom) dd-MS2 spectra of dimethylone. **(B)** Experimental dd-MS2 spectrum of dimethylone highlighted with fragment ions (Table S27) and the neutral losses of $C_4H_{11}N$ and $C_5H_{11}NO$.

Table S28. Fragment ions and neutral losses of *N*-methyl homarylamine assigned at confidence level 3 as tentative candidate

Molecular Structure	Molecular Formula	ΔMass [ppm]	Exact Mass	Adduct	*m/z*	RT [min]	Area (Max.)
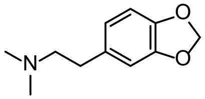	$C_{11}H_{15}NO_2$	-0.57	193.1102	[M+H]+	194.1174	5.48	1.79E+08
Neutral Loss	Formula	ΔMass [ppm]	Exact Mass	Neutral Loss	Formula	ΔMass [ppm]	Exact Mass
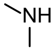	C_2H_7N	-0.15	45.0579	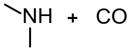	C_3H_7NO	0.18	73.0528
Fragment Ion	Formula	ΔMass [ppm]	*m/z*	Fragment Ion	Formula	ΔMass [ppm]	*m/z*
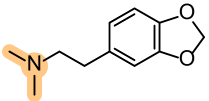	$[C_2H_6N+H]+H+$	-1.52	46.0651	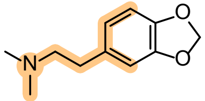	$[C_7H_{10}N]+$	-0.19	108.0808
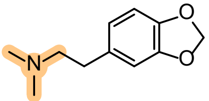	$[C_3H_8N]+$	-0.34	58.0651	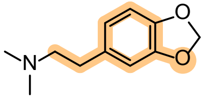	$[C_8H_7O+H]+H+$	-1.82	121.0646
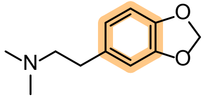	$[C_6H_3+H]+H+$	1.95	77.0388	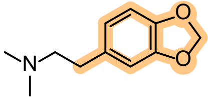	$[C_8H_7O_2]+$	0.81	135.0442
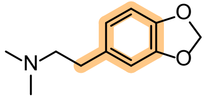	$[C_7H_5+H]+H+$	-1.43	91.0541	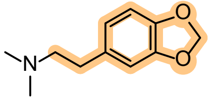	$[C_9H_9O_2]+$	-1.68	149.0595

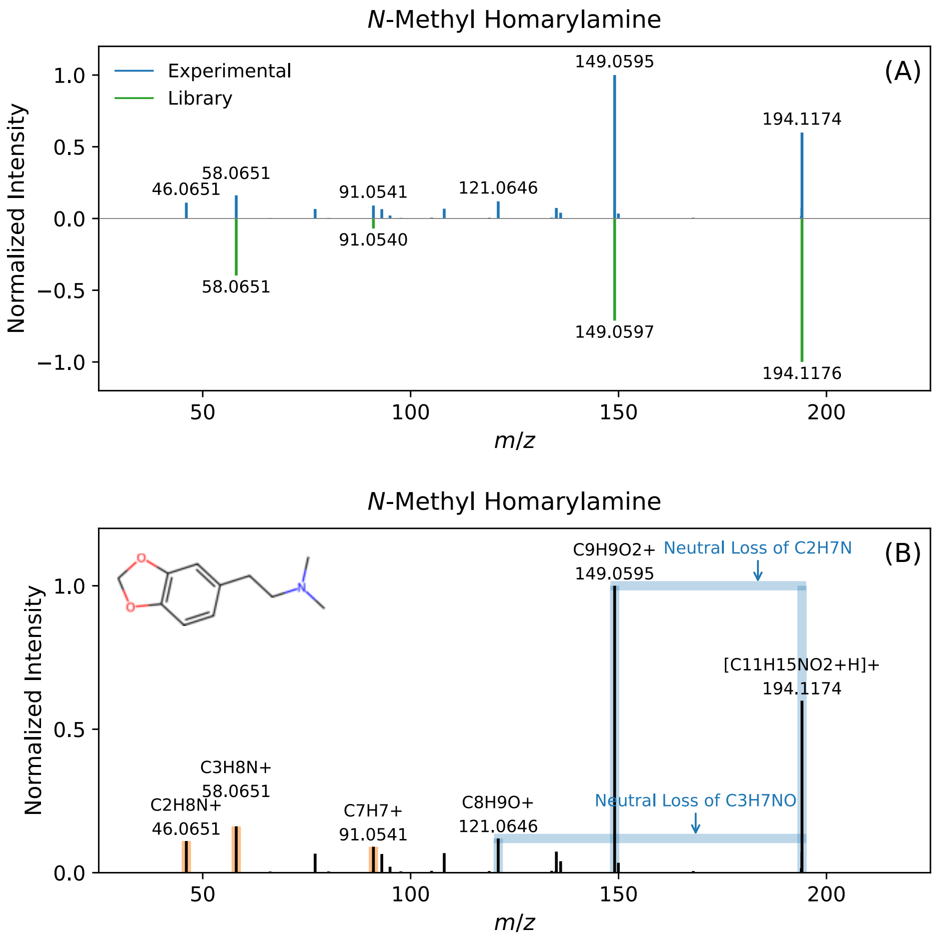

Figure S13. *N*-Methyl homarylamine assigned at confidence level 3 as tentative candidate: **(A)** Head-to-tail plots of experimental (top) and library (bottom) dd-MS2 spectra of *N*-methyl homarylamine. **(B)** Experimental dd-MS2 spectrum of *N*-methyl homarylamine highlighted with fragment ions (Table S28) and the neutral losses of C_2H_7N and C_3H_7NO.

Table S29. Fragment ions and neutral loss of 5-fluoro AMB-PICA (MMB-2201) assigned at confidence level 3 as tentative candidate							
Molecular Structure	Molecular Formula	ΔMass [ppm]	Exact Mass	Adduct	*m/z*	RT [min]	Area (Max.)
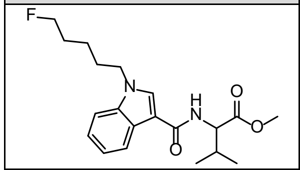	$C_{20}H_{27}FN_2O_3$	0.01	362.2006	[M+H]+	363.2078	17.17	1.41E+08
Neutral Loss	Formula	ΔMass [ppm]	Exact Mass	Neutral Loss	Formula	ΔMass [ppm]	Exact Mass
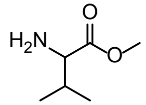	$C_6H_{13}NO_2$	-1.59	131.0946				
Fragment Ion	Formula	ΔMass [ppm]	*m/z*	Fragment Ion	Formula	ΔMass [ppm]	*m/z*
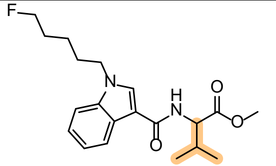	$[C_4H_8]+H+$	-1.22	57.0699	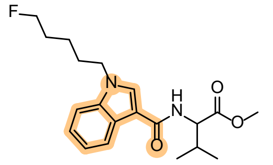	$[C_9H_5NO]+H+$	-2.71	144.0440
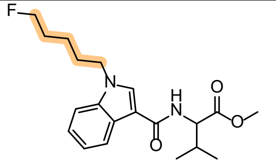	$[C_5H_{10}-H]+$	2.90	69.0704	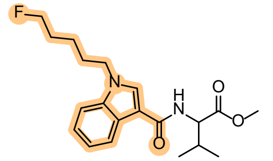	$[C_{14}H_{15}FNO]+$	-1.42	232.1130

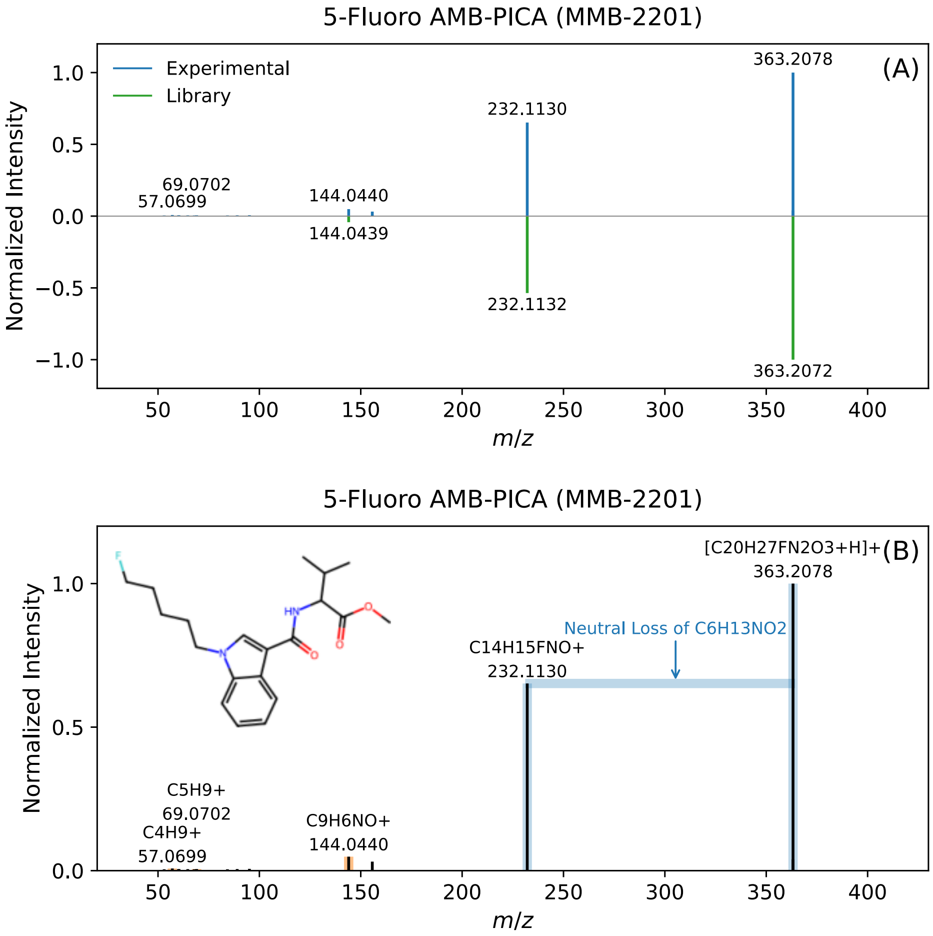

Figure S14. 5-Fluoro AMB-PICA (MMB-2201) assigned at confidence level 3 as tentative candidate: **(A)** Head-to-tail plots of experimental (top) and library (bottom) dd-MS2 spectra of 5-fluoro AMB-PICA. **(B)** Experimental dd-MS2 spectrum of 5-fluoro AMB-PICA highlighted with fragment ions (Table S29) and the neutral loss of $C_6H_{13}NO_2$.

S7. Population normalization factors

Table S30. Summary of population normalization factors reported by WBE studies

NH_3-N (g/day/person)	BOD_5 (g/day/person)	$CBOD_5$ (g/day/person)	TKN (g/day/person)	TP (g/day/person)	Caffeine (mg/d/person)	Sucralose (mg/d/person)	References
8.8±1.3	67±10	60±9	12.2±2.1	-	-	-	This work
-	60	-	-	-	-	-	[32]
-	59	-	12.5	1.7	-	-	[68]
8.1±0.4	-	-	-	-	-	-	[69]
6±2	-	-	-	-	-	-	[70]
9.46	-	-	-	1.02	-	-	[71]
9.5	-	-	-	-	-	-	[72]
7.5±0.3	22±4.2	-	10.6±0.7	0.79±0.07	-	-	[73]
9.74	-	-	12.91	0.94	-	-	[74]
7.2±0.7	-	66.1 (63.7 – 71.3)	12.8 (12.6 – 13.4)	-	-	-	[75]
-	50.7	-	10.6	1.24	-	-	[76]
-	-	-	-	-	165	-	[77]
-	-	-	-	-	224	-	[78]
-	-	-	-	-	-	18.5	[79]
-	-	-	-	-	-	26	[80]

NH_3-N = ammonia nitrogen; BOD_5 = 5-day biochemical oxygen demand; $CBOD_5$ = 5-day carbonaceous biochemical oxygen demand; TKN = total Kjeldahl nitrogen; TP = total phosphorus.

S8. Consumption rates of substances

Table S31. Summary of substance consumption rates reported by WBE studies in the U.S.

Substance [DTR]	Min (mg/d/1000 people)	Mean (mg/d/1000 people)	Median (mg/d/1000 people)	Max (mg/d/1000 people)	Reference
Fentanyl [Norfentanyl]	-	1.27±0.10	-	-	81
	4	-	14	191	82
	31	-	134	169	83
	2.1	-	2.4	17.6	84
	-	-	-	15	85
	7	49	37	230	86
	36	-	46	72	80
Codeine	-	140±127	-	-	81
	-	50	-	-	87
	102	-	341	4169	82
	50	-	52	192	83
	587	-	1307	2090	88
	-	-	240	-	65
	51	-	67	100	84
	-	188	-	275	85
	9	39	32	112	86
	54	73	68	112	80
Methadone [EDDP]	-	131±82	-	-	81
	-	72	-	-	87
	793	1387	1390	2200	89
	13	-	128	1080	88
	92	-	428	1410	83
	17	72	55	231	65
	37	126	109	316	84
	30	-	135	421	86
	170	377	361	617	80
Tramadol [*O*-Desmethyltramadol & *N*-Desmethyltramadol]	1330	-	-	2330	88
	190	937	713	3900	65
	2	1119	-	3250	85
	324	496	458	759	80
Diphenhydramine	-	9200	-	-	90
	1507	14637	13181	45903	65
Cocaine [Benzoylecgonine]	-	938±706	-	-	81
	287	1310	1032	3240	89
	-	551	-	-	87
	517	-	723	926	91
	363	1813	1515	3830	83
	104	-	-	488	88
	285	1853	1241	6053	65
	332	712	641	1260	84
	11	1648	-	8899	85
	102	-	721	2413	86
	339	1256	1274	2512	80
Methamphetamine	-	1740±1190	-	-	81
	1060	2510	2460	5600	89
	20	-	22	64	91
	1030	-	1160	1660	83
	984	-	-	6510	88
	20	158	116	503	65
	1360	2888	2890	4510	84
	-	3077	-	9519	85
	29	-	43	354	86
	1622	7031	6964	11749	80

Table S31. Summary of substance consumption rates reported by WBE studies in the U.S. (continued)					
Substance [DTR]	Min (mg/d/1000 people)	Mean (mg/d/1000 people)	Median (mg/d/1000 people)	Max (mg/d/1000 people)	Reference
Amphetamine	-	967±455	-	-	[81]
	350	741	649	1450	[89]
	-	256	-	-	[87]
	124	-	216	229	[91]
	344	-	400	738	[83]
	420	-	865	1710	[88]
	18	159	129	407	[65]
	452	670	691	879	[84]
	-	2078	-	8738	[85]
	32	-	555	1263	[86]
Nicotine [Cotinine & *trans*-3'-Hydroxycotinine]	2400	8044	3300	22500	[55]
	780	3200	-	4630	[85]
Δ^9-Tetrahydrocannabinol [THC-COOH & 11-OH-THC]	-	26900±8310	-	-	[81]
	35200	94120	75650	172000	[89]
	78700	-	-	88100	[83]
	24900	45200	44900	60600	[84]
	289000	1587000	-	2615000	[85]
	34674	65880	60320	120226	[80]
Caffeine [Paraxanthine]	33932	131573	109907	471991	[65]
	148000	349000	-	693000	[85]
	691831	998235	944123	1778279	[80]
Sucralose	15400	-	-	21600	[79]
	3236	14854	13950	44086	[65]
	16218	28740	24833	47863	[80]

**Table S32.** Consumption rates of the 12 most frequently detected target substances by sewershed				
Substance [DTR]	Min (mg/d/1000 people)	Mean (mg/d/1000 people)	Median (mg/d/1000 people)	Max (mg/d/1000 people)
**Sewershed A**				
Fentanyl [Norfentanyl]	75	136	128	305
Codeine	44	87	75	168
Methadone [EDDP]	169	397	342	712
Tramadol [*O*-Desmethyltramadol & *N*-Desmethyltramadol]	323	1056	979	3086
Diphenhydramine	3546	18384	17568	40233
Cocaine [Benzoylecgonine]	1516	4605	4122	13386
Methamphetamine	282	554	531	1099
Amphetamine	499	815	771	1675
Nicotine [Cotinine & *trans*-3'-Hydroxycotinine]	1544	3347	3187	7289
Δ^9-Tetrahydrocannabinol [THC-COOH & 11-OH-THC]	1662	15250	11687	54183
Caffeine [Paraxanthine]	55798	124574	117919	264642
Sucralose	11575	25337	21265	78213
**Sewershed B**				
Fentanyl [Norfentanyl]	28	56	50	118
Codeine	17	121	95	634
Methadone [EDDP]	63	189	136	641
Tramadol [*O*-Desmethyltramadol & *N*-Desmethyltramadol]	228	927	877	1999
Diphenhydramine	7653	22071	21221	55832
Cocaine [Benzoylecgonine]	682	1530	1458	3876
Methamphetamine	103	269	250	555
Amphetamine	225	600	556	1515
Nicotine [Cotinine & *trans*-3'-Hydroxycotinine]	991	2184	2097	5838
Δ^9-Tetrahydrocannabinol [THC-COOH & 11-OH-THC]	970	5999	4747	18987
Caffeine [Paraxanthine]	44638	111485	106721	268316
Sucralose	6749	25515	23431	71574
**Sewershed C**				
Fentanyl [Norfentanyl]	26	59	53	139
Codeine	23	106	68	409
Methadone [EDDP]	90	166	152	358
Tramadol [*O*-Desmethyltramadol & *N*-Desmethyltramadol]	476	956	780	4734
Diphenhydramine	13483	23572	22020	42132
Cocaine [Benzoylecgonine]	705	1425	1341	2990
Methamphetamine	118	270	254	573
Amphetamine	247	667	607	1432
Nicotine [Cotinine & *trans*-3'-Hydroxycotinine]	1594	2646	2489	5728
Δ^9-Tetrahydrocannabinol [THC-COOH & 11-OH-THC]	2000	7160	5891	23264
Caffeine [Paraxanthine]	62161	140243	131733	294095
Sucralose	11896	21182	20142	37921
**Sewershed D**				
Fentanyl [Norfentanyl]	28	59	47	169
Codeine	27	99	70	384
Methadone [EDDP]	82	221	150	730
Tramadol [*O*-Desmethyltramadol & *N*-Desmethyltramadol]	181	986	961	2560
Diphenhydramine	7111	24107	23559	51363
Cocaine [Benzoylecgonine]	601	1327	1167	3092
Methamphetamine	364	942	830	2983
Amphetamine	282	693	612	2115
Nicotine [Cotinine & *trans*-3'-Hydroxycotinine]	974	2636	2464	6084
Δ^9-Tetrahydrocannabinol [THC-COOH & 11-OH-THC]	681	4555	4281	9573
Caffeine [Paraxanthine]	47209	125516	111415	324998
Sucralose	7187	26852	25321	74542

**Table S32.** Consumption rates of the 12 most frequently detected target substances by sewershed (continued)				
Substance [DTR]	Min (mg/d/1000 people)	Mean (mg/d/1000 people)	Median (mg/d/1000 people)	Max (mg/d/1000 people)
**Sewershed E**				
Fentanyl [Norfentanyl]	26	69	67	155
Codeine	15	101	67	983
Methadone [EDDP]	107	281	231	670
Tramadol [*O*-Desmethyltramadol & *N*-Desmethyltramadol]	226	994	956	2901
Diphenhydramine	8839	24936	24762	52474
Cocaine [Benzoylecgonine]	388	1472	1439	3381
Methamphetamine	126	383	400	863
Amphetamine	222	650	656	1395
Nicotine [Cotinine & *trans*-3'-Hydroxycotinine]	903	2748	2809	5907
Δ^9-Tetrahydrocannabinol [THC-COOH & 11-OH-THC]	1059	6088	5661	23090
Caffeine [Paraxanthine]	39616	158475	140202	412285
Sucralose	4902	30477	28472	73172
**Sewershed F**				
Fentanyl [Norfentanyl]	30	53	49	157
Codeine	26	64	54	198
Methadone [EDDP]	93	259	203	775
Tramadol [*O*-Desmethyltramadol & *N*-Desmethyltramadol]	368	909	851	2705
Diphenhydramine	14148	27791	26045	65559
Cocaine [Benzoylecgonine]	400	905	774	1853
Methamphetamine	107	210	197	541
Amphetamine	435	775	686	2081
Nicotine [Cotinine & *trans*-3'-Hydroxycotinine]	993	1718	1642	4862
Δ^9-Tetrahydrocannabinol [THC-COOH & 11-OH-THC]	770	4776	3790	11994
Caffeine [Paraxanthine]	63928	147024	128219	444313
Sucralose	8434	17924	16644	54043
**Sewershed G**				
Fentanyl [Norfentanyl]	63	101	78	179
Codeine	48	111	108	198
Methadone [EDDP]	186	395	269	970
Tramadol [*O*-Desmethyltramadol & *N*-Desmethyltramadol]	631	1047	1013	1810
Diphenhydramine	13389	25345	30116	40401
Cocaine [Benzoylecgonine]	1199	2996	3222	4087
Methamphetamine	2102	4963	5508	7247
Amphetamine	579	1221	1316	1655
Nicotine [Cotinine & *trans*-3'-Hydroxycotinine]	2245	4433	4646	6628
Δ^9-Tetrahydrocannabinol [THC-COOH & 11-OH-THC]	7326	39316	12197	127263
Caffeine [Paraxanthine]	73631	169520	185951	255671
Sucralose	11200	36321	26710	79629
**Sewershed H**				
Fentanyl [Norfentanyl]	73	94	96	107
Codeine	783	783	783	783
Methadone [EDDP]	184	251	245	311
Tramadol [*O*-Desmethyltramadol & *N*-Desmethyltramadol]	757	1350	1424	1927
Diphenhydramine	5670	12043	12029	17290
Cocaine [Benzoylecgonine]	1384	3689	3751	6260
Methamphetamine	387	685	640	1125
Amphetamine	307	518	478	828
Nicotine [Cotinine & *trans*-3'-Hydroxycotinine]	394	1511	1502	2629
Δ^9-Tetrahydrocannabinol [THC-COOH & 11-OH-THC]	6123	11283	8563	27850
Caffeine [Paraxanthine]	45594	89790	76258	209596
Sucralose	10903	18639	16403	37685

Table S32. Consumption rates of the 12 most frequently detected target substances by sewershed (continued)				
Substance [DTR]	Min (mg/d/1000 people)	Mean (mg/d/1000 people)	Median (mg/d/1000 people)	Max (mg/d/1000 people)
**Sewershed I**				
Fentanyl [Norfentanyl]	89	125	131	136
Codeine	65	138	88	364
Methadone [EDDP]	205	240	215	390
Tramadol [*O*-Desmethyltramadol & *N*-Desmethyltramadol]	820	1551	1413	2918
Diphenhydramine	13163	29613	32363	37848
Cocaine [Benzoylecgonine]	1349	3158	3096	4772
Methamphetamine	1094	2165	2132	4597
Amphetamine	776	1336	1340	2347
Nicotine [Cotinine & *trans*-3'-Hydroxycotinine]	2191	3853	3989	5643
Δ^9-Tetrahydrocannabinol [THC-COOH & 11-OH-THC]	10006	28062	24802	53962
Caffeine [Paraxanthine]	128246	299877	342786	410931
Sucralose	15309	37962	39608	60760
**Sewershed J**				
Fentanyl [Norfentanyl]	53	77	78	107
Codeine	41	157	112	344
Methadone [EDDP]	115	126	122	166
Tramadol [*O*-Desmethyltramadol & *N*-Desmethyltramadol]	429	666	605	974
Diphenhydramine	8915	19563	15430	35375
Cocaine [Benzoylecgonine]	2073	6349	5138	12370
Methamphetamine	710	1308	1373	2307
Amphetamine	443	739	639	1273
Nicotine [Cotinine & *trans*-3'-Hydroxycotinine]	1495	2967	2035	5655
Δ^9-Tetrahydrocannabinol [THC-COOH & 11-OH-THC]	11812	23086	19419	52188
Caffeine [Paraxanthine]	50992	99501	87875	153353
Sucralose	9556	23542	20830	45117

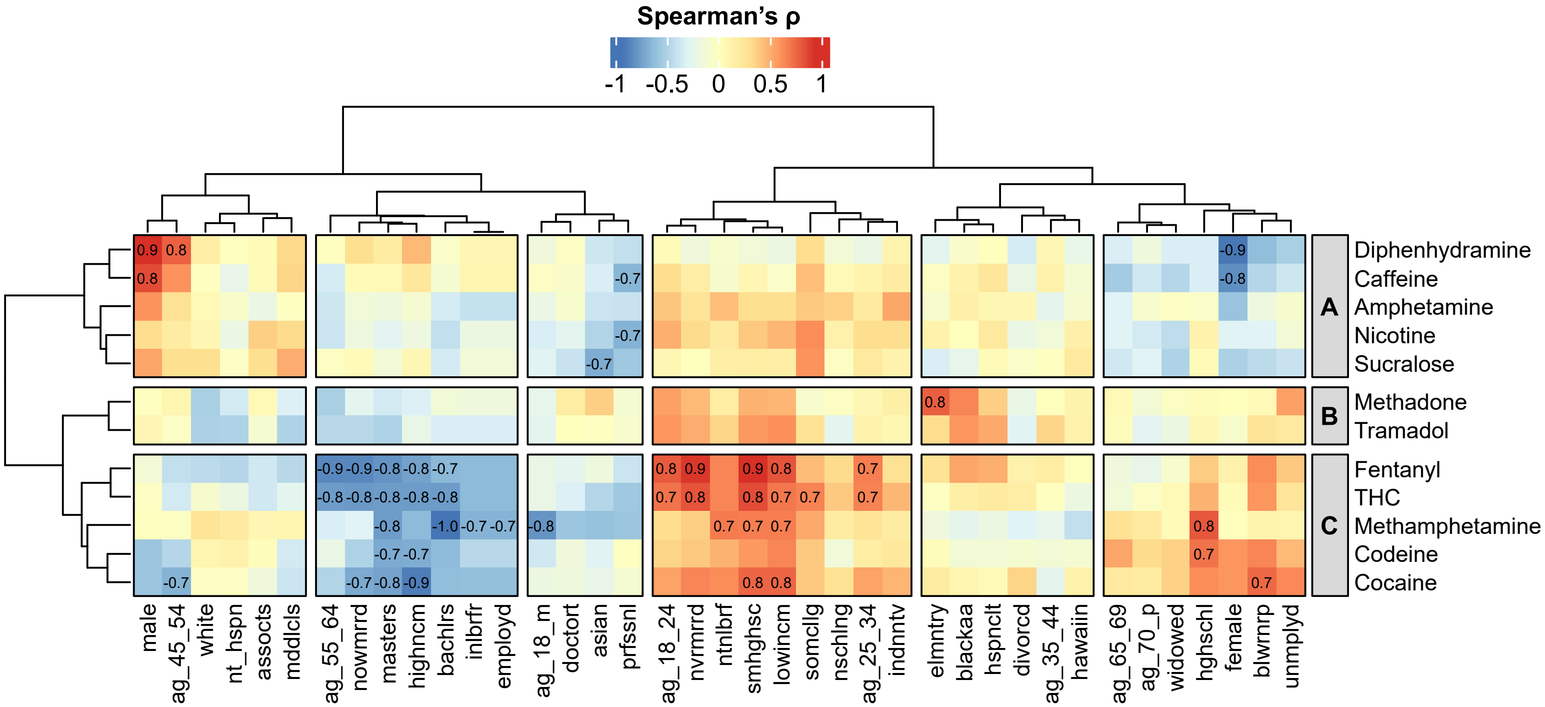

Figure S15. Hierarchical clustering of the Spearman's rank correlation coefficients (ρ) between the consumption rates of 12 target substances (Table S32) and the sociodemographic attributes of sewershed populations (Table S2) based on Ward's method and Euclidean distance. The color scale (red to blue) measures Spearman's ρ values. The column annotations correspond to five-year estimates and corresponding margins of error for gender, age groups, race, ethnicity, marital status, educational attainment, household income, and employment status sourced from the ACS 2017-2021 5-Year Data[2] at the census block-group level and intersected with the sewersheds.

S9. Population-normalized mass load (PNML) ratios for substances

Table S33. PNML ratios of the 12 most frequently detected target substances by sewershed

PNML Ratio	Mean ± Std	Median
**Sewershed A**		
Fentanyl : Norfentanyl	0.20±0.03	0.19
Methadone : EDDP	0.59±0.14	0.64
Tramadol: *O*-Desmethyltramadol	1.85±0.61	1.71
Cocaine : Benzoylecgonine	0.55±0.10	0.58
Nicotine : Cotinine	3.94±0.44	3.84
Caffeine : Paraxanthine	1.55±0.14	1.53
Amphetamine : Methamphetamine[a]	1.61±0.18	1.61
THC-COOH : 11-OH-THC[a]	2.44±0.87	2.35
**Sewershed B**		
Fentanyl : Norfentanyl	0.19±0.03	0.19
Methadone : EDDP	0.55±0.11	0.54
Tramadol: *O*-Desmethyltramadol	2.17±0.69	2.21
Cocaine : Benzoylecgonine	0.51±0.15	0.54
Nicotine : Cotinine	2.50±0.33	2.50
Caffeine : Paraxanthine	1.42±0.09	1.41
Amphetamine : Methamphetamine[a]	3.26±1.16	3.59
THC-COOH : 11-OH-THC[a]	2.44±0.39	2.48
**Sewershed C**		
Fentanyl : Norfentanyl	0.18±0.03	0.18
Methadone : EDDP	0.53±0.15	0.52
Tramadol: *O*-Desmethyltramadol	2.57±0.76	2.65
Cocaine : Benzoylecgonine	0.43±0.13	0.41
Nicotine : Cotinine	3.02±0.36	2.97
Caffeine : Paraxanthine	1.40±0.18	1.34
Amphetamine : Methamphetamine[a]	2.68±0.38	2.66
THC-COOH : 11-OH-THC[a]	3.14±1.20	3.17
**Sewershed D**		
Fentanyl : Norfentanyl	0.22±0.04	0.23
Methadone : EDDP	0.64±0.13	0.67
Tramadol: *O*-Desmethyltramadol	2.81±1.05	2.88
Cocaine : Benzoylecgonine	0.45±0.14	0.42
Nicotine : Cotinine	2.48±0.28	2.45
Caffeine : Paraxanthine	1.39±0.11	1.40
Amphetamine : Methamphetamine[a]	0.81±0.08	0.80
THC-COOH : 11-OH-THC[a]	2.28±1.22	2.51
**Sewershed E**		
Fentanyl : Norfentanyl	0.19±0.03	0.17
Methadone : EDDP	0.48±0.12	0.45
Tramadol: *O*-Desmethyltramadol	2.23±0.69	2.27
Cocaine : Benzoylecgonine	0.51±0.15	0.50
Nicotine : Cotinine	2.39±0.36	2.38
Caffeine : Paraxanthine	1.54±0.20	1.52
Amphetamine : Methamphetamine[a]	1.85±0.22	1.87
THC-COOH : 11-OH-THC[a]	3.00±1.31	3.26

**Table S33.** PNML ratios of the 12 most frequently detected target substances by sewershed (continued)		
PNML Ratio	Mean ± Std	Median
**Sewershed F**		
Fentanyl : Norfentanyl	0.23±0.04	0.23
Methadone : EDDP	0.46±0.13	0.43
Tramadol: *O*-Desmethyltramadol	2.58±0.79	2.47
Cocaine : Benzoylecgonine	0.54±0.13	0.55
Nicotine : Cotinine	4.17±0.71	4.11
Caffeine : Paraxanthine	1.41±0.14	1.40
Amphetamine : Methamphetamine[a]	4.06±0.55	3.85
THC-COOH : 11-OH-THC[a]	2.27±0.92	2.04
**Sewershed G**		
Fentanyl : Norfentanyl	0.24±0.04	0.26
Methadone : EDDP	0.59±0.15	0.63
Tramadol: *O*-Desmethyltramadol	2.12±0.94	2.14
Cocaine : Benzoylecgonine	0.49±0.09	0.51
Nicotine : Cotinine	2.60±0.21	2.65
Caffeine : Paraxanthine	1.65±0.12	1.72
Amphetamine : Methamphetamine[a]	0.27±0.03	0.28
THC-COOH : 11-OH-THC[a]	2.32±0.88	2.25
**Sewershed H**		
Fentanyl : Norfentanyl	0.22±0.03	0.22
Methadone : EDDP	0.53±0.07	0.52
Tramadol: *O*-Desmethyltramadol	1.46±0.41	1.35
Cocaine : Benzoylecgonine	0.41±0.08	0.39
Nicotine : Cotinine	2.65±0.19	2.63
Caffeine : Paraxanthine	1.51±0.24	1.44
Amphetamine : Methamphetamine[a]	0.83±0.10	0.80
THC-COOH : 11-OH-THC[a]	3.77±0.79	3.71
**Sewershed I**		
Fentanyl : Norfentanyl	0.21±0.04	0.19
Methadone : EDDP	0.50±0.05	0.49
Tramadol: *O*-Desmethyltramadol	1.33±0.17	1.35
Cocaine : Benzoylecgonine	0.64±0.10	0.68
Nicotine : Cotinine	4.03±0.27	4.08
Caffeine : Paraxanthine	1.63±0.22	1.61
Amphetamine : Methamphetamine[a]	0.71±0.13	0.75
THC-COOH : 11-OH-THC[a]	2.51±1.24	2.04
**Sewershed J**		
Fentanyl : Norfentanyl	0.22±0.04	0.20
Methadone : EDDP	0.47±0.01	0.46
Tramadol: *O*-Desmethyltramadol	2.89±0.65	2.95
Cocaine : Benzoylecgonine	0.50±0.14	0.47
Nicotine : Cotinine	2.80±0.34	2.76
Caffeine : Paraxanthine	1.51±0.12	1.53
Amphetamine : Methamphetamine[a]	0.63±0.14	0.63
THC-COOH : 11-OH-THC[a]	3.57±0.70	3.57

[a] Not a parent-to-metabolite ratio but calculated for comparison with the literature.

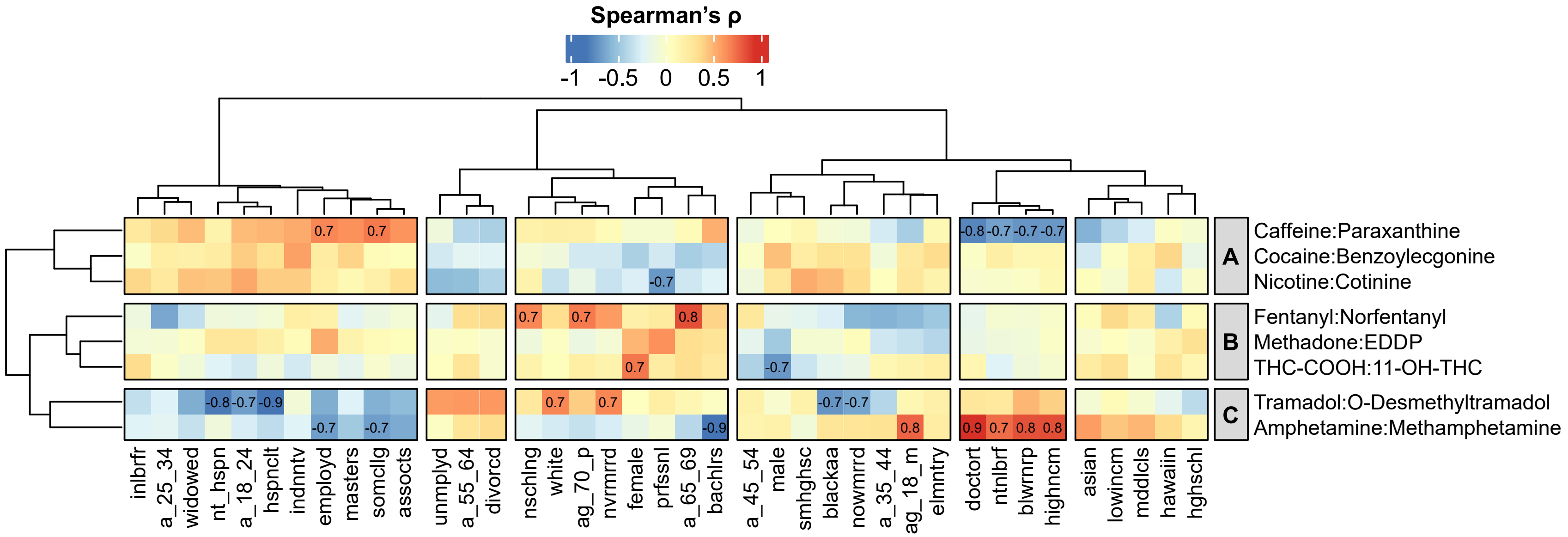

Figure S16. Hierarchical clustering of the Spearman's rank correlation coefficients (ρ) between the population-normalized mass load (PNML) ratios for structurally related target substances (Table S33) and the sociodemographic attributes of sewershed populations (Table S2) based on Ward's method and Euclidean distance. The color scale (red to blue) measures Spearman's ρ values. The column annotations correspond to five-year estimates and corresponding margins of error for gender, age groups, race, ethnicity, marital status, educational attainment, household income, and employment status sourced from the ACS 2017-2021 5-Year Data[2] at the census block-group level and intersected with the sewersheds.

References

(1) Kapo, K. E.; Paschka, M.; Vamshi, R.; Sebasky, M.; McDonough, K. Estimation of U.S. sewer residence time distributions for national-scale risk assessment of down-the-drain chemicals. *Science of the Total Environment* **2017**, *603-604*, 445-452.
(2) U.S. Census Bureau. *American Community Survey 2017-2021 5-Year Data*. U.S. Census Bureau, Washington, D.C., https://www.census.gov/programs-surveys/acs (accessed March 31, 2024).
(3) New York State Department of Health. *New York State Opioid Data Dashboard*. New York State Department of Health, Albany, NY, 2023. https://www.health.ny.gov/statistics/opioid/ (accessed March 31, 2024).
(4) New York State Department of Health. *Expanded Behavioral Risk Factor Surveillance System*. New York State Department of Health, Albany, NY, 2023. https://www.health.ny.gov/statistics/brfss/expanded/ (accessed March 31, 2024).
(5) Baker, D. R.; Kasprzyk-Hordern, B. Critical evaluation of methodology commonly used in sample collection, storage and preparation for the analysis of pharmaceuticals and illicit drugs in surface water and wastewater by solid phase extraction and liquid chromatography–mass spectrometry. *Journal of Chromatography A* **2011**, *1218* (44), 8036-8059.
(6) Pagsuyoin, S. A.; Luo, J.; Chain, F. J. Effects of sewer biofilm on the degradation of drugs in sewage: A microcosm study. *Journal of Hazardous Materials* **2022**, *424*, 127666.
(7) Ahmed, F.; Li, J.; O'Brien, J. W.; Tscharke, B. J.; Samanipour, S.; Thai, P. K.; Yuan, Z.; Mueller, J. F.; Thomas, K. V. In-sewer stability of selected analgesics and their metabolites. *Water Research* **2021**, *204*, 117647.
(8) Chen, C.; Kostakis, C.; Irvine, R. J.; Felgate, P. D.; White, J. M. Evaluation of pre-analysis loss of dependent drugs in wastewater: stability and binding assessments. *Drug Testing and Analysis* **2013**, *5* (8), 716-721.
(9) Senta, I.; Krizman, I.; Ahel, M.; Terzic, S. Assessment of stability of drug biomarkers in municipal wastewater as a factor influencing the estimation of drug consumption using sewage epidemiology. *Science of the Total Environment* **2014**, *487*, 659-665.
(10) Östman, M.; Fick, J.; Näsström, E.; Lindberg, R. H. A snapshot of illicit drug use in Sweden acquired through sewage water analysis. *Science of the Total Environment* **2014**, *472*, 862-871.
(11) Ramin, P.; Libonati Brock, A.; Polesel, F.; Causanilles, A.; Emke, E.; de Voogt, P.; Plósz, B. G. Transformation and sorption of illicit drug biomarkers in sewer systems: Understanding the role of suspended solids in raw wastewater. *Environmental Science & Technology* **2016**, *50* (24), 13397-13408.
(12) Gao, J.; Banks, A.; Li, J.; Jiang, G.; Lai, F. Y.; Mueller, J. F.; Thai, P. K. Evaluation of in-sewer transformation of selected illicit drugs and pharmaceutical biomarkers. *Science of the Total Environment* **2017**, *609*, 1172-1181.
(13) O'Brien, J. W.; Banks, A. P. W.; Novic, A. J.; Mueller, J. F.; Jiang, G.; Ort, C.; Eaglesham, G.; Yuan, Z.; Thai, P. K. Impact of in-sewer degradation of pharmaceutical and personal care products (PPCPs) population markers on a population model. *Environmental Science & Technology* **2017**, *51* (7), 3816-3823.
(14) Li, J.; Gao, J.; Thai, P. K.; Sun, X.; Mueller, J. F.; Yuan, Z.; Jiang, G. Stability of illicit drugs as biomarkers in sewers: From lab to reality. *Environmental Science & Technology* **2018**, *52* (3), 1561-1570.
(15) Gao, J.; Li, J.; Jiang, G.; Shypanski, A. H.; Nieradzik, L. M.; Yuan, Z.; Mueller, J. F.; Ort, C.; Thai, P. K. Systematic evaluation of biomarker stability in pilot scale sewer pipes. *Water Research* **2019**, *151*, 447-455.
(16) Choi, P. M.; Li, J.; Gao, J.; O'Brien, J. W.; Thomas, K. V.; Thai, P. K.; Jiang, G.; Mueller, J. F. Considerations for assessing stability of wastewater-based epidemiology biomarkers using biofilm-free and sewer reactor tests. *Science of the Total Environment* **2020**, *709*, 136228.
(17) González-Mariño, I.; Quintana, J. B.; Rodríguez, I.; Cela, R. Determination of drugs of abuse in water by solid-phase extraction, derivatisation and gas chromatography–ion trap-tandem mass spectrometry. *Journal of Chromatography A* **2010**, *1217* (11), 1748-1760.

(18) Li, J.; Gao, J.; Thai, P. K.; Shypanski, A.; Nieradzik, L.; Mueller, J. F.; Yuan, Z.; Jiang, G. Experimental investigation and modeling of the transformation of illicit drugs in a pilot-scale sewer system. *Environmental Science & Technology* **2019**, *53* (8), 4556-4565.
(19) van Nuijs, A. L. N.; Abdellati, K.; Bervoets, L.; Blust, R.; Jorens, P. G.; Neels, H.; Covaci, A. The stability of illicit drugs and metabolites in wastewater, an important issue for sewage epidemiology? *Journal of Hazardous Materials* **2012**, *239-240*, 19-23.
(20) Bisceglia, K. J.; Lippa, K. A. Stability of cocaine and its metabolites in municipal wastewater – the case for using metabolite consolidation to monitor cocaine utilization. *Environmental Science and Pollution Research* **2014**, *21* (6), 4453-4460.
(21) Thai, P. K.; Jiang, G.; Gernjak, W.; Yuan, Z.; Lai, F. Y.; Mueller, J. F. Effects of sewer conditions on the degradation of selected illicit drug residues in wastewater. *Water Research* **2014**, *48* (0), 538-547.
(22) McCall, A.-K.; Palmitessa, R.; Blumensaat, F.; Morgenroth, E.; Ort, C. Modeling in-sewer transformations at catchment scale – implications on drug consumption estimates in wastewater-based epidemiology. *Water Research* **2017**, *122*, 655-668.
(23) Liu, A.; Lin, W.; Ming, R.; Guan, W.; Wang, X.; Hu, N.; Ren, Y. Stability of 28 typical prescription drugs in sewer systems and interaction with the biofilm bacterial community. *Journal of Hazardous Materials* **2022**, *436*, 129142.
(24) Castiglioni, S.; Bagnati, R.; Melis, M.; Panawennage, D.; Chiarelli, P.; Fanelli, R.; Zuccato, E. Identification of cocaine and its metabolites in urban wastewater and comparison with the human excretion profile in urine. *Water Research* **2011**, *45* (16), 5141-5150.
(25) Mardal, M.; Kinyua, J.; Ramin, P.; Miserez, B.; Van Nuijs, A. L. N.; Covaci, A.; Meyer, M. R. Screening for illicit drugs in pooled human urine and urinated soil samples and studies on the stability of urinary excretion products of cocaine, MDMA, and MDEA in wastewater by hyphenated mass spectrometry techniques. *Drug Testing and Analysis* **2017**, *9* (1), 106-114.
(26) Banks, A. P. W.; Lai, F. Y.; Mueller, J. F.; Jiang, G.; Carter, S.; Thai, P. K. Potential impact of the sewer system on the applicability of alcohol and tobacco biomarkers in wastewater-based epidemiology. *Drug Testing and Analysis* **2018**, *10* (3), 530-538.
(27) Tscharke, B. J.; White, J. M.; Gerber, J. P. Estimates of tobacco use by wastewater analysis of anabasine and anatabine. *Drug Testing and Analysis* **2016**, *8* (7), 702-707.
(28) Gao, J.; Li, J.; Jiang, G.; Yuan, Z.; Eaglesham, G.; Covaci, A.; Mueller, J. F.; Thai, P. K. Stability of alcohol and tobacco consumption biomarkers in a real rising main sewer. *Water Research* **2018**, *138*, 19-26.
(29) Zheng, Q.; Eaglesham, G.; Tscharke, B. J.; O'Brien, J. W.; Li, J.; Thompson, J.; Shimko, K. M.; Reeks, T.; Gerber, C.; Thomas, K. V.; Thai, P. K. Determination of anabasine, anatabine, and nicotine biomarkers in wastewater by enhanced direct injection LC-MS/MS and evaluation of their in-sewer stability. *Science of the Total Environment* **2020**, *743*, 140551.
(30) Ordóñez, E. Y.; Quintana, J. B.; Rodil, R.; Cela, R. Determination of artificial sweeteners in water samples by solid-phase extraction and liquid chromatography–tandem mass spectrometry. *Journal of Chromatography A* **2012**, *1256*, 197-205.
(31) Baker, D. R.; Kasprzyk-Hordern, B. Multi-residue determination of the sorption of illicit drugs and pharmaceuticals to wastewater suspended particulate matter using pressurised liquid extraction, solid phase extraction and liquid chromatography coupled with tandem mass spectrometry. *Journal of Chromatography A* **2011**, *1218* (44), 7901-7913.
(32) Baker, D. R.; Očenášková, V.; Kvicalova, M.; Kasprzyk-Hordern, B. Drugs of abuse in wastewater and suspended particulate matter — Further developments in sewage epidemiology. *Environment International* **2012**, *48*, 28-38.
(33) Senta, I.; Krizman, I.; Ahel, M.; Terzic, S. Integrated procedure for multiresidue analysis of dissolved and particulate drugs in municipal wastewater by liquid chromatography–tandem mass spectrometry. *Analytical and Bioanalytical Chemistry* **2013**, *405* (10), 3255-3268.

(34) Subedi, B.; Kannan, K. Mass loading and removal of select illicit drugs in two wastewater treatment plants in New York State and estimation of illicit drug usage in communities through wastewater analysis. *Environmental Science & Technology* **2014**, *48* (12), 6661-6670.
(35) Metcalfe, C.; Tindale, K.; Li, H.; Rodayan, A.; Yargeau, V. Illicit drugs in Canadian municipal wastewater and estimates of community drug use. *Environmental Pollution* **2010**, *158* (10), 3179-3185.
(36) Pandopulos, A. J.; Simpson, B. S.; White, J. M.; Bade, R.; Gerber, C. Partitioning of phytocannabinoids between faeces and water – Implications for wastewater-based epidemiology. *Science of the Total Environment* **2022**, *805*, 150269.
(37) Baker, D. R.; Barron, L.; Kasprzyk-Hordern, B. Illicit and pharmaceutical drug consumption estimated via wastewater analysis. Part A: Chemical analysis and drug use estimates. *Science of the Total Environment* **2014**, *487*, 629-641.
(38) Dave, R. A.; Morris, M. E. A quantitative threshold for high/low extent of urinary excretion of compounds in humans. *Biopharmaceutics & Drug Disposition* **2016**, *37* (5), 287-309.
(39) Ahmed, F.; Tscharke, B.; O'Brien, J. W.; Hall, W. D.; Cabot, P. J.; Sowa, P. M.; Samanipour, S.; Thomas, K. V. National wastewater reconnaissance of analgesic consumption in Australia. *Environmental Science & Technology* **2023**, *57* (4), 1712-1720.
(40) Depriest, A. Z.; Puet, B. L.; Holt, A. C.; Roberts, A.; Cone, E. J. Metabolism and disposition of prescription opioids: A review. *Forensic Science Review* **2015**, *27*, 115-145.
(41) Thai, P. K.; Lai, F. Y.; Bruno, R.; van Dyken, E.; Hall, W.; O'Brien, J.; Prichard, J.; Mueller, J. F. Refining the excretion factors of methadone and codeine for wastewater analysis - Combining data from pharmacokinetic and wastewater studies. *Environment International* **2016**, *94*, 307-314.
(42) Thai, P. K.; O'Brien, J. W.; Tscharke, B. J.; Mueller, J. F. Analyzing wastewater samples collected during Census to determine the correction factors of drugs for wastewater-based epidemiology: The case of codeine and methadone. *Environmental Science & Technology Letters* **2019**, *6* (5), 265-269.
(43) Cone, E. J.; Heltsley, R.; Black, D. L.; Mitchell, J. M.; LoDico, C. P.; Flegel, R. R. Prescription opioids. II. Metabolism and excretion patterns of hydrocodone in urine following controlled single-dose administration. *Journal of Analytical Toxicology* **2013**, *37* (8), 486-494.
(44) Been, F.; Benaglia, L.; Lucia, S.; Gervasoni, J.-P.; Esseiva, P.; Delémont, O. Data triangulation in the context of opioids monitoring via wastewater analyses. *Drug and Alcohol Dependence* **2015**, *151*, 203-210.
(45) Postigo, C.; Lopez de Alda, M. J.; Barceló, D. Analysis of drugs of abuse and their human metabolites in water by LC-MS2: A non-intrusive tool for drug abuse estimation at the community level. *TrAC Trends in Analytical Chemistry* **2008**, *27* (11), 1053-1069.
(46) Dong, Z.; Senn, D. B.; Moran, R. E.; Shine, J. P. Prioritizing environmental risk of prescription pharmaceuticals. *Regulatory Toxicology and Pharmacology* **2013**, *65* (1), 60-67.
(47) Thomaidis, N. S.; Gago-Ferrero, P.; Ort, C.; Maragou, N. C.; Alygizakis, N. A.; Borova, V. L.; Dasenaki, M. E. Reflection of socio-economic changes in wastewater: Licit and illicit drug use patterns. *Environmental Science & Technology* **2016**, *50* (18), 10065-10072.
(48) Postigo, C.; de Alda, M. L.; Barceló, D. Evaluation of drugs of abuse use and trends in a prison through wastewater analysis. *Environment International* **2011**, *37* (1), 49-55.
(49) Castiglioni, S.; Bijlsma, L.; Covaci, A.; Emke, E.; Hernández, F.; Reid, M.; Ort, C.; Thomas, K. V.; van Nuijs, A. L. N.; de Voogt, P.; Zuccato, E. Evaluation of uncertainties associated with the determination of community drug use through the measurement of sewage drug biomarkers. *Environmental Science & Technology* **2013**, *47* (3), 1452-1460.
(50) Been, F.; Bijlsma, L.; Benaglia, L.; Berset, J.-D.; Botero-Coy, A. M.; Castiglioni, S.; Kraus, L.; Zobel, F.; Schaub, M. P.; Bücheli, A.; Hernández, F.; Delémont, O.; Esseiva, P.; Ort, C. Assessing geographical differences in illicit drug consumption—A comparison of results from epidemiological and wastewater data in Germany and Switzerland. *Drug and Alcohol Dependence* **2016**, *161*, 189-199.
(51) van Nuijs, A. L. N.; Mougel, J.-F.; Tarcomnicu, I.; Bervoets, L.; Blust, R.; Jorens, P. G.; Neels, H.; Covaci, A. Sewage epidemiology — A real-time approach to estimate the consumption of illicit drugs in Brussels, Belgium. *Environment International* **2011**, *37* (3), 612-621.

(52) Nefau, T.; Karolak, S.; Castillo, L.; Boireau, V.; Levi, Y. Presence of illicit drugs and metabolites in influents and effluents of 25 sewage water treatment plants and map of drug consumption in France. *Science of the Total Environment* **2013**, *461-462* (Supplement C), 712-722.
(53) Gracia-Lor, E.; Zuccato, E.; Castiglioni, S. Refining correction factors for back-calculation of illicit drug use. *Science of the Total Environment* **2016**, *573*, 1648-1659.
(54) Castiglioni, S.; Senta, I.; Borsotti, A.; Davoli, E.; Zuccato, E. A novel approach for monitoring tobacco use in local communities by wastewater analysis. *Tobacco Control* **2015**, *24* (1), 38-42.
(55) Chen, J.; Venkatesan, A. K.; Halden, R. U. Alcohol and nicotine consumption trends in three U.S. communities determined by wastewater-based epidemiology. *Science of the Total Environment* **2019**, *656*, 174-183.
(56) Wang, D. G.; Dong, Q. Q.; Du, J.; Yang, S.; Zhang, Y. J.; Na, G. S.; Ferguson, S. G.; Wang, Z.; Zheng, T. Using Monte Carlo simulation to assess variability and uncertainty of tobacco consumption in a city by sewage epidemiology. *BMJ Open* **2016**, *6* (2), e010583.
(57) Been, F.; Schneider, C.; Zobel, F.; Delémont, O.; Esseiva, P. Integrating environmental and self-report data to refine cannabis prevalence estimates in a major urban area of Switzerland. *International Journal of Drug Policy* **2016**, *36*, 33-42.
(58) Burgard, D. A.; Williams, J.; Westerman, D.; Rushing, R.; Carpenter, R.; LaRock, A.; Sadetsky, J.; Clarke, J.; Fryhle, H.; Pellman, M.; Banta-Green, C. J. Using wastewater-based analysis to monitor the effects of legalized retail sales on cannabis consumption in Washington State, USA. *Addiction* **2019**, *114* (9), 1582-1590.
(59) Gracia-Lor, E.; Rousis, N. I.; Zuccato, E.; Bade, R.; Baz-Lomba, J. A.; Castrignanò, E.; Causanilles, A.; Hernández, F.; Kasprzyk-Hordern, B.; Kinyua, J.; McCall, A.-K.; van Nuijs, A. L. N.; Plósz, B. G.; Ramin, P.; Ryu, Y.; Santos, M. M.; Thomas, K.; de Voogt, P.; Yang, Z.; Castiglioni, S. Estimation of caffeine intake from analysis of caffeine metabolites in wastewater. *Science of the Total Environment* **2017**, *609*, 1582-1588.
(60) Li, D.; Zheng, Q.; Thomas, K. V.; Dang, A. K.; Binh, V. N.; Anh, N. T. K.; Thai, P. K. Use of artificial sweeteners and caffeine in a population of Hanoi: An assessment by wastewater-based epidemiology. *Science of the Total Environment* **2023**, *868*, 161515.
(61) Chiaia, A. C.; Banta-Green, C.; Field, J. Eliminating solid phase extraction with large-volume injection LC/MS/MS: Analysis of illicit and legal drugs and human urine indicators in US wastewaters. *Environmental Science & Technology* **2008**, *42* (23), 8841-8848.
(62) Postigo, C.; Lopez de Alda, M. J.; Barceló, D. Fully automated determination in the low nanogram per liter level of different classes of drugs of abuse in sewage water by on-line solid-phase extraction-liquid chromatography-electrospray-tandem mass spectrometry. *Analytical Chemistry* **2008**, *80* (9), 3123-3134.
(63) Heuett, N. V.; Ramirez, C. E.; Fernandez, A.; Gardinali, P. R. Analysis of drugs of abuse by online SPE-LC high resolution mass spectrometry: Communal assessment of consumption. *Science of the Total Environment* **2015**, *511*, 319-330.
(64) López-García, E.; Mastroianni, N.; Postigo, C.; Barceló, D.; López de Alda, M. A fully automated approach for the analysis of 37 psychoactive substances in raw wastewater based on on-line solid phase extraction-liquid chromatography-tandem mass spectrometry. *Journal of Chromatography A* **2018**, *1576*, 80-89.
(65) Wang, S.; Green, H. C.; Wilder, M. L.; Du, Q.; Kmush, B. L.; Collins, M. B.; Larsen, D. A.; Zeng, T. High-throughput wastewater analysis for substance use assessment in central New York during the COVID-19 pandemic. *Environmental Science: Processes and Impacts* **2020**, *22* (11), 2147-2161.
(66) Senta, I.; Rodríguez-Mozaz, S.; Corominas, L.; Covaci, A.; Petrovic, M. Applicability of an on-line solid-phase extraction liquid chromatography – tandem mass spectrometry for the wastewater-based assessment of human exposure to chemicals from personal care and household products. *Science of the Total Environment* **2022**, *845*, 157309.
(67) New York State Department of Environmental Conservation. *Municipal Wastewater Treatment Plants*. New York State Department of Environmental Conservation, Albany, NY, https://data.ny.gov/Energy-Environment/Municipal-Wastewater-Treatment-Plants/rsuw-xxks (accessed March 31, 2024).

(68) Lopes, A.; Silva, N.; Bronze, M. R.; Ferreira, J.; Morais, J. Analysis of cocaine and nicotine metabolites in wastewater by liquid chromatography–tandem mass spectrometry. Cross abuse index patterns on a major community. *Science of the Total Environment* **2014**, *487*, 673-680.
(69) Been, F.; Rossi, L.; Ort, C.; Rudaz, S.; Delémont, O.; Esseiva, P. Population normalization with ammonium in wastewater-based epidemiology: Application to illicit drug monitoring. *Environmental Science & Technology* **2014**, *48* (14), 8162-8169.
(70) Zheng, Q.-D.; Lin, J.-G.; Pei, W.; Guo, M.-X.; Wang, Z.; Wang, D.-G. Estimating nicotine consumption in eight cities using sewage epidemiology based on ammonia nitrogen equivalent population. *Science of the Total Environment* **2017**, *590-591*, 226-232.
(71) Zhang, X.; Huang, R.; Li, P.; Ren, Y.; Gao, J.; Mueller, J. F.; Thai, P. K. Temporal profile of illicit drug consumption in Guangzhou, China monitored by wastewater-based epidemiology. *Environmental Science and Pollution Research* **2019**, *26* (23), 23593-23602.
(72) Zhang, Y.; Duan, L.; Wang, B.; Du, Y.; Cagnetta, G.; Huang, J.; Blaney, L.; Yu, G. Wastewater-based epidemiology in Beijing, China: Prevalence of antibiotic use in flu season and association of pharmaceuticals and personal care products with socioeconomic characteristics. *Environment International* **2019**, *125*, 152-160.
(73) Zhao, J.; Lu, J.; Zhao, H.; Yan, Y.; Dong, H.; Li, W. Illicit drugs and their metabolites in urban wastewater: Analysis, occurrence and consumption in Xinjiang, China. *Science of the Total Environment* **2022**, *852*, 158457.
(74) Duan, L.; Zhang, Y.; Wang, B.; Yu, G.; Gao, J.; Cagnetta, G.; Huang, C.; Zhai, N. Wastewater surveillance for 168 pharmaceuticals and metabolites in a WWTP: Occurrence, temporal variations and feasibility of metabolic biomarkers for intake estimation. *Water Research* **2022**, *216*, 118321.
(75) Hoar, C.; Li, Y.; Silverman, A. I. Assessment of commonly measured wastewater parameters to estimate sewershed populations for use in wastewater-based epidemiology: Insights into population dynamics in New York City during the COVID-19 pandemic. *ACS ES&T Water* **2022**, *2* (11), 2014-2024.
(76) Sim, W.; Park, S.; Ha, J.; Kim, D.; Oh, J.-E. Evaluation of population estimation methods for wastewater-based epidemiology in a metropolitan city. *Science of the Total Environment* **2023**, *857*, 159154.
(77) Mitchell, D. C.; Knight, C. A.; Hockenberry, J.; Teplansky, R.; Hartman, T. J. Beverage caffeine intakes in the U.S. *Food and Chemical Toxicology* **2014**, *63*, 136-142.
(78) Rodak, K.; Kokot, I.; Kratz, E. M. Caffeine as a factor influencing the functioning of the human body - Friend or foe? *Nutrients* **2021**, *13* (9), 3088.
(79) Subedi, B.; Kannan, K. Fate of artificial sweeteners in wastewater treatment plants in New York State, U.S.A. *Environmental Science & Technology* **2014**, *48* (23), 13668-13674.
(80) Gerrity, D.; Crank, K.; Oh, E. C.; Quinones, O.; Trenholm, R. A.; Vanderford, B. J. Wastewater surveillance of high risk substances in Southern Nevada: Sucralose normalization to translate data for potential public health action. *Science of the Total Environment* **2024**, *908*, 168369.
(81) Skees, A. J.; Foppe, K. S.; Loganathan, B.; Subedi, B. Contamination profiles, mass loadings, and sewage epidemiology of neuropsychiatric and illicit drugs in wastewater and river waters from a community in the Midwestern United States. *Science of the Total Environment* **2018**, *631-632*, 1457-1464.
(82) Gushgari, A. J.; Venkatesan, A. K.; Chen, J.; Steele, J. C.; Halden, R. U. Long-term tracking of opioid consumption in two United States cities using wastewater-based epidemiology approach. *Water Research* **2019**, *161*, 171-180.
(83) Croft, T. L.; Huffines, R. A.; Pathak, M.; Subedi, B. Prevalence of illicit and prescribed neuropsychiatric drugs in three communities in Kentucky using wastewater-based epidemiology and Monte Carlo simulation for the estimation of associated uncertainties. *Journal of Hazardous Materials* **2020**, *384*, 121306.
(84) Montgomery, A. B.; Bowers, I.; Subedi, B. Trends in substance use in two United States communities during early COVID-19 lockdowns based on wastewater analysis. *Environmental Science & Technology Letters* **2021**, *8* (10), 890-896.
(85) Driver, E. M.; Bowes, D. A.; Halden, R. U.; Conroy-Ben, O. Implementing wastewater monitoring on American Indian reservations to assess community health indicators. *Science of the Total Environment* **2022**, *823*, 153882.

(86) Luo, J.; Bello, D.; Pagsuyoin, S. Long-term wastewater-based surveillance and impacts of the COVID-19 pandemic on drug use trends in a U.S. Northeast rural town. *Science of the Total Environment* **2023**, *877*, 162806.
(87) Gushgari, A. J.; Driver, E. M.; Steele, J. C.; Halden, R. U. Tracking narcotics consumption at a Southwestern U.S. university campus by wastewater-based epidemiology. *Journal of Hazardous Materials* **2018**, *359*, 437-444.
(88) Bishop, N.; Jones-Lepp, T.; Margetts, M.; Sykes, J.; Alvarez, D.; Keil, D. E. Wastewater-based epidemiology pilot study to examine drug use in the Western United States. *Science of the Total Environment* **2020**, 140697.
(89) Foppe, K. S.; Hammond-Weinberger, D. R.; Subedi, B. Estimation of the consumption of illicit drugs during special events in two communities in Western Kentucky, USA using sewage epidemiology. *Science of the Total Environment* **2018**, *633*, 249-256.
(90) Subedi, B.; Kannan, K. Occurrence and fate of select psychoactive pharmaceuticals and antihypertensives in two wastewater treatment plants in New York State, USA. *Science of the Total Environment* **2015**, *514*, 273-280.
(91) Pagsuyoin, S.; Luo, J.; Bello, D. Detection in sewage and community consumption of stimulant drugs in northeastern United States. In *Wastewater-Based Epidemiology: Estimation of Community Consumption of Drugs and Diets*, Subedi, B., Burgard, D. A., Loganathan, B. G. Eds.; ACS Symposium Series, Vol. 1319; American Chemical Society, 2019; pp 167-183.
